# Cyclopropenylmethyl Cation: A Concealed Intermediate in Gold(I)‐Catalyzed Reactions

**DOI:** 10.1002/anie.202006245

**Published:** 2020-08-10

**Authors:** Mathis Kreuzahler, Gebhard Haberhauer

**Affiliations:** ^1^ Institut für Organische Chemie Universität Duisburg-Essen Universitätsstraße 7 45117 Essen Germany

**Keywords:** C−C coupling, DFT calculations, enynes, gold catalysis, vinyl cations

## Abstract

The last years have witnessed many gold‐catalyzed reactions of alkynes. One of the most prominent species in the reaction of two alkyne units is the vinyl‐substituted gold vinylidene intermediate. Here, we were able to show that the reaction of a haloacetylene and an alkyne proceeds via a hitherto overlooked intermediate, namely the cyclopropenylmethyl cation. The existence and relative stability of this concealed intermediate is verified by quantum chemical calculations and ^13^C‐labeling experiments. A comparison between the cyclopropenylmethyl cation and the well‐known vinylidene intermediate reveals that the latter is more stable only for smaller cycles. However, this stability reverses in larger cycles. In the case of the smallest representative of both species, the vinylidene cation is the transition state en route to the cyclopropenylmethyl cation. The discovery of this intermediate should help to get a deeper understanding for gold‐catalyzed carbon–carbon bond‐forming reactions of alkynes. Furthermore, since enynes can be formed from the cyclopropenylmethyl cation, the inclusion of this intermediate should enable the development of new synthetic methods for the construction of larger cyclic halogenated and non‐halogenated conjugated enyne systems.

## Graphical abstract

**A more thorough investigation** of the gold(I)‐catalyzed haloalkynylation of arylalkynes was conducted. ^13^C‐Labeling experiments and quantum chemical calculations reveal a hitherto overlooked intermediate, namely the cyclopropenylmethyl cation. Consideration of this intermediate should facilitate the development of new synthetic methods for the construction of larger cyclic conjugated enyne systems.

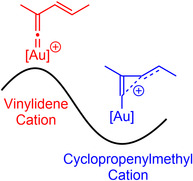

## Introduction

**Scheme 1:**
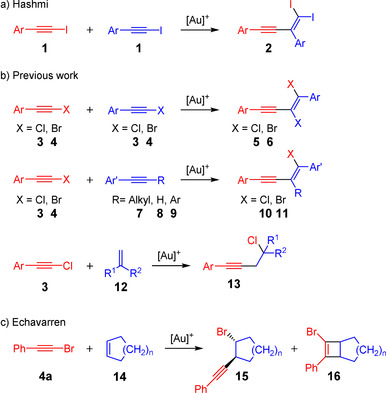
Gold(I)‐catalyzed haloalkynylation of alkynes and alkenes.

In the field of homogenous gold catalysis,^1^, ^2^, ^3^, ^4^, ^5^ alkynes represent one of the most important substance classes.^6^, ^7^ Unlike other transition‐metal‐catalyzed reactions,^8^, ^9^ the alkyne unit is usually consumed during gold‐catalyzed reactions.^6^, ^7^ However, in recent years a few exceptions to this have been found.^10^, ^11^, ^12^, ^13^, ^14^, ^15^, ^16^, ^17^, ^18^, ^19^ Gold‐catalyzed reactions of haloalkynes^20^ make up by far the greatest number of cases.^10^, ^11^, ^12^, ^13^, ^15^ For example, Hashmi et al. were able to show that the dual gold‐catalyzed^21^, ^22^, ^23^, ^24^ reaction of iodoarylacetylenes **1** leads via a head‐to‐tail dimerization to the cross‐conjugated enyne products **2** (Scheme 1 a).^15^ Recently, we presented the dimerization of chloro‐ and bromoarylacetylenes (**3** and **4**) to yield the corresponding head‐to‐head coupling products **5** and **6** (Scheme 1 b, top).^13^ In another study, this reaction was extended to the addition of haloarylacetylenes to alkynes **7**–**9**, whereby the *trans* addition enyne products **10** and **11**, respectively, are formed (Scheme 1 b, middle).^11^

Instead of alkynes, the usage of alkenes as coupling partners with haloacetylenes has also been described.^10^, ^12^, ^25^, ^26^ Here, the formation of the product strongly depends on the gold catalyst and the nature of the alkene reactant. The reaction of 1,2‐disubstituted alkenes and gold carbene complexes leads to the corresponding [2+2] cycloaddition products.^26^ If, however, gold complexes with phosphine ligands and 1,1‐disubstituted alkenes **12** are employed, the 1,2‐chloroalkynylation products **13** are formed (Scheme 1 b, bottom).^25^ The reaction of (bromoethynyl)benzene (**4 a**) and cyclic alkenes **14** leads to both bromoalkynylation products **15** and [2+2] cycloaddition products **16** (Scheme 1 c).^12^

In this study, we thoroughly examine the mechanism of the gold(I)‐catalyzed 1,2‐haloalkynylation reaction of alkynes and alkenes via ^13^C‐labeling experiments and quantum chemical calculations. We demonstrate that—unlike the current hypothesis—the formation of the products always proceeds via a head‐to‐tail addition. The formation of the head‐to‐head products only takes place in the later course of the reaction via the generation of a cyclopropenylmethyl cation. Surprisingly, when vinyl‐substituted, this intermediate is more stable than the well‐established vinylidene cation, which is preferred only in specific cases (e.g. small cycles).

## Results and Discussion

### ^13^C‐Labeling Experiments and Quantum Chemical Calculations

**Scheme 2:**
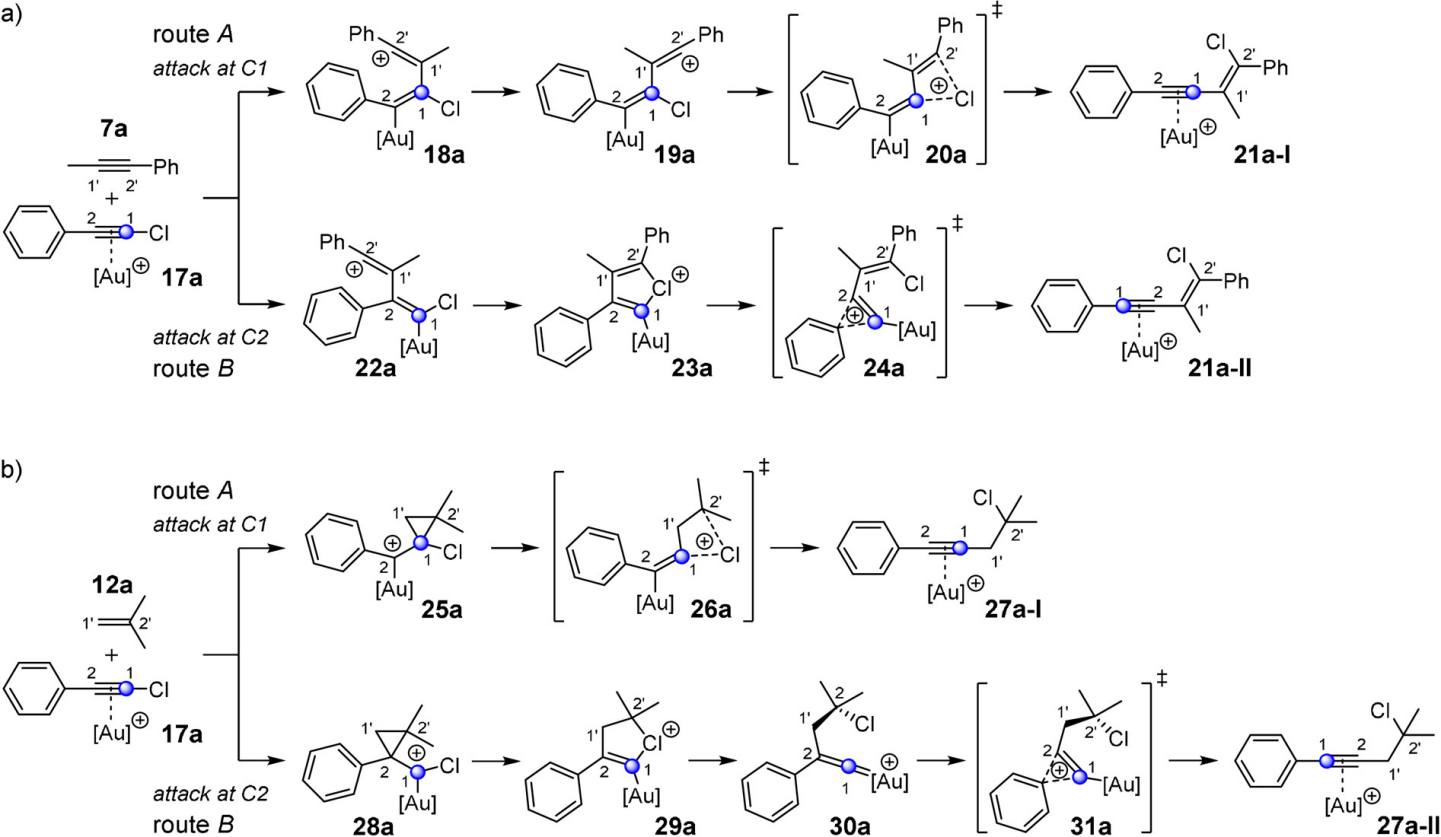
Proposed mechanisms for the gold(I)‐catalyzed 1,2‐chloroalkynylation of arylalkyne **7 a** (a) and alkene **12 a** (b). The reactions proceed via an attack at either carbon atom C1 (route A) or C2 (route B) of the alkyne complex **17 a**. The labeled carbon atoms are highlighted in blue.

In the first step, we wanted to compare the mechanism for the 1,2‐haloalkynylation of alkynes and alkenes. Therefore, we considered a labeled chloroacetylene as a model compound. The labeled carbon atoms are highlighted in blue in Scheme 2. The following mechanistic pathways for the 1,2‐haloalkynylation of alkynes and alkenes have been formerly proposed (Scheme 2):^11^, ^12^, ^13^, ^25^ The addition of the gold complex **17 a** to the alkyne **7 a** can take place via 1,1′‐ (route A) or via 2,1′ (route B) carbon–carbon bond formation. In the first case (route A), the vinyl cation **18 a** is formed. After rotation along the C1–C1′ axis, the thus formed vinyl cation **19 a** leads to the enyne complex **21 a‐I** via a 1,3‐chlorine shift. The labeled carbon atom is now directly attached to the alkenyl unit (Scheme 2 a). Starting from the vinyl cation **22 a** in route B, the chloronium ion **23 a** is formed, which is then transformed into the enyne complex **21 a‐II** via rearrangement of the phenyl group. The labeled carbon is now directly linked to the phenyl group, that is, the position of the labeled atom in the alkyne has been swapped relative to the labeled position in the initial alkyne gold complex **17 a**.

Similar pathways can be described for the addition to 1,1‐disubstituted alkenes **12** (Scheme 2 b). The addition of isobutene (**12 a**) to the carbon atom C1 (route A) of the gold complex **17 a** leads to the alkyne product **27 a‐I** via a 1,3‐chlorine shift, whereas the attack at the carbon atom C2 (route B) of **17 a** results in the formation of the chloronium ion **29 a**. After the formation of the cationic vinylidene intermediate **30 a**, a subsequent aryl shift delivers the alkyne complex **27 a‐II**.

**Scheme 3:**
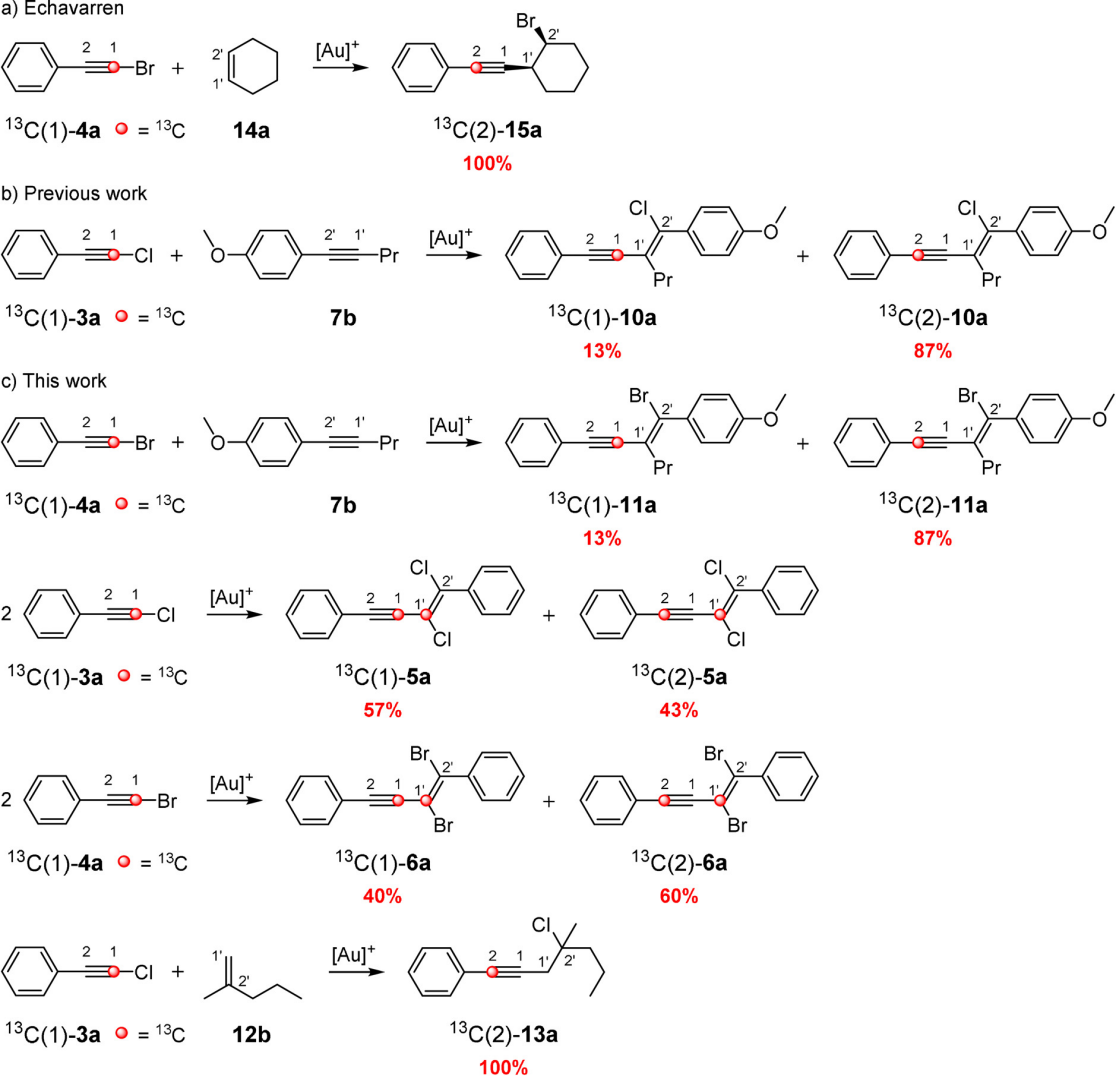
Investigation of the reaction mechanism of the gold(I)‐catalyzed 1,2‐haloalkynylation of arylalkynes and alkenes with ^13^C‐labeled starting materials. As ligands for the gold(I) complexes, *t*BuXPhos and JohnPhos were used.

Replacing the hypothetically labeled atoms (blue carbon atoms in Scheme 2) by ^13^C‐labeled atoms (red carbon atoms in Scheme 3) should help to determine which of the routes (routes A and B) is taken. Echavarren et al. were able to show that in the case of the gold(I)‐catalyzed reaction of ^13^C‐labeled (bromoethynyl)benzene (^13^C(1)‐**4 a**) and cyclohexene (**14 a**) (Scheme 3 a), the addition solely proceeds via an attack at the carbon atom C2 (analogous to route B in Scheme 2 b): The 100 % rearranged product ^13^C(2)‐**15 a** is formed via an aryl shift from a vinylidene cation that originated from a bromonium ion.^12^ Recently, we were able to demonstrate that the gold(I)‐catalyzed addition of ^13^C‐labeled (chloroethynyl)benzene (^13^C(1)‐**3 a**) to **7 b** delivers two products (^13^C(1)‐**10 a** and ^13^C(2)‐**10 a**) in a ratio of 13:87 (Scheme 3 b).^11^ On the basis of the accepted mechanisms (Scheme 2 a), we previously assumed that both routes (A and B in Scheme 2 a) are passed through, whereby route B is slightly energetically favored compared to route A.

To examine which parameters affect the preference for either route, we have conducted a further series of ^13^C‐labeling experiments (Scheme 3 c). JohnPhos[Au(NCMe)]SbF_6_ was used as gold(I) catalyst. Instead of ^13^C(1)‐**3 a**, we used the ^13^C‐labeled bromoacetylene ^13^C(1)‐**4 a** for the addition to the alkyne **7 b**. Furthermore, we have examined the gold(I)‐catalyzed dimerization of bromo‐ and chloroarylacetylenes. Finally, we investigated the addition of ^13^C‐labeled chloroacetylene ^13^C(1)‐**3 a** to the alkene **12 b**. It turns out that in the case of the addition to **7 b**, the exchange of chloro‐ by bromoacetylene results in no change of the product ratio of ^13^C(1)‐**11 a** and ^13^C(2)‐**11 a** (Scheme 3 c) compared to that of ^13^C(1)‐**10 a** and ^13^C(2)‐**10 a** (Scheme 3 b). For the gold(I)‐catalyzed dimerization of chloroacetylene ^13^C(1)‐**3 a**, we obtained a product ratio of 57:43 for ^13^C(1)‐**5 a** and ^13^C(2)‐**5 a**. The analogous dimerization of bromoacetylene ^13^C(1)‐**4 a** gave the enyne products ^13^C(1)‐**6 a** and ^13^C(2)‐**6 a** in a ratio of 40:60. The addition of ^13^C‐labeled chloroacetylene ^13^C(1)‐**3 a** to the double bond of the 1,1‐disubstituted alkene **12 b** delivered only the rearranged product ^13^C(2)‐**13 a**.

Based on the above mentioned ^13^C‐labeling experiments, we have assumed that the addition of haloacetylenes to alkynes proceeds via both routes (route A and B), whereas the addition to alkenes exclusively occurs via route B.

**Figure 1:**
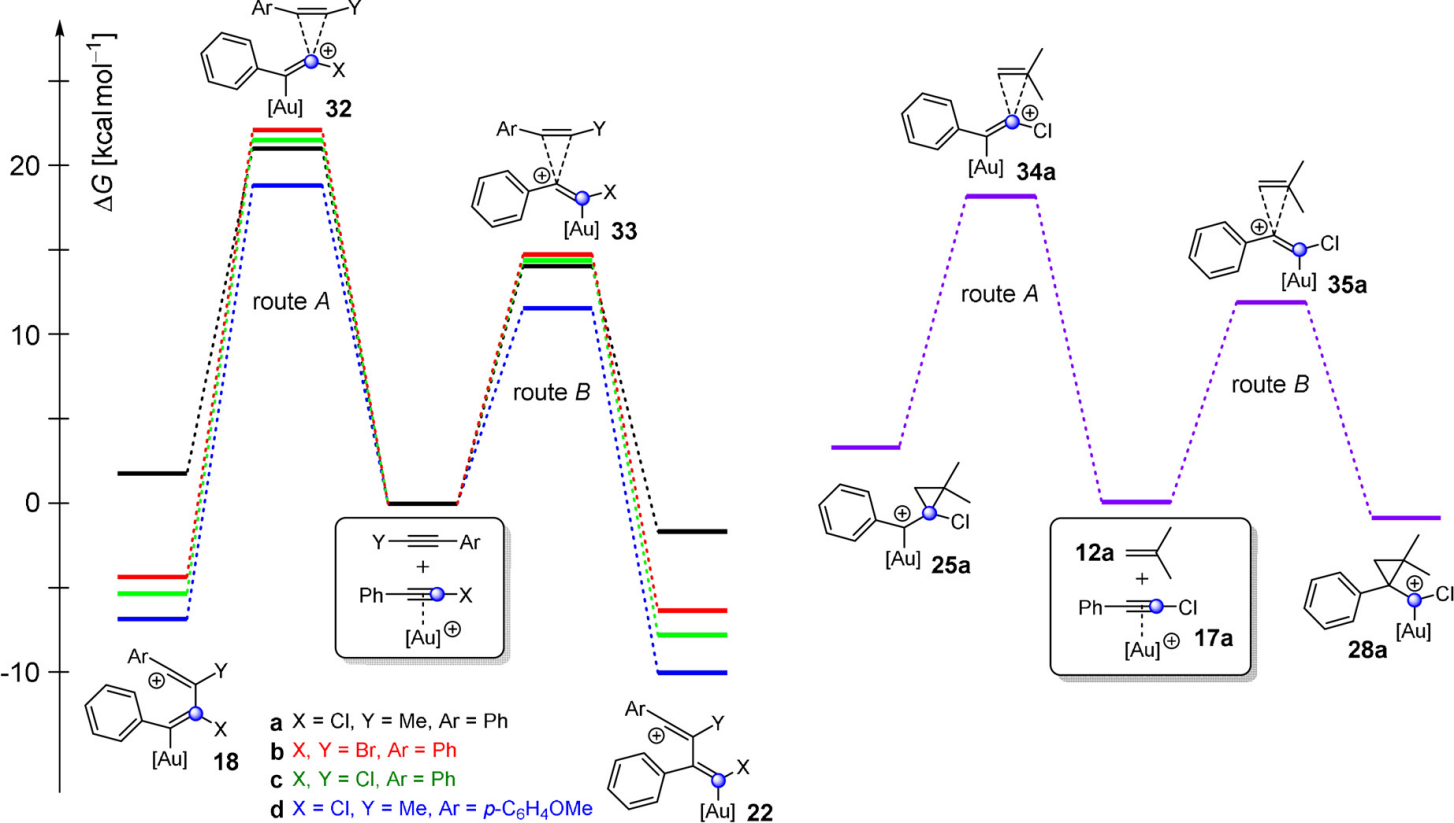
Free energy (Δ*G*) profile for the gold(I)‐catalyzed 1,2‐haloalkynylation of alkynes and alkenes via an attack at carbon atom C1 (route A) or C2 (route B) of the alkyne complex calculated by means of B3LYP‐D3BJ(SMD). [Au]^+^=JohnPhosAu^+^.

To find an explanation for this, we performed quantum chemical calculations (Figure 1). The focus was on the attack at the carbon atom C1 (route A) and C2 (route B), respectively. We used the gold complexes of (chloroethynyl)benzene (**17 a**) (X=Cl in Figure 1) and (bromoethynyl)benzene (**17 b**) (X=Br) as haloacetylenes. Furthermore, 1‐phenyl‐1‐propyne (**7 a**) (Y=Me, Ar=Ph), (chloroethynyl)benzene (**3 a**) (Y=Cl, Ar=Ph), (bromoethynyl)benzene (**4 a**) (Y=Br, Ar=Ph), and 1‐methoxy‐4‐(prop‐1‐yn‐1‐yl)benzene (**7 c**, Y=Me, Ar=*p*‐C_6_H_4_OMe) were employed as alkynes. Isobutene (**12 a**) was used as the alkene reactant. The model compounds correspond or are closely related to the compounds used in the ^13^C‐labeling experiments (Scheme 3). JohnPhos was applied as the ligand of the cationic gold(I) catalyst. B3LYP^27^, ^28^, ^29^ together with the dispersion correction D3BJ^30^ was employed as the method for optimization of the geometrical parameters. The basis set 6‐31G(d) was applied for the elements C, H, O, P, Cl, and Br; Au was calculated with the def2‐TZVP basis set. Additionally, single‐point calculations were performed on the thus obtained structures. Here, B3LYP‐D3BJ was used with the large basis set 6‐311++G(d,p) (for C, H, O, P, Cl, and Br) and def2‐TZVP for Au. To take solvent effects into account, dichloroethane was considered as the reaction solvent by using the SMD^31^ model. The data are summarized in Tables S1–S8 and Figures 1 and S1–S18.

The size of the activation energy and the energy of the formed intermediates differ considerably. Nevertheless, in each system, route B is energetically more favored (6.3–7.3 kcal mol^−1^) compared to route A (Figure 1). The different ratios for the generated ^13^C‐labeled products ranging from 57:43 up to 0:100 (Scheme 3) cannot be explained by comparing the activation energies for the rate‐determining step of the two routes. For all systems, only a single product (via route B) is expected for a ΔΔ*G* value of 6.3–7.3 kcal mol^−1^. If any, one would anticipate a product mixture for the addition of alkene **12 a** to the gold complex **17 a** showing the smallest energy difference (6.3 kcal mol^−1^). However, only one product is formed in this case (Scheme 3 c).

### Detailed Quantum Chemical Calculations for the Fate of the Intermediary Formed Cations

**Figure 2:**
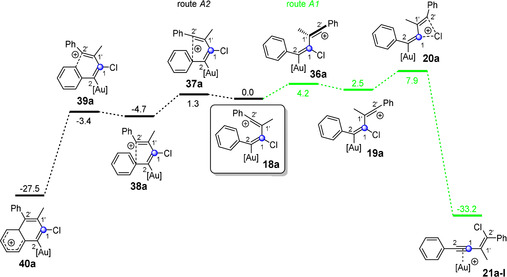
Possible reaction pathways (routes A1 and A2) of the vinyl cation **18 a**, which is formed via 1,1′‐linking during the gold(I)‐catalyzed reaction of chloroacetylene **3 a** and alkyne **7 a**. The indicated free energy (Δ*G* in kcal mol^−1^) values were calculated using B3LYP‐D3BJ(SMD) and are relative to the vinyl cation **18 a**. [Au]^+^=JohnPhosAu^+^.

**Figure 3:**
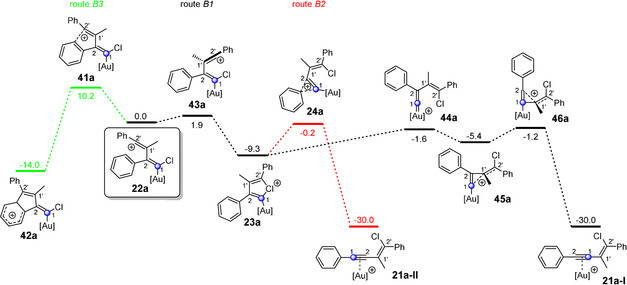
Possible reaction pathways (routes B1, B2, and B3) of the vinyl cation **22 a**, which is formed via 2,1′‐linking during the gold(I)‐catalyzed reaction of chloroacetylene **3 a** and alkyne **7 a**. The indicated free energy (Δ*G* in kcal mol^−1^) values were calculated using B3LYP‐D3BJ(SMD) and are relative to the vinyl cation **22 a**. [Au]^+^=JohnPhosAu^+^.

**Figure 4:**
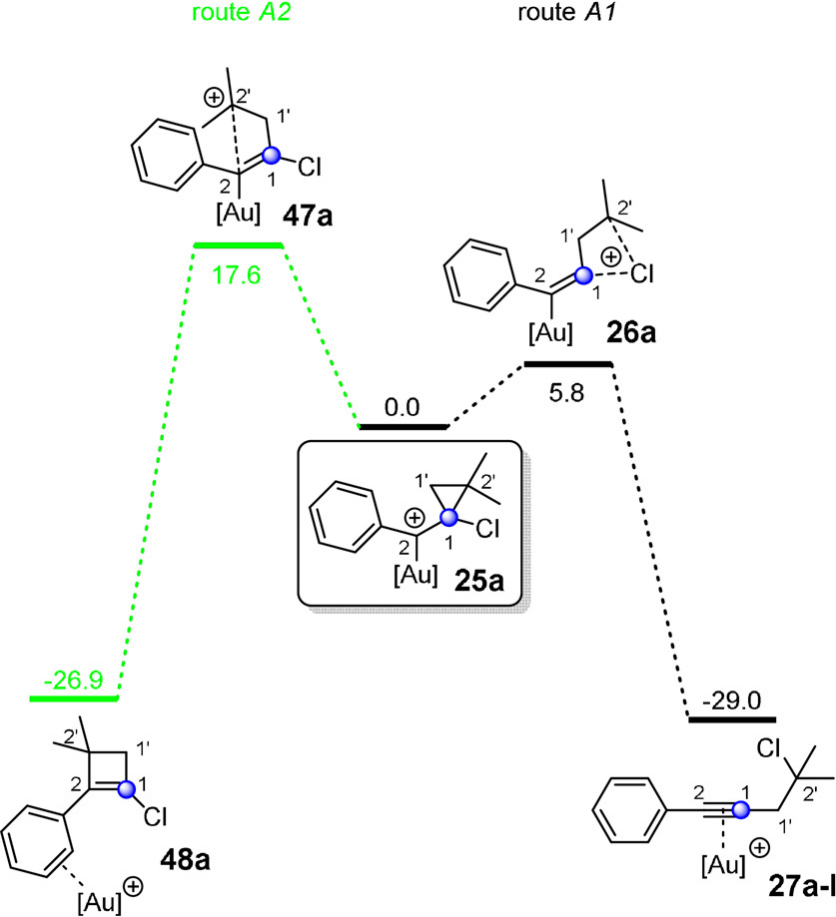
Possible reaction pathways (routes A1 and A2) of the cyclopropylmethyl cation **25 a**, which is formed via an attack at the carbon atom C1 in the gold(I)‐catalyzed reaction of chloroacetylene **3 a** and alkene **12 a**. The indicated free energy (Δ*G* in kcal mol^−1^) values were calculated using B3LYP‐D3BJ(SMD) and are relative to the cyclopropylmethyl cation **25 a**. [Au]^+^=JohnPhosAu^+^.

**Figure 5:**
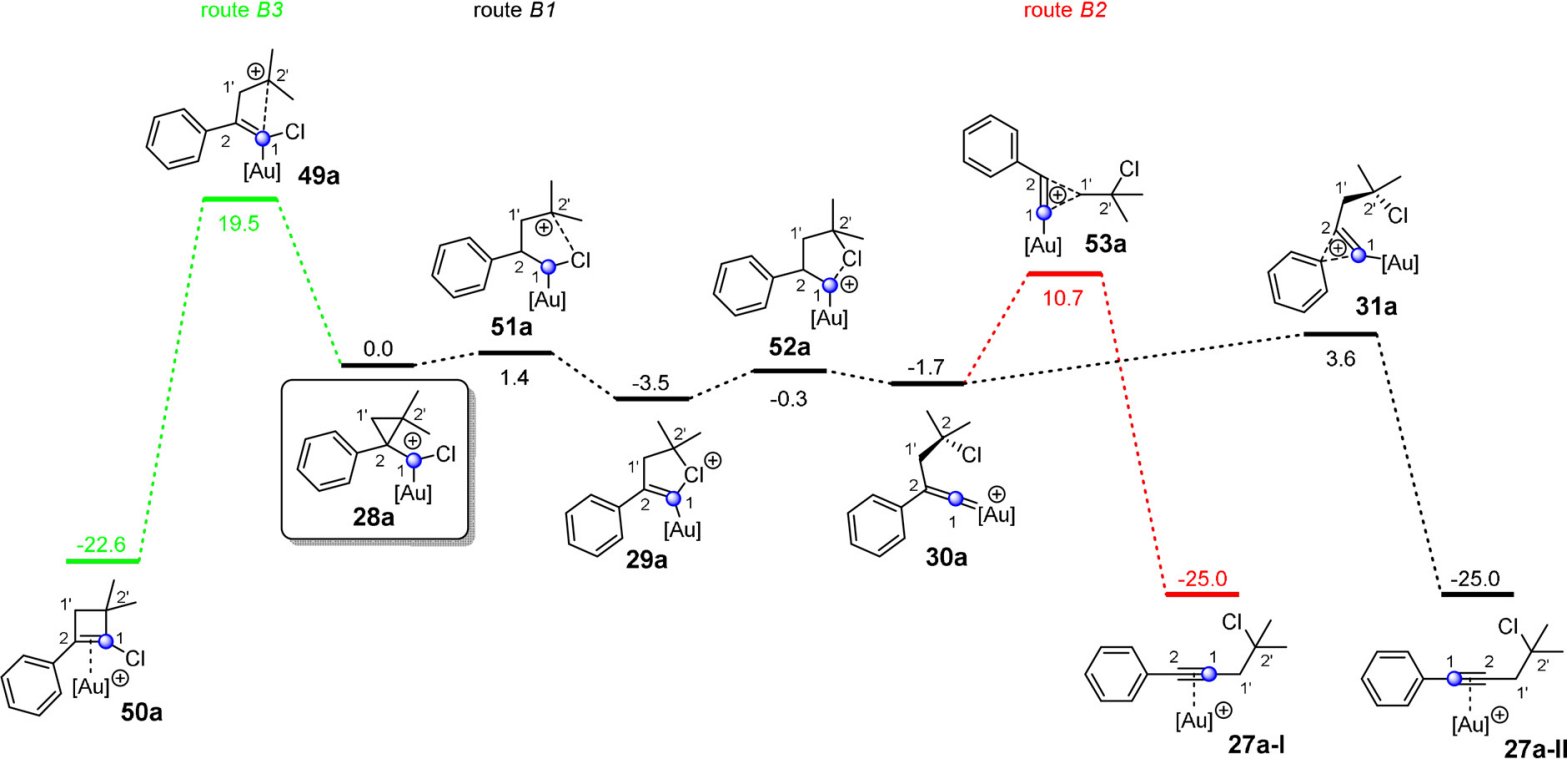
Possible reaction pathways (route B1, B2 and B3) of the cyclopropylmethyl cation **28 a**, which is formed via an attack at the carbon atom C2 in the gold(I)‐catalyzed reaction of chloroacetylene **3 a** and alkene **12 a**. The indicated free‐energy (Δ*G* in kcal mol^−1^) values were calculated using B3LYP‐D3BJ(SMD) and are relative to the cyclopropylmethyl cation **28 a**. [Au]^+^=JohnPhosAu^+^.

To figure out why the results of the ^13^C‐labeling experiments do not match the quantum chemical calculations, we re‐examined the possible reaction pathways for the intermediary formed cations more accurately via quantum chemical calculations. Therefore, we rotated the intermediary formed cations **18** and **22** (see Figure 1) along the initially formed bond and explored the thus newly formed cations. In addition, we searched for other intermediates that can be formed via further rotation and rearrangement processes. As model compounds for the gold(I)‐catalyzed addition of a haloacetylene to an alkyne, chlorophenylacetylene (**3 a**) and 1‐phenyl‐1‐propyne (**7 a**) were chosen, while isobutene (**12 a**) was employed as the alkene reactant. The fates of both reactions are depicted in Figures 2–5.

First, we have a look at the vinyl cation **18 a**, which is formed via 1,1′‐linking (route A, Scheme 2 a) in the gold(I)‐catalyzed reaction of chlorophenylacetylene (**3 a**) and 1‐phenyl‐1‐propyne (**7 a**). The calculations reveal that the vinyl cation **18 a** can take two pathways (Figure 2): The first is the previously described pathway to form enyne complex **21 a‐I** via a 1,3‐chlorine shift; the highest activation energy amounts to 7.9 kcal mol^−1^ (route A1 (green) in Figure 2). A considerably lower activation energy is found for the rotation along the C1–C1′ axis in the other direction (1.3 kcal mol^−1^, route A2 (black) in Figure 2). Here, the bicyclic cation **40 a** is formed in a two‐step mechanism via a Friedel–Crafts type alkylation of **38 a**. After rearomatization and subsequent protodesauration, the product would correspond to a chloronaphthalene derivative. That means that the 1,1′‐linking (route A) of the gold‐catalyzed reaction of chlorophenylacetylene (**3 a**) and 1‐phenyl‐1‐propyne (**7 a**) does not give the enyne but a naphthalene system. However, we have never identified such a product in our experiments. Thus, the formation of the enyne product **21 a‐I** via a 1,1′‐linking can now be excluded as an actual reaction pathway for the gold(I)‐catalyzed reaction of chlorophenylacetylene (**3 a**) and 1‐phenyl‐1‐propyne (**7 a**).

Let us now consider the vinyl cation **22 a**, which is formed via 2,1′‐linking (route B, Scheme 2 a) in this reaction (Figure 3). Here, we identified three possible reaction pathways. The first one (route B3) leads via a Friedel–Crafts type reaction to the indene derivative **42 a**. This route shows the highest activation energy of the three with 10.2 kcal mol^−1^, which is why this reaction pathway is unlikely to be passed through. The other two reaction paths proceed via the chloronium ion **23 a**. Now, two rearrangement processes are conceivable. The shift of the aryl group can lead to the enyne complex **21 a‐II** (route B2), whereas route B1 proceeds via the cyclopropenylmethyl cation **45 a** to the corresponding enyne complex **21 a‐I**. The transition state to this highly unusual cation **45 a** represents the vinylidene cation **44 a**, which is not stabilized by the chlorine atom. The enyne complex **21 a‐I** is then formed via opening of the C2−C1′ bond. A closer look at the enyne complex **21 a‐I** reveals that the relative orientation of the carbon atoms C1 and C2 differs in routes B1 and B2. The difference in the activation energy for routes B1 (**24 a**) and B2 (**46 a**) amounts to 1.0 kcal mol^−1^ in favor of route B1. This slight difference in energy should result in both reaction pathways being passed through. In fact, this assumption agrees better with our experimental observations (Scheme 3 b).

For the fate of the cyclopropylmethyl cation **25 a**, which is formed via an attack at the carbon atom C1 of the gold alkyne complex **17 a** (see Scheme 2 b, route A), two reaction pathways were found (Figure 4); both have already been described in the literature.^25^ In one case, the [2+2] cycloaddition product **48 a** is generated (route A2); in the other case the alkyne product **27 a‐I** is formed (route A1). Here, route A1 is clearly preferred.

Starting from the cyclopropylmethyl cation **28 a**, which is formed via an attack at the C2 atom of the gold alkyne complex **17 a** (see Scheme 2 b, route B), three reaction pathways were localized (Figure 5). The first and highest activation energy pathway is route B3, which leads to the [2+2] cycloaddition product **50 a**. Routes B1 and B2 both proceed via the chloronium ion **29 a** and the vinylidene cation **30 a**. From there, the outcome of the reaction is determined by two options: The rearrangement of the aryl group leads to the alkyne product **27 a‐II** (route B1), whereas the rearrangement of the isobutyl group delivers the alkyne complex **27 a‐I** (route B2). Here again, both products **27 a‐I** and **27 a‐II** differ in their relative orientation of the carbon atoms C1 and C2 in the alkyne unit (Figure 5). The most striking difference to the gold(I)‐catalyzed reaction with the alkyne (see Figure 3) is that all cyclic structures (**31 a** and **53 a**) are transition states, while the vinylidene cation **30 a** is now an intermediate. The difference in the activation energy for the two transition states **31 a** and **53 a** is so high (7.1 kcal mol^−1^) that the gold(I)‐catalyzed reaction of a chloroacetylene and a 1,1‐disubstituted alkene should only proceed via route B1. This key finding matches our experimental results, as the ^13^C‐labeled carbon atom can be found with 100 % directly next to the aromatic unit.

**Figure 6:**
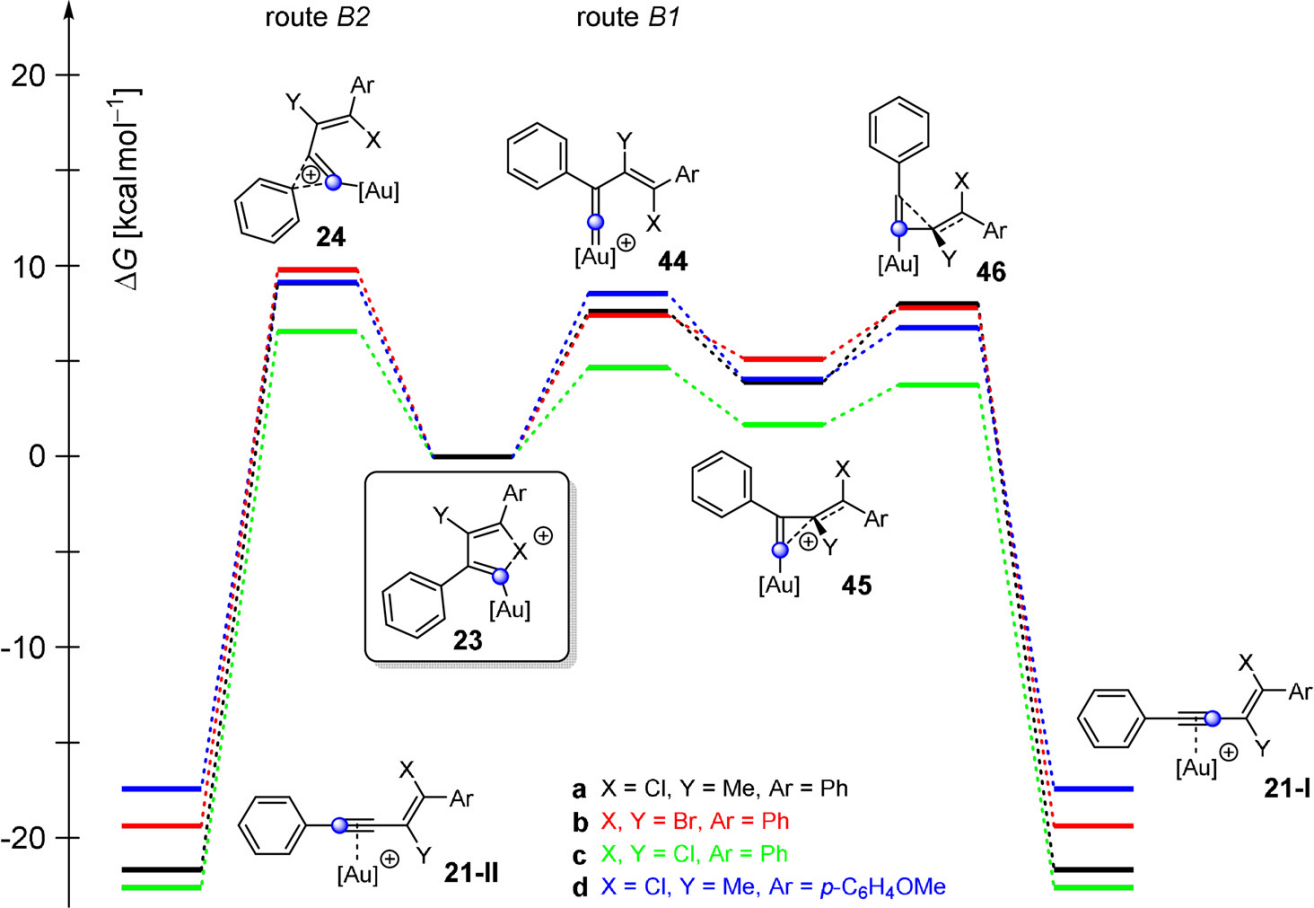
Possible reaction pathways of the halonium ion **23** via formation of the cyclopropenylmethyl cation **45** (route B1) or via aryl shift (route B2) to the enyne complex **21‐I** and **21‐II**, respectively. The indicated free‐energy (Δ*G* in kcal mol^−1^) values were calculated using B3LYP‐D3BJ(SMD) and are relative to the halonium ion **23**. [Au]^+^=JohnPhosAu^+^.

As mentioned above, the relative ratio of the alkyne complexes **21 a‐I** and **21 a‐II** for the 1,2‐chloroalkynylation of 1‐phenyl‐1‐propyne (**7 a**) depends on the energy of the transition states **24 a**, **44 a**, and **46 a** (Figure 3). During our experimental studies, we realized that the final ratio of the ^13^C‐labeled products depends on the nature of the employed alkyne (see Scheme 3). To verify whether this observation is also reflected in the quantum chemical calculations, we investigated the transition states **24**, **44**, and **46** as well as intermediates **23** and **45** for different systems by computational methods (Figure 6 and Tables S1–S8). Indeed, the experimental trend can also be found in our calculations; for example, the transition state **44 c** (route B1) for the gold(I)‐catalyzed dimerization of chlorophenylacetylene (**3 a**) is more stable by 1.9 kcal mol^−1^ than the corresponding transition state **24 c** of route B2. Therefore, we expected the preferred formation of the enyne product **21 c‐I**, which corresponds to the gold complex of ^13^C(1)‐**5 a** (see Scheme 3 c). This prediction could be confirmed by ^13^C‐labeling experiments, as more ^13^C(1)‐**5 a** (57 %) was formed (see Scheme 3 c). Furthermore, this ratio should shift towards the **21‐II** enyne product (via route B2) with a decreasing energy difference between both transition states **44** (**46**) and **24**. That should also apply to the gold(I)‐catalyzed dimerization of bromophenylacetylene (**4 a**) and to the gold(I)‐catalyzed addition of chloro‐ and bromophenylacetylene (**3 a** and **4 a**) to alkyne **7 b** (Scheme 3). Please note, we used 1‐methoxy‐4‐(prop‐1‐yn‐1‐yl)benzene as a representative of **7 b** for the calculations. In fact, our ^13^C‐labeling experiments showed a decrease in the formation of the ^13^C(1)‐enyne product for these reactions (40 % for ^13^C(1)‐**6 a**, 13 % for ^13^C(1)‐**10 a**, and 13 % for ^13^C(1)‐**11 a**).

**Figure 7:**
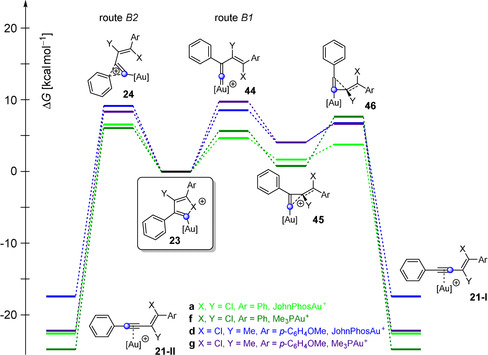
Possible reaction pathways of the halonium ion **23** with different phosphine ligands via formation of the cyclopropenylmethyl cation **45** (route B1) or via aryl shift (route B2) to the enyne complexes **21‐I** and **21‐II**, respectively. The indicated free energy (Δ*G* in kcal mol^−1^) values were calculated using B3LYP‐D3BJ(SMD) and are relative to the halonium ion **23**. [Au]^+^=JohnPhosAu^+^ or Me_3_PAu^+^.

**Scheme 4:**
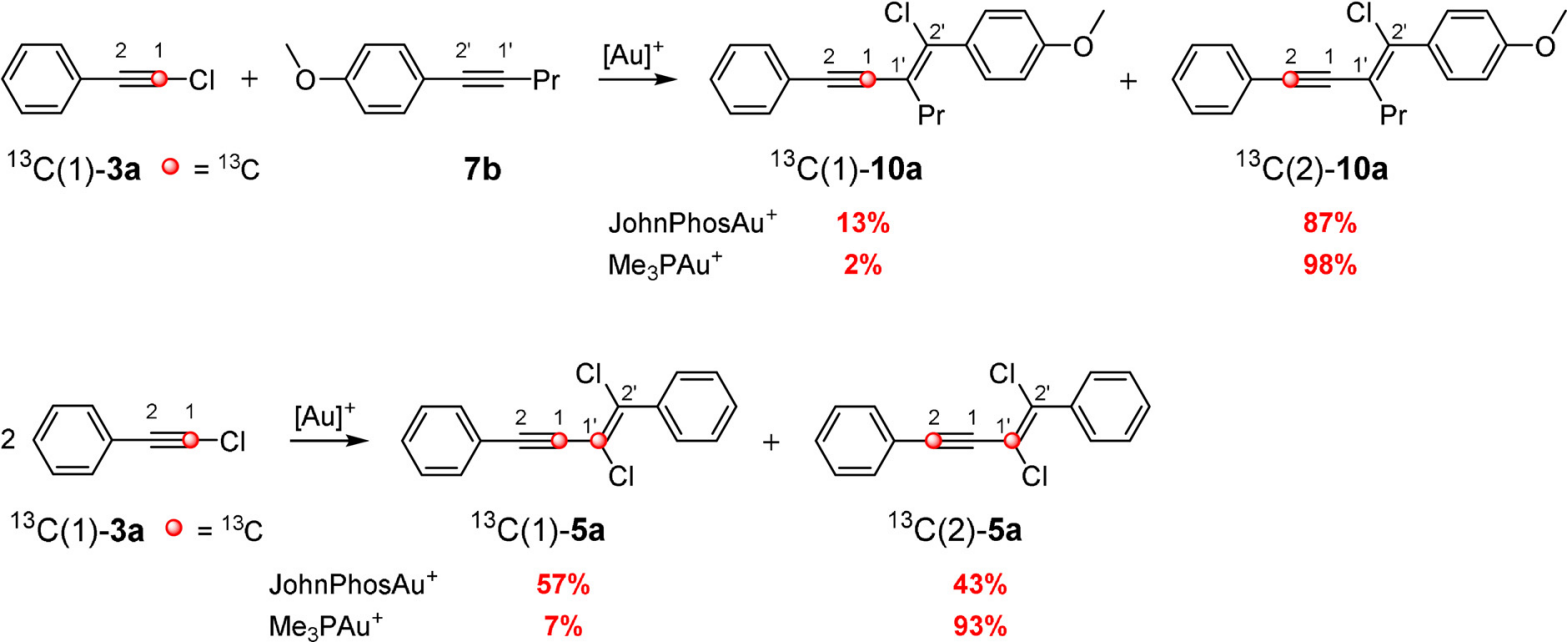
Comparison of the 1,2‐haloalkynylation of arylalkynes using ^13^C‐labeled starting materials and gold catalysts having different phosphine ligands.

For the further investigation of the mechanistic course of the haloalkynylation reaction, we tried to validate whether the ratio of **21‐I** (corresponds to ^13^C(1)‐enyne) and **21‐II** (corresponds to ^13^C(2)‐enyne) is also dependent on the nature of the ligand of the gold complex. Therefore, we examined the gold‐catalyzed dimerization of ^13^C‐labeled chlorophenylacetylene (^13^C(1)‐**3 a**) and the addition of ^13^C(1)‐**3 a** to the alkyne **7 b** by replacing the JohnPhos ligand by PMe_3_ (Scheme 4 and Figure 7). The catalytic species Me_3_PAu^+^ that was used for the calculations was generated during the experiment in situ by mixing Me_3_PAuCl and AgSbF_6_.

When we used Me_3_PAu^+^ in our experimental studies, both reactions showed an increase in the ratio of the molecules corresponding to **21‐II** of up to 98 % (^13^C(2)‐**5 a** and ^13^C(2)‐**10 a**, Scheme 4). The shift towards **21‐II** could also be confirmed by calculations. The calculated activation energy of route B1 is higher for Me_3_PAu^+^ than for JohnPhosAu^+^ (Figure 7). For example, in the case of the dimerization with JohnPhosAu^+^, route B1 is more favored by 1.9 kcal mol^−1^ over route B2. This selectivity is reversed by using Me_3_PAu^+^ as catalyst so that route B2 is favored by 1.6 kcal mol^−1^. This inversion is reflected in the experimental results (Scheme 4).

### The Nature of Cyclopropenylmethyl Cations as Intermediates in Gold(I)‐Catalyzed Reactions

**Figure 8:**
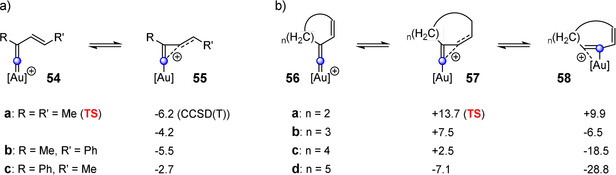
a) Relative energies (Δ*E* in kcal mol^−1^) of vinylidene cations **54** and cyclopropenylmethyl cations **55**. If not stated otherwise, the data were calculated via B2PLYP‐D3(SMD)/B3LYP‐D3BJ. b) Relative energies (Δ*E* in kcal mol^−1^) of cyclic vinylidene cations **56**, cyclopropenylmethyl cations **57**, and alkyne complexes **58** calculated using B2PLYP‐D3(SMD)/B3LYP‐D3BJ.

**Figure 9:**
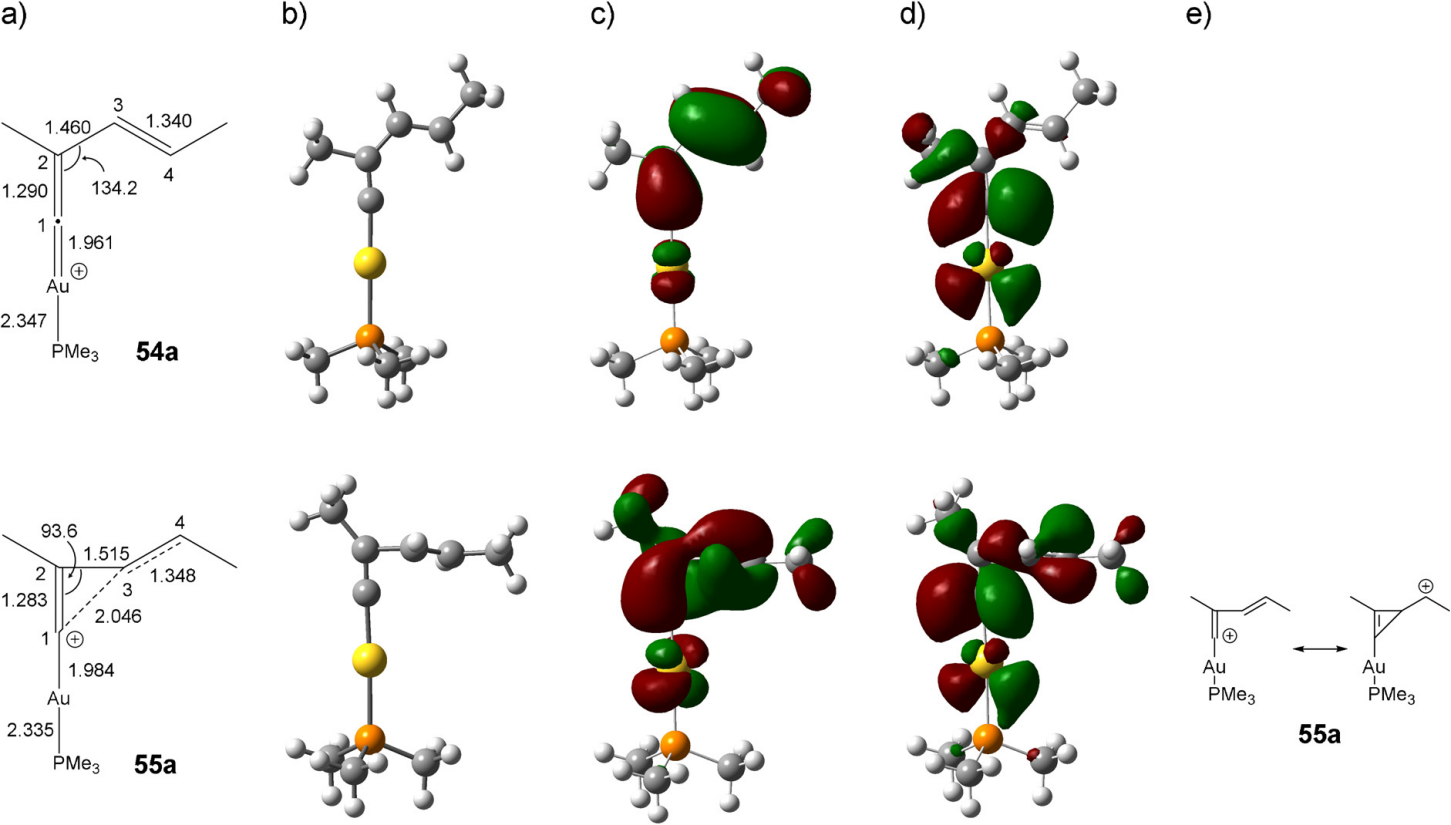
Distances [Å] and angles [°] (a), molecular structures (b) as well as HOMOs (c) and LUMOs (d) of the vinylidene cation **54 a** and cyclopropenylmethyl cation **55 a** calculated using B3LYP‐D3BJ/6‐311++G(d,p),aug‐cc‐pVTZ‐PP+ECP. e) Resonance structures of the cyclopropenylmethyl cation **55 a**.

As shown above, the commonly described cationic vinylidene intermediate is not an intermediate for the haloalkynylation of alkynes but a transition state (Figure 3). Please note that this does not apply for the haloalkynylation of alkenes, where it represents a minimum on the potential energy surface (Figure 5). For the reaction of haloalkynes with alkynes, we identified the cyclopropenylmethyl cation and the halonium ion as key intermediates. The latter can be considered as a vinylidene cation stabilized by a halogen atom. This raised the question of how the cationic vinylidene and cyclopropenylmethyl species behave when there is no halogen substitution. Therefore, we calculated the geometries of the simple model compounds **54** and **55** by means of B3LYP‐D3BJ (Figures 8 and 9). As basis sets, 6‐311++G(d,p) (for C, H, and O) and aug‐cc‐pVTZ‐PP (for Au) were applied. Subsequent frequency calculations showed that **54 a** is a transition state, whereas all other stationary points are minima on the potential energy surface. Furthermore, we conducted single‐point calculations using the same basis sets and by means of the double‐hybrid density functional approximation B2PLYP,^32^ which delivers very reliable data even for high‐energy intermediates of reactions involving alkynes.^33^, ^34^, ^35^, ^36^ To determine the solvent effect, B2PLYP single‐point calculations were conducted by using the SMD^31^ model and dichloroethane as the solvent. Additionally, the CCSD(T)^37^ approximation was employed to compute the energy difference between the transition state **54 a** and the intermediate **55 a** (Figure 8).

A comparison of the energy values shows that in all cases the cyclopropenylmethyl cation **55** is the most stable species (Figure 8 a). Please note that the vinylidene cation **54 a** represents a transition state. Thus, the energy difference and the nature of the stationary points (minimum or maximum) strongly depend on the substitution pattern. A phenyl group at the C4 position increases the stability of the cyclopropenylmethyl cation **55 b**, as the positive charge at the carbon atom C4 can be stabilized by the adjacent aromatic system. A phenyl group at the position C2 stabilizes the vinylidene cation **54 c**; however, the cyclopropenylmethyl cation **55 c** remains the most stable intermediate.

The distances and angles of **54 a** and **55 a** obtained by B3LYP‐D3BJ/6‐311++G(d,p),aug‐cc‐pVTZ‐PP are depicted in Figure 9 a. A glance at the values of **55 a** shows that the structure of **55 a** is not an isosceles triangle, as the distances for the bonds C1−C3 and C2−C3 differ significantly. The C2−C3 bond (1.515 Å) is a slightly shorter single bond, whereas the C2−C3 bond (2.046 Å) is relatively long. Bonds of this magnitude can also be found for nonclassical carbocations such as norbornyl cations.^38^ The C1‐C2‐C3 angle of **55 a** is 94° and varies noticeably from the corresponding angle of **54 a** (134°). The length of the C2−C3 bond in **54 a** amounts to 1.460 Å. Therefore, this bond is significantly shorter than that in **55 a** (1.515 Å) due to the conjugation of the two double bonds (C1=C2 and C3=C4). The most pronounced difference in the frontier orbitals of the twp species **54 a** and **55 a** can be found in the LUMOs (Figure 9 d): For **54 a** the largest coefficient is located at the carbon atom C1, while both carbon atoms C1 and C4 in **55 a** show large coefficients. Accordingly, the characterization of the electronic nature of the cyclopropenylmethyl cation **55 a** is best represented by the resonance structures in Figure 9 e.

Our investigations clearly reveal that the cyclopropenylmethyl cation is more stable than the vinylidene cation. However, so far only the cationic vinylidene intermediate has been described in the literature as an important species in the gold‐catalyzed reactions of alkynes.^21^, ^23^, ^24^, ^39^, ^40^, ^41^, ^42^ To shed light on this issue, we calculated the vinylidene cation **56** that often emerges during gold(I)‐catalyzed intramolecular reaction of diynes^6^, ^43^ and compared it with the corresponding cyclopropenylmethyl cation **57** and the rearranged cation **58**, respectively (Figure 8 b). We considered different ring sizes (five‐membered ring with *n*=2 up to the eight‐membered ring with *n*=5). For the five‐membered ring (*n*=2), the cyclopropenylmethyl cation **57 a** was located as a transition state on the potential energy surface and the corresponding rearranged six‐membered ring **58 a** is ca. 10 kcal mol^−1^ higher in energy. A derivative of **57 a** has been discussed as a potential transition state for the formation of a vinylidene and vinyl cation during the dual gold‐catalyzed intramolecular reaction of diynes.^44^ With increasing ring size, the energy of the cyclopropenylmethyl cation **57** decreases and is in the case of *n*=5 (eight‐membered ring) considerably more stable than the corresponding vinylidene cation **56**. Please note that the vinyl cation **58 d** should rather be considered as a gold alkyne π complex for larger cycles. Consequently, it can be expected that larger cycles (eight‐membered rings or larger) facilitate the formation of a cyclopropenylmethyl cation, which can undergo rearrangement to form an enyne gold complex.

## Conclusion

In this work, we thoroughly investigated the gold‐catalyzed reaction of two alkyne units. Unlike the previously assumed mechanism, the rate‐determining step, namely the nucleophilic attack of the gold alkyne complex at the alkyne, does not determine the connectivity of the carbon atoms in the final product. Starting from a halonium ion, two mechanisms are possible: The first mechanism proceeds via the rearrangement of an aryl group, the second via the formation of a cyclopropenylmethyl cation. The thus formed products are identical and can only be distinguished from each other by ^13^C‐labeling. Furthermore, ^13^C‐labeling experiments impressively show that the distribution of these products can be modified by variation of the substituents of the aromatic backbone and the gold catalyst, which is in accordance with our quantum chemical calculations. A closer look at the cyclopropenylmethyl cation reveals that this species is generally more stable than the commonly discussed vinylidene cation. In case of the smallest representative of both species, the vinylidene cation is the transition state en route to the cyclopropenylmethyl cation. The formation of vinylidene cations is only preferred by the incorporation into smaller cyclic systems (five‐ to seven‐membered ring systems). Future strategies could employ this principle to synthesize larger cyclic enyne systems, as enyne gold complexes are always formed from the corresponding cyclopropenylmethyl cation. As the formation of this cation does not necessarily require the presence of a halonium ion, the proof of this key intermediate also questions previously reported mechanisms of the gold‐catalyzed reaction of two terminal alkynes.

## Conflict of interest

The authors declare no conflict of interest.

## Supporting information

As a service to our authors and readers, this journal provides supporting information supplied by the authors. Such materials are peer reviewed and may be re‐organized for online delivery, but are not copy‐edited or typeset. Technical support issues arising from supporting information (other than missing files) should be addressed to the authors.

Supplementary
