# Supplementary material for: Cyclopropenylmethyl Cation: A Concealed Intermediate in Gold(I)‐Catalyzed Reactions
Source: Angew Chem Int Ed Engl. 2020 Aug 10;59(40):17739–49. doi: 10.1002/anie.202006245 (PMC7540476; doi:10.1002/anie.202006245)
Supplement: Supplementary file 1 — Supplementary [file ANIE-59-17739-s001.pdf]

Angewandte Chemie
Eine Zeitschrift der Gesellschaft Deutscher Chemiker

Supporting Information

Cyclopropenylmethyl Cation: A Concealed Intermediate in Gold(I)-Catalyzed Reactions

*Mathis Kreuzahler and Gebhard Haberhauer**

anie_202006245_sm_miscellaneous_information.pdf

Supporting Information

1. **Figures and Tables** S2
2. **^{13}C-Labeling Experiments** S16
3. **Computational Details** S23
4. **Cartesian Coordinates and Absolute Energies for All Calculated Compounds** S24
5. **^{1}H and ^{13}C NMR Spectra of the ^{13}C-Labeled Products** S155
6. **Supporting Information References** S160

1. Figures and Tables

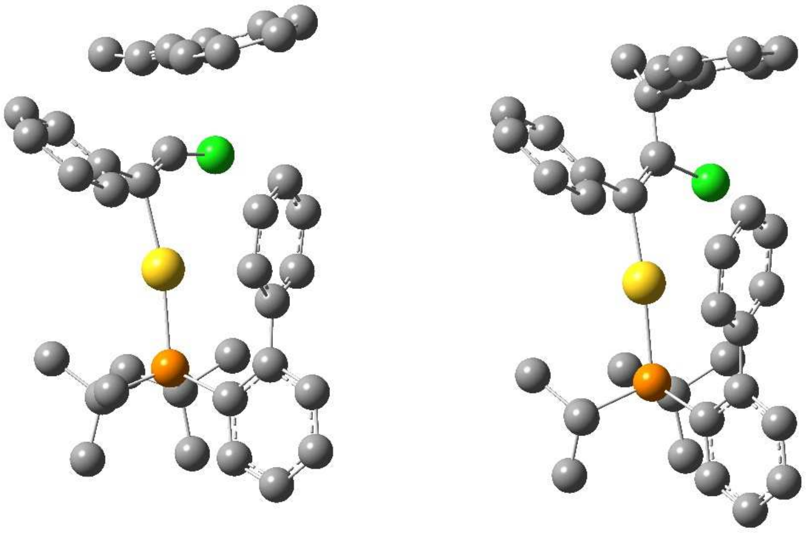

Figure S1. Molecular structure of **32a** (left) and **18a** (right) calculated by means of B3LYP-D3BJ/6-31G(d),def2-TZVP. All hydrogen atoms are omitted for clarity.

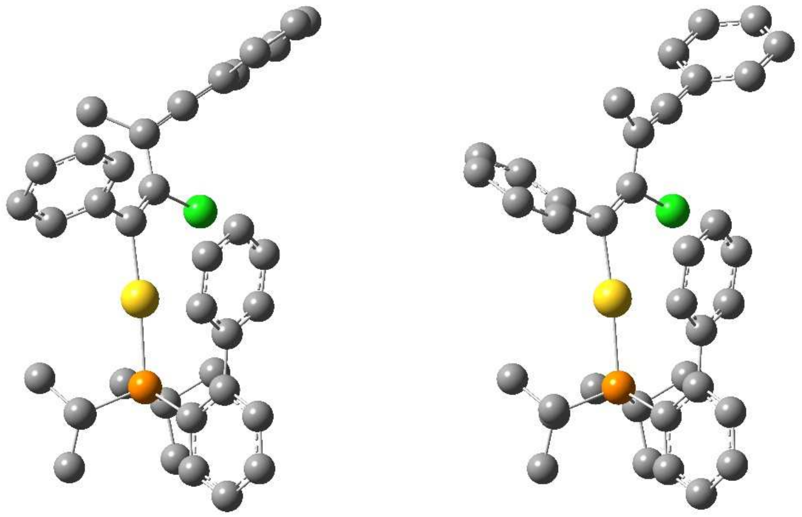

Figure S2. Molecular structure of **36a** (left) and **19a** (right) calculated by means of B3LYP-D3BJ/6-31G(d),def2-TZVP. All hydrogen atoms are omitted for clarity.

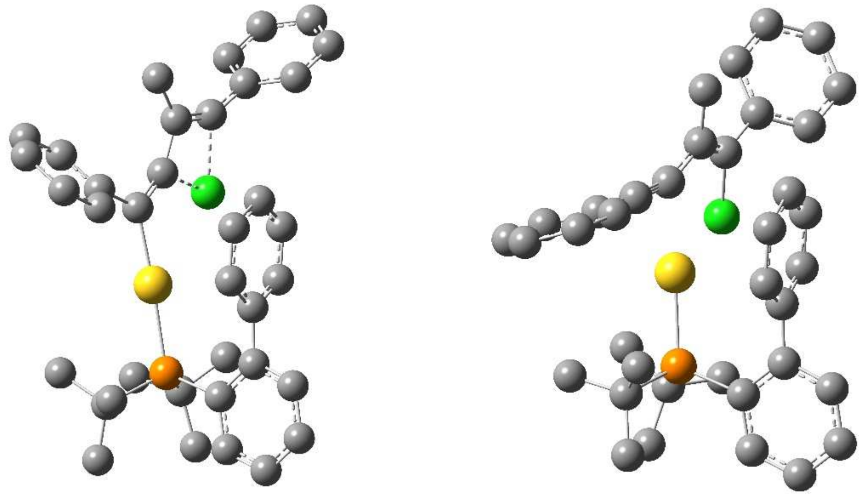

Figure S3. Molecular structure of **20a** (left) and **21a** (right) calculated by means of B3LYP-D3BJ/6-31G(d),def2-TZVP. All hydrogen atoms are omitted for clarity.

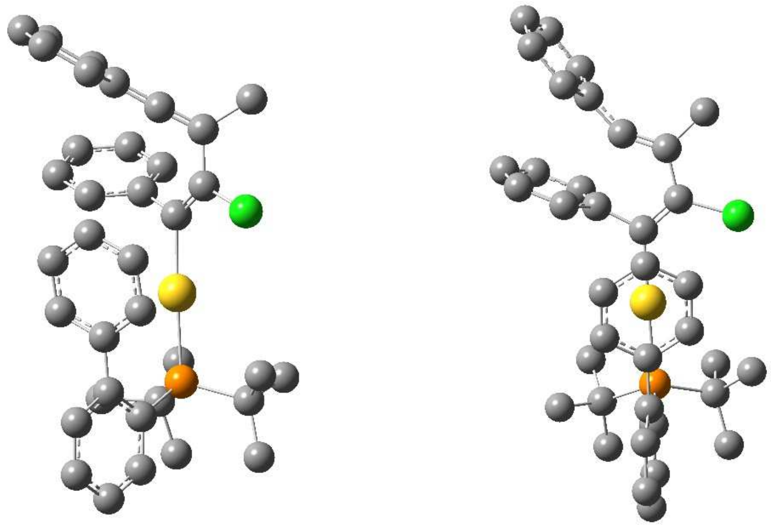

Figure S4. Molecular structure of **37a** (left) and **38a** (right) calculated by means of B3LYP-D3BJ/6-31G(d),def2-TZVP. All hydrogen atoms are omitted for clarity.

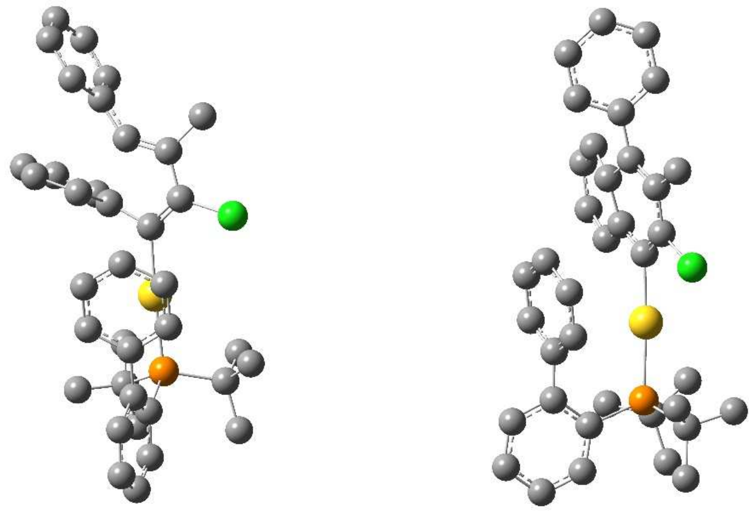

Figure S5. Molecular structure of **39a** (left) and **40a** (right) calculated by means of B3LYP-D3BJ/6-31G(d),def2-TZVP. All hydrogen atoms are omitted for clarity.

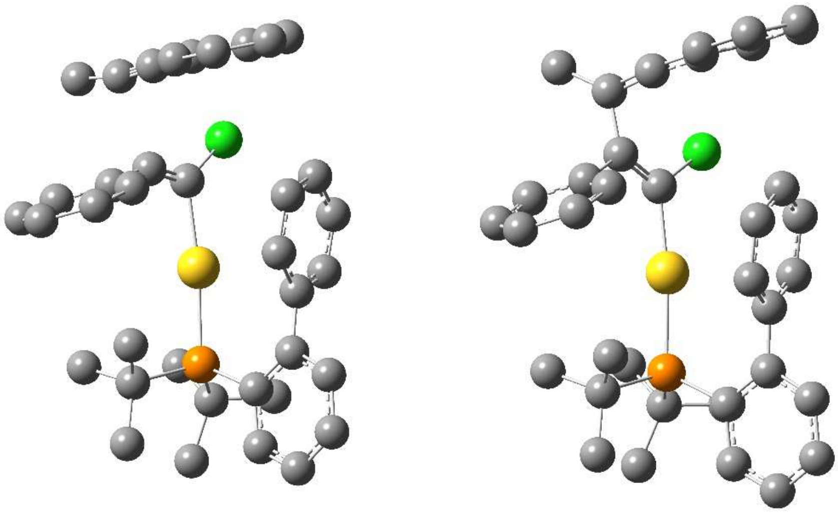

Figure S6. Molecular structure of **33a** (left) and **22a** (right) calculated by means of B3LYP-D3BJ/6-31G(d),def2-TZVP. All hydrogen atoms are omitted for clarity.

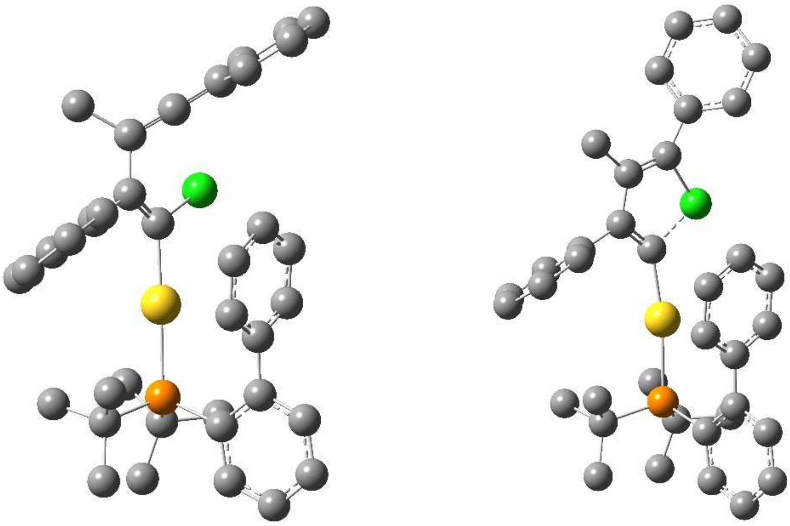

Figure S7. Molecular structure of **43a** (left) and **23a** (right) calculated by means of B3LYP-D3BJ/6-31G(d),def2-TZVP. All hydrogen atoms are omitted for clarity.

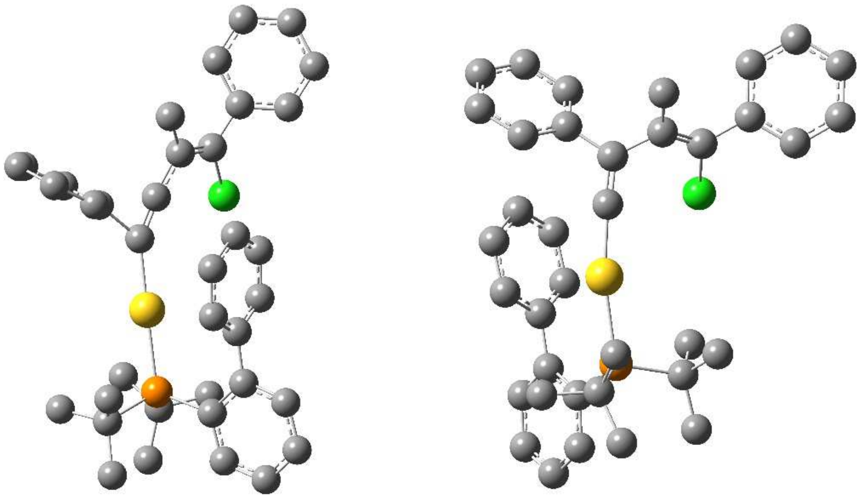

Figure S8. Molecular structure of **24a** (left) and **44a** (right) calculated by means of B3LYP-D3BJ/6-31G(d),def2-TZVP. All hydrogen atoms are omitted for clarity.

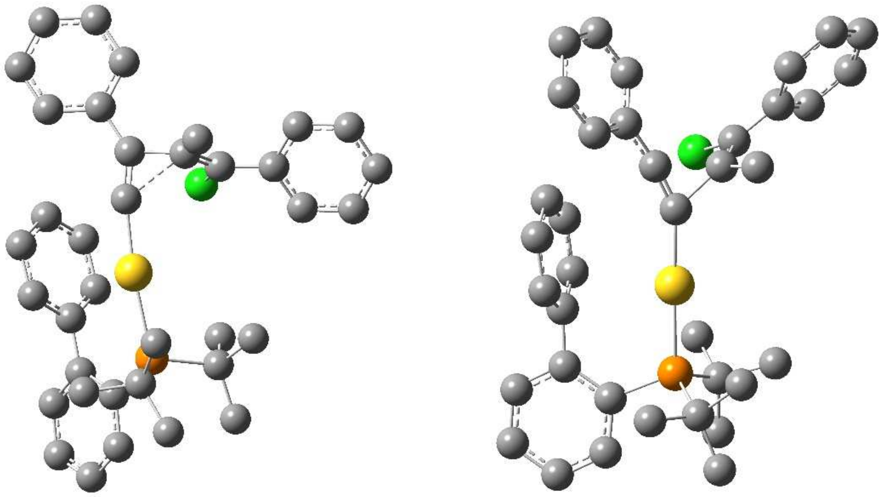

Figure S9. Molecular structure of **45a** (left) and **46a** (right) calculated by means of B3LYP-D3BJ/6-31G(d),def2-TZVP. All hydrogen atoms are omitted for clarity.

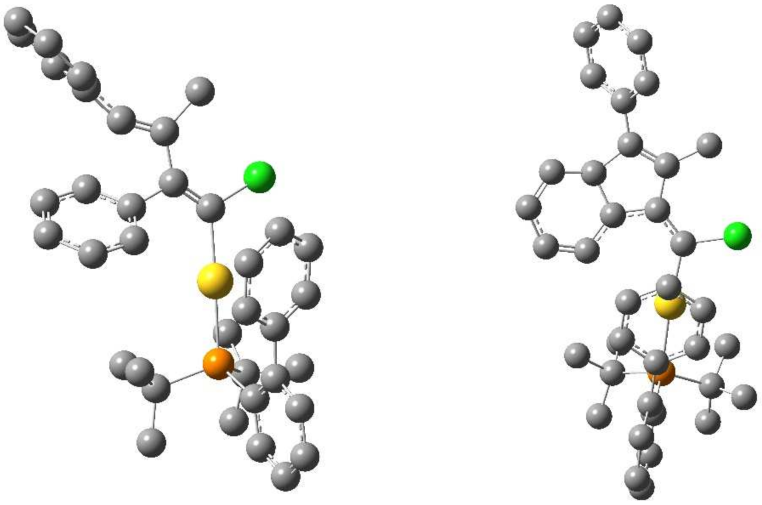

Figure S10. Molecular structure of **41a** (left) and **42a** (right) calculated by means of B3LYP-D3BJ/6-31G(d),def2-TZVP. All hydrogen atoms are omitted for clarity.

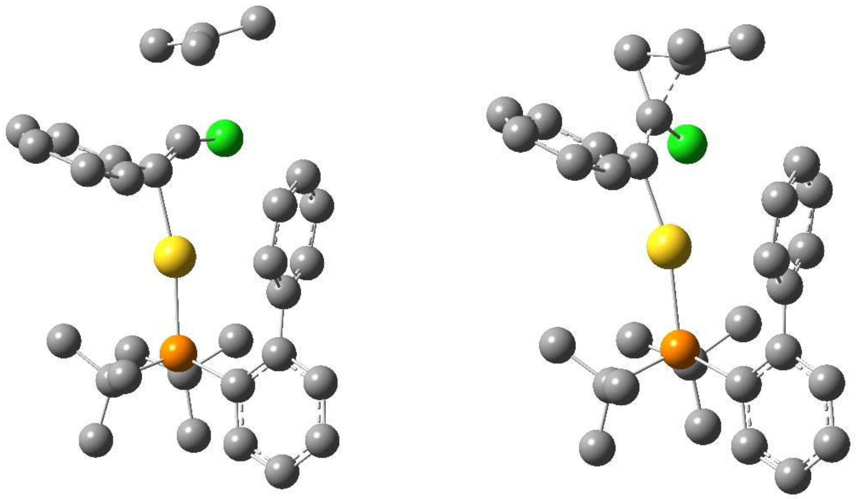

Figure S11. Molecular structure of **34a** (left) and **25a** (right) calculated by means of B3LYP-D3BJ/6-31G(d),def2-TZVP. All hydrogen atoms are omitted for clarity.

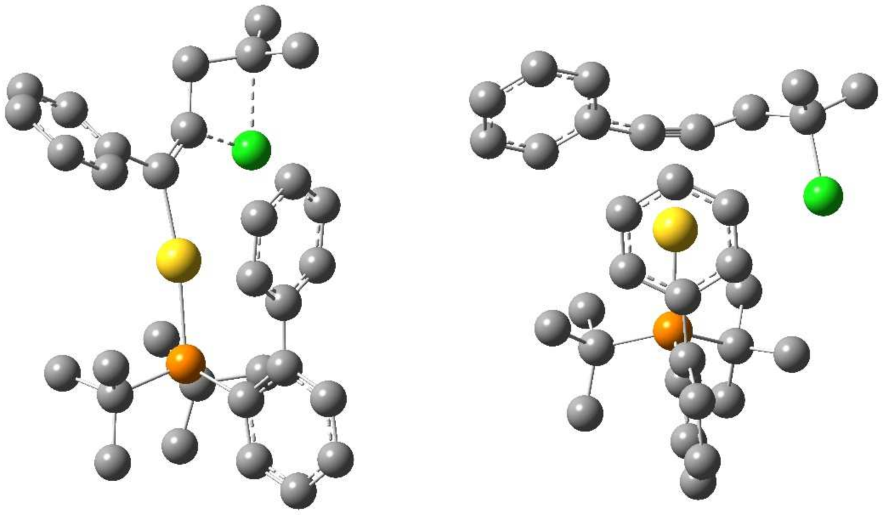

Figure S12. Molecular structure of **26a** (left) and **27a** (right) calculated by means of B3LYP-D3BJ/6-31G(d),def2-TZVP. All hydrogen atoms are omitted for clarity.

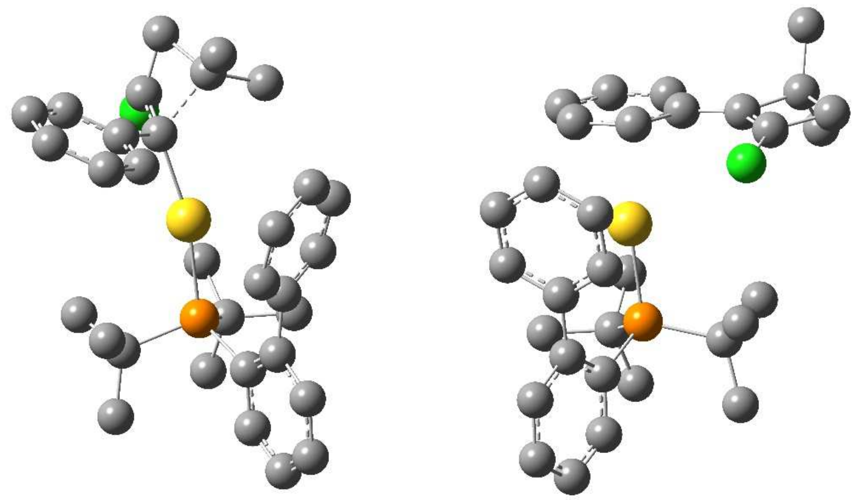

Figure S13. Molecular structure of **47a** (left) and **48a** (right) calculated by means of B3LYP-D3BJ/6-31G(d),def2-TZVP. All hydrogen atoms are omitted for clarity.

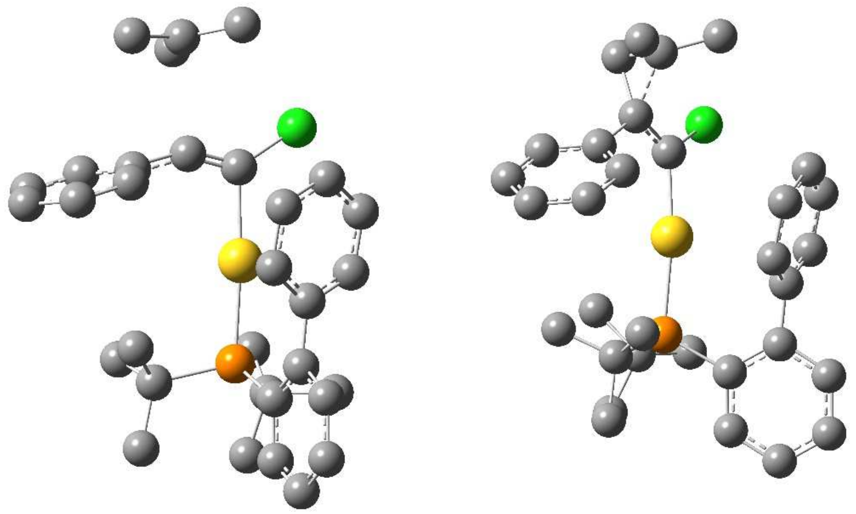

Figure S14. Molecular structure of **35a** (left) and **28a** (right) calculated by means of B3LYP-D3BJ/6-31G(d),def2-TZVP. All hydrogen atoms are omitted for clarity.

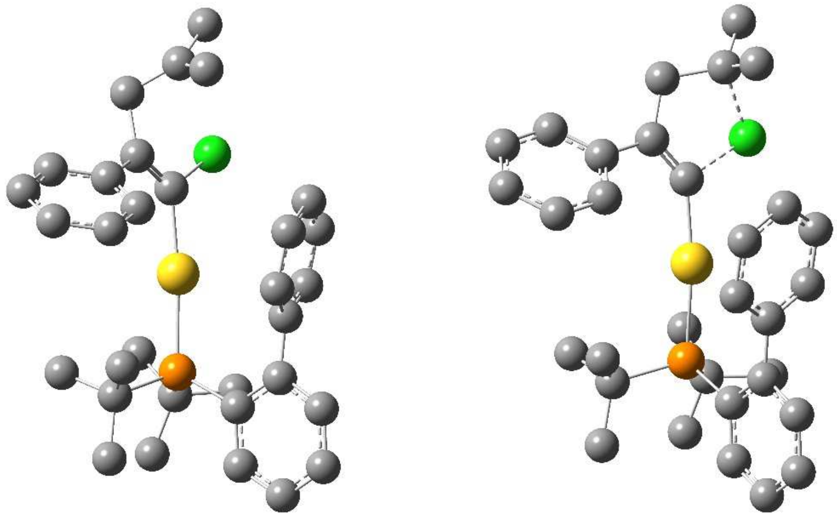

Figure S15. Molecular structure of **51a** (left) and **29a** (right) calculated by means of B3LYP-D3BJ/6-31G(d),def2-TZVP. All hydrogen atoms are omitted for clarity.

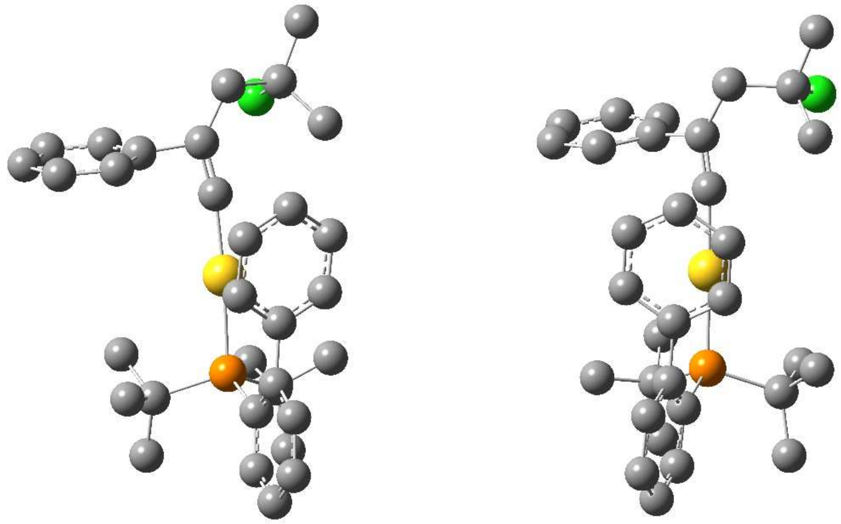

Figure S16. Molecular structure of **52a** (left) and **30a** (right) calculated by means of B3LYP-D3BJ/6-31G(d),def2-TZVP. All hydrogen atoms are omitted for clarity.

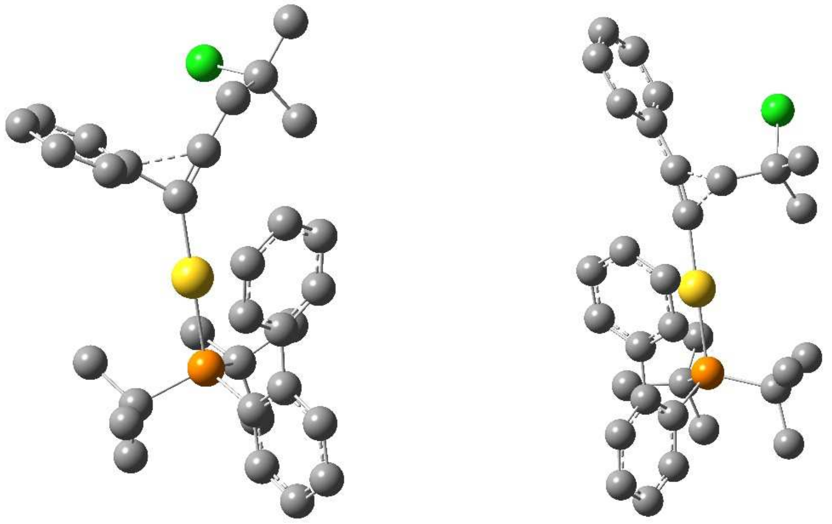

Figure S17. Molecular structure of **31a** (left) and **53a** (right) calculated by means of B3LYP-D3BJ/6-31G(d),def2-TZVP. All hydrogen atoms are omitted for clarity.

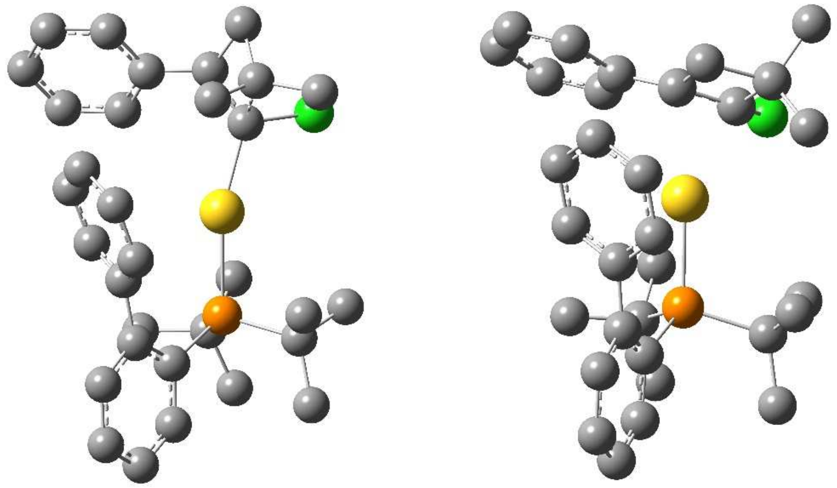

Figure S18. Molecular structure of **49a** (left) and **50a** (right) calculated by means of B3LYP-D3BJ/6-31G(d),def2-TZVP. All hydrogen atoms are omitted for clarity.

Table S1. Energy (Δ*E* in kcal/mol) and free-energy (Δ*G* in kcal/mol) of **18a-24a**, **32a-33a** and **36a-46a** relative to the starting materials (**7a** and **17a**) as calculated by means different methods.

	ΔE^a	ΔG^a	ΔE^b	ΔG^b
**32a**	-5.68	10.56	4.73	20.97
**18a**	-29.77	-10.63	-17.47	1.67
**36a**	-21.79	-4.02	-11.94	5.83
**19a**	-22.21	-5.47	-12.60	4.14
**20a**	-18.03	-1.92	-6.53	9.57
**21a**	-61.21	-42.57	-50.15	-31.52
**37a**	-26.24	-7.53	-15.76	2.96
**38a**	-34.99	-16.42	-21.58	-3.01
**39a**	-34.62	-15.36	-21.02	-1.75
**40a**	-59.15	-38.45	-46.51	-25.81
**33a**	-12.88	3.42	-2.35	13.95
**22a**	-31.68	-13.73	-19.53	-1.57
**43a**	-28.05	-9.26	-18.47	0.32
**23a**	-43.37	-23.81	-30.38	-10.82
**24a**	-31.55	-14.13	-19.17	-1.76
**44a**	-33.04	-14.82	-21.43	-3.21
**45a**	-36.89	-19.04	-24.77	-6.92
**46a**	-33.89	-16.06	-20.62	-2.79
**41a**	-22.18	-3.97	-9.59	8.62
**42a**	-48.05	-27.27	-36.33	-15.55

[a] B3LYP-D3BJ/6-31G(d),def2-TZVP. [b] B3LYP-D3BJ(DCE as solvent)//6-311++G(d,p),def2-TZVP//B3LYP-D3BJ/6-31G(d),def2-TZVP.

Table S2. Energy (Δ*E* in kcal/mol) and free-energy (Δ*G* in kcal/mol) of **25a-31a**, **34a-35a** and **47a-53a** relative to the starting materials (**12a** and **17a**) as calculated by means different methods.

	ΔE^a	ΔG^a	ΔE^b	ΔG^b
**34a**	-0.64	12.48	4.99	18.11
**25a**	-22.27	-4.51	-14.57	3.19
**26a**	-7.77	8.14	-6.96	8.96
**27a**	-47.99	-30.01	-43.81	-25.83
**47a**	-3.72	13.49	3.57	20.78
**48a**	-50.20	-31.79	-42.07	-23.66
**35a**	-7.41	5.63	-1.21	11.83

**28a**	-26.59	-9.10	-18.29	-0.81
**51a**	-16.31	-0.26	-15.50	0.56
**29a**	-26.42	-9.29	-21.41	-4.29
**52a**	-21.47	-5.43	-17.19	-1.14
**30a**	-21.48	-7.13	-16.85	-2.50
**31a**	-15.35	-1.32	-11.21	2.82
**53a**	-7.57	7.08	-4.78	9.87
**49a**	-6.12	11.71	0.85	18.68
**50a**	-49.35	-30.31	-42.46	-23.43

[a] B3LYP-D3BJ/6-31G(d),def2-TZVP. [b] B3LYP-D3BJ(DCE as solvent)//6-311++G(d,p),def2-TZVP//B3LYP-D3BJ/6-31G(d),def2-TZVP.

Table S3. Energy (ΔE in kcal/mol) and free-energy (ΔG in kcal/mol) of **18b**, **21b-24b**, **32b-33b** and **44b-46b** relative to the starting materials (**4a** and **17b**) as calculated by means different methods.

	ΔE^a	ΔG^a	ΔE^b	ΔG^b
32b	-1.61	12.19	8.28	22.09
18b	-30.00	-13.53	-20.75	-4.28
33b	-9.15	4.58	0.99	14.72
22b	-31.31	-15.75	-21.82	-6.26
23b	-48.81	-31.86	-35.45	-18.50
24b	-33.90	-19.76	-22.87	-8.73
21b	-64.14	-47.77	-54.10	-37.73
44b	-35.46	-20.28	-26.27	-11.09
45b	-39.30	-24.17	-28.53	-13.40
46b	-37.18	-22.44	-25.42	-10.68

[a] B3LYP-D3BJ/6-31G(d),def2-TZVP. [b] B3LYP-D3BJ(DCE as solvent)//6-311++G(d,p),def2-TZVP//B3LYP-D3BJ/6-31G(d),def2-TZVP.

Table S4. Energy (ΔE in kcal/mol) and free-energy (ΔG in kcal/mol) of **18c**, **21c-24c**, **32c-33c** and **44c-46c** relative to the starting materials (**3a** and **17a**) as calculated by means different methods.

	ΔE^a	ΔG^a	ΔE^b	ΔG^b
**32c**	-2.03	12.67	6.77	21.47
**18c**	-31.42	-14.13	-22.57	-5.29

**33c**	-8.83	5.66	-0.12	14.38
**22c**	-32.21	-16.17	-23.80	-7.76
**23c**	-45.74	-27.99	-36.13	-18.38
**24c**	-35.20	-20.80	-26.23	-11.82
**21c**	-64.52	-47.49	-57.89	-40.85
**44c**	-37.02	-20.93	-29.77	-13.68
**45c**	-41.42	-25.10	-32.97	-16.65
**46c**	-38.94	-23.98	-29.59	-14.62

[a] B3LYP-D3BJ/6-31G(d),def2-TZVP. [b] B3LYP-D3BJ(DCE as solvent)//6-311++G(d,p),def2-TZVP//B3LYP-D3BJ/6-31G(d),def2-TZVP.

Table S5. Energy (Δ*E* in kcal/mol) and free-energy (Δ*G* in kcal/mol) of **18d**, **21d-24d**, **32d-33d** and **44d-46d** relative to the starting materials (**7c** and **17a**) as calculated by means different methods.

	Δ*E*[a]	Δ*G*[a]	Δ*E*[b]	Δ*G*[b]
32d	-8.09	8.00	2.71	18.80
18d	-37.93	-19.01	-25.71	-6.79
33d	-15.08	0.82	-4.43	11.47
22d	-39.34	-20.94	-28.36	-9.95
23d	-45.46	-26.20	-31.33	-12.07
24d	-33.54	-16.43	-19.98	-2.86
21d	-63.42	-44.46	-51.68	-32.72
44d	-34.65	-16.31	-21.81	-3.47
45d	-39.60	-21.44	-26.15	-7.99
46d	-37.19	-19.85	-22.58	-5.23

[a] B3LYP-D3BJ/6-31G(d),def2-TZVP. [b] B3LYP-D3BJ(DCE as solvent)//6-311++G(d,p),def2-TZVP//B3LYP-D3BJ/6-31G(d),def2-TZVP.

Table S6. Energy (Δ*E* in kcal/mol) and free-energy (Δ*G* in kcal/mol) of **18e**, **21e-24e**, **32e-33e** and **44e-46e** relative to the starting materials (**7c** and **17b**) as calculated by means different methods.

	Δ*E*[a]	Δ*G*[a]	Δ*E*[b]	Δ*G*[b]
32e	-7.53	8.50	4.08	20.11
18e	-36.78	-18.34	-23.99	-5.54
33e	-15.15	0.50	-3.80	11.85

22e	-38.66	-20.63	-26.63	-8.60
23e	-48.33	-29.36	-32.04	-13.07
24e	-31.95	-15.04	-17.83	-0.92
21e	-62.31	-43.00	-49.02	-29.70
44e	-33.40	-15.06	-19.39	-1.05
45e	-37.99	-20.02	-23.18	-5.21
46e	-35.97	-18.45	-19.72	-2.20

[a] B3LYP-D3BJ/6-31G(d),def2-TZVP. [b] B3LYP-D3BJ(DCE as solvent)//6-311++G(d,p),def2-TZVP//B3LYP-D3BJ/6-31G(d),def2-TZVP.

Table S7. Energy (Δ*E* in kcal/mol) and free-energy (Δ*G* in kcal/mol) of **18f**, **21f-24f**, **32f-33f** and **44f-46f** relative to the starting materials (**3a** and **17c**) as calculated by means different methods.

	Δ*E*[a]	Δ*G*[a]	Δ*E*[b]	Δ*G*[b]
**32f**	-1.83	11.33	7.29	20.45
**18f**	-34.53	-17.74	-23.02	-6.23
**33f**	-9.54	3.61	0.92	14.07
**22f**	-35.12	-19.80	-24.40	-9.09
**23f**	-47.74	-30.10	-35.67	-18.03
**24f**	-38.50	-23.45	-26.95	-11.90
**21f**	-63.17	-48.63	-57.25	-42.72
**44f**	-35.81	-19.58	-28.58	-12.35
**45f**	-44.48	-28.73	-33.02	-17.28
**46f**	-42.23	-25.96	-26.61	-10.34

[a] B3LYP-D3BJ/6-31G(d),def2-TZVP. [b] B3LYP-D3BJ(DCE as solvent)//6-311++G(d,p),def2-TZVP//B3LYP-D3BJ/6-31G(d),def2-TZVP.

Table S8. Energy (Δ*E* in kcal/mol) and free-energy (Δ*G* in kcal/mol) of **18g**, **21g-24g**, **32g-33g** and **44g-46g** relative to the starting materials (**7c** and **17c**) as calculated by means different methods.

	Δ*E*[a]	Δ*G*[a]	Δ*E*[b]	Δ*G*[b]
32g	-7.08	7.20	3.89	18.17
18g	-42.75	-24.39	-27.19	-8.82
33g	-15.22	-0.83	-2.84	11.55
22g	-42.63	-25.76	-28.25	-11.37
23g	-48.40	-29.56	-31.19	-12.36

24g	-37.77	-20.10	-21.63	-3.96
21g	-62.70	-46.02	-51.09	-34.41
44g	-34.57	-15.81	-21.36	-2.60
45g	-43.15	-25.14	-26.23	-8.22
46g	-41.53	-23.23	-23.97	-5.66

[a] B3LYP-D3BJ/6-31G(d),def2-TZVP. [b] B3LYP-D3BJ(DCE as solvent)//6-311++G(d,p),def2-TZVP//B3LYP-D3BJ/6-31G(d),def2-TZVP.

2. ^{13}C-Labeling Experiments

General remarks: Chemicals were purchased from ABCR, Alfa Aesar, Acros Organics, Carbolution, TCI, or Sigma-Aldrich. All chemicals were reagent grade and used without further purification. Dry reaction solvents were purchased from Sigma-Aldrich. Reactions were monitored by TLC analysis with silica gel 60 F254 thin-layer plates. Flash chromatography was carried out on silica 60 (40−63 μm, 230−400 mesh). ^{1}H and ^{13}C NMR spectra were measured with Bruker Avance NEO 400 and Avance HD 600 spectrometers. All chemical shifts (δ) are given in ppm. The spectra were referenced to the peak for the protium impurity in the deuterated solvents indicated in brackets in the analytical data ($CDCl_3$, ^{1}H: 7.26 ppm, ^{13}C: 77.16 ppm; acetonitrile-d_3, ^{1}H: 1.94 ppm, ^{13}C: 1.32, 118.31 ppm). Signal multiplicities were determined as s (singlet), d (doublet), t (triplet), sext (sextet), m (multiplet), and dd (doublet of doublets). Coupling constants (J) are reported in Hertz (Hz). ^{13}C NMR spectra were measured with ^{1}H decoupling and the ^{13}C assignment was achieved via distortionless enhancement by polarization transfer 135, HSQC, HMBC, and correlation spectroscopy spectra.

(Chloroethynyl)benzene (^{13}C(1)-**3a**)[1], (bromoethynyl)benzene (^{13}C(1)-**4a**)[2] and 1-methoxy-4-(pent-1-yn-1-yl)benzene (**7b**)[3] were synthesized according to literature procedures. 2-Methyl-1-hexene (**12b**) was purchased from TCI. JohnPhos[Au(NCMe)]SbF_6 and Me_3PAuCl and were purchased from Sigma-Aldrich. $AgSbF_6$ was purchased from Carbolution.

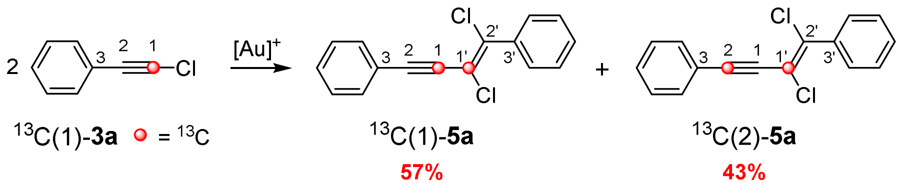

Synthesis of ^{13}C-labeled Enyne 5a. Chloroarylacetylene ^{13}C(1)-**3a** (54.6 mg, 400 μmol, 1.0 eq) was dissolved in dry DCM (0.4 mL). Then [JohnPhosAu(NCMe)]SbF_6 (5 mol%, 20 μmol, 15.4 mg) was added. The mixture was stirred at 40 °C for 21 hours. After removing the solvent in vacuo, the residue was adsorbed onto Celite® and purified by flash chromatography (SiO_2, *n*-hexane) to yield a mixture of ^{13}C(1)-**5a** and ^{13}C(2)-**5a** (42.1 mg, 154 μmol, 39%) as a yellow solid. R_f (*n*-hexane) = 0.31. ^{1}H NMR (400 MHz, CD_3CN): δ = 7.62–7.55 (m, 4H, CH_{ar}), 7.51–7.42 ppm (m, 6H, CH_{ar}). ^{13}C NMR (101 MHz, CD_3CN): δ = 136.5 (m, C3'$_{ar}$), 132.6 (m, C_{ar}H), 131.03 (C_{ar}H), 130.97 (m, C_{ar}H), 130.0 (d, J = 2.4 Hz, C_{ar}H), 129.8 (C_{ar}H), 129.8 (d, J = 5.7 Hz, C_{ar}H), 129.4 (C_{ar}H), 122.0 (dd, $^{2}J_{C3\text{-}C1}$ = 13.1 Hz, $^{3}J_{C3\text{-}C1'}$ = 2.6 Hz, C3$_{ar}$ in ^{13}C(1)-**5a**), 122.0 (dd,

$^{1}J_{C3\text{-}C2}$ = 90.0 Hz, $^{3}J_{C3\text{-}C1'}$ = 2.8 Hz, C3$_{ar}$ in ^{13}C(2)-**5a**), 110.8 (d, $^{1}J_{C1'\text{-}C1}$ = 114.8 Hz, *C*1'=C2', ^{13}C(1)-**5a**), 110.8 (d, $^{2}J_{C1'\text{-}C2}$ = 14.2 Hz, *C*1'=C2', ^{13}C(2)-**5a**), 98.2 (d, $^{2}J_{C2\text{-}C1'}$ = 14.3 Hz, *C*2≡C1), 85.2 ppm (d, $^{1}J_{C1\text{-}C1'}$ = 114.8 Hz, C2≡*C*1).

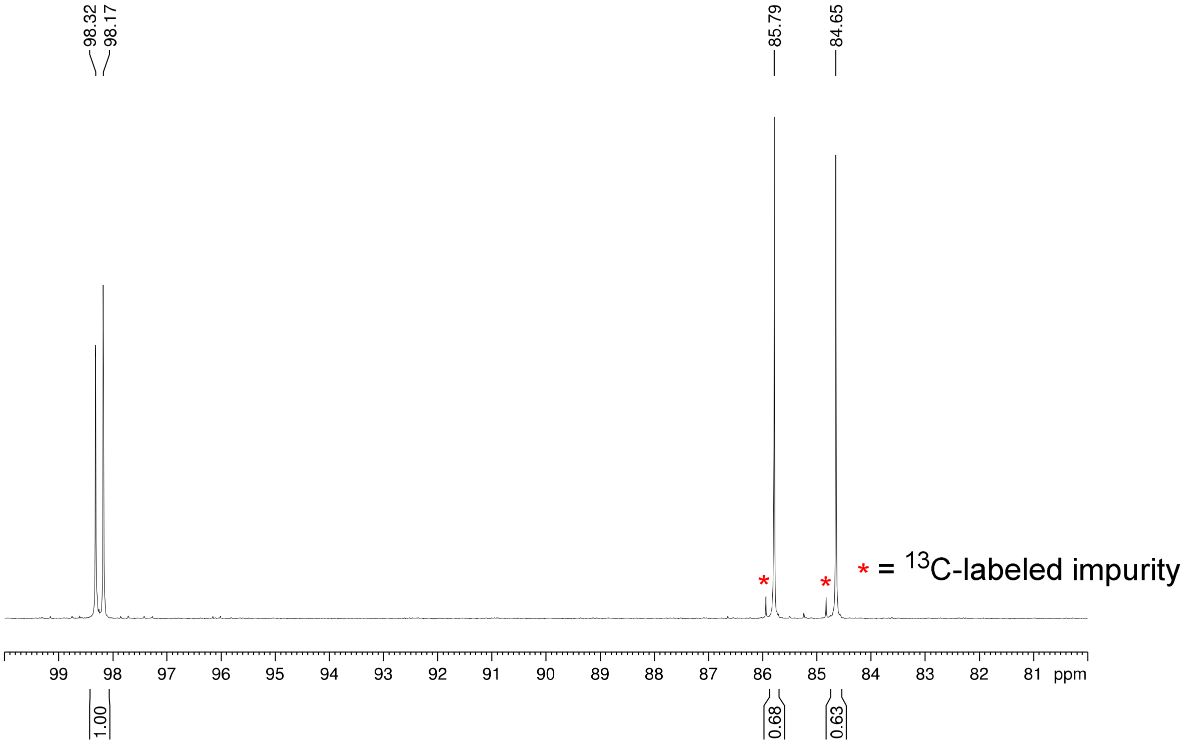

Figure S19. Extract from the quantitative ^{13}C NMR spectrum of ^{13}C(1)-**5a** and ^{13}C(2)-**5a** in CD_3CN at 101 MHz measured with inverse gated-decoupling. The signals show the ^{13}C-labeled acetylenic carbon atoms C2 and C1.

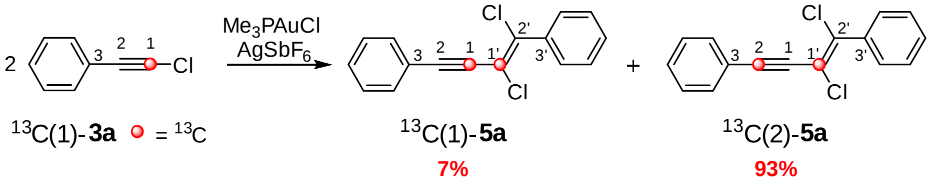

Synthesis of ^{13}C-labeled Enyne 5a via Me_3PAuCl. Chloroarylacetylene ^{13}C(1)-**3a** (19.0 mg, 139 µmol, 1.0 eq) and Me_3PAuCl (5 mol%, 7 µmol, 2.1 mg) were dissolved in dry DCM (0.3 mL). Then $AgSbF_6$ (10 mol%, 14 µmol, 4.8 mg) was added. The mixture was stirred at 40 °C for 18 hours. After removing the solvent in vacuo, the residue was dissolved in CD_3CN (0.6 mL) and directly analyzed via ^{13}C NMR spectroscopy (Figure S20).

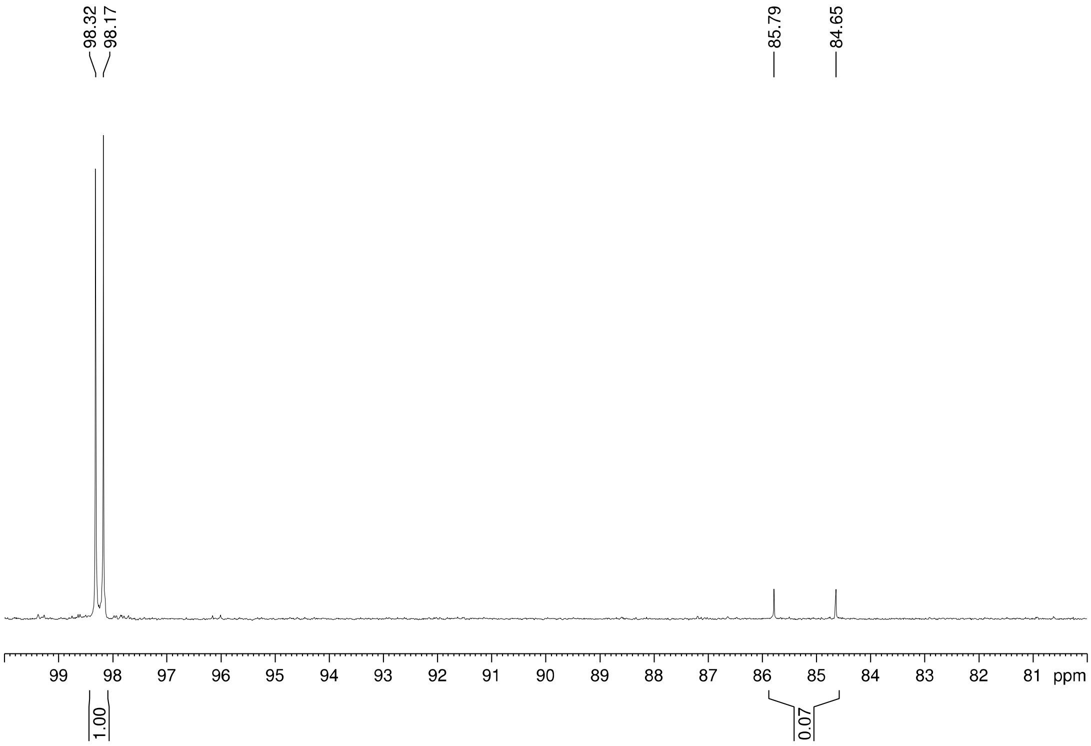

Figure S20. Extract from the ^{13}C NMR spectrum of the crude reaction mixture of ^{13}C(1)-**5a** and ^{13}C(2)-**5a** in CD_3CN at 101 MHz. The signals show the ^{13}C-labeled acetylenic carbon atoms C2 and C1.

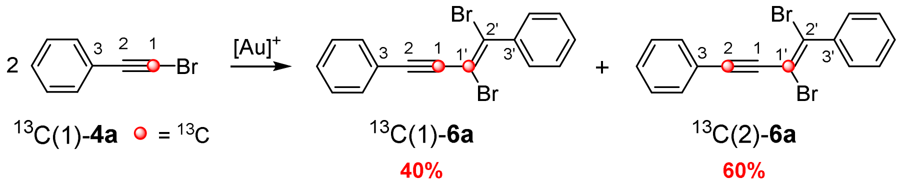

Synthesis of ^{13}C-labeled Enyne 6a. Bromoarylacetylene ^{13}C(1)-**4a** (72.4 mg, 400 µmol, 1.0 eq) was dissolved in dry DCM (0.4 mL). Then [JohnPhosAu(NCMe)]SbF_6 (5 mol%, 20 µmol, 15.4 mg) was added. The mixture was stirred at 40 °C for 18 hours. After removing the solvent in vacuo, the residue was adsorbed onto Celite® and purified by flash chromatography (SiO_2, *n*-hexane) to yield a mixture of ^{13}C(1)-**6a** and ^{13}C(2)-**6a** (19.4 mg, 54 µmol, 27%) as a yellow oil. R_f (*n*-hexane) = 0.25. ^{1}H NMR (600 MHz, CD_3CN): δ = 7.61–7.54 (m, 2H, CH_{ar}), 7.51–7.43 ppm (m, 8H, CH_{ar}). ^{13}C NMR (151 MHz, CD_3CN): δ = 140.0 (m, C_{ar}), 132.6 ($C_{ar}H$), 131.0 ($C_{ar}H$), 130.8 ($C_{ar}H$), 129.92 (d, J = 2.9 Hz, $C_{ar}H$), 129.85 ($C_{ar}H$), 129.85 (d, J = 5.4 Hz, $C_{ar}H$), 129.5 ($C_{ar}H$), 122.1 (m, $C3_{ar}$), 98.3 (d, $^1J_{C1'-C1}$ = 114.0 Hz, *C*1'=C2', ^{13}C(1)-**6a**), 98.3 (d, $^2J_{C1'-C2}$ = 13.5

Hz, $C1'$=C2', ^{13}C(2)-**6a**), 97.9 (d, $^{2}J_{C2\text{-}C1'}$ = 14.0 Hz, $C2$≡C1), 88.2 ppm (d, $^{1}J_{C1\text{-}C1'}$ = 114.3 Hz, C2≡$C1$).

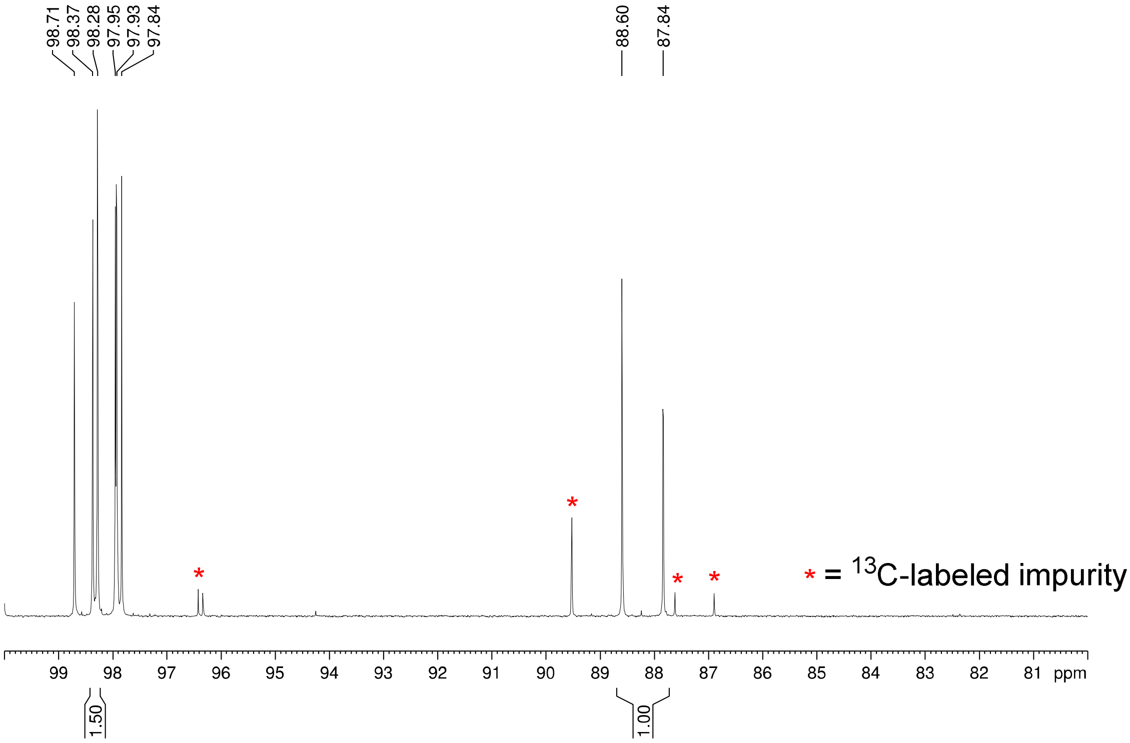

Figure S21. Extract from the quantitative ^{13}C NMR spectrum of ^{13}C(1)-**6a** and ^{13}C(2)-**6a** in CD_3CN at 151 MHz measured with inverse gated-decoupling. The signals show the ^{13}C-labeled vinyl carbon atom C1' and the acetylenic carbon atoms C2 and C1.

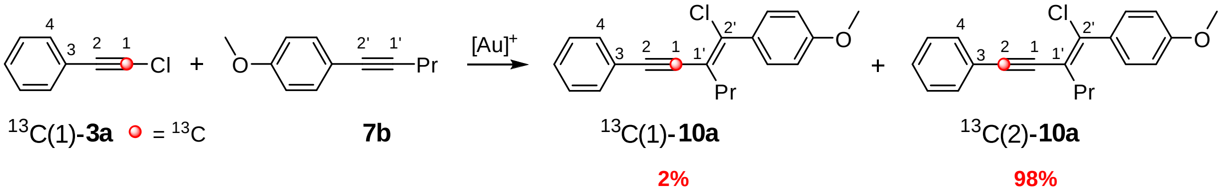

Synthesis of ^{13}C-labeled Enyne 10a.[1] Chloroarylacetylene ^{13}C(1)-**3a** (27.0 mg, 198 µmol, 1.0 eq), 1-methoxy-4-(pent-1-yn-1-yl)benzene (**7b**) (34.5 mg, 198 µmol, 1.0 eq) and Me_3PAuCl (5 mol%, 10 µmol, 3.1 mg) were dissolved in dry DCE (2 mL). Then $AgSbF_6$ (10 mol%, 20 µmol, 6.9 mg) was added. The mixture was stirred at room temperature for 3 days. After removing the solvent in vacuo, the residue was adsorbed onto Celite® and purified by flash chromatography (SiO_2, *n*-hexane/Et_2O 97:3) to yield a mixture of ^{13}C(1)-**10a** and ^{13}C(2)-**10a** (37.2 mg, 120 µmol, 61%) as a pale yellow oil. R_f (*n*-hexane/Et_2O 97:3) = 0.32. ^{1}H NMR (400 MHz, $CDCl_3$): δ = 7.56–7.49 (m, 2H, CH_{ar}), 7.38–7.28 (m, 5H, CH_{ar}), 6.94–6.88 (m, 2H, CH_{ar}), 3.84 (s, 3H, OCH_3),

2.30–2.23 (m, 2H, CH_2), 1.72–1.62 (m, 2H, CH_2), 0.90 ppm (t, $^3J_{H,H}$ = 7.4 Hz, CH_3). ^{13}C NMR (101 MHz, $CDCl_3$): δ = 159.9 ($C_{ar}OCH_3$), 135.7 (d, $^3J_{C2'\text{-}C2}$ = 4.4 Hz, C=*C*Cl), 131.8 (d, J = 1.4 Hz, $C_{ar}H$), 130.5 ($C_{ar}H$), 130.4 (C_{ar}), 128.5 ($C_{ar}H$), 128.4 ($C_{ar}H$), 123.5 (d, $^1J_{C3\text{-}C2}$ = 91.0 Hz, $C3_{ar}$), 122.5 (d, $^2J_{C1'\text{-}C2}$ = 11.1 Hz, *C*=CCl), 113.8 ($C_{ar}H$), 96.3 (*C*2≡C1), 96.3 (d, $^1J_{C2\text{-}C3}$ = 90.8 Hz, *C*2≡C1), 88.4 (C2≡*C*1), 55.5 (OCH_3), 35.8 (CH_2), 22.3 (CH_2), 13.8 ppm (CH_3).

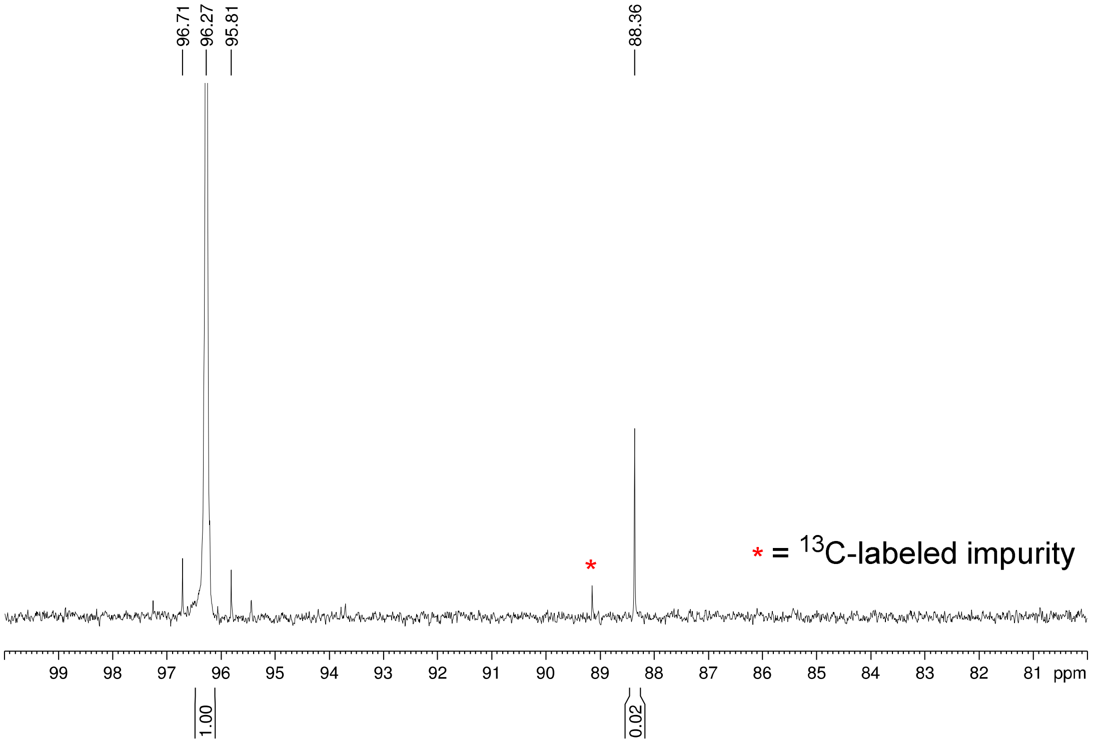

Figure S22. Extract from the ^{13}C NMR spectrum of ^{13}C(1)-**10a** and ^{13}C(2)-**10a** in $CDCl_3$ at 101 MHz. The signals show the ^{13}C-labeled acetylenic carbon atoms C2 and C1.

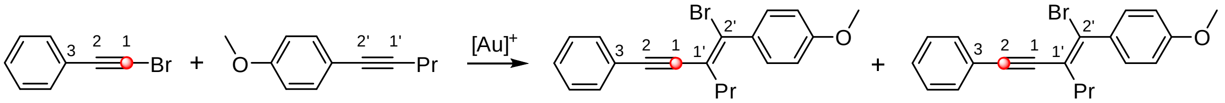

^{13}C(1)-**4a** ● = ^{13}C   **7b** ^{13}C(1)-**11a** 13% ^{13}C(2)-**11a** 87%

Synthesis of ^{13}C-labeled Enyne 11a. Bromoarylacetylene ^{13}C(1)-**4a** (72.4 mg, 400 µmol, 1.0 eq) and 1-methoxy-4-(pent-1-yn-1-yl)benzene (**7b**) (69.7 mg, 400 µmol, 1.0 eq) were dissolved in dry DCE (4 mL). Then [JohnPhosAu(NCMe)]SbF_6 (5 mol%, 20 µmol, 15.4 mg) was added. The mixture was stirred at room temperature for 4 days. After removing the solvent in vacuo, the residue was adsorbed onto Celite® and purified by flash chromatography (SiO_2, *n*-hexane/Et_2O 97:3) to yield a mixture of ^{13}C(1)-**11a** and ^{13}C(2)-**11a** (71.7 mg, 202 µmol, 51%) as a white solid.

R_f (*n*-hexane/Et_2O 97:3) = 0.35. ^1H NMR (600 MHz, $CDCl_3$): δ = 7.56–7.51 (m, 2H, CH_{ar}), 7.36–7.32 (m, 3H, CH_{ar}), 7.30–7.27 (m, 2H, CH_{ar}), 6.91–6.87 (m, 2H, CH_{ar}), 3.84 (s, 3H, OCH_3), 2.25–2.20 (m, 2H, CH_2), 1.70–1.63 (m, 2H, CH_2), 0.88 ppm (t, $^3J_{H,H}$ = 7.4 Hz, 3H, CH_3). ^{13}C NMR (151 MHz, $CDCl_3$): δ = 159.8 ($C_{ar}OCH_3$), 132.1 (C_{ar}), 131.8 ($C_{ar}H$), 130.6 ($C_{ar}H$), 128.6 ($C_{ar}H$), 128.5 ($C_{ar}H$), 128.5 (d, J = 5.5 Hz, $C_{ar}H$), 127.1 (d, J = 4.5 Hz, C=*C*Br), 126.2 (d, J = 11.1 Hz, *C*=CBr), 123.4 (d, $^2J_{C3\text{-}C2}$ = 91.2 Hz, $C3_{ar}$), 113.8 ($C_{ar}H$), 95.4 (*C*2≡C1), 95.4 (d, $^2J_{C2\text{-}C3}$ = 90.8 Hz, *C*2≡C1), 90.3 (C2≡*C*1), 55.5 (OCH_3), 36.3 (CH_2), 22.2 (CH_2), 13.7 ppm (CH_3).

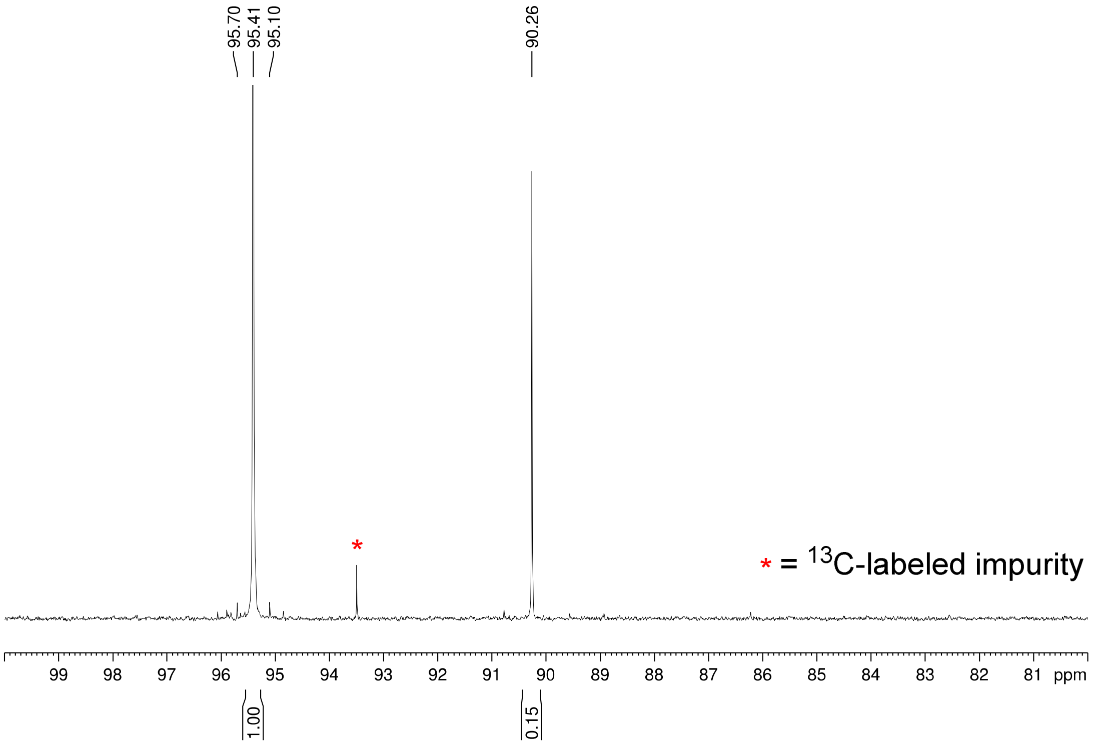

Figure S23. Extract from the quantitative ^{13}C NMR spectrum of ^{13}C(1)-**11a** and ^{13}C(2)-**11a** in $CDCl_3$ at 151 MHz measured with inverse gated-decoupling. The signals show the ^{13}C-labeled acetylenic carbon atoms C2 and C1.

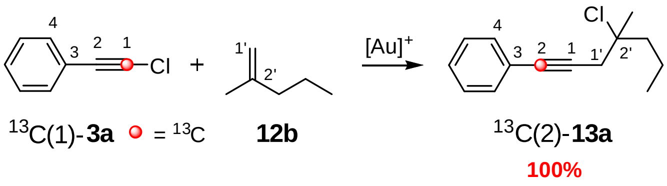

Synthesis of ^{13}C-labeled Alkyne 13a. Chloroarylacetylene ^{13}C(1)-**3a** (54.6 mg, 400 µmol, 1.0 eq) and 2-methyl-1-hexene (**12b**) (392.8 mg, 4 mmol, 10.0 eq) were dissolved in dry DCM (4 mL). Then [JohnPhosAu(NCMe)]SbF_6 (5 mol%, 20 µmol, 15.4 mg) was added. The mixture was

stirred at room temperature for 22 hours. After removing the solvent in vacuo, the residue was adsorbed onto Celite® and purified by flash chromatography (SiO_2, *n*-hexane) to yield ^{13}C(2)-**13a** (60.4 mg, 257 µmol, 64%) as a colorless oil. R_f (*n*-hexane) = 0.31. ^{1}H NMR (600 MHz, $CDCl_3$): δ = 7.45–7.39 (m, 2H, CH_{ar}), 7.33–7.27 (m, 3H, CH_{ar}), 2.96–2.87 (m, 2H, CH_2), 1.97–1.91 (m, 1H, CH_2), 1.91–1.84 (m, 1H, CH_2), 1.69 (s, 3H, CH_3), 1.54–1.46 (m, 2H, CH_2), 1.38 (sext, $^3J_{H,H}$ = 7.2 Hz, 2H, CH_2), 0.95 ppm (t, $^3J_{H,H}$ = 7.4 Hz, 3H, CH_3). ^{13}C NMR (151 MHz, $CDCl_3$): δ = 131.8 (C_{ar}H), 128.4 (d, $^2J_{C4\text{-}C2}$ = 5.5 Hz, C4$_{ar}$H), 128.1 (C_{ar}H), 123.5 (d, $^1J_{C3\text{-}C2}$ = 90.7 Hz, C3$_{ar}$), 83.2 (*C*2≡C1), 83.2 (d, $^2J_{C2\text{-}C3}$ = 90.5 Hz, *C*2≡C1), 71.8 (d, $^3J_{C2'\text{-}C2}$ = 2.2 Hz, CCl), 43.0 (CH_2), 35.6 (d, $^2J_{C1'\text{-}C2}$ = 10.0 Hz, CH_2), 30.0 (CH_3), 27.0 (CH_2), 22.9 (CH_2), 14.2 ppm (CH_3).

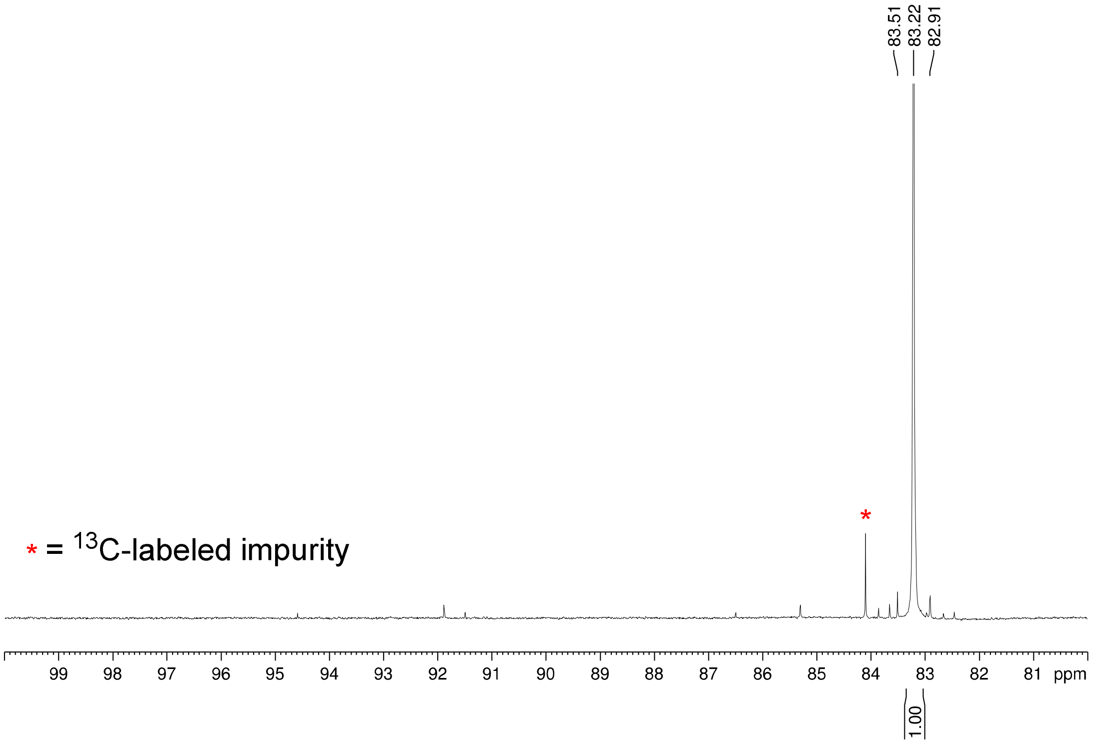

Figure S24. Extract from the quantitative ^{13}C NMR spectrum of ^{13}C(2)-**13a** in $CDCl_3$ measured at 151 MHz with inverse gated-decoupling. The signal shows the ^{13}C-labeled acetylenic carbon atom C2.

3. Computational Details

General remarks. All calculations were performed by using the program package Gaussian 16[4]. The geometrical parameters of all stationary points were optimized by means of the density functional B3LYP[5-7] together with the dispersion correction with Becke-Johnson damping[8] (D3BJ). For all structures C1 symmetry was applied. Frequency calculations were carried out at each of the stationary points to verify the nature of the stationary point. It turned out that all stationary states have no imaginary frequency.

Calculation of **3-4**, **7**, **12** and **17-53**: The basis set 6-31G(d) was applied for the elements C, H, O, P, Cl and Br; Au was calculated with the def2-TZVP basis set. Furthermore, single-point calculations on the B3LYP-D3BJ-optimized structures were performed using B3LYP-D3BJ. As basis sets 6-311++G(d,p) (for C, H, O, P, Cl and Br) and def2-TZVP for Au were employed. To determine the solvent effect, single-point calculations were conducted using dichloroethane as solvent.

Calculation of **54** and **55**: The basis set 6-311++G(d,p) was applied for the elements C, H and P; Au was calculated with the aug-cc-pVTZ-PP basis set. Furthermore, single-point calculations on the B3LYP-D3BJ-optimized structures were performed using the double-hybrid density functional approximation B2PLYP-D3[9] and CCSD(T)[10]. As basis sets 6-311++G(d,p) (for C, H, O, P, Cl and Br) and aug-cc-pVTZ-PP for Au were employed. To determine the solvent effect, the single-point calculations with B2PLYP-D3 were conducted using dichloroethane as solvent.

Calculation of **56-58**: The basis set 6-31G(d) was applied for the elements C, H and P; Au was calculated with the def2-TZVP basis set. Furthermore, single-point calculations on the B3LYP-D3BJ-optimized structures were performed using the double-hybrid density functional approximation B2PLYP-D3[9]. As basis sets 6-311++G(d,p) (for C, H, O, P, Cl and Br) and aug-cc-pVTZ-PP for Au were employed. To determine the solvent effect, the single-point calculations were conducted using dichloroethane as solvent.

4. Cartesian Coordinates and Absolute Energies for All Calculated Compounds

Table S9 Absolute energies [au] calculated by means of different methods.

	E[a]	*G*[a]	*E*[b]
JohnPhosAu$^+$	-1255.475093	-1255.105478	-1255.794733
Me_3PAu^+	-596.707922	-596.626241	-596.869363
**3a**	-767.997889	-767.929750	-768.125992
**4a**	-2879.212398	-2879.144731	-2882.050639
**17a**	-2023.536105	-2023.077866	-2023.964398
**17b**	-4134.752568	-4134.295808	-4137.888855
**17c**	-1364.777586	-1364.610219	-1365.030847
**7a**	-347.740961	-347.638084	-347.853638
**7c**	-462.269101	-462.136372	-462.418431
**12a**	-157.237382	-157.156029	-157.292507

[a] B3LYP-D3BJ/6-31G(d),def2-TZVP.
[b] B3LYP-D3BJ(DCE as solvent)//6-311++G(d,p),def2-TZVP//B3LYP-D3BJ/6-31G(d),def2-TZVP.

Table S10 Absolute energies [au] calculated by means of different methods.

	E[a]	*G*[a]	*E*[b]
**32a**	-2371.286116	-2370.699121	-2371.810504
**18a**	-2371.324505	-2370.732896	-2371.845870
**36a**	-2371.311786	-2370.722349	-2371.837061
**19a**	-2371.312459	-2370.724661	-2371.838116
**20a**	-2371.305795	-2370.719016	-2371.828450
**21a**	-2371.374603	-2370.783796	-2371.897953
**37a**	-2371.318886	-2370.727945	-2371.843144
**38a**	-2371.332823	-2370.742113	-2371.852423
**39a**	-2371.332241	-2370.740424	-2371.851533
**40a**	-2371.371326	-2370.777221	-2371.892152
**33a**	-2371.297586	-2370.710495	-2371.821778
**22a**	-2371.327559	-2370.737823	-2371.849159
**43a**	-2371.321768	-2370.730706	-2371.847467
**23a**	-2371.346187	-2370.753897	-2371.866453
**24a**	-2371.327342	-2370.738474	-2371.848590
**44a**	-2371.329711	-2370.739571	-2371.852180
**45a**	-2371.335861	-2370.746298	-2371.857511
**46a**	-2371.331078	-2370.741549	-2371.850901
**41a**	-2371.312412	-2370.722272	-2371.833320
**42a**	-2371.353639	-2370.759410	-2371.875930

[a] B3LYP-D3BJ/6-31G(d),def2-TZVP.
[b] B3LYP-D3BJ(DCE as solvent)//6-311++G(d,p),def2-TZVP//B3LYP-D3BJ/6-31G(d),def2-TZVP.

Table S11 Absolute energies [au] calculated by means of different methods.

	E[a]	*G*[a]	*E*[b]
**34a**	-2180.774499	-2180.214001	-2181.248954
**25a**	-2180.808972	-2180.241075	-2181.280125
**26a**	-2180.785876	-2180.220928	-2181.267990
**27a**	-2180.849959	-2180.281713	-2181.326729
**47a**	-2180.779414	-2180.212395	-2181.251215
**48a**	-2180.853482	-2180.284551	-2181.323943
**35a**	-2180.785299	-2180.224924	-2181.258830
**28a**	-2180.815856	-2180.248394	-2181.286060
**51a**	-2180.799484	-2180.234303	-2181.281599
**29a**	-2180.815584	-2180.248706	-2181.291025
**52a**	-2180.807709	-2180.242546	-2181.284299
**30a**	-2180.807719	-2180.245256	-2181.283753

**31a**	-2180.797943	-2180.235998	-2181.274768
**53a**	-2180.785546	-2180.222612	-2181.264525
**49a**	-2180.783232	-2180.215228	-2181.255549
**50a**	-2180.852125	-2180.282204	-2181.324575

[a] B3LYP-D3BJ/6-31G(d),def2-TZVP.
[b] B3LYP-D3BJ(DCE as solvent)//6-311++G(d,p),def2-TZVP//B3LYP-D3BJ/6-31G(d),def2-TZVP.

Table S12 Absolute energies [au] calculated by means of different methods.

	E[a]	*G*[a]	*E*[b]
**32b**	-7013.967539	-7013.421109	-7019.926300
**18b**	-7014.012769	-7013.462095	-7019.972558
**33b**	-7013.979543	-7013.433235	-7019.937922
**22b**	-7014.014859	-7013.465631	-7019.974272
**23b**	-7014.042744	-7013.491318	-7019.995981
**24b**	-7014.018986	-7013.472032	-7019.975932
**21b**	-7014.067184	-7013.516665	-7020.025708
**44b**	-7014.021472	-7013.472862	-7019.981355
**45b**	-7014.027590	-7013.479053	-7019.984954
**46b**	-7014.024211	-7013.476298	-7019.980002

[a] B3LYP-D3BJ/6-31G(d),def2-TZVP.
[b] B3LYP-D3BJ(DCE as solvent)//6-311++G(d,p),def2-TZVP//B3LYP-D3BJ/6-31G(d),def2-TZVP.

Table S13 Absolute energies [au] calculated by means of different methods.

	E[a]	*G*[a]	*E*[b]
**32c**	-2791.537229	-2790.987420	-2792.079602
**18c**	-2791.584060	-2791.030136	-2792.126362
**33c**	-2791.548067	-2790.998590	-2792.090578
**22c**	-2791.585330	-2791.033389	-2792.128323
**23c**	-2791.606893	-2791.052219	-2792.147975
**24c**	-2791.590089	-2791.040756	-2792.132184
**21c**	-2791.636819	-2791.083292	-2792.182644
**44c**	-2791.592991	-2791.040972	-2792.137825
**45c**	-2791.600005	-2791.047612	-2792.142934
**46c**	-2791.596047	-2791.045826	-2792.137538

[a] B3LYP-D3BJ/6-31G(d),def2-TZVP.
[b] B3LYP-D3BJ(DCE as solvent)//6-311++G(d,p),def2-TZVP//B3LYP-D3BJ/6-31G(d),def2-TZVP.

Table S14 Absolute energies [au] calculated by means of different methods.

	E[a]	*G*[a]	*E*[b]
**32d**	-2485.818095	-2485.201482	-2486.378508
**18d**	-2485.865658	-2485.244528	-2486.423808
**33d**	-2485.829240	-2485.212927	-2486.389889
**22d**	-2485.867906	-2485.247607	-2486.428016
**23c**	-2485.877645	-2485.255996	-2486.432751
**24c**	-2485.858663	-2485.240414	-2486.414664
**21c**	-2485.906267	-2485.285084	-2486.465193
**44c**	-2485.860421	-2485.240224	-2486.417593
**45c**	-2485.868305	-2485.248402	-2486.424503
**46c**	-2485.864474	-2485.245869	-2486.418805

[a] B3LYP-D3BJ/6-31G(d),def2-TZVP.
[b] B3LYP-D3BJ(DCE as solvent)//6-311++G(d,p),def2-TZVP//B3LYP-D3BJ/6-31G(d),def2-TZVP.

Table S15 Absolute energies [au] calculated by means of different methods.

	E^a	G^a	E^b
**32e**	-4597.033674	-4596.418641	-4600.300786
**18e**	-4597.080278	-4596.461401	-4600.345509
**33e**	-4597.045808	-4596.431388	-4600.313340
**22e**	-4597.083279	-4596.465061	-4600.349728
**23e**	-4597.098681	-4596.478967	-4600.358342
**24e**	-4597.072588	-4596.456151	-4600.335703
**21e**	-4597.120973	-4596.500700	-4600.385401
**44e**	-4597.074888	-4596.456176	-4600.338184
**45e**	-4597.082215	-4596.464081	-4600.344226
**46e**	-4597.078995	-4596.461580	-4600.338712

[a] B3LYP-D3BJ/6-31G(d),def2-TZVP.
[b] B3LYP-D3BJ(DCE as solvent)//6-311++G(d,p),def2-TZVP//B3LYP-D3BJ/6-31G(d),def2-TZVP.

Table S16 Absolute energies [au] calculated by means of different methods.

	E^a	G^a	E^b
**32f**	-2132.778398	-2132.521906	-2133.145228
**18f**	-2132.830497	-2132.568233	-2133.193518
**33f**	-2132.790682	-2132.534223	-2133.155372
**22f**	-2132.831442	-2132.571529	-2133.195730
**23f**	-2132.851561	-2132.587934	-2133.213691
**24f**	-2132.836823	-2132.577338	-2133.199784
**21f**	-2132.876139	-2132.617471	-2133.248081
**44f**	-2132.832542	-2132.571169	-2133.202386
**45f**	-2132.846352	-2132.585759	-2133.209458
**46f**	-2132.842781	-2132.581347	-2133.199245

[a] B3LYP-D3BJ/6-31G(d),def2-TZVP.
[b] B3LYP-D3BJ(DCE as solvent)//6-311++G(d,p),def2-TZVP//B3LYP-D3BJ/6-31G(d),def2-TZVP.

Table S17 Absolute energies [au] calculated by means of different methods.

	E^a	G^a	E^b
**32g**	-1827.057963	-1826.735115	-1827.443072
**18g**	-1827.114820	-1826.785455	-1827.492604
**33g**	-1827.070948	-1826.747918	-1827.453811
**22g**	-1827.114629	-1826.787645	-1827.494290
**23g**	-1827.123824	-1826.793706	-1827.498990
**24g**	-1827.106882	-1826.778627	-1827.483755
**21g**	-1827.146605	-1826.819930	-1827.530690
**44g**	-1827.101777	-1826.771790	-1827.483316
**45g**	-1827.115455	-1826.786657	-1827.491079
**46g**	-1827.112875	-1826.783609	-1827.487473

[a] B3LYP-D3BJ/6-31G(d),def2-TZVP.
[b] B3LYP-D3BJ(DCE as solvent)//6-311++G(d,p),def2-TZVP//B3LYP-D3BJ/6-31G(d),def2-TZVP.

Table S18 Absolute energies [au] calculated by means of different methods.

	E^a	G^a	E^b	E^c
**54a**	-830.265001	-830.079902	-829.585938	-828.397558
**55a**	-830.272002	-830.088069	-829.592618	-828.407508
**54b**	-1022.072139	-1021.840537	-1021.195280	
**55b**	-1022.082487	-1021.850727	-1021.204015	
**54c**	-1022.076519	-1021.845351	-1021.196565	
**55c**	-1022.078634	-1021.846409	-1021.200807	

[a] B3LYP-D3BJ/6-311++G(d,p),aug-cc-pVTZ-PP.

[b] B2PLYP-D3(DCE as solvent)/6-311++G(d,p),aug-cc-pVTZ-PP//B3LYP-D3BJ/6-311++G(d,p),aug-cc-pVTZ-PP.
[c] CCSD(T)/6-311++G(d,p),aug-cc-pVTZ-PP//B3LYP-D3BJ/6-311++G(d,p),aug-cc-pVTZ-PP.

Table S19 Absolute energies [au] calculated by means of different methods.

	E^a	G^a	E^b
**56a**	-828.931239	-828.764510	-828.386974
**57a**	-828.904861	-828.736586	-828.365117
**58a**	-828.913624	-828.745279	-828.371181
**56b**	-868.259299	-868.061943	-867.676736
**57b**	-868.245426	-868.048883	-867.664869
**58b**	-868.261953	-868.064781	-867.687042
**56c**	-907.571202	-907.346798	-906.950616
**57c**	-907.568588	-907.344068	-906.946585
**58c**	-907.594048	-907.369185	-906.980115
**56d**	-946.880879	-946.629036	-946.221747
**57d**	-946.892713	-946.639963	-946.232991
**58d**	-946.921697	-946.668993	-946.267638

[a] B3LYP-D3BJ/6-31G*,def2-TZVP.
[b] B2PLYP-D3(DCE as solvent)/6-311++G(d,p),aug-cc-pVTZ-PP//B3LYP-D3BJ/6-31G*,def2-TZVP.

Cartesian coordinates of the optimized geometry for Me_3PAu^+ at B3LYP-D3BJ/6-31G(d),def2-TZVP level of theory (number of imaginary frequencies = 0):

Au	-0.94531500	-0.00000700	0.00000300
P	1.32938100	0.00001700	-0.00004700
C	1.98190600	-1.44017700	-0.91009300
C	1.98187900	-0.06809400	1.70225400
C	1.98181000	1.50831200	-0.79213300
H	1.63974900	-2.36666600	-0.44153400
H	3.07720900	-1.40682100	-0.89002000
H	1.63838500	-1.41580500	-1.94758100
H	1.63987800	1.56573200	-1.82885000
H	3.07710200	1.47443300	-0.77300400
H	1.63799800	2.39453300	-0.25233000
H	1.63879400	-0.97910300	2.19958500
H	1.63927600	0.80057400	2.27061300
H	3.07718000	-0.06683600	1.66338800

Cartesian coordinates of the optimized geometry for $JohnPhosAu^+$ at B3LYP-D3BJ/6-31G(d),def2-TZVP level of theory (number of imaginary frequencies = 0):

Au	0.96134700	-1.21126000	-0.24412600
P	-1.11203000	-0.26326400	0.12328000
C	-1.63324400	-0.45033700	1.92877200
C	-2.31284100	-0.98896600	-1.15996500
C	-0.44074400	0.03197200	2.77635400
H	0.45764700	-0.56897500	2.60268000
H	-0.70641400	-0.05525100	3.83557400
H	-0.20231600	1.08060000	2.57660300
C	-2.86255200	0.40816900	2.27852100
H	-3.09485500	0.25358600	3.33816100
H	-3.75013000	0.13341300	1.70602800
H	-2.66679500	1.47362300	2.13613100
C	-1.90919500	-1.93467200	2.21919800
H	-1.06153700	-2.56941700	1.93951700
H	-2.80276800	-2.29869200	1.70532800
H	-2.07718600	-2.05673400	3.29484900
C	-2.02996200	-2.50187200	-1.27406700
H	-1.01246400	-2.69676600	-1.62719600

H	-2.72687800	-2.93105200	-2.00232100
H	-2.16956300	-3.02936500	-0.32795100
C	-3.80195400	-0.80065200	-0.81602000
H	-4.08304900	-1.32554500	0.09997600
H	-4.39401000	-1.23023000	-1.63175400
H	-4.09791100	0.24594400	-0.72422800
C	-1.98643300	-0.31941400	-2.50825700
H	-0.92446300	-0.41643800	-2.76141500
H	-2.24668200	0.74109800	-2.52182500
H	-2.56021100	-0.82180500	-3.29461600
C	-0.91181500	1.54774800	-0.18415300
C	-2.09237300	2.30168300	-0.31889700
C	0.33527000	2.22844000	-0.24516400
C	-2.06510200	3.68020600	-0.51072500
H	-3.05484200	1.81074000	-0.26612000
C	0.33934800	3.61821100	-0.42881700
C	-0.84151000	4.34374100	-0.56509500
H	-2.99646300	4.22781500	-0.61263400
H	1.29614700	4.12911300	-0.47103300
H	-0.80356200	5.41837900	-0.71213300
C	1.66298500	1.56040700	-0.09341800
C	2.22909300	0.82179500	-1.15738000
C	2.34703200	1.61372300	1.12478200
C	3.39761100	0.06200500	-0.95324500
H	1.77017200	0.86372700	-2.14117500
C	3.52415100	0.88083600	1.32171300
H	1.93720000	2.20591900	1.93678400
C	4.03508400	0.08177000	0.30039600
H	3.83627200	-0.48457100	-1.78278100
H	4.03232500	0.92825200	2.27954100
H	4.93931000	-0.49764800	0.45557500

Cartesian coordinates of the optimized geometry for **3a** at B3LYP-D3BJ/6-31G(d),def2-TZVP level of theory (number of imaginary frequencies = 0):

C	-2.63469900	-1.20884700	0.00001300
C	-1.24265400	-1.21361900	-0.00001500
C	-0.53026600	0.00019000	-0.00001200
C	-1.24288600	1.21374400	0.00002300
C	-2.63500400	1.20862000	0.00005200
C	-3.33485700	-0.00016100	0.00004800
H	-3.17508700	-2.15106200	0.00000900
H	-0.69347400	2.14954900	0.00002500
H	-3.17556300	2.15073700	0.00007700
H	-0.69288400	-2.14921200	-0.00004300
C	0.89671500	0.00019200	-0.00005000
Cl	3.75635500	-0.00005600	-0.00001100
C	2.10697500	0.00009300	-0.00005200
H	-4.42097100	-0.00033600	0.00007100

Cartesian coordinates of the optimized geometry for **4a** at B3LYP-D3BJ/6-31G(d),def2-TZVP level of theory (number of imaginary frequencies = 0):

C	3.46080400	1.20865900	-0.00000200
C	2.06879800	1.21369100	-0.00002300
C	1.35604700	0.00002000	0.00000800
C	2.06875000	-1.21367300	0.00006000
C	3.46076000	-1.20869400	0.00008200
C	4.16086600	-0.00003200	0.00005000
H	4.00129000	2.15081800	-0.00002700
H	1.51917800	-2.14937600	0.00008400
H	4.00120500	-2.15087700	0.00012300
H	1.51925800	2.14941000	-0.00006400
C	-0.06985500	0.00005100	-0.00001400

C	-1.28088600	0.00004000	-0.00003400
H	5.24699200	-0.00005100	0.00006700
Br	-3.07541800	-0.00000800	-0.00002700

Cartesian coordinates of the optimized geometry for **7a** at B3LYP-D3BJ/6-31G(d),def2-TZVP level of theory (number of imaginary frequencies = 0):

C	-2.14367700	-1.20822200	-0.00010500
C	-0.75141000	-1.21196700	-0.00001200
C	-0.03562700	0.00032100	0.00010300
C	-0.75187700	1.21218300	0.00008000
C	-2.14423900	1.20777900	-0.00002000
C	-2.84516800	-0.00032700	-0.00010800
H	-2.68358100	-2.15098700	-0.00017600
H	-0.20301600	2.14850600	0.00013800
H	-2.68446100	2.15036300	-0.00003100
H	-0.20190100	-2.14789100	0.00000900
C	1.39227300	0.00040900	0.00010600
C	2.60409700	0.00030300	0.00013100
H	-3.93140000	-0.00063800	-0.00018200
C	4.06265700	-0.00020100	-0.00005000
H	4.46135800	-0.50195700	0.89046700
H	4.46089000	-0.52101500	-0.87974900
H	4.45994300	1.02194900	-0.01122500

Cartesian coordinates of the optimized geometry for **7c** at B3LYP-D3BJ/6-31G(d),def2-TZVP level of theory (number of imaginary frequencies = 0):

C	1.08039000	1.41552000	-0.00000100
C	-0.30147800	1.31642200	0.00000100
C	-0.93827300	0.05737300	0.00000500
C	-0.13153500	-1.09155000	0.00000800
C	1.25957100	-1.00100600	0.00000700
C	1.87286300	0.25752400	0.00000200
H	1.57604400	2.38084000	-0.00000400
H	-0.60492700	-2.06823400	0.00001200
H	1.84943700	-1.90983300	0.00001100
H	-0.90985000	2.21512900	0.00000000
C	-2.36158900	-0.04330700	0.00000500
C	-3.57090700	-0.12524900	0.00000700
O	3.22061900	0.46226200	0.00000200
C	4.06571900	-0.67553000	-0.00003700
H	3.90948400	-1.29302800	0.89437300
H	3.90943000	-1.29300500	-0.89445400
H	5.08707300	-0.29124200	-0.00006300
C	-5.02621700	-0.22412000	0.00001400
H	-5.46006600	0.25632100	0.88606900
H	-5.46028900	0.25975400	-0.88405700
H	-5.35254700	-1.27126600	-0.00197400

Cartesian coordinates of the optimized geometry for **12a** at B3LYP-D3BJ/6-31G(d),def2-TZVP level of theory (number of imaginary frequencies = 0):

C	0.00031100	1.45937800	-0.00000200
H	-0.92482700	2.03034700	-0.00003700
H	0.92566100	2.03000900	-0.00003600
C	0.00003000	0.12314600	0.00005200
C	-1.27649800	-0.67855800	-0.00000700
H	-1.32883300	-1.33441500	0.88015100
H	-2.16159000	-0.03519100	-0.00007500
H	-1.32872800	-1.33444400	-0.88015200
C	1.27620700	-0.67903700	-0.00001000
H	1.32830700	-1.33483000	0.88021400

H	1.32814200	-1.33502300	-0.88009800
H	2.16156500	-0.03602800	-0.00016100

Cartesian coordinates of the optimized geometry for **17a** at B3LYP-D3BJ/6-31G(d),def2-TZVP level of theory (number of imaginary frequencies = 0):

C	2.87308600	0.25667500	0.37740100
C	2.51915900	1.13196800	1.18526800
C	3.30327600	-0.78360900	-0.49623600
Cl	2.76091800	2.42751500	2.20365300
C	3.91910400	-1.93229200	0.03960500
C	3.08825200	-0.67692100	-1.88504300
C	3.49150600	-1.70945900	-2.72334400
C	4.10684300	-2.84660500	-2.18952400
C	4.32152700	-2.95536600	-0.81227900
H	4.80178300	-3.83862500	-0.40387500
H	4.07496800	-2.00523300	1.11073600
H	2.60273100	0.20917700	-2.27760700
H	3.32987800	-1.63036500	-3.79362800
Au	0.55962900	0.22191200	0.70774200
H	4.42054700	-3.64983100	-2.84900000
P	-1.54024700	-0.76166200	0.45024400
C	-2.42392100	-0.69082700	2.12326700
C	-1.20923400	-2.51489100	-0.22043600
C	-2.38626600	0.79149900	2.54770600
H	-1.36103700	1.15543900	2.67473600
H	-2.90327700	0.89904800	3.50786100
H	-2.89617800	1.43190000	1.82077000
C	-3.89185100	-1.14855700	2.09746800
H	-4.27914500	-1.11234900	3.12211800
H	-4.01281100	-2.17316100	1.73938700
H	-4.51260900	-0.48602400	1.49062000
C	-1.63365700	-1.53603100	3.13762100
H	-0.57080000	-1.27194700	3.15451400
H	-1.72385800	-2.60719500	2.93731400
H	-2.03874900	-1.35430600	4.13936100
C	0.02640800	-3.10717700	0.48827800
H	0.92795800	-2.51927100	0.29430000
H	0.19851100	-4.11612200	0.09631800
H	-0.10373400	-3.18950100	1.56930200
C	-2.38582000	-3.48972100	-0.05249100
H	-2.62178400	-3.67349000	0.99894200
H	-2.10363800	-4.45178500	-0.49542600
H	-3.28941500	-3.15536000	-0.56523200
C	-0.87600300	-2.34149800	-1.71549000
H	-0.05133000	-1.63517700	-1.86586100
H	-1.73705800	-1.99734400	-2.29326900
H	-0.55917100	-3.31025900	-2.11812700
C	-2.61516000	0.07613600	-0.78017500
C	-3.86617900	-0.50421000	-1.06895900
C	-2.21321100	1.21760500	-1.51422900
C	-4.70378100	0.01213000	-2.05214400
H	-4.19579900	-1.37672500	-0.52316200
C	-3.06947600	1.71749700	-2.50881200
C	-4.30057700	1.12943100	-2.78144100
H	-5.66161900	-0.45974800	-2.24676700
H	-2.75340200	2.59423100	-3.06519200
H	-4.94032500	1.54331900	-3.55457900
C	-0.93427900	1.96402200	-1.32630400
C	0.07784300	1.87187600	-2.29275500
C	-0.77224400	2.86062100	-0.25795600
C	1.23594300	2.64474400	-2.18270000
H	-0.05343100	1.19730800	-3.13393300
C	0.38391800	3.63693200	-0.15276300
H	-1.56614200	2.96547100	0.47455100

C	1.39333700	3.52618100	-1.11145500
H	2.00668500	2.57444100	-2.94508800
H	0.48968500	4.33393900	0.67250800
H	2.28874600	4.13475300	-1.03245400

Cartesian coordinates of the optimized geometry for **17b** at B3LYP-D3BJ/6-31G(d),def2-TZVP level of theory (number of imaginary frequencies = 0):

C	2.69894500	0.33727000	-0.02827500
C	2.61801900	-0.79057400	-0.53815600
C	2.87814800	1.64902300	0.50508400
C	3.40551900	2.66537400	-0.31495900
C	2.49930600	1.93370200	1.83152900
C	2.65634100	3.22222500	2.32899600
C	3.18586600	4.22885300	1.51520900
C	3.56092300	3.94917200	0.19799900
H	3.97345400	4.73286100	-0.42914700
H	3.68804800	2.43595200	-1.33698200
H	2.08484200	1.14147500	2.44431400
H	2.36883100	3.44447500	3.35169700
Au	0.48259700	-0.16190600	-0.48607000
H	3.30689700	5.23329300	1.90880000
P	-1.76182800	0.42570500	-0.69675100
C	-2.41101300	-0.33323900	-2.30538400
C	-1.80057200	2.32951800	-0.62165700
C	-2.08655200	-1.83813200	-2.21213900
H	-1.00915300	-2.02124400	-2.13945900
H	-2.45339800	-2.33700400	-3.11620100
H	-2.57792200	-2.30348300	-1.35161300
C	-3.92499500	-0.17828000	-2.52609200
H	-4.17411900	-0.60896100	-3.50266900
H	-4.24967400	0.86436900	-2.53716300
H	-4.50192500	-0.72034800	-1.77380900
C	-1.64655300	0.28482600	-3.48921800
H	-0.56127600	0.23560600	-3.34927600
H	-1.92909800	1.32716800	-3.66044000
H	-1.89190600	-0.27529600	-4.39844900
C	-0.60054300	2.89513200	-1.40911300
H	0.35407500	2.58085800	-0.97774200
H	-0.64138900	3.98932300	-1.36155900
H	-0.61201000	2.60869600	-2.46270600
C	-3.08970600	2.96037300	-1.17199500
H	-3.22497500	2.75769300	-2.23763900
H	-3.01990300	4.04785400	-1.05416500
H	-3.98290400	2.63811100	-0.63389100
C	-1.62389900	2.69856400	0.86469200
H	-0.71743800	2.24917100	1.28657800
H	-2.47872500	2.39067100	1.47149900
H	-1.52160200	3.78669200	0.94524000
C	-2.82987100	-0.17624700	0.66950500
C	-4.18272400	0.21737100	0.67380300
C	-2.34342900	-0.93894400	1.75796400
C	-5.04016600	-0.11935200	1.71601300
H	-4.57819300	0.80017700	-0.14584900
C	-3.22315900	-1.26054700	2.80432900
C	-4.55569300	-0.86047900	2.79253500
H	-6.07714100	0.19964900	1.68562100
H	-2.84146800	-1.84712700	3.63400500
H	-5.21016700	-1.12903900	3.61586500
C	-0.94935500	-1.45247800	1.90335900
C	-0.08522900	-0.87420000	2.84457500
C	-0.52407800	-2.59231800	1.20263200
C	1.18488700	-1.41156700	3.06506300
H	-0.41892500	-0.00666400	3.40663200
C	0.74335200	-3.13224800	1.42975100

H	-1.19959800	-3.06785900	0.49886900
C	1.60354700	-2.53898800	2.35599200
H	1.84052700	-0.96209100	3.80550100
H	1.05355800	-4.02028500	0.88857400
H	2.58699000	-2.96218800	2.53507500
Br	3.14897300	-2.40115400	-1.17745500

Cartesian coordinates of the optimized geometry for **18a** at B3LYP-D3BJ/6-31G(d),def2-TZVP level of theory (number of imaginary frequencies = 0):

C	-1.55838600	-1.14469300	-0.08024000
C	-2.19089300	-1.41020200	-1.26211800
C	-2.29239400	-1.20968100	1.19717200
Cl	-1.34967500	-1.26892000	-2.81284500
C	-3.20555000	-2.23463600	1.51260100
C	-2.06713400	-0.20158500	2.15518600
C	-2.77318700	-0.18602100	3.35660300
C	-3.68700900	-1.20071700	3.64839300
C	-3.88660300	-2.23458200	2.72743700
H	-4.57042800	-3.04550700	2.96012800
H	-3.35531600	-3.05354500	0.81755200
H	-1.34814600	0.57825000	1.93199200
H	-2.59502400	0.61019600	4.07352100
Au	0.46224700	-0.69995700	-0.01414300
H	-4.22266300	-1.20159000	4.59278300
P	2.80620600	-0.45503800	0.06743100
C	3.49794800	-1.36614100	-1.44793300
C	3.39719800	-1.14437100	1.74132300
C	2.78089200	-0.73850700	-2.66136000
H	1.69659200	-0.87409900	-2.60988400
H	3.14181600	-1.22153600	-3.57702800
H	2.99580000	0.33242900	-2.74302400
C	5.01371400	-1.24860500	-1.67573800
H	5.28032000	-1.87073200	-2.53842600
H	5.60291800	-1.60043500	-0.82623800
H	5.31050000	-0.22418300	-1.91069200
C	3.10981900	-2.85187400	-1.33854300
H	2.04172700	-2.98178200	-1.13485600
H	3.67822900	-3.36566800	-0.55788400
H	3.33470200	-3.34860600	-2.28953100
C	2.54006100	-2.37790200	2.09303600
H	1.47695800	-2.12709100	2.15393100
H	2.85714700	-2.75710800	3.07171500
H	2.65468600	-3.18818300	1.36950000
C	4.87876000	-1.54408500	1.81104000
H	5.11529300	-2.36386700	1.12731000
H	5.09777800	-1.89472400	2.82668100
H	5.55342700	-0.70986000	1.60905000
C	3.10323300	-0.03719800	2.77313700
H	2.05975500	0.29470800	2.71880400
H	3.74844100	0.83418000	2.63568600
H	3.27568700	-0.43513400	3.77986700
C	3.46173100	1.26389900	-0.02563500
C	4.85523800	1.45570400	0.04779200
C	2.63016400	2.40687400	-0.08574100
C	5.42583900	2.72439000	0.05785700
H	5.51218000	0.60018100	0.10579800
C	3.22401800	3.68041600	-0.06730500
C	4.60311900	3.84771600	0.00053900
H	6.50471000	2.83044500	0.11471300
H	2.57565300	4.54975000	-0.11552400
H	5.02987600	4.84596400	0.00902100
C	1.13878000	2.39836200	-0.14649700
C	0.40714400	2.84119300	0.96531900
C	0.45073700	2.08087400	-1.32702100

C	-0.98087300	2.96282400	0.89912900
H	0.93649800	3.09787000	1.87822000
C	-0.93895300	2.20603000	-1.39240000
H	1.00798400	1.75572000	-2.19936900
C	-1.65624800	2.64784200	-0.28097000
H	-1.53382200	3.31241800	1.76654200
H	-1.45453500	1.95610100	-2.31468600
H	-2.73301300	2.75638300	-0.33375700
C	-4.17762400	-0.62296300	-1.17974100
C	-3.63891300	-1.75426500	-1.47516600
C	-4.65671500	0.60546400	-0.76625500
C	-4.86979600	1.64533900	-1.71498100
C	-4.93215100	0.82679900	0.61295900
C	-5.33995700	2.87405000	-1.28646400
H	-4.65548600	1.45682500	-2.76138600
C	-5.40122200	2.06649000	1.01937400
H	-4.77921000	0.02264900	1.32146600
C	-5.60370200	3.08279200	0.07707400
H	-5.50539100	3.67421200	-1.99997600
H	-5.61371900	2.24639800	2.06784100
H	-5.97443900	4.04937200	0.40495300
C	-4.15948900	-3.05616700	-1.99861800
H	-3.72969700	-3.24559100	-2.98768800
H	-3.84484600	-3.87103500	-1.33840500
H	-5.24868100	-3.04717500	-2.07399400

Cartesian coordinates of the optimized geometry for **19a** at B3LYP-D3BJ/6-31G(d),def2-TZVP level of theory (number of imaginary frequencies = 0):

C	1.32341700	1.14855300	-0.16669900
C	2.42591100	0.38135200	-0.20898800
C	1.38585300	2.59972700	0.08194600
Cl	2.32615400	-1.29236800	-0.84051600
C	2.12906100	3.43120100	-0.77547900
C	0.65030500	3.19044700	1.12540100
C	0.70973600	4.56481200	1.34076500
C	1.46793000	5.38003500	0.49549200
C	2.16636800	4.80937100	-0.57116300
H	2.73869500	5.43918500	-1.24614900
H	2.66341100	2.98554500	-1.60976900
H	0.04942900	2.55621900	1.76772800
H	0.15328800	5.00479300	2.16330500
Au	-0.57155100	0.35818000	-0.44298100
H	1.50043900	6.45312200	0.65705400
P	-2.77108900	-0.39465000	-0.80699200
C	-2.72356900	-1.76754000	-2.11377500
C	-3.80112900	1.12829200	-1.31013500
C	-1.72706700	-2.81330200	-1.57309500
H	-0.73080700	-2.38734300	-1.42007000
H	-1.64275300	-3.63227600	-2.29726900
H	-2.07253300	-3.24052100	-0.62584200
C	-4.06083200	-2.47483600	-2.38563900
H	-3.90626700	-3.21141100	-3.18323200
H	-4.84449700	-1.79182400	-2.72000600
H	-4.41841300	-3.01628400	-1.50681700
C	-2.17454300	-1.17276600	-3.42284100
H	-1.23924800	-0.62634800	-3.26135000
H	-2.89303700	-0.49961100	-3.89919200
H	-1.97186900	-1.98834900	-4.12689900
C	-2.92585900	2.03121800	-2.20493800
H	-2.01933100	2.35719000	-1.68657100
H	-3.50378900	2.92361300	-2.47298600
H	-2.62562300	1.53843500	-3.13251900
C	-5.11272600	0.83559800	-2.05492100
H	-4.93801800	0.34333600	-3.01533500

H	-5.61364000	1.78825900	-2.26461000
H	-5.80950600	0.23107300	-1.47122100
C	-4.09709900	1.88549300	-0.00034100
H	-3.17874300	2.07853800	0.56637100
H	-4.79019900	1.33888400	0.64392300
H	-4.54826300	2.85418300	-0.24469300
C	-3.61288500	-1.11656900	0.66430500
C	-4.97005200	-1.47660900	0.55287700
C	-2.98329700	-1.25365900	1.92396100
C	-5.69918200	-1.94948000	1.63900100
H	-5.47346000	-1.38161100	-0.39855700
C	-3.73816200	-1.72496300	3.01154200
C	-5.07929000	-2.07022900	2.88140700
H	-6.74401700	-2.21596000	1.51316400
H	-3.24454300	-1.82590500	3.97311600
H	-5.63302300	-2.43324400	3.74185900
C	-1.55267500	-0.94657000	2.22595600
C	-1.22827400	0.18076100	2.99513900
C	-0.53318800	-1.84673000	1.88344800
C	0.08809000	0.41048100	3.39830400
H	-2.01643100	0.87138500	3.28074400
C	0.78079800	-1.61958600	2.29221900
H	-0.77685400	-2.73319300	1.30767300
C	1.09584300	-0.48858800	3.04565000
H	0.32298000	1.28563900	3.99728800
H	1.55577500	-2.32723500	2.01621200
H	2.11843300	-0.31438900	3.36863100
C	4.72670400	-0.22217600	0.00186200
C	3.79643600	0.65667700	0.26038500
C	5.77060400	-1.09217700	-0.19158800
C	6.63842100	-0.93211300	-1.31518100
C	6.00060400	-2.15815600	0.73183800
C	7.69538300	-1.80514100	-1.49662600
H	6.44802600	-0.12247900	-2.01113800
C	7.06690500	-3.01581000	0.53838800
H	5.32529700	-2.27489000	1.57232400
C	7.90815100	-2.83961600	-0.57233400
H	8.35921700	-1.69275900	-2.34708300
H	7.25282700	-3.82568400	1.23566500
H	8.74108400	-3.52066400	-0.72030200
C	4.16670000	1.84952500	1.13639200
H	4.21507800	2.76637900	0.54844600
H	3.39441200	1.97558700	1.89861100
H	5.13033500	1.68282100	1.62342900

Cartesian coordinates of the optimized geometry for **20a** at B3LYP-D3BJ/6-31G(d),def2-TZVP level of theory (number of imaginary frequencies = 1):

C	1.22903500	1.41324700	-0.24047100
C	2.37022700	0.75171300	-0.35287300
C	1.30114400	2.84244200	0.14679400
Cl	2.41576600	-1.05345900	-1.02800700
C	2.12993700	3.73564600	-0.55347400
C	0.50224600	3.33653700	1.19282600
C	0.57191700	4.67823700	1.55860500
C	1.41112900	5.55603000	0.86664200
C	2.18008200	5.08273700	-0.19767100
H	2.81546400	5.76362200	-0.75630400
H	2.70564600	3.37236500	-1.39957100
H	-0.15995200	2.65628400	1.71647700
H	-0.04045900	5.04422400	2.37748300
Au	-0.62352000	0.51131900	-0.45933900
H	1.45172400	6.60458900	1.14486500
P	-2.76916300	-0.40251900	-0.73681000
C	-2.70470100	-1.68479500	-2.13079500

C	-3.95020000	1.05574700	-1.06871100
C	-1.59290900	-2.67400900	-1.72636700
H	-0.62432400	-2.17571800	-1.61967900
H	-1.49974100	-3.44297300	-2.50219500
H	-1.82898200	-3.17693200	-0.78304100
C	-4.00035000	-2.48202900	-2.34975100
H	-3.84847800	-3.16372000	-3.19506400
H	-4.85504700	-1.84727600	-2.59196300
H	-4.25197400	-3.09184400	-1.47906700
C	-2.30278900	-0.97151900	-3.43378000
H	-1.40230500	-0.36176800	-3.30359300
H	-3.10335700	-0.33078800	-3.81407300
H	-2.09255300	-1.72476000	-4.20202900
C	-3.20737900	2.08939000	-1.94209800
H	-2.30548900	2.46341300	-1.44855500
H	-3.87462100	2.94097700	-2.11952100
H	-2.91712700	1.68636900	-2.91512500
C	-5.27422500	0.70158200	-1.76391300
H	-5.11524100	0.29382100	-2.76559000
H	-5.86275200	1.61977900	-1.87671400
H	-5.88503100	0.00046700	-1.19216500
C	-4.23032100	1.69380400	0.30622700
H	-3.29957200	1.92668400	0.83677500
H	-4.83674500	1.04740800	0.94527000
H	-4.77284300	2.63441200	0.15699700
C	-3.42966800	-1.29273000	0.73348000
C	-4.76519100	-1.73954600	0.70371200
C	-2.67255500	-1.51565700	1.90700600
C	-5.35098400	-2.38228100	1.78920300
H	-5.36372200	-1.58316500	-0.18250200
C	-3.28135000	-2.16538100	2.99383800
C	-4.60327100	-2.59590400	2.94590100
H	-6.38390700	-2.71069600	1.72912000
H	-2.69051100	-2.33118800	3.88929300
H	-5.04392200	-3.09430600	3.80384700
C	-1.24551400	-1.12177200	2.11882600
C	-0.93636300	0.03936400	2.84334700
C	-0.20358300	-1.98057900	1.74209700
C	0.38683700	0.34307700	3.16748700
H	-1.74192600	0.69560600	3.16004400
C	1.11804900	-1.67830500	2.07146700
H	-0.43510800	-2.89399000	1.20367900
C	1.41753800	-0.51431600	2.77979900
H	0.61104700	1.24466300	3.72943400
H	1.91132900	-2.35956200	1.77877500
H	2.44613200	-0.28193500	3.04059900
C	4.27939600	-0.43934700	-0.33767100
C	3.77834900	0.75408100	-0.01260200
C	5.37387200	-1.34483700	-0.36705600
C	6.25504300	-1.35602100	-1.47109100
C	5.59207400	-2.22551200	0.71624900
C	7.34534400	-2.21703700	-1.47529700
H	6.07220800	-0.68359700	-2.30269100
C	6.68493300	-3.08195500	0.70128200
H	4.90357100	-2.21458600	1.55517300
C	7.55830400	-3.07750700	-0.39274200
H	8.02974400	-2.22192300	-2.31728000
H	6.86152100	-3.75264200	1.53581100
H	8.41051000	-3.75023600	-0.40149600
C	4.53209400	1.85638300	0.67129200
H	4.66185700	2.71393300	0.00630600
H	3.97032800	2.20149900	1.54457500
H	5.51492200	1.50205600	0.99145500

Cartesian coordinates of the optimized geometry for **21a** at B3LYP-D3BJ/6-31G(d),def2-TZVP level of theory (number of imaginary frequencies = 0):

C	0.76725500	2.42632300	0.00819600
C	1.67919600	1.58601400	-0.09038300
C	-0.21758900	3.45368300	0.14311000
Cl	2.36888700	-1.10524400	-1.39601800
C	-0.68687400	3.80226500	1.42379800
C	-0.74582000	4.08788200	-0.99733900
C	-1.73232100	5.05772700	-0.85186700
C	-2.19640700	5.40148300	0.42134500
C	-1.67118200	4.77643200	1.55601700
H	-2.03222100	5.04758100	2.54306500
H	-0.28157200	3.29435900	2.29199000
H	-0.37739400	3.81064300	-1.97961400
H	-2.13883500	5.54853800	-1.73043000
Au	-0.21121100	0.37498200	-0.34499900
C	3.03152700	1.11735800	0.01326800
C	3.48176900	-0.03450200	-0.53648800
C	4.86579000	-0.53734200	-0.49031900
C	5.93394900	0.29168400	-0.86879200
C	5.13295300	-1.85191600	-0.07260900
C	7.24442000	-0.17866400	-0.80849900
H	5.73156400	1.29455800	-1.23030600
C	6.44402300	-2.31374200	-0.00427700
H	4.31069000	-2.50676800	0.19837800
C	7.50258800	-1.47863000	-0.37113000
H	8.06187500	0.46782100	-1.11222300
H	6.64076700	-3.32744800	0.33064000
H	8.52386100	-1.84385000	-0.32481500
H	-2.96684300	6.15863600	0.52937900
P	-2.15661100	-0.84634300	-0.70956800
C	-3.60424500	0.38142000	-0.70576300
C	-1.94062700	-1.81094700	-2.33513200
C	3.91102700	2.02819100	0.84945500
H	4.14364400	2.95205600	0.30735700
H	3.37746100	2.30972800	1.76258000
H	4.84756300	1.54129500	1.12253400
C	-1.15829000	-0.92198600	-3.32526100
H	-0.16665200	-0.66200300	-2.94536200
H	-1.02457200	-1.47666100	-4.26104100
H	-1.68552100	0.00530000	-3.56155100
C	-1.08016700	-3.04249400	-1.99017200
H	-0.79307100	-3.54494000	-2.92077100
H	-0.15968300	-2.75993700	-1.46698600
H	-1.61938200	-3.76011600	-1.36716300
C	-3.24441300	-2.25526100	-3.01861900
H	-2.98396600	-2.82171500	-3.92019900
H	-3.85545900	-2.91151200	-2.39630300
H	-3.85319700	-1.40452500	-3.33528700
C	-3.43664300	1.35762500	-1.88236400
H	-4.17932900	2.15779200	-1.78532100
H	-2.44662100	1.82458800	-1.88652400
H	-3.60058000	0.87207100	-2.84834600
C	-3.49942400	1.15953800	0.62121000
H	-2.57086000	1.73194900	0.69153300
H	-4.33535000	1.86595000	0.68141600
H	-3.56379700	0.49064100	1.48520500
C	-4.98883200	-0.28509600	-0.76670200
H	-5.74937800	0.50416600	-0.78322100
H	-5.13078700	-0.89395300	-1.66157000
H	-5.17860600	-0.90409700	0.11308100
C	-2.52217000	-2.05734400	0.62120800
C	-3.59129700	-2.95455600	0.43318000
C	-1.78731800	-2.12298500	1.82780300
C	-3.93826900	-3.89374700	1.39845900
H	-4.16856000	-2.91955300	-0.48026200

C	-2.15766700	-3.07349700	2.79325300
C	-3.21710600	-3.95219900	2.58997900
H	-4.76688300	-4.57151800	1.21913800
H	-1.58917700	-3.11554900	3.71697500
H	-3.47530700	-4.67726200	3.35552900
C	-0.61683000	-1.26475500	2.18890900
C	0.68818200	-1.68588300	1.88522800
C	-0.79373800	-0.11212700	2.96759500
C	1.79125400	-0.96603100	2.34750300
H	0.83219300	-2.58901100	1.30015400
C	0.31059300	0.60904400	3.42470100
H	-1.79860200	0.20737200	3.22520500
C	1.60429900	0.18236600	3.11891600
H	2.79452400	-1.30606000	2.11155300
H	0.15970800	1.48907000	4.04360800
H	2.46257500	0.73108900	3.49421500

Cartesian coordinates of the optimized geometry for **22a** at B3LYP-D3BJ/6-31G(d),def2-TZVP level of theory (number of imaginary frequencies = 0):

C	-1.57994000	-0.78047900	-1.18351000
C	-2.40222400	-1.50222700	-0.36623600
Au	0.39437300	-0.43018000	-0.74540600
P	2.68156400	-0.27403200	-0.21552600
C	2.99204700	-1.40880900	1.27253500
C	3.66672400	-0.75074800	-1.77481700
C	2.01184700	-0.92824800	2.36098000
H	0.97139000	-0.99450800	2.03384000
H	2.12654700	-1.56247000	3.24789400
H	2.21840900	0.10497300	2.65779200
C	4.41778900	-1.35222000	1.84188500
H	4.47822100	-2.04034900	2.69354900
H	5.17723800	-1.65850600	1.11998400
H	4.66710600	-0.35397100	2.20919000
C	2.63801400	-2.85291000	0.87640700
H	1.63116400	-2.92129900	0.45202400
H	3.35062800	-3.26979500	0.15894800
H	2.66588900	-3.48332000	1.77300700
C	2.87113500	-1.84213100	-2.52348400
H	1.88445300	-1.48411500	-2.83108300
H	3.42672100	-2.12412600	-3.42562700
H	2.73075400	-2.74418800	-1.92271400
C	5.09031100	-1.28245300	-1.54010200
H	5.08977900	-2.21720500	-0.97345800
H	5.54458200	-1.49403000	-2.51533700
H	5.74316100	-0.56938600	-1.03358300
C	3.71040600	0.51077500	-2.65881300
H	2.70650400	0.91988800	-2.82226000
H	4.33420200	1.29736300	-2.22753300
H	4.12413700	0.24444200	-3.63828200
C	3.27342500	1.38110300	0.33284500
C	4.65783400	1.58619100	0.48608200
C	2.39661100	2.44029000	0.66364800
C	5.17700000	2.78971300	0.95181700
H	5.34773700	0.78931500	0.24606800
C	2.93758400	3.64319700	1.14766200
C	4.30907700	3.82604800	1.29152100
H	6.25095700	2.91151000	1.05273500
H	2.25460900	4.44830800	1.40026400
H	4.69562300	4.77058000	1.66180700
C	0.90817100	2.41086700	0.53719300
C	0.29032100	2.65616500	-0.69934000
C	0.10798800	2.27718900	1.67902400
C	-1.09870900	2.76011900	-0.79020600
H	0.90613100	2.78538900	-1.58428400

C	-1.28081200	2.38147100	1.58652200
H	0.57943500	2.10248800	2.64123900
C	-1.88723200	2.62261500	0.35329800
H	-1.56109700	2.94848800	-1.75386800
H	-1.88745700	2.29397800	2.48392300
H	-2.96479600	2.71988800	0.28579000
C	-4.40114000	-0.78341100	-0.43176900
C	-3.83502600	-1.89663600	-0.71424600
Cl	-2.23027400	-0.17410400	-2.71027600
C	-1.93021900	-1.97034600	0.96824900
C	-1.94902500	-1.10644800	2.07167000
C	-1.46265000	-3.28186300	1.12967900
C	-1.51030900	-1.54831700	3.31825800
H	-2.27991700	-0.08316400	1.93442100
C	-1.01281600	-3.71890900	2.37674000
H	-1.42793400	-3.94774300	0.27225800
C	-1.04247700	-2.85594900	3.47327600
H	-1.52028300	-0.86991400	4.16636600
H	-0.64128600	-4.73276200	2.49048400
H	-0.69701200	-3.19840400	4.44396200
C	-4.88711200	0.48088700	-0.12556400
C	-5.19794200	0.81697600	1.21951800
C	-5.06934100	1.43814000	-1.15897000
C	-5.66899500	2.08539200	1.51575100
H	-5.05014700	0.07387100	1.99577000
C	-5.55766100	2.69776700	-0.84484100
H	-4.82037300	1.16526700	-2.17741800
C	-5.85290100	3.02044600	0.48603100
H	-5.90446400	2.35369200	2.54026500
H	-5.70678100	3.43359700	-1.62797200
H	-6.23117600	4.00979100	0.72492800
C	-4.26466800	-3.25686300	-1.15557300
H	-5.34725500	-3.31180000	-1.28648100
H	-3.76865100	-3.50054100	-2.10106900
H	-3.95156000	-3.99505600	-0.40962900

Cartesian coordinates of the optimized geometry for **23a** at B3LYP-D3BJ/6-31G(d),def2-TZVP level of theory (number of imaginary frequencies = 0):

C	1.54738700	0.31882700	-0.47051800
C	2.29413800	1.27246400	0.13334200
Au	-0.44953800	-0.00509300	-0.64146400
P	-2.79387900	-0.02554400	-0.67979600
C	-3.34724500	1.72744500	-0.20795400
C	-3.37896800	-0.59963700	-2.39692200
C	-2.63899500	2.04190900	1.12622900
H	-1.54986100	2.01718500	1.03330200
H	-2.92316600	3.05001600	1.44910300
H	-2.93988500	1.34149300	1.91190000
C	-4.86132700	1.89034900	-0.00122700
H	-5.06523200	2.93964300	0.24276700
H	-5.44222300	1.64344500	-0.89214800
H	-5.22344600	1.28457600	0.83256800
C	-2.85767300	2.70861000	-1.28754300
H	-1.78611900	2.59897800	-1.48108700
H	-3.39929200	2.58725800	-2.22992400
H	-3.03012200	3.73325700	-0.93822700
C	-2.39135200	-0.04560300	-3.44551800
H	-1.37384000	-0.40884100	-3.27300200
H	-2.70831900	-0.38298800	-4.43921200
H	-2.36219200	1.04652100	-3.45824100
C	-4.79918700	-0.16920100	-2.79714300
H	-4.89464900	0.91746300	-2.86599800
H	-5.01818600	-0.57857100	-3.79036700
H	-5.56864600	-0.54604500	-2.12073600

C	-3.27446600	-2.13723900	-2.39132100
H	-2.27769200	-2.47090200	-2.07954900
H	-4.01160500	-2.59840100	-1.72963300
H	-3.44728100	-2.50856400	-3.40795500
C	-3.56687200	-1.13804800	0.56426300
C	-4.96602200	-1.29695200	0.54592300
C	-2.82073000	-1.84631500	1.53458900
C	-5.62321400	-2.12491600	1.44990800
H	-5.55739400	-0.76412900	-0.18554600
C	-3.50195200	-2.67455500	2.44170400
C	-4.88531100	-2.81879800	2.40751500
H	-6.70318000	-2.22443600	1.40443600
H	-2.91974800	-3.21286000	3.18307800
H	-5.38144700	-3.46850200	3.12186700
C	-1.33384400	-1.80959000	1.69090000
C	-0.54069100	-2.79814900	1.08817200
C	-0.72553500	-0.88487600	2.55121900
C	0.83245800	-2.85258400	1.33277100
H	-1.01026800	-3.53160500	0.43935000
C	0.64779200	-0.94031500	2.79361400
H	-1.33309400	-0.12806000	3.03662400
C	1.43002500	-1.92317000	2.18645900
H	1.43011600	-3.63085900	0.86673800
H	1.10128600	-0.22336000	3.47102000
H	2.49696600	-1.97031500	2.38572900
C	3.73301900	1.06849400	0.27176600
C	4.20876500	-0.08228300	-0.25593500
C	5.51735200	-0.70179900	-0.41443100
C	5.72235600	-2.05332600	-0.08564500
C	6.59495200	0.06497200	-0.89168700
C	6.98457200	-2.62221600	-0.22214600
H	4.89472100	-2.64885500	0.28938500
C	7.85910300	-0.50779800	-1.01103800
H	6.43219400	1.09795300	-1.18048500
C	8.05570000	-1.84967400	-0.67947200
H	7.13600900	-3.66557600	0.03572400
H	8.68693000	0.09042300	-1.37834800
H	9.04027200	-2.29507400	-0.78179400
Cl	2.80478400	-0.98992200	-0.97403200
C	1.56588200	2.46249400	0.65285600
C	1.42935000	2.67048900	2.03266300
C	0.95995700	3.35721400	-0.23994500
C	0.70172700	3.75931600	2.51053100
H	1.87694400	1.96682200	2.72768400
C	0.23231300	4.44605000	0.24064500
H	1.06444000	3.19268000	-1.30774400
C	0.10244800	4.64893700	1.61536600
H	0.59855500	3.91098900	3.58068500
H	-0.23094300	5.13499900	-0.45903800
H	-0.46271400	5.49709900	1.98961500
C	4.61307000	2.07231800	0.96310500
H	4.68142000	2.98982600	0.36715300
H	4.18736400	2.35219800	1.93081200
H	5.61983100	1.68220700	1.12008400

Cartesian coordinates of the optimized geometry for **24a** at B3LYP-D3BJ/6-31G(d),def2-TZVP level of theory (number of imaginary frequencies = 1):

C	1.14254200	1.12434300	-0.16282700
C	2.36145400	0.83717000	0.02594800
Au	-0.81881700	0.62734800	-0.32025300
C	3.68427400	0.32970000	0.16397400
C	4.05262600	-0.74282400	-0.58952100
C	5.37381000	-1.38746600	-0.59418200
C	6.53732600	-0.60508400	-0.69465000

C	5.49357500	-2.78554800	-0.51875600
C	7.79272500	-1.20855300	-0.69862800
H	6.45257900	0.47137100	-0.80073000
C	6.75031200	-3.38257300	-0.51235500
H	4.59999900	-3.39807200	-0.45924700
C	7.90231900	-2.59674300	-0.60102600
H	8.68386000	-0.59528400	-0.78913200
H	6.83194500	-4.46274100	-0.44243300
H	8.88123600	-3.06607200	-0.60381700
P	-3.06304100	0.00687700	-0.56560800
C	-4.15652800	1.45126500	-0.01097700
C	-3.31300500	-0.51764400	-2.37942100
C	1.87567000	2.50573700	0.26905900
C	2.38004300	3.38459000	-0.71098700
C	1.72991700	2.94116300	1.60361300
C	2.72538500	4.68423200	-0.35835200
H	2.49399900	3.02888700	-1.72968700
C	2.06293100	4.24552800	1.94264200
H	1.34415900	2.24418500	2.34020600
C	2.56301900	5.11386900	0.96358000
H	3.11727200	5.36462500	-1.10749100
H	1.94379100	4.59023600	2.96484700
H	2.83076800	6.13069700	1.23421100
Cl	2.85702800	-1.48579800	-1.64533300
C	4.56068600	0.97626300	1.21335100
H	4.90251200	1.96570000	0.88787800
H	3.99089800	1.11699800	2.13716000
H	5.43356200	0.36095200	1.43028500
C	-3.90638000	2.64643200	-0.94731100
H	-4.40132800	3.53235200	-0.53299200
H	-2.83898500	2.87214600	-1.04487400
H	-4.31833600	2.47855600	-1.94636200
C	-3.68007700	1.80748600	1.41183900
H	-2.62790600	2.10962900	1.42663900
H	-4.28088500	2.64279600	1.78977200
H	-3.80820800	0.96529100	2.09928600
C	-5.66083100	1.14001300	0.04959500
H	-6.18946700	2.05091300	0.35424800
H	-6.07111800	0.83154300	-0.91401500
H	-5.88510600	0.36874800	0.78992900
C	-2.47690000	0.42275100	-3.27309000
H	-1.41110300	0.36673000	-3.03252800
H	-2.60476100	0.11723600	-4.31807100
H	-2.78829200	1.46670000	-3.19136900
C	-2.73435900	-1.94165100	-2.49384700
H	-2.70969200	-2.22969200	-3.55104100
H	-1.70809300	-1.99016700	-2.11132500
H	-3.33661900	-2.67629100	-1.95397200
C	-4.76490200	-0.49673100	-2.88320100
H	-5.19082200	0.50981700	-2.86730700
H	-4.77409600	-0.83703300	-3.92533500
H	-5.42254300	-1.16512500	-2.32445800
C	-3.54518200	-1.42675100	0.47990100
C	-4.82851900	-1.98028900	0.30429700
C	-2.67857500	-2.02007600	1.42713100
C	-5.25776900	-3.08658100	1.02945800
H	-5.50855600	-1.54232300	-0.41269400
C	-3.13013000	-3.13764200	2.14937300
C	-4.40050400	-3.67176600	1.95974600
H	-6.25352500	-3.48649200	0.86570100
H	-2.45795300	-3.58582100	2.87435200
H	-4.71728100	-4.53661400	2.53442400
C	-1.28893200	-1.57376100	1.75739700
C	-1.06576400	-0.61505000	2.75705100
C	-0.18545100	-2.23717700	1.19774600
C	0.23118700	-0.32846600	3.18747600

H	-1.91382100	-0.11491300	3.21374000
C	1.11009100	-1.94714900	1.62623000
H	-0.35143300	-2.99122800	0.43421700
C	1.32081200	-0.99678600	2.62627300
H	0.38420000	0.39776900	3.98127000
H	1.95201200	-2.46818300	1.18211600
H	2.32841200	-0.78834400	2.97410400

Cartesian coordinates of the optimized geometry for **32a** at B3LYP-D3BJ/6-31G(d),def2-TZVP level of theory (number of imaginary frequencies = 1):

C	-1.61365700	-1.04721500	-0.12436600
C	-2.08124200	-0.95551500	-1.30930300
C	-2.13599800	-1.34634900	1.20868300
Cl	-1.82200700	-0.63282500	-2.92534600
C	-3.01726700	-2.41938800	1.41702900
C	-1.76693700	-0.54108400	2.29973800
C	-2.30982100	-0.77479700	3.56067900
C	-3.20375300	-1.83003500	3.75695200
C	-3.54597300	-2.65660100	2.68404300
H	-4.22701400	-3.48890300	2.83312400
H	-3.28249000	-3.06410200	0.58902800
H	-1.07995500	0.28111600	2.13988700
H	-2.02987500	-0.13565500	4.39269300
Au	0.48878500	-0.65103700	-0.22975300
H	-3.62192200	-2.01534500	4.74147100
P	2.80592300	-0.46880200	-0.04713900
C	3.57240100	-1.11446800	-1.65573700
C	3.29507100	-1.44174500	1.51528500
C	2.90181000	-0.30920400	-2.78707200
H	1.81835600	-0.46391700	-2.81141100
H	3.31493000	-0.63548600	-3.74844500
H	3.09732000	0.76328000	-2.68624800
C	5.09469300	-0.92874000	-1.77062600
H	5.42484000	-1.37717700	-2.71492800
H	5.64600200	-1.41983000	-0.96607900
H	5.37310400	0.12706000	-1.79568000
C	3.21713800	-2.60391400	-1.80781800
H	2.14313800	-2.78285400	-1.68858500
H	3.75546600	-3.22921400	-1.09013800
H	3.50398300	-2.93566100	-2.81226100
C	2.41822400	-2.70817700	1.60585200
H	1.35480000	-2.46022900	1.67495100
H	2.69543900	-3.25931800	2.51188800
H	2.55610200	-3.37805400	0.75430500
C	4.77101100	-1.86498000	1.58514600
H	5.03923400	-2.55626100	0.78192800
H	4.93615100	-2.38914800	2.53367800
H	5.45852800	-1.01740800	1.56557500
C	2.95251800	-0.52594400	2.70713500
H	1.91247000	-0.18229100	2.66344200
H	3.60289700	0.35076500	2.75404800
H	3.07664500	-1.09458500	3.63574900
C	3.42707700	1.24513700	0.19025100
C	4.80507700	1.42646000	0.42085600
C	2.58093000	2.37830200	0.23062200
C	5.34559500	2.67952600	0.68967800
H	5.47189600	0.57645500	0.39574800
C	3.14496800	3.63376800	0.51142900
C	4.50803100	3.79260500	0.73961600
H	6.41244300	2.78117900	0.86210100
H	2.48708400	4.49669500	0.53940400
H	4.91212600	4.77769100	0.95139800
C	1.10660600	2.37077500	-0.00553200
C	0.22437700	2.53235400	1.07317100

C	0.58591700	2.33802200	-1.30799400
C	-1.15070900	2.63596900	0.85463500
H	0.62540800	2.58083800	2.08173300
C	-0.78829000	2.45159300	-1.52357200
H	1.26391500	2.24348600	-2.15009100
C	-1.66089200	2.59370900	-0.44343000
H	-1.82758300	2.75557100	1.69505200
H	-1.17567300	2.43730700	-2.53751800
H	-2.72706200	2.68575800	-0.61024000
C	-4.55301300	-0.62988700	-1.03952500
C	-4.28298800	-1.71966800	-1.52755300
C	-4.82325900	0.64719400	-0.47954800
C	-5.15329500	1.72510900	-1.32523600
C	-4.75645300	0.84506600	0.91370200
C	-5.40465700	2.98157700	-0.78257400
H	-5.20992100	1.56230100	-2.39648500
C	-4.99910000	2.10856000	1.44281900
H	-4.51483400	0.01116600	1.56115900
C	-5.32132200	3.17653000	0.59969800
H	-5.66307900	3.80969600	-1.43493400
H	-4.94569300	2.25956300	2.51658300
H	-5.51430400	4.15905200	1.01942300
C	-4.23489400	-3.04078100	-2.15089800
H	-3.71524500	-2.99881200	-3.11436300
H	-3.71269500	-3.76538400	-1.51717400
H	-5.25119100	-3.40991000	-2.32872700

Cartesian coordinates of the optimized geometry for **33a** at B3LYP-D3BJ/6-31G(d),def2-TZVP level of theory (number of imaginary frequencies = 1):

C	-1.52469300	-0.71722600	-1.40903400
C	-2.08394500	-1.20382900	-0.35733900
Au	0.49540100	-0.46479600	-0.85113500
P	2.69378200	-0.37024600	-0.05867200
C	2.74399200	-1.53852800	1.44180900
C	3.88323600	-0.83534500	-1.46548000
C	1.61759300	-1.05633400	2.37873400
H	0.63334300	-1.13707300	1.91163600
H	1.61334200	-1.68078200	3.27968800
H	1.76829700	-0.01755200	2.68998000
C	4.06144400	-1.53569200	2.23444400
H	3.98116200	-2.28124000	3.03435000
H	4.92838500	-1.80175600	1.62679000
H	4.24685700	-0.56916800	2.70737700
C	2.43059400	-2.96692100	0.96289100
H	1.51517200	-3.00389300	0.36286200
H	3.24943100	-3.39309500	0.37624500
H	2.28409700	-3.60961300	1.83880300
C	3.21013000	-1.92527400	-2.32510300
H	2.26512400	-1.57551300	-2.75210000
H	3.88043500	-2.18433000	-3.15285300
H	3.00934100	-2.83907300	-1.76082500
C	5.26183500	-1.34753100	-1.01865900
H	5.19046900	-2.28096000	-0.45439600
H	5.86140000	-1.55337600	-1.91306200
H	5.81592700	-0.61842500	-0.42391100
C	4.04774200	0.43378100	-2.32355400
H	3.07837900	0.81902900	-2.65875000
H	4.56888100	1.23040000	-1.78646000
H	4.63204400	0.18463500	-3.21665300
C	3.20363400	1.27023800	0.59814700
C	4.51221800	1.41390700	1.09784300
C	2.31681200	2.36890800	0.70132300
C	4.93767200	2.58660200	1.71389700
H	5.21276100	0.59449300	1.01572300

C	2.75638000	3.53422800	1.35046100
C	4.04697900	3.64918800	1.85753700
H	5.95522200	2.66357500	2.08393100
H	2.06791600	4.37005500	1.42665600
H	4.35868900	4.56730300	2.34588700
C	0.94943900	2.42989300	0.10691300
C	0.80237400	2.59782300	-1.27995300
C	-0.19404500	2.44923900	0.91734900
C	-0.46164400	2.76949600	-1.84410900
H	1.68785200	2.61627200	-1.90754700
C	-1.46032500	2.61625100	0.35049100
H	-0.08450500	2.33305600	1.99180600
C	-1.59452900	2.77731500	-1.02875900
H	-0.55985400	2.90112600	-2.91710800
H	-2.34405000	2.63901100	0.98226000
H	-2.57768600	2.91314100	-1.46340800
C	-4.56682700	-0.83788200	-0.51842400
C	-4.24811700	-1.93358400	-0.95813000
Cl	-2.14978600	-0.31466000	-2.95207200
C	-2.01756500	-1.61933500	1.00725900
C	-2.16250200	-0.66835300	2.03838200
C	-1.76415800	-2.96828600	1.33062500
C	-2.05956400	-1.06695900	3.36685200
H	-2.32901600	0.36793300	1.77513200
C	-1.65905800	-3.35464500	2.66159000
H	-1.63083800	-3.68929200	0.53109700
C	-1.81139700	-2.40686600	3.67967800
H	-2.16410000	-0.33343000	4.16019800
H	-1.45561200	-4.39149100	2.90961000
H	-1.72877900	-2.71289900	4.71809100
C	-4.88095400	0.43887700	0.01742000
C	-5.13198000	0.58494300	1.39646100
C	-4.92672300	1.56840900	-0.82304200
C	-5.40173400	1.84488700	1.92343900
H	-5.11129600	-0.29120300	2.03520700
C	-5.20702500	2.82094500	-0.28715200
H	-4.73909100	1.44496000	-1.88419900
C	-5.43707900	2.96295900	1.08533800
H	-5.59614300	1.95486600	2.98581500
H	-5.24805600	3.68952600	-0.93715900
H	-5.65532100	3.94297400	1.49843500
C	-4.09052700	-3.26639100	-1.53118700
H	-5.06324800	-3.65735900	-1.85089400
H	-3.42739800	-3.24069500	-2.40202000
H	-3.67269300	-3.96176800	-0.79499300

Cartesian coordinates of the optimized geometry for **36a** at B3LYP-D3BJ/6-31G(d),def2-TZVP level of theory (number of imaginary frequencies = 1):

C	1.34984900	1.06027000	-0.41873300
C	2.40066900	0.28799600	-0.74553300
C	1.52118800	2.43551500	0.07208100
Cl	2.19998000	-1.45461900	-1.04432800
C	0.77782200	3.49508700	-0.48107900
C	2.39237900	2.71512900	1.14234700
C	2.53406300	4.01366200	1.62586600
C	1.81310000	5.06050700	1.04512000
C	0.93633500	4.79532000	-0.01118700
H	0.36962300	5.60420000	-0.46255400
H	0.08970300	3.28621300	-1.29447900
H	2.94509400	1.89878700	1.59563200
H	3.20532600	4.21122900	2.45680800
Au	-0.59929400	0.34386400	-0.48757600
H	1.92484200	6.07330100	1.41985600
P	-2.83275000	-0.37557500	-0.68112400

C	-2.87456200	-1.85308400	-1.86866500
C	-3.86940800	1.11673300	-1.25384800
C	-1.86491400	-2.86635100	-1.29119600
H	-0.85528400	-2.44869400	-1.23295700
H	-1.83510800	-3.74868500	-1.94151000
H	-2.16116600	-3.19847100	-0.29084400
C	-4.23556000	-2.55366900	-2.00499200
H	-4.13509800	-3.36547400	-2.73537400
H	-5.02386300	-1.88828400	-2.36320200
H	-4.55616200	-3.00107600	-1.06145800
C	-2.38833500	-1.38311900	-3.25091700
H	-1.43681600	-0.84480500	-3.18563000
H	-3.12068200	-0.73837200	-3.74544200
H	-2.23689900	-2.25947900	-3.89203500
C	-3.03003100	1.93066800	-2.26071000
H	-2.09433700	2.27668700	-1.81268900
H	-3.60734700	2.81016600	-2.56966900
H	-2.78305300	1.36096900	-3.15955400
C	-5.21907100	0.78715600	-1.91056100
H	-5.09709600	0.21625600	-2.83485200
H	-5.71707800	1.72824200	-2.17303700
H	-5.89524400	0.24330500	-1.24852700
C	-4.09122600	1.98128900	0.00290600
H	-3.14362900	2.20625000	0.50615500
H	-4.75541700	1.49742000	0.72318200
H	-4.54573000	2.93316800	-0.29564500
C	-3.60003500	-0.97244600	0.88294700
C	-4.96798600	-1.30742400	0.88538100
C	-2.87964900	-1.07961800	2.09496800
C	-5.61882400	-1.73257700	2.03892200
H	-5.53992900	-1.23665100	-0.02889200
C	-3.55301900	-1.51042500	3.24987100
C	-4.90581800	-1.83440700	3.23236500
H	-6.67507200	-1.98072800	2.00249500
H	-2.98887300	-1.58831600	4.17407700
H	-5.39780700	-2.16358800	4.14252800
C	-1.42890400	-0.76703300	2.27132000
C	-1.03499600	0.48023900	2.77925400
C	-0.45465600	-1.75113300	2.05521500
C	0.30719200	0.74022900	3.05615500
H	-1.78793100	1.24190400	2.95867200
C	0.88769400	-1.49063000	2.33288100
H	-0.75344500	-2.72229400	1.67410600
C	1.26942000	-0.24657300	2.83522100
H	0.59969100	1.71352700	3.43699300
H	1.63117500	-2.26142400	2.15566700
H	2.31348300	-0.04800700	3.06040700
C	4.75264200	-0.08108400	-0.57894100
C	3.81927000	0.74772800	-0.92884200
C	5.69960600	-1.03366600	-0.30298300
C	6.05787300	-1.99512800	-1.29834700
C	6.33898000	-1.06576800	0.97419300
C	7.02650700	-2.94068500	-1.01935500
H	5.55008500	-1.96748200	-2.25601300
C	7.30036500	-2.02392100	1.23610200
H	6.05653100	-0.33027100	1.71955400
C	7.64319400	-2.95439300	0.24215400
H	7.30692800	-3.67435800	-1.76751400
H	7.79150400	-2.05779500	2.20265800
H	8.40087900	-3.70287600	0.45454200
C	4.14618900	2.10053600	-1.54950500
H	3.40577200	2.31255700	-2.32419700
H	4.10041800	2.89493200	-0.80343400
H	5.14377700	2.08533900	-1.99417300

Cartesian coordinates of the optimized geometry for **37a** at B3LYP-D3BJ/6-31G(d),def2-TZVP level of theory (number of imaginary frequencies = 1):

C	-1.45080500	-0.90653700	-0.38853600
C	-2.05402100	-0.96487400	-1.58484800
C	-2.24612000	-1.03228300	0.85268200
Cl	-1.16314000	-0.84156400	-3.10934100
C	-2.79703800	-2.27124100	1.23381700
C	-2.40730400	0.06948200	1.71058900
C	-3.12254900	-0.05566100	2.89840900
C	-3.67466900	-1.28764900	3.26507900
C	-3.50479100	-2.39608300	2.42819800
H	-3.91558300	-3.36082400	2.71211000
H	-2.65368700	-3.13343800	0.58926200
H	-1.96083400	1.01720400	1.43218500
H	-3.24055200	0.80662700	3.54854800
Au	0.58271900	-0.63938000	-0.12949700
H	-4.21563900	-1.38850900	4.20128400
P	2.90741200	-0.49206500	0.20735000
C	3.73893000	-1.42546600	-1.22062700
C	3.27954600	-1.20764200	1.93354400
C	3.17728800	-0.78572200	-2.50719400
H	2.08962300	-0.88741100	-2.57266300
H	3.62040900	-1.28574500	-3.37649900
H	3.43261300	0.27754300	-2.56882900
C	5.27287300	-1.34324900	-1.27474400
H	5.62181200	-1.96432700	-2.10838600
H	5.75306400	-1.71622600	-0.36746800
H	5.61789600	-0.32401300	-1.46215200
C	3.30372600	-2.90047000	-1.15512100
H	2.21629200	-3.00186000	-1.07487200
H	3.76565900	-3.42588600	-0.31427900
H	3.62273600	-3.40622200	-2.07394400
C	2.33753700	-2.40517800	2.17775300
H	1.28595000	-2.10926500	2.12000500
H	2.52619600	-2.79949300	3.18337600
H	2.49899100	-3.21798200	1.46605400
C	4.72528000	-1.67144700	2.16792800
H	5.00483200	-2.49502200	1.50535600
H	4.81075200	-2.03923500	3.19748200
H	5.45371000	-0.86616100	2.05566800
C	2.91579800	-0.09299100	2.93451300
H	1.89957400	0.28162400	2.76445100
H	3.60714900	0.75155300	2.87870700
H	2.95615800	-0.50152700	3.95098600
C	3.62792800	1.20344100	0.20162700
C	5.00380700	1.34856200	0.46662200
C	2.85174700	2.37334000	0.02802600
C	5.60929700	2.59704800	0.56399000
H	5.61801900	0.47099700	0.60692500
C	3.47928100	3.62620700	0.13825400
C	4.83948100	3.74736300	0.40186400
H	6.67301300	2.66670500	0.76932100
H	2.87382200	4.51681000	0.00158800
H	5.29309300	4.73088600	0.47721700
C	1.38854300	2.42087100	-0.26760900
C	0.50074600	2.88315000	0.71459600
C	0.89638000	2.16141200	-1.55534700
C	-0.84900700	3.07553100	0.41742100
H	0.87828100	3.10177800	1.70909300
C	-0.45278300	2.35737400	-1.85291500
H	1.57811100	1.82492600	-2.32930500
C	-1.32890800	2.81093300	-0.86676300
H	-1.52037300	3.44825800	1.18644200
H	-0.81396900	2.15307600	-2.85502400
H	-2.37851100	2.96971700	-1.09950000

C	-4.32301800	-0.36981200	-1.11215900
C	-3.52977500	-1.09604600	-1.83000700
C	-5.20024700	0.39405400	-0.38889800
C	-5.58868700	1.68288600	-0.87200800
C	-5.73839600	-0.09896200	0.83947400
C	-6.48513000	2.44184200	-0.14577100
H	-5.16655700	2.04219700	-1.80435400
C	-6.64653300	0.67223900	1.54177500
H	-5.41649900	-1.06812000	1.19915000
C	-7.01462500	1.93420400	1.05360800
H	-6.78663100	3.42208900	-0.49907700
H	-7.06483800	0.30699800	2.47347800
H	-7.72448300	2.53490000	1.61475300
C	-4.06342000	-2.03946300	-2.89476400
H	-3.72044900	-1.70871100	-3.87827500
H	-3.65961200	-3.03945200	-2.70785300
H	-5.15432300	-2.08087600	-2.88691400

Cartesian coordinates of the optimized geometry for **38a** at B3LYP-D3BJ/6-31G(d),def2-TZVP level of theory (number of imaginary frequencies = 0):

C	1.41756400	0.03154300	-0.64255400
C	2.02348100	-0.56198500	-1.68761800
C	2.37623100	0.50535300	0.36942800
Cl	1.16100900	-1.20321200	-3.07280200
C	2.88432600	1.83701300	0.31077900
C	2.67501200	-0.28361700	1.51840400
C	3.47473200	0.22280000	2.53034700
C	3.98577800	1.52493900	2.43272000
C	3.67856900	2.33545800	1.33278600
H	4.06368000	3.34844500	1.28015500
H	2.62773400	2.44598300	-0.55025800
H	2.24913600	-1.27888700	1.57509200
H	3.70426700	-0.38457900	3.39985200
Au	-0.59503800	0.32380800	-0.33445000
H	4.61613900	1.91430500	3.22635000
P	-2.88159100	0.82678100	-0.08716700
C	-3.57170400	1.16644300	-1.82133600
C	-3.02283000	2.30105400	1.11159100
C	-3.21222100	-0.08096400	-2.65496800
H	-2.13079200	-0.24223400	-2.70630200
H	-3.58584000	0.05393800	-3.67679100
H	-3.67996400	-0.98257100	-2.24582200
C	-5.09193000	1.37735200	-1.90476500
H	-5.35139600	1.60814500	-2.94483500
H	-5.44196000	2.20880300	-1.28917800
H	-5.64183000	0.47620700	-1.62444200
C	-2.84671500	2.39268900	-2.40429700
H	-1.75840600	2.29728300	-2.32771500
H	-3.15124600	3.32084500	-1.91195900
H	-3.10258300	2.48508700	-3.46621200
C	-1.84390400	3.26019500	0.84504300
H	-0.88053700	2.76204700	0.98899400
H	-1.90553000	4.09644200	1.55170000
H	-1.86134500	3.67574400	-0.16508100
C	-4.32881300	3.10674300	1.03199600
H	-4.45692300	3.58686700	0.05817500
H	-4.29326900	3.90328300	1.78489000
H	-5.21381600	2.50493100	1.24661700
C	-2.85665900	1.70969000	2.52547700
H	-1.94896100	1.09933200	2.60024500
H	-3.70980400	1.09145900	2.81529300
H	-2.76897800	2.53095000	3.24639100
C	-3.92790700	-0.50851600	0.62856300
C	-5.28905400	-0.24212700	0.87439700

C	-3.40981700	-1.76715700	1.01298300
C	-6.12390600	-1.17406200	1.48214300
H	-5.70891600	0.71243000	0.59152000
C	-4.26632100	-2.69310400	1.63236400
C	-5.60756100	-2.40985400	1.86799500
H	-7.16801100	-0.93203700	1.65462800
H	-3.85767500	-3.65619100	1.92198200
H	-6.24321700	-3.14880700	2.34612800
C	-2.00176900	-2.22789100	0.82275200
C	-1.15120900	-2.33435700	1.93223800
C	-1.55563800	-2.70063900	-0.42068400
C	0.11855000	-2.89881700	1.80009100
H	-1.49771600	-1.98680400	2.90104800
C	-0.28698600	-3.26539500	-0.55177400
H	-2.21412700	-2.64165200	-1.28088500
C	0.55316000	-3.36617900	0.55815300
H	0.75629800	-2.99325800	2.67518400
H	0.04107400	-3.62752100	-1.52016700
H	1.53461400	-3.82104700	0.45714300
C	4.19026800	-0.21060900	-0.67450500
C	3.49293000	-0.69526600	-1.68804700
C	5.43700300	0.01132200	-0.05497200
C	5.96750200	-0.93998800	0.84972600
C	6.18431100	1.17902400	-0.34405400
C	7.22616300	-0.75085100	1.40443700
H	5.38327300	-1.82307200	1.08428900
C	7.44180300	1.35983800	0.21517000
H	5.76598800	1.91524100	-1.02181000
C	7.96228600	0.39626000	1.08706900
H	7.63809500	-1.49157700	2.08210400
H	8.02032500	2.24619900	-0.02412500
H	8.94566300	0.54188500	1.52385300
C	4.22715500	-1.38427800	-2.82020300
H	3.86594000	-2.41253300	-2.92147400
H	4.01944700	-0.86601500	-3.76161500
H	5.30455600	-1.39736500	-2.64747900

Cartesian coordinates of the optimized geometry for **39a** at B3LYP-D3BJ/6-31G(d),def2-TZVP level of theory (number of imaginary frequencies = 1):

C	1.41080600	-0.06357100	0.62198200
C	2.04097900	0.49945400	1.67103600
C	2.35387700	-0.43392700	-0.44089600
Cl	1.21581400	1.10701300	3.09056300
C	2.69066000	-1.77995800	-0.76437900
C	2.87864700	0.61597900	-1.26097400
C	3.61267400	0.31226600	-2.42692400
C	3.93846600	-1.00092400	-2.69966900
C	3.48972000	-2.04807900	-1.85465600
H	3.75231900	-3.07359700	-2.09427200
H	2.29571700	-2.57678900	-0.14310100
H	2.54537400	1.62693900	-1.05833300
H	3.93499600	1.11186100	-3.08519900
Au	-0.60322000	-0.33590000	0.31423800
H	4.52864200	-1.24445900	-3.57746200
P	-2.89031500	-0.83994700	0.07604200
C	-3.57011000	-1.21593200	1.80598400
C	-3.03869900	-2.28892400	-1.15232700
C	-3.20674000	0.01335900	2.66433900
H	-2.12526900	0.17586100	2.71188700
H	-3.57288600	-0.14501600	3.68544700
H	-3.67896500	0.92281000	2.27863300
C	-5.09034800	-1.42684100	1.88872600
H	-5.34840800	-1.67418700	2.92528800
H	-5.44204200	-2.24773200	1.26022800

H	-5.63969200	-0.52072700	1.62358300
C	-2.84306000	-2.45442500	2.35965500
H	-1.75494100	-2.35583400	2.28391100
H	-3.14699800	-3.37183200	1.84720100
H	-3.09700800	-2.57115200	3.41958900
C	-1.85153800	-3.24682100	-0.91937900
H	-0.89310300	-2.74180500	-1.07254700
H	-1.92128200	-4.07290800	-1.63710200
H	-1.85007700	-3.67692000	0.08481300
C	-4.33915800	-3.10380500	-1.07257300
H	-4.45125300	-3.60604700	-0.10803500
H	-4.30950800	-3.88317700	-1.84342900
H	-5.23066300	-2.50299500	-1.26146200
C	-2.89297700	-1.66863600	-2.55595300
H	-1.99092500	-1.04969700	-2.62871900
H	-3.75370300	-1.05131400	-2.82420600
H	-2.80709400	-2.47479300	-3.29389300
C	-3.93654300	0.51360400	-0.60261700
C	-5.29653400	0.25344900	-0.86076600
C	-3.41915400	1.78543400	-0.94087400
C	-6.13088000	1.20491000	-1.43811900
H	-5.71562400	-0.71086700	-0.61126000
C	-4.27514800	2.73153100	-1.52943700
C	-5.61522800	2.45426800	-1.77889100
H	-7.17416700	0.96783400	-1.62193500
H	-3.86733900	3.70480100	-1.78405400
H	-6.25075800	3.20845000	-2.23275900
C	-2.01250100	2.24145400	-0.72731300
C	-1.14453400	2.35768900	-1.82270600
C	-1.58584900	2.70190000	0.52745900
C	0.12209000	2.92245600	-1.66604000
H	-1.47534800	2.01782100	-2.79965300
C	-0.32035000	3.26741700	0.68331700
H	-2.25710400	2.63299800	1.37696900
C	0.53473300	3.38255300	-0.41382500
H	0.77517600	3.02045700	-2.52906500
H	-0.00724600	3.62176400	1.65958800
H	1.51047200	3.84595200	-0.29392300
C	4.13834000	0.31829500	0.48533800
C	3.50839100	0.61271300	1.62441000
C	5.44315800	-0.01687500	0.00489400
C	6.22251700	0.91476800	-0.71319900
C	5.97042800	-1.29765200	0.27022100
C	7.50714000	0.58352600	-1.12108300
H	5.81132600	1.89627000	-0.92637900
C	7.25890600	-1.62506900	-0.14460700
H	5.36331500	-2.01434400	0.81266400
C	8.02519300	-0.68724600	-0.83908700
H	8.11083100	1.30978400	-1.65594700
H	7.66460100	-2.60787000	0.07326400
H	9.02932700	-0.94344100	-1.16303300
C	4.30622800	1.00979400	2.84289300
H	4.02615400	2.02171200	3.15450700
H	4.07319000	0.33751800	3.67516100
H	5.37858400	0.98194000	2.64429000

Cartesian coordinates of the optimized geometry for **40a** at B3LYP-D3BJ/6-31G(d),def2-TZVP level of theory (number of imaginary frequencies = 0):

C	1.31741800	-0.61075100	-0.34843900
C	2.06358600	-1.26341500	0.63044300
C	2.05941400	0.18090800	-1.24750600
Cl	1.19580600	-2.29753200	1.74079900
C	1.46020400	0.77638000	-2.39078800
C	3.49373300	0.50409200	-0.98446000

C	4.27303500	1.05709100	-2.13235700
C	3.63645000	1.52308000	-3.23265800
C	2.21133700	1.41190900	-3.34646200
H	1.72294900	1.81783500	-4.22738900
H	0.38978200	0.65263600	-2.51583800
H	3.33532000	1.44211100	-0.37993900
H	5.34662900	1.14596900	-2.01639300
Au	-0.76084200	-0.60461700	-0.34750800
H	4.20187300	1.97119700	-4.04331100
P	-3.09945000	-0.48822900	-0.24968400
C	-3.74497400	-1.90326800	0.83354700
C	-3.76813200	-0.50980400	-2.03408500
C	-2.98137500	-1.78137500	2.16773600
H	-1.89884700	-1.85620200	2.02765100
H	-3.29708300	-2.59290300	2.83372900
H	-3.20421400	-0.83396000	2.66922900
C	-5.25095200	-1.86708600	1.13831300
H	-5.50228400	-2.74688000	1.74253100
H	-5.86972600	-1.90148600	0.23941600
H	-5.52492700	-0.98354200	1.71953200
C	-3.37830600	-3.23730400	0.15918300
H	-2.31705700	-3.28132200	-0.10792200
H	-3.97173800	-3.41801600	-0.74157400
H	-3.58430000	-4.05718600	0.85702600
C	-2.90701600	-1.49457100	-2.85315800
H	-1.85114500	-1.20833900	-2.84929900
H	-3.25929600	-1.48995600	-3.89143600
H	-2.97644300	-2.51987300	-2.48299200
C	-5.24256500	-0.91012200	-2.20027700
H	-5.42915500	-1.93527100	-1.87011400
H	-5.49772000	-0.86101100	-3.26560500
H	-5.92976800	-0.24235300	-1.67748900
C	-3.54529300	0.91074000	-2.58904500
H	-2.50742200	1.23686300	-2.45143800
H	-4.19839600	1.64632100	-2.11331600
H	-3.75713900	0.90980700	-3.66457600
C	-3.71804200	1.06696600	0.52201100
C	-5.10475500	1.31260200	0.50464300
C	-2.86835000	2.05553600	1.07433900
C	-5.65304000	2.49107900	0.99986100
H	-5.77366400	0.57099900	0.09167400
C	-3.44024400	3.24299700	1.56281000
C	-4.81250100	3.46818000	1.52956400
H	-6.72743700	2.64266400	0.96734700
H	-2.78039400	3.99436300	1.98527800
H	-5.22018600	4.39634000	1.91804300
C	-1.38064800	1.97376700	1.21397800
C	-0.55860600	2.75801200	0.39048000
C	-0.79173100	1.25832100	2.26795000
C	0.81796800	2.82273800	0.61256300
H	-1.00875800	3.32977400	-0.41562600
C	0.58408000	1.32111800	2.48902200
H	-1.41873200	0.66605400	2.92538800
C	1.39208200	2.10731200	1.66512900
H	1.43405700	3.44928900	-0.02709200
H	1.02047900	0.76520900	3.31317400
H	2.45965300	2.17610400	1.86054600
C	4.20273100	-0.31918000	0.04514200
C	3.48646300	-1.16977100	0.85037200
C	5.65771900	-0.08535800	0.20221500
C	6.13922100	1.16874800	0.60929400
C	6.57337000	-1.10371500	-0.10254800
C	7.50983500	1.39497600	0.72182800
H	5.43908200	1.96268800	0.85793100
C	7.94431400	-0.87144900	0.00479200
H	6.20715200	-2.07207200	-0.42989000

C	8.41497800	0.37593200	0.41772300
H	7.87009900	2.36443300	1.05188700
H	8.64350900	-1.66639200	-0.23533000
H	9.48229600	0.55345800	0.50433500
C	4.13384500	-1.95472700	1.96277500
H	3.65410900	-1.72923800	2.92055300
H	4.02194000	-3.03166400	1.79846600
H	5.19493000	-1.72376100	2.04406800

Cartesian coordinates of the optimized geometry for **41a** at B3LYP-D3BJ/6-31G(d),def2-TZVP level of theory (number of imaginary frequencies = 1):

C	1.15183900	-0.91825900	-1.19313700
C	2.34358500	-0.50216700	-0.70069800
Au	-0.70154900	-0.15353300	-0.71956600
P	-2.81157600	0.83734200	-0.36559000
C	-2.52829600	2.53087000	0.44366400
C	-3.68211000	0.92232700	-2.05806900
C	-1.68928300	2.24033100	1.70506200
H	-0.71893400	1.80557300	1.45172200
H	-1.51364000	3.18084200	2.24118500
H	-2.20823500	1.55725900	2.38590500
C	-3.79586400	3.28655800	0.87274000
H	-3.49595100	4.25740500	1.28529000
H	-4.47791000	3.48173100	0.04280300
H	-4.34022300	2.75308600	1.65501700
C	-1.70562700	3.40198100	-0.52215000
H	-0.81425700	2.87769400	-0.88289600
H	-2.29365000	3.72180400	-1.38708700
H	-1.37748900	4.30607900	0.00438200
C	-2.61708400	1.21734300	-3.13555900
H	-1.84572300	0.44221700	-3.16533400
H	-3.10834600	1.24508300	-4.11534900
H	-2.12314800	2.18000600	-2.98353100
C	-4.79871900	1.97048100	-2.18765700
H	-4.42253800	2.98931900	-2.06313600
H	-5.22263800	1.90201600	-3.19656900
H	-5.62019200	1.81180900	-1.48657400
C	-4.25234400	-0.48695500	-2.31118100
H	-3.47628400	-1.25504500	-2.21664200
H	-5.06564100	-0.73261500	-1.62365900
H	-4.64302000	-0.53431700	-3.33420500
C	-3.94941600	-0.04830500	0.78242000
C	-5.25640900	0.44775400	0.95248000
C	-3.55525500	-1.17387400	1.54330000
C	-6.15065700	-0.12427400	1.85149800
H	-5.58366600	1.30423600	0.38056400
C	-4.46446900	-1.72604600	2.46119200
C	-5.74801900	-1.21440700	2.62107200
H	-7.15129900	0.28459800	1.95157800
H	-4.14877500	-2.58797600	3.04074200
H	-6.42975100	-1.66924200	3.33317200
C	-2.23991300	-1.87175000	1.43982100
C	-1.98641100	-2.75858700	0.38208700
C	-1.29409600	-1.75522800	2.46690500
C	-0.80571300	-3.50122700	0.34510300
H	-2.72864400	-2.87605500	-0.40152600
C	-0.11052700	-2.49565500	2.42725100
H	-1.49547800	-1.08799800	3.30028200
C	0.13801800	-3.36598700	1.36438700
H	-0.62298000	-4.17888600	-0.48222900
H	0.61066100	-2.40236500	3.23479400
H	1.05560500	-3.94593700	1.33665500
C	4.66321000	-0.18321900	-0.34269900
C	3.73678400	-0.93073600	-0.92840400

Cl	1.12479400	-2.18774700	-2.43019900
C	2.40311200	0.57822200	0.31904300
C	1.73768100	0.51901000	1.55102600
C	3.39523500	1.57319300	0.11432100
C	2.07438200	1.41503200	2.55902600
H	1.00690100	-0.26269000	1.72081500
C	3.73295100	2.47018700	1.15619200
H	3.69470100	1.81799200	-0.90079600
C	3.08264300	2.38407800	2.37221800
H	1.57386800	1.34578300	3.52064500
H	4.48088800	3.23725500	0.98104500
H	3.33090700	3.06670400	3.17857700
C	5.99272200	0.13146000	-0.00246000
C	6.79016900	0.93593800	-0.85461800
C	6.54354800	-0.36834800	1.20331100
C	8.10616500	1.21019300	-0.51535700
H	6.36051100	1.32052400	-1.77408300
C	7.86451700	-0.09282900	1.52853600
H	5.92000500	-0.97074400	1.85524100
C	8.64237000	0.69678400	0.67366300
H	8.72213400	1.81671900	-1.17125500
H	8.29232200	-0.48566900	2.44509100
H	9.67357400	0.91483300	0.93438400
C	4.19453600	-2.15849700	-1.68687500
H	5.27196500	-2.30305100	-1.57615500
H	3.67357600	-3.04338500	-1.31226300
H	3.95577100	-2.05623100	-2.74840100

Cartesian coordinates of the optimized geometry for **42a** at B3LYP-D3BJ/6-31G(d),def2-TZVP level of theory (number of imaginary frequencies = 0):

C	1.16023100	1.11323500	-0.55026700
C	2.38279100	0.59578600	-0.12811200
Au	-0.77909300	0.70540500	0.00397000
P	-3.04453700	0.46970400	0.61593000
C	-3.13189700	0.02068100	2.45828300
C	-3.91090400	2.10592000	0.17281800
C	-2.23014600	-1.22002100	2.62302300
H	-1.18200900	-0.98000800	2.42623200
H	-2.30557400	-1.58180200	3.65513000
H	-2.53452900	-2.03553100	1.95804400
C	-4.52895300	-0.33070800	2.99585300
H	-4.44784900	-0.50112800	4.07590300
H	-5.25794600	0.46807300	2.84642500
H	-4.91664100	-1.24836200	2.54850700
C	-2.54296300	1.18615800	3.27311400
H	-1.56834300	1.50390200	2.88723300
H	-3.20963500	2.05274800	3.28827700
H	-2.40500800	0.86071700	4.31081400
C	-2.95542500	3.27286700	0.49885500
H	-2.01681500	3.19558300	-0.05812700
H	-3.44118800	4.21280500	0.21221800
H	-2.71472400	3.33472700	1.56239900
C	-5.24912000	2.35629100	0.88555100
H	-5.13067600	2.43780100	1.96915900
H	-5.65680500	3.31031300	0.53119600
H	-5.99658400	1.59056800	0.66871200
C	-4.13040700	2.06812500	-1.35224500
H	-3.19086200	1.89225700	-1.88785400
H	-4.84648700	1.29668800	-1.64688100
H	-4.52080600	3.03899600	-1.67798500
C	-3.94466200	-0.87293100	-0.26683000
C	-5.31635800	-1.04934800	0.00027100
C	-3.31793700	-1.75781400	-1.17640000
C	-6.05295100	-2.07641800	-0.58083500

H	-5.82228500	-0.37876900	0.67985900
C	-4.07316000	-2.80098800	-1.73749300
C	-5.42342500	-2.96825000	-1.44734600
H	-7.10922500	-2.17872300	-0.35255600
H	-3.58088700	-3.47534000	-2.43123600
H	-5.98066400	-3.78046100	-1.90381400
C	-1.90386000	-1.66296200	-1.64784600
C	-1.55587500	-0.75384800	-2.66029200
C	-0.93804000	-2.57154400	-1.19427000
C	-0.26596300	-0.74283800	-3.19192500
H	-2.30820300	-0.06990200	-3.04039500
C	0.35435400	-2.55591500	-1.72315700
H	-1.20867100	-3.29493000	-0.42994400
C	0.69433600	-1.64000800	-2.71940300
H	-0.01294500	-0.03393600	-3.97392600
H	1.09277600	-3.26505000	-1.36121700
H	1.69744300	-1.63304200	-3.13487000
C	4.64686100	0.13603200	0.21926000
C	3.79184600	0.95247300	-0.46111600
Cl	1.19178200	2.34820700	-1.79140400
C	2.42969800	-0.51910300	0.76930800
C	1.46137800	-1.43515400	1.20726800
C	3.84904500	-0.74024400	1.15690800
C	1.86965500	-2.61942100	1.79152600
H	0.41542800	-1.26843100	0.98683600
C	4.22644200	-2.10642900	1.59484800
H	3.90083500	-0.19442900	2.13462500
C	3.24769400	-2.97841500	1.95239200
H	1.11155900	-3.33654100	2.09458300
H	5.28002600	-2.33462700	1.71956900
H	3.49447500	-3.95782500	2.34894600
C	6.11383100	0.10310200	0.20597300
C	6.84073000	0.01662100	1.40734000
C	6.82264500	0.11234100	-1.00799800
C	8.23249500	-0.03796500	1.39534600
H	6.31653900	0.02223600	2.35974600
C	8.21479500	0.05611300	-1.01758200
H	6.27475100	0.13851100	-1.94435000
C	8.92420300	-0.01738200	0.18245000
H	8.77737000	-0.09069300	2.33302800
H	8.74567900	0.06112500	-1.96467900
H	10.00877500	-0.06206700	0.17310900
C	4.23447600	2.09514800	-1.32852000
H	5.31477000	2.22861800	-1.24672500
H	3.98635500	1.93674600	-2.38290100
H	3.75190000	3.02796400	-1.02425300

Cartesian coordinates of the optimized geometry for **43a** at B3LYP-D3BJ/6-31G(d),def2-TZVP level of theory (number of imaginary frequencies = 1):

C	1.53056500	0.52442900	-0.98257100
C	2.26512400	1.44389600	-0.31643000
Au	-0.49496200	0.24216800	-0.74532100
P	-2.80413100	-0.02683700	-0.41958100
C	-3.47278500	1.52057000	0.44985900
C	-3.59700100	-0.36269100	-2.12004000
C	-2.61488500	1.68410400	1.72085000
H	-1.55098800	1.78535100	1.49183900
H	-2.93326800	2.58975400	2.25064000
H	-2.74936300	0.83597000	2.39954800
C	-4.94820500	1.44313700	0.87286200
H	-5.22248100	2.39226000	1.34887300
H	-5.62613400	1.29223800	0.03047100
H	-5.11725400	0.64969100	1.60460300
C	-3.25562600	2.73317900	-0.47202000

H	-2.22087100	2.79468500	-0.82225200
H	-3.91800100	2.71134800	-1.34207500
H	-3.47792100	3.64979100	0.08720400
C	-2.85075000	0.49204400	-3.16695600
H	-1.78577600	0.24544400	-3.20467400
H	-3.28232100	0.29203300	-4.15485400
H	-2.94247300	1.56380100	-2.97470300
C	-5.09914000	-0.06065500	-2.24108400
H	-5.31793800	0.99893400	-2.08545400
H	-5.42210100	-0.31308600	-3.25813000
H	-5.71402800	-0.64868900	-1.55725800
C	-3.33021400	-1.84912100	-2.42806600
H	-2.26755000	-2.09577600	-2.32028900
H	-3.90403600	-2.51454500	-1.77848400
H	-3.61652800	-2.05361200	-3.46626300
C	-3.26457300	-1.43518300	0.67502100
C	-4.62367100	-1.78591900	0.78831400
C	-2.31185300	-2.19986300	1.38783000
C	-5.04555100	-2.85819400	1.56737400
H	-5.37084700	-1.21239100	0.25805400
C	-2.75601700	-3.28010100	2.16857700
C	-4.10313100	-3.61428400	2.26226500
H	-6.10234400	-3.09911700	1.62790300
H	-2.01557500	-3.85988900	2.71089900
H	-4.41317300	-4.45592600	2.87406700
C	-0.83478200	-1.96608800	1.40097500
C	0.00771500	-2.74081300	0.58975200
C	-0.25991700	-1.08069500	2.32347500
C	1.39395100	-2.62036900	0.68806500
H	-0.43125700	-3.43880200	-0.11668900
C	1.12621900	-0.96318800	2.42315700
H	-0.90192800	-0.49173000	2.96947900
C	1.95536200	-1.73066500	1.60478700
H	2.03198700	-3.21865500	0.04540300
H	1.55166800	-0.27428000	3.14583300
H	3.03541000	-1.64487700	1.68578600
C	4.52148200	0.55595800	-0.35254600
C	3.75927900	1.59752300	-0.39710100
Cl	2.36309700	-0.62306400	-2.05578600
C	1.59928300	2.42328900	0.58947900
C	1.92790300	2.46777200	1.95176900
C	0.64013300	3.31834600	0.09279500
C	1.28967700	3.36522400	2.80714100
H	2.67740500	1.78406300	2.34053900
C	0.00114900	4.21490300	0.94879300
H	0.40231000	3.30219600	-0.96562100
C	0.32072100	4.23837300	2.30763300
H	1.54570300	3.38173500	3.86241100
H	-0.74405600	4.89789500	0.55217100
H	-0.17611700	4.93796800	2.97288000
C	5.36686700	-0.52126600	-0.23896200
C	5.85000500	-0.90568800	1.04841400
C	5.77724500	-1.24816100	-1.39653100
C	6.71504900	-1.97870700	1.16491400
H	5.52978900	-0.34145700	1.91774100
C	6.65270000	-2.30918200	-1.26205500
H	5.37888800	-0.95746600	-2.36168200
C	7.11472200	-2.67378900	0.01314000
H	7.08680900	-2.28078000	2.13805400
H	6.97522200	-2.86676600	-2.13475200
H	7.79417000	-3.51545600	0.11061000
C	4.35008200	2.99642900	-0.50112400
H	5.44030600	2.98087700	-0.55670200
H	3.94720600	3.47262800	-1.40065900
H	4.03593100	3.58074100	0.36741000

Cartesian coordinates of the optimized geometry for **44a** at B3LYP-D3BJ/6-31G(d),def2-TZVP level of theory (number of imaginary frequencies = 1):

C	1.25882000	0.65946100	0.78103600
C	2.51213100	0.99477600	0.71605400
Au	-0.59837500	0.08914300	0.63042700
P	-2.69715300	-0.99836100	0.42793700
C	-2.35315200	-2.67819700	-0.38262200
C	-3.50065300	-1.09019000	2.14730200
C	-1.55841200	-2.36115700	-1.66654400
H	-0.59458200	-1.89197200	-1.44701500
H	-1.36327000	-3.29773000	-2.20135100
H	-2.12033700	-1.70354300	-2.33747000
C	-3.61146800	-3.46927700	-0.77468900
H	-3.29449400	-4.42767500	-1.20208300
H	-4.25970900	-3.68975300	0.07574500
H	-4.19577300	-2.94893300	-1.53668500
C	-1.48030800	-3.51836500	0.56609000
H	-0.58953300	-2.97264000	0.89515800
H	-2.03322800	-3.84838700	1.45005100
H	-1.14469200	-4.41621100	0.03469600
C	-2.38930300	-1.33954700	3.18828100
H	-1.65069600	-0.53215100	3.19360900
H	-2.84538000	-1.38443600	4.18390400
H	-1.86124600	-2.28134300	3.02191300
C	-4.57425200	-2.17808700	2.31029900
H	-4.16293200	-3.18317800	2.18702200
H	-4.97950400	-2.11462000	3.32684800
H	-5.41429100	-2.05610800	1.62372400
C	-4.11422700	0.29943600	2.40660800
H	-3.37201800	1.09660000	2.28180800
H	-4.95706800	0.50892300	1.74342500
H	-4.47375600	0.34073300	3.44100200
C	-3.86123900	-0.13461800	-0.69921300
C	-5.16527200	-0.64599100	-0.84182900
C	-3.48605900	0.99143800	-1.46838700
C	-6.07828100	-0.08222900	-1.72704500
H	-5.47427100	-1.50410400	-0.26098700
C	-4.41549100	1.53486200	-2.36988100
C	-5.69726400	1.01032900	-2.50476400
H	-7.07681100	-0.49938000	-1.81093000
H	-4.11752600	2.39641300	-2.95905700
H	-6.39509100	1.45691400	-3.20620500
C	-2.17115300	1.69472900	-1.38389400
C	-1.93894300	2.63678000	-0.36552300
C	-1.20200200	1.52563200	-2.38284500
C	-0.76414100	3.39562100	-0.35631500
H	-2.69909700	2.79622500	0.39369800
C	-0.02558100	2.27877000	-2.36462300
H	-1.37680400	0.80565600	-3.17658400
C	0.19225800	3.22084400	-1.35826600
H	-0.60123000	4.12814400	0.42768600
H	0.71679000	2.13259100	-3.14327000
H	1.10156700	3.81206600	-1.34862000
C	3.58810600	-0.04094400	0.67400800
C	3.63930100	-1.01283900	-0.26335900
C	4.75917600	-1.95645400	-0.45683100
C	4.52331900	-3.33598500	-0.56635500
C	6.07484100	-1.47644600	-0.55256700
C	5.58679400	-4.21767200	-0.73724000
H	3.50683600	-3.71182400	-0.50991300
C	7.13585000	-2.36165400	-0.73295000
H	6.25983600	-0.40799700	-0.50543000
C	6.89469600	-3.73349100	-0.82060000
H	5.39538600	-5.28386900	-0.80919600

H	8.14838800	-1.97859300	-0.81388300
H	7.72147700	-4.42285400	-0.96146100
Cl	2.33424200	-1.22113000	-1.44042700
C	2.83250400	2.45267700	0.67864900
C	2.22327600	3.34996200	1.56486000
C	3.70773000	2.91927200	-0.31192600
C	2.48215800	4.71588000	1.45083700
H	1.56659300	2.97338700	2.34230200
C	3.96806100	4.28362600	-0.41262700
H	4.16772400	2.21412200	-0.99758400
C	3.34949400	5.18377600	0.46215700
H	2.01527700	5.41230600	2.14051900
H	4.65101000	4.64694000	-1.17433800
H	3.55361000	6.24662800	0.37807300
C	4.61010000	0.11597000	1.77588100
H	5.20800000	1.02339400	1.63349200
H	4.09818400	0.21622400	2.73984600
H	5.28095400	-0.74182600	1.82500600

Cartesian coordinates of the optimized geometry for **45a** at B3LYP-D3BJ/6-31G(d),def2-TZVP level of theory (number of imaginary frequencies = 0):

C	1.53902400	0.81461200	0.72660000
C	2.81422600	0.97689500	0.59805300
Au	-0.36459600	0.26983000	0.65528100
P	-2.54235900	-0.59992500	0.46567200
C	-2.40512000	-2.19985900	-0.54518400
C	-3.25607900	-0.82842400	2.21369300
C	-1.68589700	-1.80405400	-1.85117100
H	-0.68965500	-1.39076600	-1.66552900
H	-1.57646500	-2.69594000	-2.47971700
H	-2.26088100	-1.06594600	-2.41883800
C	-3.75227100	-2.84202900	-0.91231900
H	-3.55534800	-3.76015300	-1.47831700
H	-4.34436200	-3.11785700	-0.03782800
H	-4.35143800	-2.18746100	-1.54929300
C	-1.53655400	-3.20574200	0.23077300
H	-0.57663900	-2.77251400	0.53190700
H	-2.04194700	-3.58351500	1.12400000
H	-1.32666600	-4.06662300	-0.41490000
C	-2.10681500	-1.27062100	3.14428800
H	-1.31035200	-0.52169800	3.18729300
H	-2.50539700	-1.39819000	4.15735200
H	-1.66245400	-2.22132600	2.83968400
C	-4.39736900	-1.85052000	2.33848100
H	-4.06966800	-2.86366400	2.09139300
H	-4.73651400	-1.86391800	3.38083900
H	-5.26551000	-1.60401000	1.72428000
C	-3.74105700	0.56406800	2.66209200
H	-2.95165200	1.31781000	2.55867600
H	-4.61186200	0.90056300	2.09450000
H	-4.01932300	0.51806500	3.72109900
C	-3.69166700	0.48101600	-0.47692800
C	-5.05027300	0.11706200	-0.54323300
C	-3.27020900	1.65031800	-1.15331700
C	-5.97690600	0.87149100	-1.25526700
H	-5.39434600	-0.77259500	-0.03393400
C	-4.21871000	2.39323700	-1.87444800
C	-5.55719100	2.01681500	-1.93006900
H	-7.01725500	0.56311800	-1.28284600
H	-3.88544800	3.28742100	-2.39178400
H	-6.26635300	2.61541000	-2.49331100
C	-1.87593600	2.18994600	-1.16402100
C	-1.45420700	3.08217900	-0.16553200
C	-1.00995500	1.90752400	-2.23010800

C	-0.18541000	3.66666600	-0.22756900
H	-2.13023200	3.32801500	0.64817500
C	0.25722500	2.48963700	-2.28805300
H	-1.33586200	1.23290300	-3.01567700
C	0.67355400	3.36958000	-1.28789100
H	0.12060800	4.36439000	0.54670900
H	0.91731100	2.26107100	-3.11898800
H	1.65922700	3.82137700	-1.33502800
C	3.02940100	-0.53325800	0.60510700
C	2.84374300	-1.27393700	-0.53278400
C	3.09327700	-2.71761200	-0.65969700
C	2.14495600	-3.54164800	-1.29304600
C	4.27385400	-3.29128200	-0.15731600
C	2.35884000	-4.91266200	-1.38421300
H	1.23970800	-3.10322100	-1.69844400
C	4.49301400	-4.66186100	-0.27393500
H	5.03205500	-2.66006800	0.29286400
C	3.53342800	-5.47603300	-0.87778700
H	1.61397600	-5.54291600	-1.86007200
H	5.41608700	-5.09180400	0.10163600
H	3.70390300	-6.54473100	-0.96268300
Cl	2.26824000	-0.51743700	-2.00236000
C	3.84478100	2.00403300	0.54904300
C	3.53508600	3.32968600	0.89696300
C	5.14273300	1.67453200	0.13749900
C	4.51702800	4.31160900	0.82211800
H	2.53178100	3.57392700	1.23121300
C	6.12154600	2.66338700	0.06528600
H	5.37470500	0.65000500	-0.13781900
C	5.81097300	3.98115100	0.40603200
H	4.27777800	5.33502500	1.09414400
H	7.12525800	2.40599700	-0.25798500
H	6.57524600	4.75007300	0.35134100
C	3.46723700	-1.12074300	1.92634900
H	4.53133600	-0.92641000	2.10677900
H	2.90987200	-0.64322700	2.73618100
H	3.30134300	-2.19886800	1.96987400

Cartesian coordinates of the optimized geometry for **46a** at B3LYP-D3BJ/6-31G(d),def2-TZVP level of theory (number of imaginary frequencies = 1):

C	1.37283100	0.36262900	0.63114100
C	2.33777500	1.18520300	0.59683700
Au	-0.57856200	-0.18793300	0.47887700
P	-2.81643800	-0.87239300	0.32030400
C	-2.88368200	-2.42501300	-0.76478400
C	-3.46737100	-1.12681200	2.09222300
C	-2.16789000	-2.04375600	-2.07678400
H	-1.12663100	-1.75166800	-1.90666100
H	-2.17487800	-2.90903900	-2.74982400
H	-2.67908600	-1.22062800	-2.58600700
C	-4.29896300	-2.91296300	-1.11479300
H	-4.21032800	-3.82302900	-1.71980000
H	-4.89411200	-3.16352400	-0.23429900
H	-4.84450200	-2.17696400	-1.70959400
C	-2.10129500	-3.55016300	-0.06516700
H	-1.09857800	-3.22527000	0.23300800
H	-2.62279900	-3.92376200	0.82056800
H	-1.99034100	-4.39102600	-0.75959800
C	-2.33368100	-1.74054200	2.94051100
H	-1.45642500	-1.08755200	2.97291300
H	-2.69619600	-1.87289400	3.96658300
H	-2.01497500	-2.71820700	2.57164200
C	-4.70770200	-2.02491000	2.22331000
H	-4.51060000	-3.04893400	1.89560200

H	-4.99108600	-2.07124700	3.28141200
H	-5.57277500	-1.64224300	1.67878200
C	-3.77644900	0.28134100	2.63742500
H	-2.91649400	0.95232400	2.52731200
H	-4.63350100	0.73815600	2.13689000
H	-4.00523400	0.20493000	3.70660800
C	-3.92085200	0.35953400	-0.48190800
C	-5.30587300	0.10382300	-0.50480900
C	-3.44891800	1.57089700	-1.03895100
C	-6.21069200	1.00757700	-1.05137800
H	-5.68944200	-0.81675500	-0.08796400
C	-4.37862600	2.47220700	-1.58390300
C	-5.74356100	2.20373100	-1.59371200
H	-7.27144900	0.77711800	-1.05035900
H	-4.00612100	3.39884500	-2.00918400
H	-6.43529100	2.92197400	-2.02294100
C	-2.01931400	2.00225800	-1.11461300
C	-1.51233500	2.92483700	-0.18670000
C	-1.21264500	1.61565600	-2.19563200
C	-0.22381000	3.44171900	-0.33344600
H	-2.13901000	3.24252300	0.64147200
C	0.07350000	2.13608300	-2.34186900
H	-1.60513700	0.91976800	-2.93008600
C	0.56987000	3.05209000	-1.41342800
H	0.15553200	4.15815400	0.38918100
H	0.68341800	1.83318300	-3.18746500
H	1.56829400	3.46116500	-1.53081300
C	2.70899000	-0.50360400	0.73103300
C	3.22893300	-1.06274100	-0.41825200
C	4.36079800	-1.98555200	-0.48248600
C	4.36222300	-3.08477600	-1.36229300
C	5.49083700	-1.75152500	0.32612500
C	5.45144400	-3.94735400	-1.40134900
H	3.50230500	-3.26446000	-1.99788700
C	6.58668200	-2.60631200	0.26442300
H	5.52081100	-0.87933600	0.96958900
C	6.56572300	-3.71035800	-0.59088300
H	5.43616100	-4.80262600	-2.06926500
H	7.45928300	-2.40728400	0.87834100
H	7.41908000	-4.38003600	-0.63298200
Cl	2.48744500	-0.68186800	-1.95224500
C	3.26558900	2.27006700	0.65554700
C	3.00600700	3.33576000	1.53871100
C	4.40378200	2.30128000	-0.17099300
C	3.86522500	4.43020300	1.56935400
H	2.13190000	3.29439400	2.17980500
C	5.26250300	3.39353500	-0.12000000
H	4.60184000	1.47266000	-0.84282600
C	4.99279100	4.45942100	0.74464900
H	3.66194900	5.25569500	2.24410800
H	6.14161800	3.41816400	-0.75600400
H	5.66525000	5.31091700	0.77842700
C	3.09980600	-0.94912300	2.12388000
H	3.77966200	-0.23428400	2.60100400
H	2.19596500	-1.00071700	2.73910800
H	3.57844400	-1.93021500	2.12131000

Cartesian coordinates of the optimized geometry for **25a** at B3LYP-D3BJ/6-31G(d),def2-TZVP level of theory (number of imaginary frequencies = 0):

C	-2.25438700	0.40554600	0.27458200
C	-3.03269800	-0.04962900	1.36505900
C	-2.84423900	1.10212500	-0.85774500
Cl	-2.10275200	-0.44059100	2.84434000
C	-3.77115200	2.15533100	-0.69383000

C	-2.40867700	0.77681200	-2.16319200
C	-2.94848500	1.42423100	-3.26693100
C	-3.86868500	2.46333700	-3.08932300
C	-4.25688600	2.84308500	-1.80129500
H	-4.94065700	3.67444500	-1.66180800
H	-4.05289400	2.47497200	0.30299700
H	-1.67379000	-0.01184700	-2.28744900
H	-2.63477500	1.14094000	-4.26673400
Au	-0.20304600	0.28069400	0.27356600
H	-4.26653900	2.98903100	-3.95178800
P	2.12524700	0.67647800	0.36369000
C	2.64142400	0.75000700	2.18573000
C	2.42141400	2.29410300	-0.60181000
C	2.16628200	-0.57947700	2.80583400
H	1.08297100	-0.70765300	2.71943500
H	2.42574400	-0.58704500	3.87080800
H	2.66052000	-1.43745900	2.33837800
C	4.15242000	0.88318100	2.43508800
H	4.31799800	0.95690100	3.51636200
H	4.58363600	1.77594100	1.97766100
H	4.69967600	0.00648300	2.08122100
C	1.89696900	1.91749000	2.85826600
H	0.81854500	1.87670800	2.67177300
H	2.27257300	2.88930100	2.52592600
H	2.05217600	1.85918300	3.94170700
C	1.25578000	3.26407900	-0.31502800
H	0.29568800	2.84775200	-0.63350400
H	1.42634200	4.18938000	-0.87766200
H	1.17712400	3.52807500	0.74186000
C	3.74069200	3.01728800	-0.28893600
H	3.78439000	3.36195800	0.74755200
H	3.81051000	3.90448200	-0.92930600
H	4.62121000	2.40591900	-0.49450800
C	2.37457900	1.90901000	-2.09372700
H	1.45884900	1.35735600	-2.33718900
H	3.23160700	1.29935000	-2.38963500
H	2.38231700	2.82492900	-2.69571300
C	3.18056400	-0.59315100	-0.44476200
C	4.56628200	-0.36118500	-0.54035700
C	2.64467700	-1.74199800	-1.07224300
C	5.40785400	-1.21951900	-1.24057600
H	4.99969200	0.50891100	-0.06826100
C	3.50789400	-2.58980700	-1.78654000
C	4.87323600	-2.33889900	-1.87629500
H	6.47148800	-1.00918900	-1.29238500
H	3.08746000	-3.47005900	-2.26269200
H	5.51419500	-3.01615600	-2.43215600
C	1.21584200	-2.16984000	-1.02775300
C	0.44784300	-2.17558100	-2.20223800
C	0.66500600	-2.71587400	0.14240900
C	-0.83538700	-2.72570300	-2.20964300
H	0.87238400	-1.76546800	-3.11397000
C	-0.61563600	-3.27085800	0.13340800
H	1.25840000	-2.73979600	1.04947700
C	-1.36402300	-3.28593600	-1.04459300
H	-1.40958500	-2.74190400	-3.13164200
H	-1.01145200	-3.71196400	1.04312300
H	-2.34357400	-3.75437100	-1.06244200
C	-4.20460900	-1.19854600	0.94651200
C	-4.54580300	0.06570300	1.61575200
C	-4.57379400	-1.38656300	-0.50864000
H	-4.74945100	-0.44968400	-1.03434800
H	-3.81560600	-1.95987600	-1.04726700
H	-5.50941700	-1.95811700	-0.53073300
C	-4.10191900	-2.49199200	1.71991500
H	-5.06851100	-3.00359300	1.63530700

H	-3.33862700	-3.14489800	1.28803300
H	-3.88727600	-2.34672500	2.77748400
H	-5.08601100	0.80591200	1.03818000
H	-4.80292400	0.04527600	2.67047700

Cartesian coordinates of the optimized geometry for **26a** at B3LYP-D3BJ/6-31G(d),def2-TZVP level of theory (number of imaginary frequencies = 1):

C	2.17906400	0.51379400	-0.22850500
C	3.04099900	-0.37865100	-0.67949200
C	2.73695300	1.74521200	0.38466400
Cl	2.45993700	-1.99365000	-1.44660300
C	3.77793100	2.46824000	-0.22275800
C	2.20800700	2.21691900	1.59933600
C	2.72949700	3.35810000	2.20263200
C	3.77105400	4.06423700	1.59412000
C	4.28708700	3.62060900	0.37605900
H	5.08208600	4.17540800	-0.11348300
H	4.16026700	2.14883100	-1.18784300
H	1.39312200	1.67182600	2.06301500
H	2.31545100	3.70562000	3.14472000
Au	0.12161700	0.31035700	-0.37018500
C	4.58448500	-2.14270900	-0.83338800
C	4.52212900	-0.62663500	-0.73839100
H	4.16728100	4.96125400	2.05999200
P	-2.21326800	0.35983400	-0.66560000
C	-2.60429700	-0.33509500	-2.39399500
C	-2.74274800	2.16456000	-0.41337600
H	5.02860300	-0.24374300	0.15604000
C	5.30739900	-2.76920500	-1.97437300
H	5.07508500	-3.83106700	-2.08432600
H	6.38109200	-2.68465200	-1.73803300
H	5.13522300	-2.24172700	-2.91471700
C	4.50647500	-2.89191900	0.45444600
H	4.23638600	-3.94074900	0.31340300
H	3.82516100	-2.41097900	1.15886700
H	5.51939000	-2.86107600	0.88721500
H	4.98543800	-0.17284900	-1.62201100
C	-3.21187600	-0.61270000	0.53626000
C	-4.60874200	-0.67556400	0.36791200
C	-2.63714600	-1.29154500	1.63439700
C	-5.42509500	-1.37399900	1.25148000
H	-5.07281600	-0.16560700	-0.46418200
C	-3.47699300	-1.98315300	2.52292600
C	-4.85561300	-2.02891800	2.34224300
H	-6.49797000	-1.40127300	1.08833200
H	-3.02356500	-2.49786100	3.36433100
H	-5.47802300	-2.57456700	3.04474900
C	-1.17658700	-1.36908500	1.94143000
C	-0.59722900	-0.50431900	2.88037400
C	-0.40258100	-2.41020700	1.40963200
C	0.73048700	-0.67388400	3.27515700
H	-1.19681500	0.29235100	3.31010300
C	0.92242300	-2.58368400	1.81078400
H	-0.85292900	-3.09511200	0.69744800
C	1.49185200	-1.71658200	2.74395300
H	1.16454000	-0.00382600	4.01116000
H	1.49903300	-3.40913700	1.40389600
H	2.51557900	-1.86109800	3.07827700
C	-2.07905500	3.02872500	-1.50027500
H	-2.22668600	4.08633300	-1.25285800
H	-1.00109600	2.84663400	-1.56535100
H	-2.52141500	2.85813200	-2.48601700
C	-2.18098400	2.56263100	0.96787500
H	-1.08771100	2.51287900	0.99278600

H	-2.47968900	3.59368700	1.19024100
H	-2.57563600	1.92075700	1.76258700
C	-4.25931200	2.41406600	-0.40661200
H	-4.74872800	1.91580400	0.43317400
H	-4.43089200	3.49112500	-0.29390900
H	-4.74711700	2.10168600	-1.33248900
C	-1.48441300	0.10845500	-3.35852200
H	-1.68611400	-0.31552800	-4.34938600
H	-1.42926300	1.19409500	-3.46590800
H	-0.50396000	-0.24746200	-3.02841600
C	-2.54625800	-1.86885200	-2.25332500
H	-2.61737600	-2.32114900	-3.24936200
H	-1.59962000	-2.19299800	-1.80517200
H	-3.36517900	-2.25775400	-1.64301100
C	-3.95425700	0.08562600	-2.99589900
H	-4.01578900	1.16575700	-3.15192800
H	-4.06095400	-0.39108000	-3.97764700
H	-4.80795900	-0.23135100	-2.39402600

Cartesian coordinates of the optimized geometry for **27a** at B3LYP-D3BJ/6-31G(d),def2-TZVP level of theory (number of imaginary frequencies = 0):

C	2.58581100	0.17576200	-0.87207600
C	2.39168100	-1.04557400	-0.89644900
C	3.07429400	1.52113200	-0.81420200
Cl	0.86631300	-3.24773100	0.93969800
C	4.22291800	1.79027000	-0.04851000
C	2.42599100	2.56430500	-1.49589800
C	2.90998400	3.86456100	-1.39339700
C	4.04402000	4.13192900	-0.62128500
C	4.70310300	3.09302100	0.04237700
H	5.58955400	3.29986900	0.63354800
H	4.71869200	0.97900100	0.47339900
H	1.55133100	2.34570400	-2.09871600
H	2.40594500	4.67008600	-1.91783000
Au	0.32155500	-0.19890000	-0.57215600
C	2.57960700	-3.31861300	0.23813000
C	2.58148400	-2.49571100	-1.06803600
H	4.41802800	5.14787800	-0.54208200
P	-2.00700400	0.03527400	-0.63296000
C	-2.71880200	-1.67277700	-1.09319200
C	-2.39205600	1.40618400	-1.88618700
H	3.56135900	-2.63909000	-1.54428100
C	2.87767700	-4.78204300	-0.07546900
H	2.81717700	-5.38767900	0.83181400
H	3.89277400	-4.86943000	-0.48046900
H	2.17323200	-5.18385000	-0.80910800
C	3.53138300	-2.74352100	1.28030200
H	3.50335700	-3.34024200	2.19538600
H	3.27216100	-1.71102200	1.52485200
H	4.55476800	-2.76606400	0.88618800
H	1.82997900	-2.89698300	-1.75435000
C	-2.79549000	0.54797700	0.94102600
C	-4.20280300	0.55944400	1.00529700
C	-2.06004500	0.89698800	2.09602600
C	-4.87785900	0.90359500	2.17126700
H	-4.78519700	0.29061300	0.13476400
C	-2.76021300	1.23871800	3.26459000
C	-4.15062400	1.24546200	3.31083600
H	-5.96313400	0.90083100	2.18758300
H	-2.18699500	1.50450000	4.14712500
H	-4.66179300	1.51487100	4.22983600
C	-0.57050200	0.92943900	2.19681800
C	0.15224800	2.07082900	1.81552100
C	0.10921900	-0.13548500	2.80305900

C	1.52826400	2.14129600	2.03054200
H	-0.37135200	2.90920300	1.36795400
C	1.48613400	-0.06316900	3.01914200
H	-0.44458200	-1.01806700	3.10695600
C	2.19692500	1.07451300	2.63514500
H	2.07683000	3.02747700	1.72811600
H	1.99615100	-0.89374000	3.49682500
H	3.26591800	1.13710300	2.81401600
C	-1.84164500	0.98942300	-3.26118000
H	-1.93641200	1.83365000	-3.95353500
H	-0.78297800	0.71229100	-3.21114700
H	-2.39680700	0.15060600	-3.69011700
C	-1.64309000	2.65669500	-1.38496400
H	-0.56574600	2.48775400	-1.30181000
H	-1.80675700	3.47620300	-2.09415200
H	-2.01534100	2.98031100	-0.40815800
C	-3.88306100	1.76502000	-2.00504500
H	-4.28116800	2.15385000	-1.06529900
H	-3.98531800	2.55685800	-2.75617100
H	-4.50077900	0.92641300	-2.32954100
C	-1.71886900	-2.37952500	-2.03282900
H	-2.14609300	-3.34214800	-2.33641500
H	-1.51460800	-1.80473000	-2.93986900
H	-0.77128700	-2.58199500	-1.52629100
C	-2.81260700	-2.47474200	0.21951700
H	-3.03901800	-3.51924500	-0.02247800
H	-1.86744800	-2.45992600	0.77125700
H	-3.59955300	-2.09788900	0.87646900
C	-4.09048200	-1.64066300	-1.78719800
H	-4.04809500	-1.15060200	-2.76323900
H	-4.41190900	-2.67489000	-1.95617800
H	-4.86556900	-1.16054300	-1.18708400

Cartesian coordinates of the optimized geometry for **28a** at B3LYP-D3BJ/6-31G(d),def2-TZVP level of theory (number of imaginary frequencies = 0):

C	2.14455500	-0.54564900	-0.98116200
C	3.28275600	0.12532300	-0.48565900
Au	0.21362100	-0.05892200	-0.52040200
P	-1.99722000	0.73448400	-0.28105400
C	-2.00592300	2.20879800	0.90807700
C	-2.60695900	1.13934100	-2.04084500
C	-1.40522800	1.67640000	2.22333000
H	-0.39581800	1.28475400	2.07939500
H	-1.34841500	2.49916800	2.94572100
H	-2.03196700	0.89160000	2.65851100
C	-3.39547700	2.78800800	1.21815000
H	-3.26964600	3.63837900	1.89875400
H	-3.91376000	3.15407800	0.33051600
H	-4.03399600	2.05918000	1.72268600
C	-1.09307400	3.30614200	0.33275500
H	-0.10170700	2.92269300	0.07216900
H	-1.53082900	3.77833500	-0.55160700
H	-0.95691900	4.08786400	1.08896300
C	-1.43161500	1.74897300	-2.83434200
H	-0.59582500	1.04864900	-2.92104000
H	-1.77935600	1.98700800	-3.84629500
H	-1.05573000	2.67167100	-2.38572700
C	-3.79506200	2.11047300	-2.12799700
H	-3.53771000	3.10606600	-1.75735800
H	-4.07488200	2.21891700	-3.18244800
H	-4.68124700	1.75656900	-1.59815200
C	-2.98180000	-0.21120900	-2.68200800
H	-2.16169800	-0.93466800	-2.60552800
H	-3.87116300	-0.65170700	-2.22505000

H	-3.18738000	-0.05235700	-3.74677000
C	-3.16785800	-0.49306300	0.42682800
C	-4.54156400	-0.18452200	0.44711400
C	-2.75114200	-1.75265800	0.91660000
C	-5.48779400	-1.08392500	0.92757900
H	-4.88347900	0.77236600	0.07803400
C	-3.72110600	-2.64924700	1.39368800
C	-5.07484100	-2.32769800	1.40260900
H	-6.53900800	-0.81337600	0.92675900
H	-3.39131300	-3.61383500	1.76673500
H	-5.80004500	-3.04253000	1.77887600
C	-1.33825800	-2.23682200	0.97597400
C	-0.87399400	-3.15668300	0.02324300
C	-0.50269100	-1.89408000	2.04964500
C	0.39843000	-3.71804100	0.13851800
H	-1.52165900	-3.43670300	-0.80207600
C	0.76990500	-2.45833600	2.16554200
H	-0.86245700	-1.20396600	2.80524000
C	1.22067300	-3.37391400	1.21278000
H	0.74015300	-4.43296600	-0.60351000
H	1.39493100	-2.20259800	3.01680700
H	2.19852900	-3.83421700	1.31696000
C	4.70793200	-0.73534900	0.00412200
C	4.65889400	0.06598300	-1.20847900
Cl	2.34647800	-1.74964000	-2.21779100
C	2.96314600	1.31289200	0.37866400
C	2.45435300	1.13320900	1.67202500
C	3.13527300	2.61014500	-0.11499400
C	2.14316900	2.23543800	2.46596000
H	2.30439600	0.12612100	2.04922700
C	2.81343700	3.71424000	0.67703300
H	3.51220300	2.75922200	-1.12290400
C	2.32270400	3.52889400	1.97019500
H	1.76104100	2.08544200	3.47104100
H	2.94764900	4.71677100	0.28265100
H	2.08196200	4.38764300	2.58919700
C	4.70520800	-2.24209900	-0.00855900
H	4.21325500	-2.63953300	0.88347600
H	4.25066800	-2.67925700	-0.89418700
H	5.75310700	-2.56729800	0.01857800
C	5.32505600	-0.14279200	1.24909200
H	4.81533100	-0.48236400	2.15537900
H	6.36076200	-0.50328600	1.29671500
H	5.34275900	0.94679300	1.24257400
H	4.64042100	-0.43901800	-2.16959300
H	5.17434700	1.02284100	-1.20554000

Cartesian coordinates of the optimized geometry for **29a** at B3LYP-D3BJ/6-31G(d),def2-TZVP level of theory (number of imaginary frequencies = 0):

C	-2.15268200	-0.21297600	0.54199600
C	-3.24821900	0.45386600	0.15983200
Au	-0.12752400	-0.03417200	0.53274600
P	2.16860600	0.45786200	0.64285500
C	2.37454500	2.27275400	0.13235600
C	2.75227700	0.07072500	2.41380700
C	1.69508200	2.39044400	-1.24754400
H	0.62479300	2.17268300	-1.19489900
H	1.81269800	3.41712400	-1.61367700
H	2.15370500	1.71908100	-1.98101100
C	3.82987500	2.74770400	-0.00283800
H	3.82158000	3.81174000	-0.26695300
H	4.39954900	2.64579300	0.92298700
H	4.35680400	2.21711200	-0.79905300
C	1.62600400	3.15872700	1.14377400

H	0.59393100	2.82462900	1.29403700
H	2.12817800	3.18779100	2.11494500
H	1.59331400	4.18494000	0.76006600
C	1.61740100	0.44410200	3.39081200
H	0.70669800	-0.12772600	3.18942300
H	1.94383800	0.21339200	4.41159100
H	1.36393600	1.50608100	3.35409800
C	4.03140800	0.79293000	2.86677000
H	3.90365300	1.87817600	2.89151300
H	4.26793300	0.47011100	3.88749000
H	4.89966800	0.55605700	2.24935700
C	2.96150900	-1.45517100	2.46827800
H	2.07020600	-1.99445000	2.12636800
H	3.81045700	-1.77720400	1.86035300
H	3.15214500	-1.75115600	3.50623900
C	3.24307600	-0.49897400	-0.50309100
C	4.63935200	-0.33826500	-0.41270700
C	2.72909100	-1.37525000	-1.48595700
C	5.51246900	-1.00970000	-1.26212900
H	5.05715600	0.32786100	0.32885100
C	3.62487000	-2.03632600	-2.34246700
C	5.00125400	-1.86251900	-2.23906300
H	6.58322600	-0.86267700	-1.16136700
H	3.21841700	-2.70463200	-3.09514300
H	5.66777600	-2.39128100	-2.91337100
C	1.28413100	-1.68913100	-1.70299500
C	0.67137500	-2.73223200	-0.99159400
C	0.56603700	-1.04970700	-2.72252900
C	-0.63287700	-3.12637300	-1.29469100
H	1.23224100	-3.24684900	-0.21702500
C	-0.73880300	-1.44342200	-3.02476500
H	1.04088200	-0.25411000	-3.28861900
C	-1.33815400	-2.48508300	-2.31467500
H	-1.08829000	-3.94525100	-0.74560900
H	-1.27844300	-0.94986700	-3.82784000
H	-2.34002300	-2.81306100	-2.57480500
C	-4.65154200	-1.51092800	0.82660900
C	-4.62245500	0.00114800	0.60924900
Cl	-2.83520000	-1.78417200	1.49786600
C	-3.09623300	1.68828500	-0.64504100
C	-2.17310500	1.74699200	-1.70173700
C	-3.87962400	2.81793100	-0.36044900
C	-2.02049700	2.91992800	-2.43879700
H	-1.59945400	0.86255700	-1.95378100
C	-3.72542400	3.98756700	-1.10215000
H	-4.59828700	2.79085000	0.45363300
C	-2.79255500	4.04394100	-2.14005200
H	-1.30706600	2.95030300	-3.25717300
H	-4.33335800	4.85614800	-0.86814900
H	-2.67739600	4.95513900	-2.71892600
C	-5.59625700	-1.98909100	1.90830800
H	-5.51235700	-3.06616200	2.07484200
H	-5.43636700	-1.46090800	2.85143100
H	-6.61906000	-1.78197000	1.56973800
C	-4.71409400	-2.30925800	-0.46074800
H	-4.56867400	-3.37695300	-0.27923200
H	-5.71232900	-2.16992500	-0.89327200
H	-3.97152900	-1.95686000	-1.17946200
H	-4.90000400	0.51846700	1.53646100
H	-5.37988500	0.25176800	-0.14215600

Cartesian coordinates of the optimized geometry for **30a** at B3LYP-D3BJ/6-31G(d),def2-TZVP level of theory (number of imaginary frequencies = 0):

C	-1.96561900	-0.09636400	-0.11893900

C	-3.20332300	-0.08862100	-0.46399200
Au	-0.03626000	0.12129900	0.23783400
C	-4.42710600	-0.94422200	-0.32126400
C	-4.54188200	-1.66674300	1.03132900
P	2.24504100	0.51971600	0.69152500
C	2.56837600	0.07443200	2.50380000
C	2.58054300	2.33894100	0.24416600
C	-3.17812500	1.30594200	-1.08867000
C	-2.69387900	1.49256500	-2.39373500
C	-3.68502600	2.39000700	-0.34874000
C	-2.76848000	2.75500200	-2.97904400
H	-2.26973800	0.64908200	-2.92779000
C	-3.76568000	3.64232800	-0.94738500
H	-4.02099400	2.22814900	0.67045700
C	-3.30706900	3.82404300	-2.25863200
H	-2.40462500	2.90619800	-3.99028000
H	-4.17360500	4.48130800	-0.39273600
H	-3.36315000	4.80733700	-2.71563800
Cl	-4.60922800	-0.36525000	2.34286600
H	-4.42075900	-1.70355000	-1.11576800
H	-5.30769900	-0.31733700	-0.48342300
C	-5.85321500	-2.44859300	1.09048600
H	-5.97172000	-2.92390400	2.06714000
H	-5.84859900	-3.23196100	0.32301900
H	-6.71198800	-1.79477500	0.91674300
C	-3.33982500	-2.55794100	1.32350500
H	-3.26130100	-3.33025100	0.54902000
H	-3.45251400	-3.04840000	2.29361700
H	-2.40602700	-1.98675500	1.34077200
C	2.06126000	-1.37295100	2.66844800
H	2.60028400	-2.06433000	2.01262600
H	2.22999400	-1.69327100	3.70283300
H	0.98977900	-1.45796800	2.45920300
C	1.73741700	1.01294700	3.39667200
H	0.68432100	1.04090600	3.09676700
H	1.78122300	0.64930500	4.42954200
H	2.12772000	2.03446700	3.39263800
C	4.04436700	0.11878700	2.93161100
H	4.50058000	1.10082700	2.79027900
H	4.09895500	-0.11632000	4.00090700
H	4.64207100	-0.62702900	2.40290200
C	2.69789800	2.38252600	-1.29233700
H	2.74351600	3.42951200	-1.61305900
H	3.59718300	1.87645000	-1.65143600
H	1.82800200	1.92295100	-1.77637500
C	1.35865800	3.18192500	0.66596200
H	1.54810100	4.22814900	0.39939000
H	0.44794200	2.86361900	0.14910700
H	1.16962200	3.14237900	1.74088900
C	3.83836900	2.94641600	0.88422800
H	3.94222700	3.97936100	0.53205600
H	3.76954500	2.98087500	1.97460900
H	4.75346700	2.42071700	0.60522300
C	3.39410900	-0.49553500	-0.32147000
C	4.77730200	-0.25772200	-0.20228000
C	2.95260700	-1.45785000	-1.26004200
C	5.70804700	-0.93843900	-0.98025700
H	5.13886000	0.47379500	0.50663600
C	3.90716600	-2.13068300	-2.04001700
C	5.26966900	-1.88103400	-1.90882400
H	6.76652400	-0.72945700	-0.86158700
H	3.55855000	-2.86766800	-2.75667000
H	5.98205600	-2.41979700	-2.52579500
C	1.52971300	-1.84019600	-1.51013600
C	0.86662700	-1.36708400	-2.65253900
C	0.88116800	-2.77581900	-0.68960800

C	-0.42066800	-1.81117400	-2.96082800
H	1.37118900	-0.65824000	-3.30239700
C	-0.40613000	-3.22032600	-1.00017800
H	1.39701900	-3.16971000	0.18019500
C	-1.05966700	-2.73860500	-2.13597400
H	-0.91458700	-1.44733600	-3.85745800
H	-0.88724500	-3.95535200	-0.36217200
H	-2.05402000	-3.09687000	-2.38640100

Cartesian coordinates of the optimized geometry for **31a** at B3LYP-D3BJ/6-31G(d),def2-TZVP level of theory (number of imaginary frequencies = 1):

C	-1.95967200	0.24054300	-0.32974400
C	-2.95556800	-0.52837000	-0.26547000
Au	0.04769600	0.39411400	0.04189700
C	-4.07744100	-1.43658600	-0.09282000
C	-4.32003100	-1.84434600	1.38273800
P	2.31324900	0.61036900	0.59336400
C	2.50198600	0.23718000	2.44184500
C	2.87057000	2.35602400	0.07995600
C	-2.95103300	1.27868500	-0.98169000
C	-3.06095300	1.32850700	-2.38191400
C	-3.61651700	2.21582400	-0.17474700
C	-3.80146900	2.34753100	-2.97263000
H	-2.55094900	0.58181200	-2.98140000
C	-4.35563500	3.22897400	-0.77811800
H	-3.54663000	2.13637500	0.90401600
C	-4.44739500	3.29611800	-2.17237900
H	-3.88261400	2.40055200	-4.05369900
H	-4.86794000	3.96155700	-0.16271700
H	-5.03053900	4.08531700	-2.63671200
Cl	-4.76047900	-0.31404000	2.30481200
H	-3.89629600	-2.34404000	-0.68547400
H	-4.97776300	-0.96449600	-0.50079200
C	-5.50629700	-2.80130900	1.45978000
H	-5.71390400	-3.06412400	2.49980700
H	-5.27335700	-3.72216800	0.91181100
H	-6.40671500	-2.35486300	1.02900000
C	-3.06169100	-2.43148100	2.01660800
H	-2.76976900	-3.33983300	1.47579300
H	-3.24977900	-2.69680700	3.05961000
H	-2.22911400	-1.72276200	1.98277300
C	1.84658400	-1.14299900	2.65335400
H	2.34390000	-1.91859500	2.06192700
H	1.93373000	-1.42001100	3.71025700
H	0.78395800	-1.13416200	2.38990300
C	1.72626700	1.29901100	3.24102800
H	0.69585500	1.40628600	2.88537800
H	1.68846000	0.99430000	4.29316400
H	2.21139300	2.27830200	3.20123400
C	3.95361600	0.16270500	2.94244600
H	4.50825300	1.08891600	2.77968200
H	3.93631900	-0.02321600	4.02270000
H	4.50046500	-0.66071800	2.47763600
C	3.09660200	2.30012200	-1.44357800
H	3.27238000	3.31687400	-1.81295800
H	3.96023500	1.68625200	-1.71043400
H	2.21788200	1.90346500	-1.96560000
C	1.71399800	3.33648800	0.36744500
H	2.02325100	4.34237300	0.06063600
H	0.81304900	3.07387500	-0.19540900
H	1.45150000	3.37783800	1.42704700
C	4.13465400	2.87936200	0.78060800
H	4.36444900	3.87414000	0.38131000
H	3.99221800	2.98807400	1.85886000

H	5.01210900	2.25468500	0.60341700
C	3.40264300	-0.58688400	-0.27739400
C	4.79644600	-0.46883900	-0.11242100
C	2.90729600	-1.61036800	-1.11801000
C	5.68655300	-1.32787500	-0.74818200
H	5.19850200	0.30652000	0.52449800
C	3.82149600	-2.47038100	-1.74898400
C	5.19520400	-2.33887200	-1.57237200
H	6.75470400	-1.20583900	-0.59864300
H	3.43145500	-3.25376300	-2.39124300
H	5.87488200	-3.01935800	-2.07579700
C	1.46771700	-1.87723800	-1.42660700
C	0.89550600	-1.36044600	-2.60017300
C	0.72282300	-2.77677200	-0.65018600
C	-0.39133000	-1.73638600	-2.98786900
H	1.47519100	-0.67984100	-3.21655400
C	-0.56474100	-3.15152400	-1.03887500
H	1.16526300	-3.19866400	0.24669200
C	-1.12207200	-2.63708800	-2.21076700
H	-0.81124500	-1.34338700	-3.90944300
H	-1.11822300	-3.86716400	-0.43747600
H	-2.11187400	-2.95140800	-2.53016900

Cartesian coordinates of the optimized geometry for **34a** at B3LYP-D3BJ/6-31G(d),def2-TZVP level of theory (number of imaginary frequencies = 1):

C	-2.17613900	0.45521500	0.39479000
C	-2.67447000	-0.37801700	1.21427100
C	-2.61456600	1.48815600	-0.54001700
Cl	-2.47681800	-1.49256400	2.43552800
C	-3.29455700	2.62032300	-0.06210700
C	-2.33704400	1.37194200	-1.91203600
C	-2.77694300	2.35380400	-2.79574900
C	-3.47242100	3.46933800	-2.32091600
C	-3.72310300	3.60271900	-0.95388600
H	-4.25063000	4.47417100	-0.57851100
H	-3.47193300	2.72809100	1.00307000
H	-1.79462000	0.50428500	-2.27036100
H	-2.57380900	2.25102600	-3.85740300
Au	-0.04046900	0.27391200	0.48212600
H	-3.80916900	4.23525800	-3.01244200
P	2.27357800	0.52067600	0.42967600
C	2.94341500	0.27875100	2.18519700
C	2.59733100	2.24189000	-0.31995300
C	2.41020700	-1.09147400	2.64968000
H	1.31594100	-1.11928300	2.66731300
H	2.77257200	-1.28767900	3.66524300
H	2.76891500	-1.90082700	2.00553300
C	4.47738300	0.26049500	2.29366800
H	4.74519000	0.15940500	3.35183300
H	4.94155500	1.17781900	1.92591000
H	4.91077900	-0.59106300	1.76454300
C	2.36908700	1.38457500	3.08782300
H	1.27929500	1.45612600	3.00456100
H	2.80020600	2.36373400	2.86179900
H	2.61177200	1.15180300	4.13088300
C	1.53828700	3.22646400	0.21903700
H	0.52428000	2.92345700	-0.05858200
H	1.72619100	4.21300600	-0.22008000
H	1.57611600	3.33402300	1.30523500
C	3.99195400	2.82188000	-0.03503200
H	4.15570000	2.99409200	1.03193400
H	4.07143400	3.79318300	-0.53688000
H	4.80024900	2.19738700	-0.41993800
C	2.38993700	2.08754500	-1.83966800

H	1.41325700	1.64572400	-2.06929900
H	3.16465100	1.47084500	-2.30145500
H	2.42245200	3.08070700	-2.30176400
C	3.13580400	-0.69113500	-0.64907600
C	4.52227400	-0.53694800	-0.84535300
C	2.46347700	-1.71604600	-1.35547200
C	5.23605600	-1.35584900	-1.71399600
H	5.05788400	0.23875000	-0.31677800
C	3.20046400	-2.52666300	-2.23482900
C	4.56882000	-2.35611900	-2.41898200
H	6.30394700	-1.20794700	-1.83995100
H	2.67623000	-3.31099400	-2.77169300
H	5.10889400	-3.00188000	-3.10435500
C	1.00924900	-2.04228700	-1.25152200
C	0.14439900	-1.73110100	-2.31152000
C	0.51671500	-2.79575100	-0.17466800
C	-1.18538600	-2.15645200	-2.29067700
H	0.52547900	-1.16682700	-3.15768300
C	-0.81089400	-3.22513500	-0.15737200
H	1.18568300	-3.06575200	0.63573300
C	-1.66460500	-2.90545400	-1.21456100
H	-1.83950800	-1.92163500	-3.12557900
H	-1.17122500	-3.82320500	0.67357700
H	-2.69022700	-3.26169500	-1.21343000
C	-5.13460600	-1.09658900	0.36575000
C	-5.05622600	-0.08288500	1.26573600
C	-5.08636300	-0.84498200	-1.11389600
H	-5.04965600	0.21914600	-1.35315500
H	-4.20393100	-1.32663400	-1.55267600
H	-5.96468500	-1.28856600	-1.59938400
C	-5.28201200	-2.52691100	0.79551300
H	-6.21356400	-2.94397100	0.39184400
H	-4.46587900	-3.14018700	0.39245500
H	-5.29909800	-2.63622000	1.88207400
H	-5.05038000	0.95090900	0.93895000
H	-5.16420300	-0.26395000	2.33075300

Cartesian coordinates of the optimized geometry for **35a** at B3LYP-D3BJ/6-31G(d),def2-TZVP level of theory (number of imaginary frequencies = 1):

C	2.04974600	-0.54107000	-1.23784000
C	2.69075800	0.30190900	-0.51720900
Au	0.02018800	-0.09507100	-0.81778400
P	-2.17730700	0.57553000	-0.40074300
C	-2.05754900	2.28417700	0.42109200
C	-3.13142800	0.54877000	-2.04474000
C	-1.11991500	2.09730200	1.63139800
H	-0.11364400	1.79713900	1.32751300
H	-1.03875000	3.05002500	2.16763300
H	-1.50717700	1.34960700	2.33129400
C	-3.39331900	2.85306200	0.92704300
H	-3.20627000	3.84977900	1.34357500
H	-4.13710500	2.96469800	0.13572700
H	-3.81817400	2.23990600	1.72463300
C	-1.41360300	3.26687600	-0.57243100
H	-0.47344600	2.87829700	-0.97837400
H	-2.08011600	3.50735400	-1.40548200
H	-1.19061200	4.20372800	-0.04881500
C	-2.16965100	0.98691900	-3.16904700
H	-1.31073100	0.31381400	-3.25114900
H	-2.71015300	0.96227200	-4.12227000
H	-1.79232000	2.00228000	-3.02652800
C	-4.37842700	1.44647300	-2.09623000
H	-4.12810100	2.50454900	-1.98407900
H	-4.84998800	1.32632300	-3.07847900

H	-5.12851400	1.18185400	-1.34842200
C	-3.52888200	-0.92066400	-2.28407400
H	-2.65752800	-1.58404900	-2.24031000
H	-4.26618900	-1.27273400	-1.55841600
H	-3.96423100	-1.01125700	-3.28567000
C	-3.08962600	-0.47822700	0.79789300
C	-4.44082600	-0.17648500	1.05576800
C	-2.48449700	-1.53525700	1.51817600
C	-5.18025400	-0.87317800	2.00597500
H	-4.92645800	0.62274600	0.51338700
C	-3.24133700	-2.21275500	2.48850500
C	-4.57210300	-1.89211700	2.73726900
H	-6.22100800	-0.61559000	2.17523700
H	-2.76768600	-3.02076600	3.03718600
H	-5.13142900	-2.44098000	3.48851100
C	-1.09496700	-2.04243800	1.31432200
C	-0.79903300	-2.87815000	0.22445700
C	-0.10413400	-1.81027000	2.27723800
C	0.46341000	-3.45904800	0.09653100
H	-1.57336100	-3.09059700	-0.50639600
C	1.15946300	-2.39186400	2.14745100
H	-0.33220500	-1.18164800	3.13302700
C	1.44700100	-3.21425800	1.05711400
H	0.67456000	-4.10681200	-0.74810700
H	1.91095100	-2.22317700	2.91431900
H	2.42384100	-3.67889300	0.96412400
C	5.31129900	-0.47329900	-0.07639700
C	5.02690800	0.19095300	-1.22393800
Cl	2.49125000	-1.77678100	-2.32872600
C	2.73690400	1.35050600	0.45060600
C	2.56552900	1.04773700	1.81699300
C	2.91746800	2.68926500	0.04820400
C	2.56511200	2.07050600	2.75947900
H	2.40173500	0.01664100	2.10721600
C	2.91869400	3.70334400	0.99864800
H	3.04626500	2.91417700	-1.00542300
C	2.74419300	3.39581100	2.35301500
H	2.42345400	1.83750900	3.81008500
H	3.05291500	4.73461700	0.68830300
H	2.74625500	4.19183000	3.09125100
C	5.23851700	-1.97031000	0.02334300
H	4.46046700	-2.26553100	0.73968800
H	5.01698500	-2.43958900	-0.93688400
H	6.18526800	-2.37265000	0.40448000
C	5.72865800	0.25530200	1.16652300
H	5.10619000	-0.03631000	2.02169600
H	6.76108900	-0.01492400	1.42513700
H	5.67458600	1.34028200	1.05084200
H	4.82752300	-0.33670800	-2.15026600
H	5.14201000	1.26858500	-1.28622700

Cartesian coordinates of the optimized geometry for **47a** at B3LYP-D3BJ/6-31G(d),def2-TZVP level of theory (number of imaginary frequencies = 1):

C	-2.45596800	-0.44619500	-0.11313800
C	-3.15203600	-1.63490500	-0.48223900
C	-2.96643700	0.35056900	1.05875300
Cl	-2.41372900	-3.15267900	-0.78446000
C	-3.89764100	-0.18795200	1.96052900
C	-2.47636400	1.64539500	1.28542200
C	-2.93015200	2.39319000	2.36794300
C	-3.86567200	1.85604500	3.25608100
C	-4.33978000	0.56033300	3.05252900
H	-5.05062600	0.12321200	3.74729000
H	-4.26084200	-1.20347100	1.82903900

H	-1.73686200	2.06005100	0.61155400
H	-2.54173500	3.39532900	2.52426700
Au	-0.33774300	-0.44493700	-0.13665000
C	-3.43619800	-0.06897000	-1.55850800
C	-4.38281200	-1.25463700	-1.18839400
H	-4.20985200	2.43624200	4.10637900
P	1.96947700	-0.76767900	0.23600700
C	2.56746900	-2.18975700	-0.88426900
C	2.05359600	-1.16260900	2.09772900
H	-5.19044900	-0.91696300	-0.53745900
C	-2.63660400	-0.25050600	-2.83715900
H	-1.81809200	0.47068200	-2.86350700
H	-3.28458600	-0.07271400	-3.70316200
H	-2.20707400	-1.25122500	-2.93645600
C	-4.07148800	1.31142100	-1.43397900
H	-3.29998500	2.08449000	-1.45000800
H	-4.66160000	1.42117300	-0.52469800
H	-4.72802200	1.44859200	-2.30209700
H	-4.73679100	-1.93073400	-1.96616500
C	3.14166800	0.63122600	0.00723800
C	4.50726100	0.39463400	0.26011000
C	2.73702600	1.94753700	-0.31237100
C	5.45062500	1.41610200	0.23591600
H	4.84367600	-0.60426700	0.49668600
C	3.70054900	2.97237500	-0.30359300
C	5.04038700	2.72117800	-0.03023800
H	6.49428700	1.19213200	0.43289400
H	3.38037400	3.97950700	-0.55095400
H	5.75892000	3.53482700	-0.04180500
C	1.37566200	2.37234900	-0.74640400
C	0.87848200	1.98345300	-2.00012300
C	0.67329900	3.33807500	-0.01165600
C	-0.27725000	2.57005400	-2.51696500
H	1.43775800	1.26761000	-2.59397300
C	-0.49113900	3.91372200	-0.52253500
H	1.05800100	3.65252300	0.95401000
C	-0.96427900	3.53869100	-1.78145600
H	-0.61711000	2.30029300	-3.51251300
H	-1.01612900	4.67023800	0.05350800
H	-1.84713300	4.01588800	-2.19666500
C	1.22951700	-2.43412500	2.36996600
H	1.13151600	-2.56769800	3.45341200
H	0.22108700	-2.36655200	1.94826000
H	1.71335200	-3.33058100	1.97144000
C	1.38807000	0.04184100	2.79764100
H	0.33641900	0.15358300	2.51455700
H	1.43291400	-0.10852300	3.88252500
H	1.90732700	0.97810100	2.56756500
C	3.46492100	-1.33403400	2.68280000
H	4.04044500	-0.40752800	2.63437400
H	3.36497500	-1.60178000	3.74126300
H	4.03626400	-2.12880800	2.19897000
C	1.39211400	-3.16509300	-1.09827800
H	0.54785200	-2.67123900	-1.58732500
H	1.72737100	-3.98520000	-1.74410000
H	1.03319500	-3.60107300	-0.16278100
C	2.92204500	-1.55327800	-2.24147500
H	3.15621900	-2.34882100	-2.95826000
H	2.07968800	-0.98057000	-2.64590000
H	3.78792900	-0.89008700	-2.17407200
C	3.76658800	-2.99315100	-0.35418100
H	3.53987600	-3.49706700	0.58866900
H	4.01355200	-3.77003200	-1.08738200
H	4.66403500	-2.38667900	-0.22050500

Cartesian coordinates of the optimized geometry for **48a** at B3LYP-D3BJ/6-31G(d),def2-TZVP level of theory (number of imaginary frequencies = 0):

C	-3.58863500	-0.05542600	-0.43392500
C	-3.45242000	0.19796900	-1.75153800
C	-2.96640300	0.42065800	0.78581700
Cl	-2.38990000	1.22662700	-2.65774700
C	-3.24508100	-0.19000100	2.01960000
C	-2.06916500	1.53966500	0.77555800
C	-1.48830100	1.99077500	1.98496900
C	-1.77288300	1.34365400	3.19508500
C	-2.66204400	0.26895000	3.20303200
H	-2.90862900	-0.22011400	4.14053800
H	-3.93111400	-1.02766300	2.05285000
H	-1.99745900	2.16171400	-0.11094400
H	-0.85714800	2.87252900	1.96402800
Au	-0.12917500	0.33207900	0.47702000
H	-1.32489000	1.69660400	4.11782100
P	1.56374700	-1.18314200	0.04046600
C	0.99541900	-2.28112500	-1.39503900
C	1.89381800	-2.10396500	1.67394700
C	0.63911600	-1.31802900	-2.54462200
H	-0.17136500	-0.63511800	-2.27531100
H	0.31112100	-1.90622900	-3.40934700
H	1.50602800	-0.72619200	-2.85549000
C	2.05637000	-3.27022700	-1.90673200
H	1.60548200	-3.87309700	-2.70342500
H	2.40856100	-3.95888700	-1.13631400
H	2.91605600	-2.75269400	-2.33787800
C	-0.26725400	-3.04506600	-0.96009100
H	-1.03029100	-2.37181300	-0.55537000
H	-0.04845600	-3.81369300	-0.21357200
H	-0.69374700	-3.54914700	-1.83500900
C	0.54768100	-2.33850100	2.39092400
H	0.04541800	-1.39604200	2.62953700
H	0.74219000	-2.86521900	3.33225700
H	-0.14094900	-2.95040000	1.80392700
C	2.60633500	-3.45736900	1.52057300
H	1.99997300	-4.18109900	0.96975400
H	2.77952900	-3.87203000	2.52032900
H	3.58127200	-3.37298800	1.03696600
C	2.74818600	-1.15121200	2.53271100
H	2.28140600	-0.16351300	2.62400800
H	3.75330400	-1.01908700	2.12534900
H	2.84028600	-1.57181900	3.54042600
C	3.14223300	-0.40081600	-0.47035200
C	4.26305000	-1.23505800	-0.65094400
C	3.30633500	0.99734500	-0.61604200
C	5.51648800	-0.72030400	-0.96446800
H	4.16190900	-2.30568600	-0.54005600
C	4.58247000	1.49671300	-0.92537100
C	5.67828900	0.65752800	-1.09993800
H	6.35832600	-1.39242000	-1.09711100
H	4.70043500	2.56989600	-1.03697900
H	6.64975000	1.07731100	-1.34156700
C	2.23430800	2.02734100	-0.46727000
C	2.23961000	2.87601600	0.65028900
C	1.30325300	2.26258400	-1.49184400
C	1.33193700	3.93264700	0.74440000
H	2.96875600	2.71039700	1.43786000
C	0.39859900	3.32245700	-1.39841900
H	1.30762200	1.63004300	-2.37316400
C	0.41058400	4.16034700	-0.28112800
H	1.35891000	4.58936600	1.60931000
H	-0.30429800	3.50081900	-2.20596700
H	-0.28138500	4.99494100	-0.21827600

C	-4.70780800	-1.08879300	-0.70790600
C	-4.51781700	-0.73883900	-2.24017600
C	-4.34407400	-2.54105000	-0.38832700
H	-3.35926100	-2.79966300	-0.79195300
H	-4.32775400	-2.72921400	0.69067200
H	-5.08248000	-3.22056700	-0.82817600
C	-6.07857600	-0.69841700	-0.15334500
H	-6.85629800	-1.35642100	-0.55648500
H	-6.10447500	-0.78286700	0.93888300
H	-6.33158000	0.33393400	-0.41673800
H	-4.16931500	-1.56643300	-2.86696600
H	-5.38026000	-0.25989900	-2.71484700

Cartesian coordinates of the optimized geometry for **49a** at B3LYP-D3BJ/6-31G(d),def2-TZVP level of theory (number of imaginary frequencies = 1):

C	-2.18129600	-1.41791500	-0.17556200
C	-3.45476900	-0.81959500	0.11686200
Au	-0.24568900	-0.69380300	0.04447700
C	-4.42271300	-1.24753200	-0.92830000
C	-3.16178600	-1.00172900	-1.76944300
P	2.09905200	-0.54109200	0.29939100
C	2.47252700	-1.04625200	2.10270500
C	2.77318500	-1.76319600	-0.99472700
C	-3.56997800	0.39782300	0.89331600
C	-2.72325100	0.53516500	2.01648300
C	-4.58565600	1.35573000	0.68253200
C	-2.90017900	1.58695100	2.90761900
H	-1.96478300	-0.21567300	2.20226800
C	-4.73447300	2.42383000	1.55674600
H	-5.24660300	1.27467700	-0.17289400
C	-3.89863300	2.53636700	2.67520300
H	-2.26385900	1.66673500	3.78299100
H	-5.50928200	3.16275000	1.38074000
H	-4.03323400	3.36189700	3.36746000
Cl	-2.19382600	-3.22205300	-0.06355800
H	-4.71359500	-2.29624500	-0.90162000
C	-2.88787000	0.39612600	-2.27655400
H	-3.46897400	0.56314200	-3.19198900
H	-1.82903700	0.50751900	-2.52226900
H	-3.13771500	1.17975000	-1.56398400
C	-2.76543700	-2.06259600	-2.78951600
H	-3.31771300	-1.82527800	-3.70802400
H	-3.02063500	-3.07465800	-2.48483300
H	-1.69532700	-2.00932000	-3.00792300
H	-5.28477000	-0.60039500	-1.08077100
C	3.01238000	1.02125000	-0.02738500
C	2.41776300	2.17197200	-0.59343900
C	4.40145900	1.03337700	0.20806300
C	3.24397200	3.25694700	-0.94108500
C	5.19748900	2.12574800	-0.11783400
H	4.87940300	0.16557100	0.63790200
C	4.61497800	3.24349800	-0.71258600
H	2.77833900	4.13703000	-1.37290100
H	6.26371400	2.09639300	0.08345000
H	5.21957400	4.10315500	-0.98459100
C	0.96215900	2.40113200	-0.81604300
C	0.49445500	2.68622500	-2.10768900
C	0.08628100	2.55744700	0.26864100
C	-0.80559200	3.15023000	-2.30845500
H	1.16910000	2.57424700	-2.95154100
C	-1.21136500	3.02918700	0.06825400
H	0.44762700	2.37029300	1.27520700
C	-1.65496200	3.34039800	-1.21722700
H	-1.14518700	3.38407100	-3.31330000

H	-1.86505700	3.17943400	0.91915300
H	-2.65648100	3.73378200	-1.36610200
C	2.21471100	-3.16664600	-0.69753200
H	1.12582700	-3.16334600	-0.58260200
H	2.46120300	-3.82976400	-1.53485000
H	2.65655700	-3.60118500	0.20367900
C	2.21559100	-1.26327600	-2.34423300
H	2.59058800	-1.90846300	-3.14715800
H	1.12097000	-1.29300700	-2.36630700
H	2.53516200	-0.23826200	-2.55926000
C	4.30457200	-1.83413500	-1.10583400
H	4.55844800	-2.59186700	-1.85646700
H	4.73356600	-0.88703700	-1.43938000
H	4.78722300	-2.12804000	-0.17143700
C	1.40356500	-2.06107600	2.56127600
H	1.61411800	-2.34811600	3.59814600
H	0.39702700	-1.63340600	2.52853900
H	1.39981000	-2.97130600	1.95757500
C	3.85344500	-1.67657200	2.35023100
H	3.93527400	-1.92035200	3.41605700
H	3.98565600	-2.60802100	1.79421900
H	4.68216600	-1.00704700	2.11500000
C	2.32163500	0.23456600	2.94549400
H	3.09447000	0.97292200	2.71810800
H	1.34309500	0.70362500	2.78812100
H	2.39874200	-0.02522000	4.00763200

Cartesian coordinates of the optimized geometry for **50a** at B3LYP-D3BJ/6-31G(d),def2-TZVP level of theory (number of imaginary frequencies = 0):

C	-2.38062000	-1.21057600	-0.84310800
C	-2.73133600	0.02556800	-0.30050600
Au	-0.37817000	-0.25979100	-0.18909400
C	-3.03681700	0.58260500	-1.69460500
C	-2.71330000	-0.83404800	-2.29262400
P	1.85357500	-0.72367400	0.38397500
C	1.84475900	-1.26435000	2.20849300
C	2.45172600	-2.05523700	-0.83205200
C	-3.12796900	0.52400200	1.01087600
C	-3.00154900	-0.25462600	2.17755300
C	-3.66885900	1.81743100	1.10626800
C	-3.39380200	0.26220000	3.40839800
H	-2.61004800	-1.26316500	2.11391300
C	-4.06614000	2.32690300	2.33925600
H	-3.77945600	2.41603600	0.20801100
C	-3.92332900	1.55318500	3.49377900
H	-3.29564000	-0.34560000	4.30246100
H	-4.49047700	3.32425700	2.40075800
H	-4.23173000	1.95101800	4.45557100
Cl	-2.40116800	-2.82436300	-0.17258800
H	-4.09383200	0.84864900	-1.79581900
C	-1.58836400	-0.89224100	-3.31940700
H	-1.94854500	-0.55003500	-4.29556700
H	-1.21911400	-1.91703600	-3.43647100
H	-0.74984200	-0.25193900	-3.02546700
C	-3.96126600	-1.57793400	-2.77840700
H	-4.32471600	-1.12877300	-3.70836800
H	-4.76748300	-1.54006900	-2.03840800
H	-3.73069300	-2.62994500	-2.97455700
H	-2.41381200	1.40865300	-2.03768300
C	3.04095300	0.66218100	0.20698600
C	2.68937100	1.90173900	-0.37380000
C	4.38155200	0.44879100	0.58213000
C	3.69931600	2.84918200	-0.61035200
C	5.36293100	1.41053400	0.36577300

H	4.67198600	-0.48676000	1.03875400
C	5.02260900	2.61320800	-0.25188600
H	3.42067400	3.79716900	-1.05955900
H	6.38621900	1.21506700	0.67018300
H	5.77861600	3.36911500	-0.43993700
C	1.30314100	2.33966400	-0.70160700
C	0.92109400	2.57196600	-2.03034300
C	0.41932100	2.69329000	0.33222700
C	-0.31140600	3.16082300	-2.31929200
H	1.60385400	2.31094000	-2.83351000
C	-0.80876300	3.28936400	0.04272000
H	0.72466200	2.54963200	1.36391300
C	-1.17237400	3.53152400	-1.28346500
H	-0.58889600	3.34871000	-3.35231100
H	-1.46895400	3.57470900	0.85439500
H	-2.11771100	4.01622600	-1.50963200
C	1.58725700	-3.31600100	-0.65901200
H	0.51663900	-3.09567100	-0.70872000
H	1.82164000	-4.01667600	-1.46838900
H	1.79260600	-3.82812900	0.28527700
C	2.22398100	-1.45985800	-2.23602700
H	2.59387900	-2.16872200	-2.98551000
H	1.16437900	-1.28203300	-2.43516700
H	2.76521100	-0.51766500	-2.37014000
C	3.93914300	-2.42671800	-0.70573700
H	4.14868300	-3.22978800	-1.42169300
H	4.59044400	-1.58756600	-0.95783700
H	4.20768500	-2.79679000	0.28540300
C	0.55661500	-2.06679700	2.48144800
H	0.56381500	-2.39487100	3.52721200
H	-0.33488200	-1.45261900	2.33030400
H	0.46884500	-2.95561000	1.85309300
C	3.05004000	-2.12091400	2.63063700
H	2.95462000	-2.34528500	3.69925500
H	3.08219000	-3.07582900	2.10026400
H	4.00573100	-1.61085300	2.49671700
C	1.80053200	0.03009200	3.04252300
H	2.71064200	0.62511400	2.93251200
H	0.94233700	0.65373700	2.76822700
H	1.69149700	-0.23455300	4.10034200

Cartesian coordinates of the optimized geometry for **51a** at B3LYP-D3BJ/6-31G(d),def2-TZVP level of theory (number of imaginary frequencies = 1):

C	2.17157700	0.17397700	0.92362800
C	3.15228900	-0.58820700	0.38844800
Au	0.14215200	-0.05643500	0.67161300
P	-2.16369200	-0.50451100	0.52232600
C	-2.34887200	-2.21581600	-0.27849600
C	-2.88617200	-0.38213000	2.28061900
C	-1.57879200	-2.12197700	-1.61128800
H	-0.51701200	-1.91706400	-1.45489000
H	-1.66350100	-3.07907600	-2.13966500
H	-1.99104700	-1.34240900	-2.26093400
C	-3.78903000	-2.65760700	-0.58314400
H	-3.75586900	-3.67121000	-1.00007500
H	-4.42397600	-2.69112700	0.30446000
H	-4.26130900	-2.01398700	-1.32842600
C	-1.66739100	-3.25543400	0.62914300
H	-0.64883500	-2.95631500	0.89823900
H	-2.23360100	-3.43172000	1.54834500
H	-1.60681700	-4.20939600	0.09241900
C	-1.83704500	-0.92552400	3.27351600
H	-0.90344800	-0.35727500	3.22645300
H	-2.23777700	-0.83730100	4.29021300

H	-1.60102200	-1.97782900	3.09881900
C	-4.20455000	-1.13660100	2.51499200
H	-4.09046900	-2.21480600	2.37654100
H	-4.51680900	-0.97360800	3.55333300
H	-5.01942100	-0.78513400	1.87930600
C	-3.08501400	1.12084400	2.55580000
H	-2.15771000	1.68235800	2.39463400
H	-3.86562200	1.55624500	1.92675300
H	-3.37597200	1.25536500	3.60397300
C	-3.14596500	0.62760300	-0.54884900
C	-4.54050000	0.44406100	-0.61618000
C	-2.56169300	1.62803400	-1.36078700
C	-5.34356000	1.19784600	-1.46594900
H	-5.01202700	-0.31017900	-0.00257700
C	-3.38501200	2.36385700	-2.23006900
C	-4.75929800	2.15777400	-2.29023000
H	-6.41563600	1.02874400	-1.48657100
H	-2.92499100	3.12707100	-2.85000300
H	-5.36837500	2.74931900	-2.96676900
C	-1.12261400	2.02469400	-1.35805000
C	-0.60076900	2.79951300	-0.31009600
C	-0.31451700	1.76213000	-2.47207100
C	0.69956600	3.30044500	-0.37547900
H	-1.23096900	3.03161800	0.54262300
C	0.98806700	2.26134700	-2.53776000
H	-0.71647100	1.17529800	-3.29278500
C	1.49555900	3.03316100	-1.49094900
H	1.08415000	3.90378600	0.44034900
H	1.59619700	2.06323800	-3.41613500
H	2.49488700	3.45586500	-1.55912800
C	5.14480700	0.80309400	0.44560600
C	4.62724100	-0.49420600	0.87569400
Cl	2.67258800	1.46809600	2.04371900
C	2.87300800	-1.54741600	-0.70657300
C	2.15868500	-1.13653700	-1.84397600
C	3.32337000	-2.87614800	-0.63410100
C	1.88960200	-2.03617900	-2.87466100
H	1.81828400	-0.10972300	-1.91082900
C	3.04423300	-3.77615900	-1.65981400
H	3.87000800	-3.21591300	0.24238500
C	2.32723700	-3.35827200	-2.78474600
H	1.33768600	-1.70196500	-3.74835200
H	3.38356100	-4.80451400	-1.58148600
H	2.11824100	-4.05895700	-3.58728700
C	5.83841600	1.72292900	1.36994800
H	5.59606000	2.77245200	1.17100400
H	5.67777400	1.48272800	2.41999100
H	6.91549300	1.61452200	1.14326200
C	5.03270700	1.20545800	-0.97469900
H	4.24852000	1.97594900	-1.04202400
H	5.95882300	1.68535400	-1.31447000
H	4.74806800	0.38261100	-1.63165000
H	4.73196100	-0.64856400	1.95109900
H	5.17697200	-1.27736600	0.33773800

Cartesian coordinates of the optimized geometry for **52a** at B3LYP-D3BJ/6-31G(d),def2-TZVP level of theory (number of imaginary frequencies = 1):

C	1.98961400	0.07080100	-0.10989700
C	3.23048000	0.00599100	-0.43697500
Au	0.05581900	-0.09807700	0.24905700
C	4.49043200	0.80474700	-0.28984300
C	4.54271500	1.68695200	0.96742100
P	-2.22595100	-0.46915400	0.72478700
C	-2.55153400	0.08582800	2.50644500

C	-2.55511800	-2.31397300	0.39081800
C	3.15566300	-1.39523000	-1.04256600
C	2.70129100	-1.58051400	-2.35821300
C	3.58688900	-2.48989500	-0.27117800
C	2.73208600	-2.85479300	-2.92131500
H	2.33273600	-0.72725300	-2.91725200
C	3.62347500	-3.75514900	-0.84665100
H	3.89834100	-2.32814700	0.75602100
C	3.19604500	-3.93642600	-2.16834500
H	2.39128300	-3.00552500	-3.94062000
H	3.97235400	-4.60341900	-0.26646500
H	3.21721200	-4.92888300	-2.60814900
Cl	4.43592200	0.56156200	2.42953300
H	4.60237300	1.45138700	-1.17177700
H	5.33749300	0.11297600	-0.29440000
C	5.88805400	2.40790000	1.03791400
H	5.95931800	2.99615500	1.95587500
H	5.98584400	3.08770200	0.18294600
H	6.71907000	1.69786400	1.01606900
C	3.37469800	2.66402100	1.04683100
H	3.39662500	3.33139400	0.17692600
H	3.44328900	3.27102100	1.95305600
H	2.41061700	2.14551700	1.06047400
C	-2.04664000	1.54124200	2.58269300
H	-2.58427000	2.18987100	1.88350300
H	-2.21925800	1.92491000	3.59465200
H	-0.97447500	1.61458200	2.37245500
C	-1.72049100	-0.79480100	3.45632300
H	-0.66702200	-0.83966400	3.15989200
H	-1.76590100	-0.36809700	4.46470000
H	-2.10966700	-1.81509200	3.51494300
C	-4.02816200	0.06503400	2.93373200
H	-4.48291300	-0.92421800	2.84929500
H	-4.08493500	0.36233300	3.98730500
H	-4.62578000	0.77774500	2.36108100
C	-2.66661700	-2.45300400	-1.14037600
H	-2.70933800	-3.51791600	-1.39585900
H	-3.56529900	-1.97167100	-1.53339800
H	-1.79550900	-2.02258900	-1.64843200
C	-1.33311700	-3.12665700	0.86827500
H	-1.52055200	-4.18784400	0.66747900
H	-0.42130700	-2.84037000	0.33495100
H	-1.14718200	-3.01930400	1.93907500
C	-3.81390800	-2.88361700	1.06299200
H	-3.91329200	-3.93714000	0.77663200
H	-3.74951300	-2.84857700	2.15362000
H	-4.72928000	-2.37964300	0.74727000
C	-3.37882800	0.47682300	-0.34893700
C	-4.76126800	0.24213500	-0.21555900
C	-2.94012700	1.37941100	-1.34625700
C	-5.69373400	0.86841100	-1.03601400
H	-5.12089900	-0.44349300	0.53870500
C	-3.89632100	1.99735600	-2.16849100
C	-5.25798600	1.75123500	-2.02278200
H	-6.75157500	0.66377000	-0.90474100
H	-3.54969300	2.68883700	-2.93005400
H	-5.97182100	2.24677400	-2.67340000
C	-1.51853100	1.75191500	-1.61669900
C	-0.85147100	1.21483600	-2.72785600
C	-0.87523700	2.73679500	-0.85142400
C	0.43494500	1.64503100	-3.05910900
H	-1.35200300	0.46714200	-3.33594500
C	0.41107700	3.16671800	-1.18468600
H	-1.39454200	3.17914100	-0.00739100
C	1.06907100	2.62110400	-2.28856000
H	0.93153900	1.23164900	-3.93251600

H	0.88871800	3.93921300	-0.58985700
H	2.06344500	2.96635700	-2.55625000

Cartesian coordinates of the optimized geometry for **53a** at B3LYP-D3BJ/6-31G(d),def2-TZVP level of theory (number of imaginary frequencies = 1):

C	-1.84979700	-0.01938400	0.24632000
C	-3.06355200	0.24418200	0.40303900
Au	0.15402100	-0.27641100	0.26312400
C	-2.84685300	-0.06576600	-1.36887400
C	-3.11547200	-1.47782700	-1.88377800
P	2.46008800	-0.67549000	0.41006600
C	2.89067100	-2.04183700	-0.83152900
C	2.84086900	-1.10507400	2.22444300
C	-4.33763400	0.62063100	0.96073500
C	-4.93788500	1.83322800	0.58530000
C	-4.95478600	-0.21447100	1.90483800
C	-6.15269900	2.20501000	1.15266300
H	-4.44669500	2.47993200	-0.13619700
C	-6.16504700	0.17182500	2.47357100
H	-4.48868000	-1.15639900	2.17122200
C	-6.76524600	1.37556300	2.09615200
H	-6.61925000	3.14160200	0.86480800
H	-6.64532900	-0.47107000	3.20409700
H	-7.71319600	1.66809400	2.53686900
Cl	-4.62503400	-2.11776000	-1.05570400
H	-1.99517800	0.39388200	-1.87106100
H	-3.69568500	0.59550400	-1.55918700
C	-3.41822800	-1.36899600	-3.38302200
H	-3.67745900	-2.35150800	-3.78470600
H	-2.53222600	-1.00106800	-3.91431800
H	-4.25232400	-0.68822100	-3.57476300
C	-1.98435400	-2.46510900	-1.61779400
H	-1.05910200	-2.09688600	-2.07662500
H	-2.22806400	-3.43553700	-2.05704400
H	-1.81272400	-2.59947100	-0.54835500
C	2.36097400	-1.54496800	-2.19215000
H	2.83894300	-0.60673000	-2.49129300
H	2.58626900	-2.29568500	-2.95823900
H	1.27727400	-1.38980900	-2.17637800
C	2.14724900	-3.32797600	-0.43011700
H	1.07693200	-3.15281500	-0.27736200
H	2.25572500	-4.06639200	-1.23275300
H	2.55868600	-3.77204100	0.48052800
C	4.39322300	-2.33177200	-0.97862300
H	4.86083800	-2.63942600	-0.04140900
H	4.51704900	-3.15469900	-1.69221200
H	4.93496200	-1.47054300	-1.37577500
C	2.87737400	0.23826000	2.97952900
H	2.94303000	0.03769100	4.05488200
H	3.73711400	0.84884100	2.69332800
H	1.96663500	0.82328700	2.80542600
C	1.67340500	-1.94860100	2.77888300
H	1.88384400	-2.19046600	3.82697600
H	0.72628000	-1.40167900	2.74385700
H	1.54185500	-2.89013300	2.24069600
C	4.14893700	-1.87696300	2.46009300
H	4.26564300	-2.03665600	3.53828600
H	4.13641600	-2.86202500	1.98647300
H	5.03380800	-1.33504700	2.12131600
C	3.50669500	0.76552400	-0.04231600
C	4.90018900	0.66209800	0.13442200
C	2.97857400	1.97612900	-0.54737400
C	5.75859900	1.71190400	-0.17474300
H	5.32729600	-0.25336200	0.51903200

C	3.86114600	3.02361800	-0.85749500
C	5.23537100	2.90242300	-0.67663700
H	6.82735500	1.59743700	-0.02383500
H	3.44578600	3.94846600	-1.24531000
H	5.89069000	3.73166200	-0.92424300
C	1.53057700	2.26617100	-0.78187600
C	0.77657900	2.92295000	0.20280500
C	0.94655700	2.02300800	-2.03353400
C	-0.53698900	3.31874700	-0.05438600
H	1.23080800	3.13179500	1.16676700
C	-0.36508800	2.42721500	-2.29362400
H	1.53183200	1.53936400	-2.80934400
C	-1.10857600	3.07594600	-1.30476200
H	-1.10644600	3.82839600	0.71681700
H	-0.79339700	2.25966500	-3.27831000
H	-2.12140300	3.40865300	-1.51448900

Cartesian coordinates of the optimized geometry for **18b** at B3LYP-D3BJ/6-31G(d),def2-TZVP level of theory (number of imaginary frequencies = 0):

C	-1.28800500	-0.56437600	0.53400300
C	-2.06226100	-1.13672900	-0.44786200
C	-1.91201800	-0.04526200	1.76066800
C	-2.95614600	-0.71424500	2.43333600
C	-1.43730100	1.16789400	2.30153900
C	-2.02522900	1.72180600	3.43636500
C	-3.06600000	1.05301900	4.08526900
C	-3.51617900	-0.17544000	3.58811900
H	-4.30346900	-0.71581400	4.10501600
H	-3.30587000	-1.67333500	2.06780100
H	-0.61965200	1.67582000	1.80316700
H	-1.65671400	2.66601100	3.82644500
Au	0.76799800	-0.45533500	0.35578900
H	-3.50968600	1.47186500	4.98338900
P	3.12217800	-0.55152200	0.26452400
C	3.57828800	-2.03354400	-0.82912000
C	3.75362600	-0.66620400	2.05751100
C	2.86098500	-1.78515800	-2.17205400
H	1.77527100	-1.73302000	-2.05186000
H	3.08835100	-2.61258300	-2.85446400
H	3.20621300	-0.85950100	-2.64492500
C	5.07651400	-2.22427200	-1.11628900
H	5.19922300	-3.14388600	-1.70067300
H	5.67655600	-2.33191600	-0.21030000
H	5.48262400	-1.40451800	-1.71290800
C	3.01260600	-3.30817300	-0.17692600
H	1.95398500	-3.20046000	0.08173400
H	3.56533500	-3.58480300	0.72550300
H	3.10318400	-4.13985300	-0.88521500
C	2.78294200	-1.55568600	2.86190900
H	1.76643700	-1.15139500	2.85733500
H	3.12764500	-1.59849900	3.90182400
H	2.74037500	-2.57960100	2.48367200
C	5.17627400	-1.21957300	2.22889700
H	5.25563400	-2.25703600	1.89339200
H	5.43022400	-1.20281500	3.29547200
H	5.92999700	-0.62255100	1.71191800
C	3.67706900	0.76830100	2.61709000
H	2.68058900	1.20216800	2.47264500
H	4.41089600	1.43086700	2.15130200
H	3.87660400	0.74025200	3.69455600
C	3.96467500	0.91010100	-0.47467000
C	5.37261300	0.92538800	-0.50985700
C	3.26976600	2.05267900	-0.93656500
C	6.08671400	2.02135700	-0.98335600

H	5.92728700	0.06901600	-0.15484600
C	4.00810700	3.15322200	-1.40394500
C	5.39872100	3.14664200	-1.43324300
H	7.17185700	1.99438000	-0.99496600
H	3.46417100	4.02366800	-1.75736100
H	5.93862800	4.01228100	-1.80449000
C	1.78661200	2.22084700	-0.96442400
C	1.18335400	3.13177700	-0.08453000
C	0.99286700	1.58995100	-1.93405700
C	-0.18190500	3.40507600	-0.17139900
H	1.79535600	3.63063200	0.66121500
C	-0.37317000	1.86724700	-2.02224000
H	1.45148000	0.89700000	-2.63149900
C	-0.96181400	2.77559700	-1.14267600
H	-0.63434600	4.11685900	0.51341300
H	-0.97071400	1.36704500	-2.77819700
H	-2.01860500	3.00387700	-1.21958100
C	-3.95527500	0.03997300	-0.60973500
C	-3.52730700	-1.16912700	-0.48041300
C	-4.26703900	1.37732400	-0.62988100
C	-4.50407200	2.04534800	-1.86858100
C	-4.31467900	2.10366500	0.59878500
C	-4.76610100	3.40228300	-1.87002700
H	-4.46711500	1.47457900	-2.79002800
C	-4.57568100	3.46333300	0.56993600
H	-4.15094500	1.57872400	1.53127600
C	-4.79879400	4.10784700	-0.65462400
H	-4.94618500	3.92334400	-2.80417400
H	-4.61127400	4.02748700	1.49574500
H	-5.00603300	5.17379500	-0.66590200
Br	-4.60348100	-2.73909500	-0.39360600
Br	-1.29018800	-1.84779500	-2.05246000

Cartesian coordinates of the optimized geometry for **21b** at B3LYP-D3BJ/6-31G(d),def2-TZVP level of theory (number of imaginary frequencies = 0):

C	-0.45707700	2.41705900	-0.10444000
C	-1.35362800	1.55963100	-0.13809600
Au	0.59982200	0.36477900	-0.09435300
C	-2.63111700	0.95088400	-0.23449100
C	-3.16094200	0.09879800	0.67508000
C	-4.50696400	-0.47931800	0.72649800
C	-5.63140600	0.30753200	0.41709000
C	-4.69977200	-1.81855400	1.11223200
C	-6.90811900	-0.24552300	0.45648100
H	-5.50620800	1.35368500	0.16725600
C	-5.97646600	-2.36958700	1.13459600
H	-3.84503800	-2.41948200	1.39930900
C	-7.08462200	-1.58629300	0.80291700
H	-7.76707700	0.37628300	0.22475400
H	-6.10831700	-3.40791500	1.42230300
H	-8.08165400	-2.01491100	0.82972400
P	2.72293500	-0.54703500	0.23202000
C	3.94740000	0.57850200	-0.68665200
C	2.98252800	-0.63282500	2.11405900
C	0.46265400	3.50610800	-0.02788300
C	0.90762100	3.96171500	1.22839100
C	0.95606400	4.09426300	-1.20789500
C	1.83791300	4.99327700	1.29570500
H	0.52261700	3.49766800	2.13021100
C	1.88438500	5.12709100	-1.12572500
H	0.60760300	3.73359500	-2.17007300
C	2.32854600	5.57431400	0.12200900
H	2.17974800	5.34791100	2.26285800
H	2.26278100	5.58437300	-2.03437500

H	3.05484100	6.37897600	0.18042900
C	3.95088900	1.96472200	-0.01948500
H	4.50653300	2.66110300	-0.65756600
H	2.94410400	2.36851700	0.10813800
H	4.44650500	1.94692400	0.95501100
C	3.40317900	0.70240400	-2.12429600
H	2.42423300	1.19144500	-2.14822900
H	4.09893300	1.30779700	-2.71637400
H	3.31714000	-0.27567700	-2.60944600
C	5.38815200	0.04549000	-0.76341200
H	6.00594100	0.80476700	-1.25677500
H	5.82639800	-0.14130600	0.21963000
H	5.45400900	-0.86657600	-1.36003400
C	2.36941700	0.62461300	2.76458800
H	1.29250900	0.68607000	2.58062000
H	2.52237600	0.56584000	3.84835400
H	2.82772800	1.55112200	2.41250300
C	2.18614200	-1.86503600	2.58847200
H	2.17919200	-1.88039800	3.68428300
H	1.14484200	-1.82846800	2.24742600
H	2.62916900	-2.80017200	2.23754300
C	4.45017100	-0.75560800	2.55235700
H	5.04071000	0.12009000	2.26968700
H	4.48229700	-0.83139000	3.64545700
H	4.93323400	-1.65076800	2.15578900
C	2.95913100	-2.25405300	-0.39733100
C	4.21150900	-2.86953100	-0.20273600
C	1.91122200	-3.01622900	-0.96659200
C	4.44250600	-4.19313400	-0.56199200
H	5.02213200	-2.31345700	0.24510500
C	2.16419500	-4.35412800	-1.31552000
C	3.40962500	-4.94228600	-1.12288500
H	5.42107000	-4.63352900	-0.39906500
H	1.35731900	-4.92989500	-1.75744400
H	3.57106700	-5.97700600	-1.40876600
C	0.52296300	-2.53783600	-1.22280800
C	0.23611500	-1.59978100	-2.23204900
C	-0.54349600	-3.14288400	-0.54246100
C	-1.08765500	-1.28077500	-2.54810200
H	1.05036700	-1.16514300	-2.80324500
C	-1.86277000	-2.82508800	-0.86367300
H	-0.33050200	-3.87402500	0.23165200
C	-2.13909400	-1.89429600	-1.86449800
H	-1.29564600	-0.56202100	-3.33432700
H	-2.67746800	-3.31350600	-0.34032400
H	-3.16598800	-1.64722600	-2.11160300
Br	-1.99319400	-0.41219100	2.11895700
Br	-3.54373900	1.42312900	-1.85271500

Cartesian coordinates of the optimized geometry for **22b** at B3LYP-D3BJ/6-31G(d),def2-TZVP level of theory (number of imaginary frequencies = 0):

C	-1.29369600	-0.55179200	-0.68777000
C	-2.26202200	-0.93835300	0.20909800
Au	0.73122900	-0.49023200	-0.38906100
P	3.06872800	-0.59535600	-0.15666900
C	3.47840700	-1.50112700	1.45619800
C	3.72575900	-1.46029100	-1.72411200
C	2.74669800	-0.71560600	2.56283400
H	1.66449700	-0.71066500	2.41003600
H	2.95020100	-1.19204100	3.52922700
H	3.09655400	0.32015500	2.61960100
C	4.97423600	-1.55289500	1.80351600
H	5.09157900	-2.09947000	2.74677900
H	5.56881100	-2.07196100	1.04937600

H	5.39067800	-0.55341500	1.94980800
C	2.90150500	-2.92565800	1.38139700
H	1.84178100	-2.92037000	1.10485600
H	3.44438300	-3.55307100	0.66874800
H	2.98902100	-3.39700700	2.36722400
C	2.70141500	-2.53936100	-2.13878900
H	1.72659800	-2.10357200	-2.37559200
H	3.07267300	-3.04620000	-3.03719800
H	2.55396500	-3.29736700	-1.36575000
C	5.09854900	-2.14128100	-1.59665100
H	5.08486000	-2.95659500	-0.86889200
H	5.35732500	-2.57793300	-2.56845300
H	5.90401900	-1.45354900	-1.33368200
C	3.76666600	-0.38224900	-2.82439100
H	2.80147200	0.12911700	-2.91882100
H	4.53668600	0.37049300	-2.63940400
H	3.98242400	-0.86353900	-3.78519300
C	3.93031400	1.02677100	-0.01420500
C	5.33542100	1.04516600	-0.10288100
C	3.25832500	2.24569200	0.23844200
C	6.06965000	2.21663600	0.04896900
H	5.87231700	0.12492900	-0.28432300
C	4.01750400	3.41666900	0.40465200
C	5.40517400	3.41348700	0.30991800
H	7.15190900	2.18969200	-0.03046800
H	3.49118600	4.34574400	0.60015100
H	5.96078100	4.33752800	0.43655800
C	1.77932800	2.42912300	0.35019600
C	0.97737300	2.54305300	-0.79603100
C	1.19653900	2.64618100	1.60620600
C	-0.37798700	2.86252500	-0.68542200
H	1.42596300	2.40581600	-1.77524000
C	-0.15846700	2.96214700	1.71598200
H	1.81412300	2.57868700	2.49669900
C	-0.94952900	3.06927800	0.57037000
H	-0.98278200	2.94590200	-1.58238100
H	-0.59323600	3.13270800	2.69694600
H	-2.00031900	3.32567900	0.65659400
C	-4.13425100	0.21882600	-0.04148100
C	-3.70706600	-0.98563000	-0.16177100
C	-1.93021700	-1.26718400	1.61537400
C	-1.16868500	-0.37814600	2.39107600
C	-2.38803000	-2.46281600	2.19314100
C	-0.85580100	-0.68869200	3.71209800
H	-0.83545500	0.55577100	1.95514900
C	-2.06538600	-2.77221000	3.51325000
H	-2.97989700	-3.15533000	1.60422500
C	-1.29876500	-1.88803600	4.27560000
H	-0.26767900	0.00807800	4.30237100
H	-2.41225500	-3.70542900	3.94616700
H	-1.05229900	-2.13057400	5.30488700
C	-4.39532400	1.56673500	0.08374600
C	-4.76361800	2.11159500	1.34742900
C	-4.25909500	2.41971500	-1.05016800
C	-4.97735000	3.47366500	1.46546200
H	-4.85878400	1.44749400	2.19956800
C	-4.48292400	3.77892900	-0.91034600
H	-3.97359500	1.98234900	-1.99934700
C	-4.84014000	4.30275300	0.34073400
H	-5.25453700	3.90118100	2.42318100
H	-4.38446000	4.43805800	-1.76633600
H	-5.01630800	5.36950200	0.44131700
Br	-1.85553900	-0.05067900	-2.46210700
Br	-4.66695300	-2.52211700	-0.71682200

Cartesian coordinates of the optimized geometry for **23b** at B3LYP-D3BJ/6-31G(d),def2-TZVP level of theory (number of imaginary frequencies = 0):

C	1.09012900	0.07790700	-0.58009500
C	1.93278600	1.02925000	-0.11101300
Au	-0.92995800	-0.10958800	-0.64934300
P	-3.27078000	0.00964400	-0.61204800
C	-3.71206900	1.83512800	-0.34253900
C	-3.94658400	-0.73406800	-2.22833000
C	-2.93829600	2.26910100	0.91952800
H	-1.85634000	2.16734100	0.79939500
H	-3.15291100	3.32489700	1.12069700
H	-3.25071500	1.69174300	1.79545000
C	-5.20657700	2.10674300	-0.10917500
H	-5.34650700	3.18796300	0.00669100
H	-5.83382700	1.78372900	-0.94238800
H	-5.56620600	1.63080300	0.80596200
C	-3.21441900	2.64749600	-1.55086000
H	-2.15971600	2.45112100	-1.76668600
H	-3.80093400	2.44582300	-2.45161900
H	-3.31526500	3.71528800	-1.32461600
C	-2.97458600	-0.36154700	-3.36777200
H	-1.96932100	-0.75294000	-3.18493900
H	-3.34451300	-0.79997300	-4.30189800
H	-2.89492400	0.71798900	-3.51550500
C	-5.35907200	-0.28170100	-2.63153800
H	-5.40659200	0.79301400	-2.82499200
H	-5.63370900	-0.79224700	-3.56207400
H	-6.11912600	-0.53851000	-1.89134000
C	-3.91519900	-2.26325500	-2.03937400
H	-2.92424500	-2.60833200	-1.72130300
H	-4.64776400	-2.60293900	-1.30332000
H	-4.14413800	-2.74403300	-2.99733700
C	-4.05211700	-0.90479400	0.77906900
C	-5.45757900	-0.98471600	0.82183000
C	-3.30864000	-1.55137200	1.79370000
C	-6.12363500	-1.68013500	1.82545300
H	-6.04677800	-0.49554000	0.05880600
C	-3.99887300	-2.24870400	2.79893200
C	-5.38817200	-2.31850800	2.82270600
H	-7.20832200	-1.72158400	1.82521200
H	-3.41904300	-2.74105200	3.57335300
H	-5.89088800	-2.86634300	3.61371000
C	-1.81763500	-1.57316200	1.90771000
C	-1.09730200	-2.69003200	1.45641800
C	-1.13407900	-0.55975300	2.59459800
C	0.27746400	-2.78445900	1.67823600
H	-1.62528600	-3.48958900	0.94507000
C	0.24089400	-0.65562900	2.81444100
H	-1.68443500	0.29821600	2.96625800
C	0.95022100	-1.76674500	2.35778600
H	0.81777800	-3.66157300	1.33340000
H	0.75247900	0.13389900	3.35564400
H	2.01836000	-1.84352600	2.54039400
C	3.35245400	0.73135400	0.02816100
C	3.83086100	-0.48019100	-0.34040200
C	5.12768600	-1.13655400	-0.39663700
C	5.28049300	-2.45695100	0.06345800
C	6.24057100	-0.45069200	-0.91555500
C	6.52591400	-3.07416900	0.01758500
H	4.42468100	-2.98922700	0.46931000
C	7.48461800	-1.07415900	-0.95163000
H	6.12394100	0.56007300	-1.28852900
C	7.63065700	-2.38301900	-0.48715000
H	6.63677300	-4.09139300	0.37930400
H	8.33943200	-0.53944600	-1.35327500

H	8.60228900	-2.86569700	-0.52167400
C	1.30289400	2.32172200	0.27388900
C	1.26709800	2.72992800	1.61457200
C	0.68074900	3.10871300	-0.70505200
C	0.61733600	3.91060000	1.96818800
H	1.74664200	2.12174100	2.37405900
C	0.03397800	4.29160200	-0.34762100
H	0.71466000	2.79066600	-1.74214600
C	-0.00036400	4.69273500	0.98852400
H	0.59313100	4.22094400	3.00829100
H	-0.43932700	4.89888900	-1.11300100
H	-0.50279500	5.61399600	1.26736300
Br	2.29566200	-1.50712700	-1.00071700
Br	4.52182900	2.02372300	0.75862800

Cartesian coordinates of the optimized geometry for **24b** at B3LYP-D3BJ/6-31G(d),def2-TZVP level of theory (number of imaginary frequencies = 1):

C	-0.67396000	0.94710700	-0.06666100
C	-1.92395800	0.78329000	-0.17009400
Au	1.31317400	0.60908900	0.03101100
C	-3.21850900	0.21149800	-0.35255600
C	-3.90914600	-0.46114500	0.59384800
C	-5.23412100	-1.08109000	0.47566600
C	-6.31708200	-0.34208700	-0.02877000
C	-5.43340400	-2.41172100	0.87979000
C	-7.57280400	-0.93286100	-0.13988900
H	-6.17588500	0.69354200	-0.31585800
C	-6.68648900	-3.00144100	0.74803400
H	-4.60411000	-2.97831900	1.29027600
C	-7.75856000	-2.26360100	0.23966300
H	-8.40732300	-0.35157400	-0.51919700
H	-6.82906500	-4.03436000	1.04942100
H	-8.73805000	-2.72266300	0.14764600
P	3.62060300	0.20982600	0.17179600
C	4.48353000	1.22940400	-1.17236700
C	4.16424600	0.62866500	1.94785300
C	-1.56991800	2.39259900	-0.03436500
C	-1.82133300	2.99462800	1.21865500
C	-1.59886900	3.16319900	-1.21663500
C	-2.09539600	4.35338200	1.28125400
H	-1.80175500	2.37850300	2.11100000
C	-1.87100500	4.52262800	-1.14142200
H	-1.40839000	2.67675200	-2.16712400
C	-2.11785500	5.11336800	0.10401700
H	-2.29101400	4.82692400	2.23779500
H	-1.89378400	5.12520900	-2.04351400
H	-2.33083100	6.17677500	0.15846100
C	4.25451200	2.72309500	-0.88223800
H	4.59774900	3.30890000	-1.74271900
H	3.19499600	2.95050400	-0.72187900
H	4.81855400	3.06280800	-0.00903900
C	3.78090200	0.84982700	-2.49181300
H	2.71402400	1.09453700	-2.47239400
H	4.24403200	1.40665500	-3.31457400
H	3.88635500	-0.21777800	-2.70930300
C	5.98740500	0.95285800	-1.33151400
H	6.37909900	1.61650800	-2.11131900
H	6.55581300	1.14918000	-0.42018500
H	6.17829300	-0.07428500	-1.65023300
C	3.37669600	1.87076100	2.41529300
H	2.29775400	1.68892700	2.41093300
H	3.67598700	2.10896100	3.44261800
H	3.57452900	2.75048900	1.79856600
C	3.74166000	-0.57292400	2.81574200

H	3.89288500	-0.31831700	3.87102300
H	2.68092400	-0.81399300	2.67927100
H	4.32753700	-1.46794900	2.59333000
C	5.66386300	0.90977000	2.13320900
H	5.98924200	1.79121400	1.57462500
H	5.84962200	1.11080700	3.19482600
H	6.29526600	0.06433400	1.85415200
C	4.08766400	-1.54098300	-0.13659500
C	5.43194700	-1.91732400	0.05207500
C	3.15671300	-2.53241300	-0.52538000
C	5.86012900	-3.22766500	-0.13161600
H	6.16149200	-1.17652800	0.34767800
C	3.60888600	-3.85011300	-0.70620200
C	4.94066800	-4.20315500	-0.51387700
H	6.90398400	-3.48141300	0.02409600
H	2.88691600	-4.60340700	-1.00540200
H	5.25675400	-5.23118000	-0.66160800
C	1.69671500	-2.31792700	-0.76788600
C	1.22778300	-1.95555800	-2.03955000
C	0.76538300	-2.62217900	0.23687900
C	-0.14263300	-1.89805500	-2.29932300
H	1.94125800	-1.73648600	-2.82769200
C	-0.60502500	-2.56477900	-0.02485100
H	1.12234800	-2.91720000	1.21926300
C	-1.06117300	-2.21067400	-1.29561300
H	-0.49265400	-1.62242300	-3.28959100
H	-1.31264300	-2.79848500	0.76417000
H	-2.12508800	-2.17963000	-1.50610400
Br	-3.05982600	-0.66091500	2.30524200
Br	-3.87644000	0.40516300	-2.14366500

Cartesian coordinates of the optimized geometry for **32b** at B3LYP-D3BJ/6-31G(d),def2-TZVP level of theory (number of imaginary frequencies = 1):

C	-1.34821500	-0.47490000	0.51594100
C	-1.90741700	-0.78389900	-0.59662200
C	-1.81760900	-0.15120900	1.86355200
C	-2.80598000	-0.92695800	2.49158000
C	-1.28350300	0.95879800	2.53986700
C	-1.76999800	1.31737400	3.79452600
C	-2.77198300	0.55783500	4.40360500
C	-3.27780400	-0.57050000	3.75338700
H	-4.04158900	-1.17749700	4.22986500
H	-3.19474200	-1.80786400	1.99582700
H	-0.51013800	1.54610600	2.05971600
H	-1.36090900	2.18647400	4.30106900
Au	0.76312900	-0.48756400	0.21396400
H	-3.14586100	0.83374100	5.38476800
P	3.08948700	-0.64841000	0.17234800
C	3.54339200	-1.98586500	-1.09259200
C	3.64018600	-1.01761500	1.95678600
C	2.85043000	-1.56695000	-2.40527200
H	1.76193400	-1.52739000	-2.29731300
H	3.08965200	-2.30145600	-3.18293600
H	3.20142500	-0.58893000	-2.75047000
C	5.04818300	-2.13766600	-1.37068600
H	5.18498000	-2.96658700	-2.07497600
H	5.62595500	-2.37275800	-0.47439900
H	5.46665800	-1.24234600	-1.83549600
C	2.96474600	-3.32973900	-0.61607000
H	1.90087800	-3.25200700	-0.36749300
H	3.49803800	-3.71960900	0.25541100
H	3.06910600	-4.06588700	-1.42134900
C	2.61704000	-1.97541300	2.60216600
H	1.61503000	-1.53685800	2.63157000

H	2.92879600	-2.17452400	3.63401100
H	2.55233700	-2.93454900	2.08374900
C	5.04091800	-1.63481300	2.09422700
H	5.10621600	-2.61711600	1.61886600
H	5.25460000	-1.77505200	3.16028400
H	5.82898000	-0.99504800	1.69267200
C	3.58303100	0.33055300	2.70219700
H	2.60304800	0.80922400	2.59136300
H	4.34804400	1.02787900	2.35231900
H	3.74697700	0.15015500	3.77068700
C	3.96753100	0.88454800	-0.33646200
C	5.37583400	0.88876900	-0.29705100
C	3.29882600	2.08011000	-0.68957500
C	6.11525300	2.02955700	-0.59113400
H	5.90964800	-0.01132700	-0.02757800
C	4.06320800	3.22340600	-0.97601800
C	5.45349200	3.20843900	-0.93041100
H	7.19942100	1.99513400	-0.55064800
H	3.54027300	4.13534900	-1.24649900
H	6.01422300	4.10924600	-1.15994000
C	1.81896500	2.25373800	-0.79018600
C	1.13254700	2.97332600	0.19924900
C	1.11779900	1.82967300	-1.92907500
C	-0.22961500	3.24599900	0.06045400
H	1.67599800	3.32224900	1.07274900
C	-0.24174900	2.11217300	-2.06903700
H	1.64702500	1.29639000	-2.71225300
C	-0.92061600	2.81424600	-1.07229500
H	-0.75260300	3.80305000	0.83218800
H	-0.76913400	1.78631300	-2.95972100
H	-1.97450000	3.03787400	-1.18393100
C	-4.25836500	0.07820400	-0.42391800
C	-4.08636000	-1.13952500	-0.44155300
C	-4.32939100	1.48735000	-0.39434700
C	-4.55816600	2.20526700	-1.58767000
C	-4.16044000	2.17730400	0.82539400
C	-4.61492600	3.59395100	-1.55636800
H	-4.68908300	1.66287200	-2.51801400
C	-4.20970800	3.56613100	0.83864200
H	-3.99052400	1.61736400	1.73698700
C	-4.43636800	4.27424500	-0.34661100
H	-4.79556000	4.14865800	-2.47143900
H	-4.07828100	4.09939300	1.77479200
H	-4.47818600	5.35898000	-0.32777200
Br	-4.42890000	-2.91172800	-0.43525800
Br	-1.66357400	-1.20231900	-2.36122100

Cartesian coordinates of the optimized geometry for **33b** at B3LYP-D3BJ/6-31G(d),def2-TZVP level of theory (number of imaginary frequencies = 1):

C	-1.34832700	-0.48113000	-0.90798800
C	-1.93195800	-0.65328900	0.23395900
Au	0.72178900	-0.49471700	-0.58279500
P	2.97988700	-0.64395400	0.00715500
C	3.00062700	-1.45137400	1.72913400
C	3.91276900	-1.61366200	-1.33390200
C	2.07794500	-0.57781500	2.60341900
H	1.05045100	-0.56967300	2.23231000
H	2.06397500	-0.98350100	3.62171800
H	2.43354000	0.45633500	2.65775200
C	4.37799400	-1.52539500	2.40821100
H	4.26173200	-2.05469800	3.36137000
H	5.11734800	-2.07223900	1.81987300
H	4.77232600	-0.53230300	2.63314500
C	2.40257400	-2.86482100	1.61521700

H	1.43501500	-2.85791600	1.10224700
H	3.07080100	-3.55336400	1.09014500
H	2.24480100	-3.26453900	2.62371200
C	2.98018000	-2.72266500	-1.86346300
H	2.05994900	-2.30788800	-2.28619100
H	3.50086100	-3.27072800	-2.65728200
H	2.70291200	-3.44172800	-1.08911300
C	5.23274100	-2.25840300	-0.88226400
H	5.07676000	-3.01612400	-0.11007900
H	5.68575600	-2.76139700	-1.74462000
H	5.96079700	-1.53024100	-0.51876700
C	4.18022000	-0.61293100	-2.47451300
H	3.25267400	-0.14170800	-2.81796200
H	4.87856800	0.17398100	-2.17808400
H	4.61314500	-1.15165300	-3.32509400
C	3.82695300	0.97373700	0.22884800
C	5.18728000	0.97289400	0.59240300
C	3.15588500	2.21621400	0.12924100
C	5.87019700	2.14951900	0.88383100
H	5.72500300	0.03747700	0.66063600
C	3.85508300	3.39064300	0.45259200
C	5.19403900	3.36722900	0.83026800
H	6.92003000	2.11095100	1.15704500
H	3.33062600	4.33797200	0.37553900
H	5.70824000	4.29402200	1.06533100
C	1.76035200	2.40589300	-0.36496800
C	1.48960200	2.30743600	-1.73976700
C	0.73449100	2.80954100	0.50051700
C	0.21835400	2.59510700	-2.23539600
H	2.28896600	2.02568500	-2.41789000
C	-0.54025600	3.09327200	0.00304000
H	0.94194400	2.89975700	1.56294000
C	-0.79871900	2.98689900	-1.36350400
H	0.02395600	2.51494400	-3.30016200
H	-1.33029200	3.41443600	0.67660100
H	-1.78676000	3.21346800	-1.74665700
C	-4.23684900	0.09795200	0.14163600
C	-4.06845200	-1.10575800	-0.03548500
C	-1.76347800	-0.74569600	1.65638200
C	-1.57213400	0.42735100	2.41337500
C	-1.75884400	-2.00022600	2.29631000
C	-1.37399000	0.34052900	3.78775400
H	-1.55401400	1.38197400	1.90349100
C	-1.55591300	-2.07605300	3.66975900
H	-1.90589900	-2.89772100	1.70569200
C	-1.36691100	-0.90804400	4.41634400
H	-1.21667400	1.24378300	4.36909400
H	-1.54390000	-3.04359100	4.16142100
H	-1.20904200	-0.97258600	5.48861900
C	-4.32088800	1.48940400	0.37274300
C	-4.42153000	1.97973800	1.69178400
C	-4.27348900	2.38854100	-0.71272100
C	-4.46027400	3.35207000	1.91511200
H	-4.46476600	1.27889800	2.51798800
C	-4.32260400	3.75697300	-0.47500900
H	-4.19077400	1.99603200	-1.72002700
C	-4.40974900	4.23966500	0.83566100
H	-4.53947000	3.73190000	2.92875300
H	-4.29334800	4.45163300	-1.30854800
H	-4.44727700	5.30984300	1.01452700
Br	-2.10092000	-0.28532000	-2.61228800
Br	-4.29779800	-2.86232500	-0.35020800

Cartesian coordinates of the optimized geometry for **44b** at B3LYP-D3BJ/6-31G(d),def2-TZVP level of theory (number of imaginary frequencies = 1):

C	0.87642500	0.75902100	0.66429100
C	2.12727800	1.09161100	0.52825900
Au	-0.97693700	0.17234100	0.61542800
P	-3.05803900	-0.96604800	0.56947600
C	-2.72400800	-2.68675900	-0.15283000
C	-3.78108300	-0.96196200	2.32622000
C	-1.99389300	-2.43966200	-1.48912800
H	-1.02474800	-1.95344000	-1.34286200
H	-1.81578200	-3.40470200	-1.97735100
H	-2.59260200	-1.82593000	-2.16984600
C	-3.98774100	-3.51482900	-0.43619800
H	-3.67728800	-4.49645900	-0.81247900
H	-4.59577600	-3.68398700	0.45466200
H	-4.61081900	-3.05211500	-1.20463800
C	-1.79624400	-3.45571800	0.80400100
H	-0.90157300	-2.87836300	1.06045100
H	-2.30436900	-3.74014000	1.72964500
H	-1.46884000	-4.37888900	0.31241100
C	-2.62103400	-1.13655700	3.32871400
H	-1.89333600	-0.32281200	3.25279100
H	-3.03175800	-1.12564800	4.34475900
H	-2.08851700	-2.08112200	3.19596500
C	-4.83237500	-2.04890300	2.60212500
H	-4.41379200	-3.05536900	2.52222400
H	-5.19349200	-1.92740600	3.62992300
H	-5.70325500	-1.97768500	1.94747400
C	-4.40030500	0.43453800	2.52925000
H	-3.67381500	1.23018900	2.32646300
H	-5.27307800	0.59623700	1.89187800
H	-4.71603500	0.53339600	3.57393400
C	-4.28058300	-0.18462800	-0.55514700
C	-5.58199000	-0.71852900	-0.61356400
C	-3.95226700	0.89861500	-1.40346700
C	-6.53730200	-0.21866100	-1.49260700
H	-5.85579900	-1.54387200	0.02927400
C	-4.92422000	1.37671600	-2.29695600
C	-6.20282300	0.82989500	-2.34839800
H	-7.53261200	-0.65121100	-1.51093200
H	-4.66253500	2.20557800	-2.94710500
H	-6.93431800	1.22584100	-3.04591600
C	-2.64598100	1.62304300	-1.41125700
C	-2.39090900	2.62679700	-0.45871200
C	-1.71243400	1.41026300	-2.43580500
C	-1.23017800	3.40319100	-0.54005400
H	-3.12459100	2.81916700	0.31863800
C	-0.54968600	2.18128600	-2.50740200
H	-1.90534200	0.64258100	-3.17909700
C	-0.31031100	3.18454900	-1.56718500
H	-1.04975700	4.18242600	0.19346700
H	0.16582000	2.00017100	-3.30364900
H	0.58816800	3.78899500	-1.62768800
C	3.17646200	0.05031600	0.44043700
C	3.26316100	-0.93845000	-0.47183400
C	4.36447100	-1.90056900	-0.64451900
C	4.10024500	-3.27797500	-0.70218600
C	5.68536300	-1.44430600	-0.77647100
C	5.14551200	-4.18424300	-0.85404000
H	3.07743700	-3.63139800	-0.62152600
C	6.72502400	-2.35499200	-0.94537600
H	5.89171800	-0.37999700	-0.75590000
C	6.45902600	-3.72519200	-0.97738500
H	4.93472100	-5.24869500	-0.88327700
H	7.74255800	-1.99339000	-1.05554900
H	7.27191600	-4.43331500	-1.10548400

C	2.42340600	2.54726200	0.40679000
C	1.85783700	3.46895700	1.29702100
C	3.21650600	2.98430900	-0.66357700
C	2.08100100	4.83303500	1.10919800
H	1.26925500	3.11231200	2.13579300
C	3.44440400	4.34644500	-0.83492600
H	3.64367600	2.25871300	-1.34868900
C	2.86934000	5.27197500	0.04435800
H	1.65102900	5.55016500	1.80148700
H	4.06889300	4.68950300	-1.65394500
H	3.04805600	6.33342900	-0.09629900
Br	1.84608800	-1.14016700	-1.76773400
Br	4.48473000	0.24627200	1.81773900

Cartesian coordinates of the optimized geometry for **45b** at B3LYP-D3BJ/6-31G(d),def2-TZVP level of theory (number of imaginary frequencies = 0):

C	-1.27156600	-0.89244800	0.58823200
C	-2.51984100	-1.20022400	0.47192700
Au	0.62564400	-0.28394000	0.58408000
P	2.78051100	0.64132900	0.50100100
C	2.67106200	2.16769500	-0.62084000
C	3.33902600	1.00689800	2.28122600
C	2.06657900	1.66276700	-1.94720300
H	1.07318500	1.22491400	-1.80761400
H	1.97687700	2.50730800	-2.64095200
H	2.70568000	0.91028900	-2.41965100
C	4.02005900	2.83597000	-0.92974900
H	3.83603500	3.71133700	-1.56382300
H	4.53643200	3.18466400	-0.03344200
H	4.68577500	2.16746500	-1.48029000
C	1.71476300	3.18735300	0.02265700
H	0.75279500	2.73781600	0.29064300
H	2.14421000	3.64453300	0.91839000
H	1.51866300	3.99365800	-0.69394900
C	2.10570000	1.47244400	3.08449700
H	1.32680200	0.70464600	3.11329500
H	2.41650100	1.67629600	4.11564300
H	1.66331400	2.38754900	2.68367000
C	4.44018200	2.06928300	2.42695000
H	4.10941300	3.05293800	2.08354600
H	4.69061300	2.16367800	3.48998700
H	5.36225700	1.80752000	1.90442800
C	3.82098400	-0.33805000	2.85944700
H	3.06100400	-1.11999500	2.74574100
H	4.74286300	-0.68453100	2.38615400
H	4.01180000	-0.21435900	3.93147500
C	4.03838800	-0.45100700	-0.27398700
C	5.38375200	-0.03547500	-0.26688300
C	3.71255600	-1.67876500	-0.89724700
C	6.38812700	-0.79480100	-0.85811300
H	5.65574400	0.89919200	0.20391800
C	4.73858300	-2.42565400	-1.49814800
C	6.06248700	-1.99738600	-1.48342600
H	7.41555200	-0.44569800	-0.83136800
H	4.47836300	-3.36504000	-1.97576800
H	6.83326700	-2.60048200	-1.95315500
C	2.34501100	-2.27870100	-0.96658400
C	1.87943900	-3.10251200	0.07031300
C	1.55735100	-2.12611900	-2.11632900
C	0.64447800	-3.74850100	-0.03806000
H	2.49666400	-3.24759500	0.95199600
C	0.32327600	-2.76980600	-2.22080700
H	1.91943600	-1.50553200	-2.93028300
C	-0.13723800	-3.58136500	-1.18298000

H	0.30572700	-4.39287300	0.76819400
H	-0.27431700	-2.64621400	-3.11869700
H	-1.09649800	-4.08221400	-1.26771000
C	-2.67391700	0.29328800	0.38032400
C	-2.52554800	1.03787100	-0.77106200
C	-2.71576400	2.47744200	-0.93833800
C	-1.74113600	3.23174300	-1.62294700
C	-3.86183500	3.12632000	-0.44059600
C	-1.88774100	4.60653400	-1.75878000
H	-0.86645400	2.73580500	-2.02717500
C	-4.01811400	4.49764000	-0.61589400
H	-4.63715700	2.55455700	0.05276200
C	-3.02829100	5.24257800	-1.26019700
H	-1.12006800	5.18165500	-2.26676500
H	-4.91554200	4.98448300	-0.24824700
H	-3.14958000	6.31433400	-1.38298800
C	-3.53375300	-2.23596800	0.48723300
C	-3.18163500	-3.54816900	0.84488500
C	-4.85497200	-1.93258400	0.13385100
C	-4.14875300	-4.54730400	0.83388100
H	-2.15926300	-3.76690800	1.13496600
C	-5.81754700	-2.93893600	0.12729000
H	-5.11950600	-0.91467100	-0.13514000
C	-5.46628500	-4.24517500	0.47442100
H	-3.87940800	-5.56149500	1.11191300
H	-6.84099200	-2.70445000	-0.14752500
H	-6.21870100	-5.02761400	0.47007700
Br	-3.23389300	1.13691000	2.01893500
Br	-1.95988500	0.12780700	-2.34360200

Cartesian coordinates of the optimized geometry for **46b** at B3LYP-D3BJ/6-31G(d),def2-TZVP level of theory (number of imaginary frequencies = 1):

C	1.06030500	0.46470200	0.50134400
C	2.00164500	1.32544800	0.50293500
Au	-0.87097700	-0.15736200	0.38360600
P	-3.08316300	-0.92333600	0.27531300
C	-3.15289700	-2.37177200	-0.94422100
C	-3.62079300	-1.36375100	2.04810700
C	-2.52671200	-1.84313500	-2.25063300
H	-1.48964400	-1.52330200	-2.10700300
H	-2.53781900	-2.64353600	-2.99959700
H	-3.09600100	-0.99983000	-2.65427400
C	-4.56743000	-2.88358000	-1.26052800
H	-4.48184800	-3.73234600	-1.94927000
H	-5.10092500	-3.23420400	-0.37486000
H	-5.17229400	-2.12038300	-1.75545100
C	-2.29000100	-3.52108200	-0.39442400
H	-1.28527400	-3.18187200	-0.11975700
H	-2.74612600	-3.99622200	0.47852300
H	-2.18623200	-4.28953000	-1.16923100
C	-2.41767500	-2.00318800	2.77334700
H	-1.56363500	-1.32106100	2.82114000
H	-2.71634000	-2.24376600	3.80029600
H	-2.08448000	-2.92897000	2.29874900
C	-4.81723700	-2.32207700	2.16076200
H	-4.59915100	-3.30392400	1.73266500
H	-5.03998200	-2.47339200	3.22350100
H	-5.72509000	-1.93092800	1.69779000
C	-3.95076400	-0.02481500	2.73659600
H	-3.12387800	0.68899100	2.64371300
H	-4.85141500	0.43874800	2.32692900
H	-4.11502000	-0.20719900	3.80476000
C	-4.27055000	0.33197600	-0.35284500
C	-5.64561900	0.02860200	-0.32199400

C	-3.87006800	1.60251700	-0.82868500
C	-6.60863800	0.94145100	-0.73868400
H	-5.97484800	-0.93721700	0.03482100
C	-4.85761000	2.51161800	-1.24298500
C	-6.21167900	2.19505900	-1.20121500
H	-7.65974200	0.67325600	-0.69935100
H	-4.54004600	3.48382100	-1.60668400
H	-6.94941300	2.92101700	-1.52880400
C	-2.46127600	2.09061300	-0.94481200
C	-1.92619900	2.94047400	0.03562000
C	-1.70916600	1.83235000	-2.10083200
C	-0.66345100	3.51157300	-0.13388300
H	-2.51089500	3.15843300	0.92449900
C	-0.44928000	2.40744400	-2.26993400
H	-2.12434800	1.19370500	-2.87380700
C	0.07516400	3.25040100	-1.28916600
H	-0.26062100	4.16788100	0.63190700
H	0.11704600	2.20544400	-3.17409500
H	1.05225900	3.70319900	-1.42413600
C	2.40492400	-0.31231200	0.52118200
C	3.00163400	-0.83473100	-0.61501800
C	4.18376300	-1.67514900	-0.70255700
C	4.25787900	-2.73693100	-1.62738200
C	5.29864000	-1.39676100	0.11715500
C	5.39917300	-3.52532700	-1.69639900
H	3.41084600	-2.94699600	-2.27041100
C	6.44835700	-2.17004100	0.01617600
H	5.26743200	-0.56011100	0.80409300
C	6.49765300	-3.24232900	-0.87892900
H	5.43788200	-4.35561600	-2.39397600
H	7.30794400	-1.93594000	0.63578600
H	7.39355500	-3.85182200	-0.94532800
C	2.86691000	2.45598300	0.62171600
C	2.70905900	3.31936600	1.72197100
C	3.84819700	2.71934200	-0.35220600
C	3.51502500	4.44921700	1.82904800
H	1.95756200	3.09459000	2.47121700
C	4.65345900	3.84503900	-0.22809200
H	3.96129000	2.04478100	-1.19461800
C	4.48573000	4.71078100	0.85877900
H	3.39274700	5.12088800	2.67268800
H	5.41058800	4.05304800	-0.97729300
H	5.11643100	5.58967700	0.94971100
Br	2.88514500	-1.02631500	2.25692200
Br	2.17260600	-0.39595600	-2.27364500

Cartesian coordinates of the optimized geometry for **18c** at B3LYP-D3BJ/6-31G(d),def2-TZVP level of theory (number of imaginary frequencies = 0):

C	-1.48839400	-1.02697200	0.08999900
C	-2.15465700	-1.38342800	-1.06369600
C	-2.20667800	-0.95478600	1.36865300
Cl	-1.31945200	-1.49582300	-2.60649900
C	-3.18517500	-1.89424800	1.75822500
C	-1.89776400	0.09659500	2.25769900
C	-2.58532100	0.23942100	3.46045900
C	-3.56048200	-0.69165100	3.82604000
C	-3.84395200	-1.76825700	2.97760400
H	-4.57698700	-2.51352400	3.27107300
H	-3.40002900	-2.74497000	1.12116300
H	-1.13066700	0.80858300	1.97540600
H	-2.34538900	1.06630600	4.12231900
Au	0.54235700	-0.64870800	0.07617500
H	-4.08091100	-0.59504300	4.77403100
P	2.89665000	-0.49156000	0.09862200

C	3.52872900	-1.57739100	-1.32385500
C	3.48866500	-1.02538800	1.82719500
C	2.81247800	-1.05157900	-2.58516300
H	1.72532000	-1.14298900	-2.50528300
H	3.14066400	-1.63826300	-3.45115500
H	3.06194500	-0.00289400	-2.77946100
C	5.04361200	-1.53737700	-1.58240300
H	5.27401800	-2.25292600	-2.38059500
H	5.63479200	-1.82222900	-0.70949200
H	5.37104400	-0.55333700	-1.92468200
C	3.09167200	-3.02901200	-1.05601900
H	2.02356100	-3.09959000	-0.82505400
H	3.65619200	-3.47996300	-0.23481100
H	3.28208400	-3.62852500	-1.95369300
C	2.59774700	-2.18712700	2.31371100
H	1.54394300	-1.89660700	2.35836800
H	2.91517300	-2.47251800	3.32357200
H	2.67725300	-3.07189700	1.67809200
C	4.95763800	-1.46496600	1.91892000
H	5.15663400	-2.35791100	1.32026100
H	5.18178700	-1.71671100	2.96233700
H	5.65508500	-0.67879000	1.62318400
C	3.24638800	0.19089300	2.74308900
H	2.21292800	0.54940900	2.66863800
H	3.91598900	1.02207000	2.50805300
H	3.42290600	-0.10707400	3.78306400
C	3.60337100	1.18575800	-0.18099600
C	5.00299600	1.34000900	-0.14718000
C	2.80521500	2.34093300	-0.35262100
C	5.61065500	2.58422700	-0.28152700
H	5.63518000	0.47528300	-0.00697600
C	3.43613800	3.58988400	-0.48024500
C	4.82070900	3.71998700	-0.44921400
H	6.69294900	2.66225800	-0.25087700
H	2.81322800	4.46900200	-0.61290000
H	5.27707500	4.69943400	-0.55434200
C	1.31294200	2.37128500	-0.39181900
C	0.61015100	2.92099400	0.69056000
C	0.59948600	1.98341500	-1.53578900
C	-0.77459300	3.07968800	0.63079700
H	1.15963700	3.23264100	1.57398000
C	-0.78671400	2.14566200	-1.59519600
H	1.13504400	1.57354000	-2.38582500
C	-1.47487400	2.69562900	-0.51378100
H	-1.30489800	3.51361100	1.47389300
H	-1.32166700	1.84190700	-2.49004300
H	-2.54802300	2.83777500	-0.56435500
C	-4.13256700	-0.40897000	-1.02092800
C	-3.60419800	-1.57389000	-1.19670900
C	-4.58252200	0.84858800	-0.69572000
C	-4.83141100	1.81353700	-1.71669900
C	-4.77557200	1.19018300	0.67693200
C	-5.24919500	3.08343200	-1.36544200
H	-4.68299300	1.53334400	-2.75383900
C	-5.19351800	2.46984900	1.00279300
H	-4.60142500	0.44151100	1.43912600
C	-5.42765000	3.40986400	-0.01001600
H	-5.44074500	3.82686400	-2.13174600
H	-5.34345500	2.74178500	2.04216600
H	-5.75834000	4.40942800	0.25596600
Cl	-4.39848800	-3.07220200	-1.58424500

Cartesian coordinates of the optimized geometry for **21c** at B3LYP-D3BJ/6-31G(d),def2-TZVP level of theory (number of imaginary frequencies = 0):

C	-0.55339700	2.50737100	-0.23599600
C	-1.50512300	1.71129000	-0.23870800
Au	0.36896600	0.40035400	-0.10433800
C	-2.81892400	1.17715600	-0.33032600
C	-3.40243200	0.41835900	0.63281400
C	-4.77114000	-0.10734400	0.68176100
C	-5.85681100	0.66679600	0.23382400
C	-5.02068400	-1.38830200	1.20866100
C	-7.15051800	0.15512400	0.28040600
H	-5.69203500	1.67479400	-0.12511700
C	-6.31398900	-1.89885500	1.23914100
H	-4.19793200	-1.97846800	1.59484700
C	-7.38286600	-1.13085100	0.77121000
H	-7.97958900	0.76783900	-0.05919800
H	-6.48948000	-2.89314400	1.63777200
H	-8.39296500	-1.52732400	0.80282200
P	2.41327000	-0.64513700	0.31501700
C	3.73085600	0.34427000	-0.63149400
C	2.63041800	-0.65351300	2.20439100
C	0.43707200	3.53552100	-0.19674800
C	0.88593900	4.03018600	1.04319200
C	0.99499200	4.02055800	-1.39476400
C	1.88437400	4.99777300	1.07655100
H	0.45039700	3.64628100	1.95956700
C	1.99045800	4.99111300	-1.34658100
H	0.64312700	3.63061800	-2.34420300
C	2.43853600	5.47680200	-0.11476000
H	2.22964400	5.38233900	2.03094900
H	2.41841000	5.36941500	-2.26943000
H	3.21754200	6.23211600	-0.08271600
Cl	-2.38407500	0.01161100	2.01093300
C	3.81901300	1.76028500	-0.03626200
H	4.43274500	2.38211600	-0.69768900
H	2.84061100	2.23853400	0.04913900
H	4.29540700	1.76035700	0.94798600
C	3.22475300	0.42977500	-2.08584900
H	2.28475800	0.98593900	-2.15836900
H	3.97405300	0.95141100	-2.69212100
H	3.07736700	-0.56408500	-2.52155100
C	5.13165100	-0.29075500	-0.64787400
H	5.81118400	0.39744500	-1.16378300
H	5.53581900	-0.45693500	0.35323100
H	5.14490800	-1.23458000	-1.19672900
C	2.10273500	0.67761200	2.77842600
H	1.03703100	0.81023300	2.56795800
H	2.23051900	0.66250000	3.86687600
H	2.63661100	1.54794200	2.39149800
C	1.73500700	-1.79703700	2.72212200
H	1.71011300	-1.75730800	3.81701600
H	0.70459800	-1.69850000	2.36075700
H	2.11054200	-2.77932100	2.42539100
C	4.07652900	-0.86330600	2.67959200
H	4.73665800	-0.05013600	2.36612100
H	4.08344100	-0.88467900	3.77553200
H	4.49682100	-1.81160200	2.33898700
C	2.53621600	-2.39435600	-0.22295400
C	3.73716800	-3.08747200	0.02713500
C	1.44425600	-3.10742900	-0.77227300
C	3.87534600	-4.44174200	-0.25793000
H	4.57987900	-2.56848800	0.46013000
C	1.60286300	-4.47705100	-1.04509300
C	2.79849800	-5.14303600	-0.79766300
H	4.81634600	-4.94271300	-0.05392600
H	0.76256800	-5.01552000	-1.47150900
H	2.88752300	-6.20074000	-1.02516700
C	0.10025000	-2.54278900	-1.08446900

C	-0.09817300	-1.65757200	-2.15998000
C	-1.01980000	-3.01848900	-0.38693500
C	-1.38881800	-1.26580200	-2.52697500
H	0.75650200	-1.32189400	-2.73884700
C	-2.30615100	-2.62766100	-0.75781700
H	-0.87471700	-3.70940400	0.43823600
C	-2.49463700	-1.75293700	-1.82761100
H	-1.52849500	-0.59180300	-3.36633300
H	-3.16467900	-3.01512400	-0.22013000
H	-3.49656900	-1.45416400	-2.11640700
Cl	-3.61573800	1.54974300	-1.84888100

Cartesian coordinates of the optimized geometry for **22c** at B3LYP-D3BJ/6-31G(d),def2-TZVP level of theory (number of imaginary frequencies = 0):

C	-1.50473500	-0.68336000	-1.06268700
C	-2.44602800	-1.31079000	-0.27386700
Au	0.48282700	-0.46941600	-0.60747600
P	2.79900500	-0.41728000	-0.18673500
C	3.15509900	-1.41596600	1.38356200
C	3.64857700	-1.10238400	-1.75096800
C	2.30110200	-0.76208600	2.48749000
H	1.23582600	-0.78326100	2.24514600
H	2.44633500	-1.31627000	3.42242100
H	2.60028100	0.27589000	2.66305700
C	4.62391100	-1.40064900	1.83461100
H	4.71003300	-1.99405500	2.75262000
H	5.30025700	-1.83828700	1.09843400
H	4.96579000	-0.38875800	2.06558800
C	2.67991000	-2.86332300	1.16621800
H	1.63920600	-2.90254700	0.82713400
H	3.30259700	-3.39715700	0.44263200
H	2.74350400	-3.40497900	2.11716200
C	2.74412300	-2.20921300	-2.33637400
H	1.76596500	-1.81930200	-2.63187100
H	3.22857100	-2.62017400	-3.22983400
H	2.58216600	-3.03270900	-1.63656000
C	5.05108400	-1.70153100	-1.55326000
H	5.03094200	-2.58234200	-0.90652500
H	5.42719300	-2.02513600	-2.53091900
H	5.77766600	-0.99295200	-1.15205300
C	3.70427800	0.06150700	-2.75934900
H	2.71770600	0.51928900	-2.89709800
H	4.40404800	0.84298200	-2.45418300
H	4.02837600	-0.32707100	-3.73161100
C	3.50617300	1.24973300	0.15009200
C	4.90664200	1.39346800	0.15743700
C	2.71342800	2.38075200	0.45563000
C	5.52208800	2.60588900	0.45067100
H	5.53351600	0.54091800	-0.06308000
C	3.35257500	3.59387900	0.76240600
C	4.73828100	3.71607800	0.75940400
H	6.60517700	2.67770500	0.44201500
H	2.73435100	4.45476300	0.99718700
H	5.20024900	4.66987600	0.99484300
C	1.21944000	2.42196200	0.49219800
C	0.47442000	2.60981900	-0.68251500
C	0.55047500	2.42416200	1.72354000
C	-0.90976700	2.78851200	-0.62445900
H	0.98743900	2.63897200	-1.63903200
C	-0.83283600	2.60038600	1.78044000
H	1.12156700	2.30079500	2.63844200
C	-1.56665100	2.78123100	0.60645900
H	-1.46988500	2.93434100	-1.54248300
H	-1.33469800	2.60880300	2.74415500

H	-2.63997500	2.93121800	0.65329600
C	-4.39126900	-0.32790100	-0.46368400
C	-3.86390800	-1.47465300	-0.70862400
Cl	-2.02648900	-0.05053100	-2.62145400
C	-2.11148600	-1.78859500	1.08896000
C	-1.59586600	-0.89681400	2.04302100
C	-2.31955500	-3.13101200	1.44339000
C	-1.28275900	-1.34384700	3.32441500
H	-1.44629600	0.14186600	1.77219300
C	-1.99460400	-3.57543200	2.72440500
H	-2.71554600	-3.82798700	0.71221400
C	-1.47695300	-2.68450500	3.66695400
H	-0.88502600	-0.64569400	4.05525200
H	-2.14565500	-4.61823700	2.98578600
H	-1.22917900	-3.03297500	4.66504000
C	-4.78202000	0.96575500	-0.18315900
C	-5.20481400	1.31483600	1.13126100
C	-4.73410200	1.95780500	-1.20465800
C	-5.55997300	2.62362100	1.40801200
H	-5.23032800	0.54736600	1.89715000
C	-5.10212800	3.25957500	-0.90797700
H	-4.40917100	1.67138400	-2.19776200
C	-5.51221000	3.59069600	0.39168600
H	-5.88039500	2.90192800	2.40638800
H	-5.07477400	4.02224400	-1.67898100
H	-5.80007700	4.61343800	0.61566700
Cl	-4.57091100	-2.91455100	-1.35820800

Cartesian coordinates of the optimized geometry for **23c** at B3LYP-D3BJ/6-31G(d),def2-TZVP level of theory (number of imaginary frequencies = 0):

C	1.42010500	0.27504600	-0.59275300
C	2.20945900	1.22001800	-0.03018800
Au	-0.58255900	-0.03390800	-0.68943900
P	-2.92616200	-0.05991400	-0.63654600
C	-3.47802300	1.71249400	-0.24372100
C	-3.56728100	-0.73554500	-2.29626200
C	-2.72831700	2.10803400	1.04512800
H	-1.64256700	2.08239600	0.91870500
H	-3.00779700	3.13244500	1.31674200
H	-3.00102800	1.45470200	1.87985300
C	-4.98590500	1.87170300	0.00758100
H	-5.19282900	2.93030600	0.20390800
H	-5.59474900	1.57327100	-0.84809600
H	-5.31167800	1.30701900	0.88412200
C	-3.03798500	2.63502700	-1.39387300
H	-1.97275000	2.52477200	-1.61943600
H	-3.61050400	2.45482900	-2.30806100
H	-3.21061700	3.67618200	-1.09760900
C	-2.62393300	-0.23378600	-3.40999100
H	-1.59761900	-0.58053500	-3.25644900
H	-2.97458100	-0.62800300	-4.37084000
H	-2.60472300	0.85608100	-3.48449700
C	-5.00504300	-0.33884200	-2.66782900
H	-5.11324900	0.74140600	-2.79420700
H	-5.25526300	-0.80528000	-3.62790700
H	-5.74630400	-0.68276200	-1.94414800
C	-3.44763900	-2.26952500	-2.20808600
H	-2.43784100	-2.57755500	-1.91210200
H	-4.15759800	-2.69872900	-1.49709700
H	-3.65077500	-2.69841400	-3.19600600
C	-3.63447300	-1.10954000	0.69695000
C	-5.03062600	-1.29067100	0.73381300
C	-2.84390800	-1.75753100	1.67460400
C	-5.64306500	-2.08597100	1.69654200

H	-5.65497600	-0.80276500	-0.00163000
C	-3.48056000	-2.55595100	2.63917000
C	-4.86143300	-2.72469300	2.65778300
H	-6.72209300	-2.20394600	1.69283500
H	-2.86528700	-3.04844800	3.38563800
H	-5.32190400	-3.34943200	3.41684900
C	-1.35441900	-1.68191000	1.78860500
C	-0.55867700	-2.71319100	1.26594100
C	-0.74163500	-0.67032800	2.54195100
C	0.82021400	-2.72478400	1.48336200
H	-1.03042100	-3.51268700	0.70227800
C	0.63723800	-0.68291300	2.75682800
H	-1.34979800	0.12021400	2.96879000
C	1.42186200	-1.70909400	2.22921700
H	1.41930600	-3.53776900	1.08268100
H	1.09324000	0.10418100	3.34879900
H	2.49336900	-1.72269500	2.40838300
C	3.62827500	0.92489500	0.10468600
C	4.07489100	-0.26384200	-0.37225500
C	5.35497400	-0.94501200	-0.47746200
C	5.45809800	-2.31931000	-0.19537000
C	6.50175900	-0.22651500	-0.86010700
C	6.68892300	-2.96027400	-0.28712500
H	4.57768900	-2.87737200	0.11068700
C	7.73080600	-0.87503600	-0.93956800
H	6.42324600	0.82732400	-1.10027100
C	7.82760200	-2.23898500	-0.65559300
H	6.76209000	-4.01998900	-0.06474200
H	8.61254700	-0.31570400	-1.23539900
H	8.78782600	-2.74042700	-0.72423200
Cl	2.64222200	-1.11049600	-1.06401700
C	1.54116100	2.45589200	0.45043400
C	1.55258800	2.79501300	1.81076100
C	0.83105700	3.25648400	-0.45580400
C	0.85872900	3.91829400	2.25596000
H	2.09868400	2.17710700	2.51554500
C	0.13934900	4.38061800	-0.00603300
H	0.83369700	2.99617800	-1.50929300
C	0.14985400	4.71131100	1.34973200
H	0.87106100	4.17497500	3.31074900
H	-0.40283600	4.99837400	-0.71525900
H	-0.38694800	5.58751100	1.70037800
Cl	4.69980300	2.03703900	0.88119500

Cartesian coordinates of the optimized geometry for **24c** at B3LYP-D3BJ/6-31G(d),def2-TZVP level of theory (number of imaginary frequencies = 1):

C	-1.01872100	0.99127100	-0.03270500
C	-2.26201200	0.79449300	-0.16134100
Au	0.96289500	0.61316600	0.10358600
C	-3.56405900	0.23013500	-0.28687800
C	-4.14595800	-0.54476100	0.66320100
C	-5.46342800	-1.19024700	0.60388300
C	-6.59650800	-0.46159900	0.20455100
C	-5.60438600	-2.53959500	0.97102300
C	-7.84325300	-1.07910100	0.16120000
H	-6.50252500	0.58792500	-0.04857000
C	-6.85035600	-3.15484500	0.90789400
H	-4.73593800	-3.10273000	1.29615500
C	-7.97207500	-2.42649500	0.50408700
H	-8.71560900	-0.50553300	-0.13566000
H	-6.94780300	-4.20105900	1.17991700
H	-8.94520800	-2.90611600	0.46447500
P	3.25703500	0.17082100	0.31214300
C	4.19416400	1.33014200	-0.85812000

C	3.71147100	0.37144900	2.15017500
C	-1.86980100	2.42219100	-0.21320700
C	-2.18247300	3.17811900	0.93867600
C	-1.85707600	3.03433800	-1.48509400
C	-2.47257000	4.52897100	0.81365400
H	-2.19161100	2.68542000	1.90530100
C	-2.14264800	4.38847100	-1.59822100
H	-1.62171400	2.43227000	-2.35613100
C	-2.44986600	5.13112200	-0.45179300
H	-2.71513300	5.11836000	1.69186300
H	-2.13273600	4.86857400	-2.57122500
H	-2.67639900	6.18903000	-0.54445500
Cl	-3.21687600	-0.85055800	2.12110000
C	3.96008200	2.78244600	-0.40666800
H	4.34963300	3.46041600	-1.17481000
H	2.89529700	3.00224100	-0.27294800
H	4.48166900	3.01143100	0.52689000
C	3.55725500	1.11599300	-2.24640400
H	2.49301200	1.37281500	-2.25195100
H	4.06704000	1.75723100	-2.97471400
H	3.66394600	0.07913000	-2.58084800
C	5.70244500	1.05580900	-0.97346900
H	6.13765600	1.80193800	-1.64863100
H	6.22470000	1.13631200	-0.01797700
H	5.90286500	0.07115200	-1.40162500
C	2.91145100	1.56138900	2.72064500
H	1.83264100	1.39674000	2.64169200
H	3.16003500	1.67344200	3.78238900
H	3.14802500	2.50426200	2.22221500
C	3.23482500	-0.91740200	2.84830700
H	3.33396000	-0.79036200	3.93241800
H	2.18036700	-1.12533100	2.63196000
H	3.82375700	-1.78854000	2.55151200
C	5.20193000	0.60731500	2.44164600
H	5.56277100	1.54314900	2.00711500
H	5.33479400	0.68048500	3.52742800
H	5.83955400	-0.20881700	2.09719500
C	3.72914400	-1.53752500	-0.17492400
C	5.06009600	-1.94895500	0.03402700
C	2.81290000	-2.46631500	-0.72095500
C	5.48905900	-3.23414000	-0.27957000
H	5.77839500	-1.25609800	0.44915100
C	3.26552200	-3.75952000	-1.03146200
C	4.58395800	-4.14802200	-0.81688700
H	6.52233500	-3.51640100	-0.10368700
H	2.55487800	-4.46469600	-1.45090300
H	4.90081600	-5.15567600	-1.06747800
C	1.36794500	-2.20909600	-1.00833200
C	0.96508900	-1.70297100	-2.25336700
C	0.38580000	-2.61821600	-0.09321900
C	-0.39001100	-1.60741800	-2.57458300
H	1.71852600	-1.40331200	-2.97508000
C	-0.96907800	-2.52206900	-0.41644500
H	0.69133000	-3.02599800	0.86584900
C	-1.35956200	-2.02316100	-1.66014000
H	-0.68705800	-1.22389000	-3.54624800
H	-1.71582700	-2.84506300	0.30191200
H	-2.41201000	-1.96316700	-1.91875400
Cl	-4.32656100	0.52790400	-1.83958600

Cartesian coordinates of the optimized geometry for **32c** at B3LYP-D3BJ/6-31G(d),def2-TZVP level of theory (number of imaginary frequencies = 1):

C	-1.55009100	-0.92596300	0.04769500
C	-2.04379800	-0.97582200	-1.13200300

C	-2.07455200	-1.03216500	1.40977900
Cl	-1.78818700	-0.88989500	-2.77980600
C	-2.96465400	-2.05993900	1.76168600
C	-1.69534400	-0.08927300	2.38030800
C	-2.23986500	-0.14286700	3.66101500
C	-3.14567800	-1.15158300	3.99850300
C	-3.49606000	-2.11439900	3.04884600
H	-4.18374700	-2.91284000	3.31025900
H	-3.22905000	-2.81615900	1.03250800
H	-0.99861200	0.69499100	2.10993600
H	-1.95164400	0.60003300	4.39875600
Au	0.56090700	-0.63635000	-0.11554800
H	-3.56494900	-1.19636600	4.99886800
P	2.88697500	-0.52241200	0.00970800
C	3.59456000	-1.41370300	-1.50606500
C	3.37006000	-1.29017600	1.68389900
C	2.93370300	-0.74348600	-2.72758500
H	1.84463900	-0.85393700	-2.71443800
H	3.31340200	-1.21611800	-3.64068800
H	3.17462000	0.32325400	-2.77881100
C	5.12039400	-1.31042000	-1.66820300
H	5.41248300	-1.89893700	-2.54577500
H	5.66847300	-1.70847600	-0.81174600
H	5.44054000	-0.28118000	-1.84458500
C	3.17628900	-2.89330500	-1.44584600
H	2.09866700	-3.00775600	-1.28680500
H	3.70381000	-3.43681800	-0.65709400
H	3.42817900	-3.37166500	-2.39920100
C	2.44670000	-2.49500200	1.96115000
H	1.39524400	-2.19636600	2.01063100
H	2.72026000	-2.92743700	2.93050600
H	2.54130300	-3.28066900	1.20839200
C	4.82950100	-1.76022300	1.78957100
H	5.05361900	-2.56713900	1.08703200
H	4.99402900	-2.15373700	2.79942600
H	5.54875500	-0.95274000	1.64104600
C	3.08786900	-0.20605700	2.74279700
H	2.06110600	0.17058200	2.66808600
H	3.77241300	0.64132300	2.65797100
H	3.20946200	-0.64620800	3.73906000
C	3.57649100	1.18188500	-0.00164400
C	4.96490500	1.33761800	0.17985900
C	2.77473800	2.34309000	-0.10416100
C	5.55794600	2.59291600	0.26482300
H	5.59849000	0.46600400	0.26202500
C	3.39157600	3.60147000	-0.00801000
C	4.76423500	3.73511500	0.17427700
H	6.63127900	2.67419700	0.40469500
H	2.76761800	4.48601900	-0.08870400
H	5.20952300	4.72287800	0.24190400
C	1.29623300	2.36443000	-0.31319900
C	0.44547500	2.71427700	0.74596300
C	0.74551900	2.17301500	-1.58946100
C	-0.92854800	2.84803800	0.53721500
H	0.87022800	2.88592500	1.73101200
C	-0.62718700	2.31639300	-1.79697700
H	1.39975300	1.93202800	-2.42104800
C	-1.46871100	2.64733600	-0.73364900
H	-1.57976800	3.11664500	1.36351400
H	-1.03672400	2.17757700	-2.79260400
H	-2.53359400	2.76438800	-0.89530700
C	-4.52933900	-0.44188500	-0.82595200
C	-4.19718500	-1.57671700	-1.16322200
C	-4.74393800	0.89339500	-0.42075100
C	-5.05220000	1.87998900	-1.38185500
C	-4.63853500	1.24342900	0.94243800

C	-5.24276100	3.19752000	-0.98127900
H	-5.13750500	1.59750300	-2.42583400
C	-4.82069200	2.56675700	1.32603300
H	-4.41134500	0.47819300	1.67450600
C	-5.12051400	3.54260200	0.36929200
H	-5.48365400	3.95731100	-1.71775100
H	-4.73672900	2.83882400	2.37344400
H	-5.26589200	4.57363400	0.67684400
Cl	-4.34947300	-3.16344100	-1.61249200

Cartesian coordinates of the optimized geometry for **33c** at B3LYP-D3BJ/6-31G(d),def2-TZVP level of theory (number of imaginary frequencies = 1):

C	-1.49876000	-0.66483400	-1.27080500
C	-2.08547100	-1.06724200	-0.19205700
Au	0.54161500	-0.48934300	-0.78587800
P	2.76398800	-0.45428000	-0.05456900
C	2.79082200	-1.50475900	1.53088400
C	3.89110100	-1.09145700	-1.44452500
C	1.71162300	-0.89423400	2.44800800
H	0.71406900	-0.96563900	2.00853300
H	1.70013700	-1.44211700	3.39742500
H	1.91716100	0.15830500	2.66897400
C	4.12452300	-1.51078300	2.29598800
H	4.02516700	-2.18987300	3.15102800
H	4.96297400	-1.86579800	1.69375700
H	4.36911400	-0.52277700	2.69143200
C	2.39407500	-2.94722700	1.17024800
H	1.46527300	-2.98214300	0.59100100
H	3.17709700	-3.46006500	0.60453200
H	2.23364500	-3.51193200	2.09602900
C	3.14162500	-2.20861800	-2.19985800
H	2.20283900	-1.84589400	-2.62966000
H	3.77493400	-2.56357600	-3.02114200
H	2.91299100	-3.06550800	-1.56184000
C	5.25291800	-1.63863300	-0.98791100
H	5.14883700	-2.51841300	-0.34762100
H	5.81718200	-1.94748800	-1.87561800
H	5.85920300	-0.89433800	-0.46749600
C	4.09778200	0.09583200	-2.40454100
H	3.14058800	0.50238200	-2.74923000
H	4.67067600	0.90449800	-1.94347700
H	4.64690000	-0.25261800	-3.28663800
C	3.37736100	1.20236200	0.45927900
C	4.70344100	1.31057900	0.92034700
C	2.55621200	2.35524400	0.48947500
C	5.20886200	2.50273000	1.42933800
H	5.35459800	0.44800700	0.89298000
C	3.07620400	3.54214200	1.03137200
C	4.38314300	3.62334900	1.50174200
H	6.23765100	2.55021500	1.77225500
H	2.43765300	4.41976900	1.05223300
H	4.75748500	4.55861100	1.90638600
C	1.17861700	2.44804400	-0.07700600
C	1.00087600	2.51432400	-1.46882700
C	0.06205900	2.59976300	0.75654300
C	-0.26709000	2.71506000	-2.01404100
H	1.86703100	2.43065500	-2.11767800
C	-1.20845900	2.79700600	0.20905300
H	0.19593600	2.56308800	1.83391300
C	-1.37379800	2.85477500	-1.17491800
H	-0.38862100	2.76580300	-3.09146400
H	-2.06990100	2.92336400	0.85938100
H	-2.35978600	3.01382400	-1.59566200
C	-4.53152500	-0.55772200	-0.35840200

C	-4.17529200	-1.69031900	-0.67193100
Cl	-2.13541900	-0.32845300	-2.82743700
C	-2.00059200	-1.35758700	1.20702600
C	-2.01720300	-0.30233500	2.14215400
C	-1.87462000	-2.68820700	1.65408200
C	-1.90461000	-0.57941700	3.50045300
H	-2.09006900	0.71538000	1.78087300
C	-1.75867100	-2.95311000	3.01384000
H	-1.85840800	-3.49312600	0.92730200
C	-1.77695000	-1.90168300	3.93685500
H	-1.90752200	0.23336000	4.22009700
H	-1.65342200	-3.97700800	3.35800100
H	-1.68628300	-2.11393000	4.99779100
C	-4.77384600	0.77070200	0.05901500
C	-4.96645400	1.05315900	1.42813600
C	-4.80181200	1.81720700	-0.88610000
C	-5.16403300	2.36688000	1.84030700
H	-4.95545100	0.23987300	2.14513100
C	-5.00995500	3.12409500	-0.46127600
H	-4.65575300	1.58637700	-1.93557900
C	-5.18408000	3.40123500	0.89940000
H	-5.31333500	2.58576100	2.89287700
H	-5.03796800	3.93038100	-1.18742700
H	-5.34598900	4.42426300	1.22477200
Cl	-4.21647100	-3.26492000	-1.16793600

Cartesian coordinates of the optimized geometry for **44c** at B3LYP-D3BJ/6-31G(d),def2-TZVP level of theory (number of imaginary frequencies = 1):

C	1.17331200	0.70158500	0.71273200
C	2.42404400	1.04229700	0.60069300
Au	-0.67476800	0.11201400	0.59143500
P	-2.73041700	-1.06027900	0.41385500
C	-2.36485100	-2.65350600	-0.54631400
C	-3.42830700	-1.31976100	2.16146900
C	-1.66457000	-2.20130500	-1.84458200
H	-0.71333900	-1.69874900	-1.64398100
H	-1.45656900	-3.08441800	-2.45944700
H	-2.29689500	-1.52679700	-2.43061400
C	-3.61250700	-3.46716400	-0.92599900
H	-3.28541400	-4.37185600	-1.45150300
H	-4.19446900	-3.78588200	-0.05898000
H	-4.26600800	-2.91366700	-1.60388400
C	-1.40123300	-3.52283300	0.28018200
H	-0.51419400	-2.96393800	0.59690300
H	-1.88351900	-3.94247000	1.16738200
H	-1.06438700	-4.36248700	-0.33840200
C	-2.24772100	-1.59273400	3.11708800
H	-1.54755400	-0.75233800	3.14721800
H	-2.64251100	-1.73698800	4.12925000
H	-1.68779100	-2.49132600	2.84770000
C	-4.44025600	-2.46891900	2.29705300
H	-3.99056000	-3.44057900	2.07746800
H	-4.79076200	-2.49974400	3.33518300
H	-5.32189900	-2.34002700	1.66620100
C	-4.08722500	0.01452000	2.56195100
H	-3.39073400	0.85496000	2.45877600
H	-4.97683800	0.23162900	1.96577100
H	-4.38549600	-0.04008900	3.61496700
C	-3.98566500	-0.16255500	-0.58019700
C	-5.28063800	-0.70510900	-0.68237700
C	-3.68733300	1.02229700	-1.29275500
C	-6.25974600	-0.11303700	-1.47358900
H	-5.53033600	-1.60955100	-0.14463100
C	-4.68317600	1.59462400	-2.10050900

C	-5.95597500	1.04017400	-2.19577500
H	-7.24949000	-0.55512800	-1.52857600
H	-4.44481700	2.50165700	-2.64700400
H	-6.70642700	1.50996200	-2.82385800
C	-2.38727300	1.75600000	-1.24105600
C	-2.10802200	2.62836000	-0.17234400
C	-1.48524700	1.69061200	-2.31275900
C	-0.95526000	3.42198500	-0.18701300
H	-2.81925500	2.71236600	0.64426600
C	-0.33049600	2.47692200	-2.31791700
H	-1.69677000	1.02639700	-3.14518300
C	-0.06698300	3.35024500	-1.26142500
H	-0.75820700	4.10084800	0.63645700
H	0.35839800	2.41237400	-3.15466200
H	0.82522400	3.96704400	-1.26971500
C	3.45672200	-0.01919700	0.51393100
C	3.53810300	-0.98157900	-0.43280500
C	4.62152000	-1.96709200	-0.59611900
C	4.31569500	-3.32694600	-0.76574600
C	5.96279100	-1.55375000	-0.61371800
C	5.33732800	-4.25964300	-0.91750800
H	3.27925100	-3.64837000	-0.76856300
C	6.97978800	-2.48978200	-0.78170500
H	6.20456800	-0.50182500	-0.51140800
C	6.67065600	-3.84347300	-0.92682700
H	5.09322700	-5.31094400	-1.03380600
H	8.01380400	-2.16037000	-0.80380300
H	7.46589900	-4.57153900	-1.05385700
Cl	2.26822000	-1.09118000	-1.65242500
C	2.74493200	2.49575400	0.53292300
C	2.19936800	3.38974800	1.46290900
C	3.54132200	2.96249400	-0.52178500
C	2.44602700	4.75614500	1.33088900
H	1.60649300	3.00910700	2.28799700
C	3.78982900	4.32707300	-0.64007600
H	3.95437400	2.25876600	-1.23781000
C	3.23582600	5.22516900	0.27973400
H	2.03211800	5.45143800	2.05454900
H	4.41456400	4.69278600	-1.44902300
H	3.43141500	6.28841100	0.18126600
Cl	4.62408200	0.08298700	1.81079300

Cartesian coordinates of the optimized geometry for **45c** at B3LYP-D3BJ/6-31G(d),def2-TZVP level of theory (number of imaginary frequencies = 0):

C	1.56586600	0.74473700	0.57562900
C	2.82799900	0.99458100	0.46137800
Au	-0.36634200	0.25023100	0.56574300
P	-2.57900600	-0.53143000	0.48416400
C	-2.55947200	-2.11193200	-0.56540700
C	-3.19096700	-0.77549200	2.26795100
C	-1.91132400	-1.70950000	-1.90621500
H	-0.89543100	-1.32384100	-1.77345600
H	-1.86378200	-2.59117500	-2.55651600
H	-2.49964100	-0.94533200	-2.42349000
C	-3.94643600	-2.70419100	-0.86172800
H	-3.81397800	-3.61449300	-1.45837400
H	-4.49176500	-2.98276200	0.04185000
H	-4.56297300	-2.01727200	-1.44585600
C	-1.67855300	-3.16159800	0.13493600
H	-0.69058100	-2.76498800	0.39164400
H	-2.14429000	-3.54597500	1.04655600
H	-1.53147900	-4.01210100	-0.54112000
C	-2.00400700	-1.28124300	3.11542100
H	-1.17857400	-0.56323000	3.12654900

H	-2.34477900	-1.41912200	4.14801500
H	-1.61411500	-2.23934000	2.76335200
C	-4.36060900	-1.75760300	2.44104400
H	-4.08654100	-2.77654400	2.15532800
H	-4.63725500	-1.78120400	3.50150100
H	-5.25384000	-1.46621900	1.88517500
C	-3.59418800	0.62314300	2.77444200
H	-2.78476700	1.34958400	2.63659800
H	-4.48580000	1.00389500	2.27053200
H	-3.80671800	0.56302600	3.84783400
C	-3.74543600	0.60715200	-0.36401200
C	-5.11700700	0.28849700	-0.35821000
C	-3.32536700	1.77777400	-1.03860500
C	-6.05762400	1.08848400	-0.99862700
H	-5.46060700	-0.60109600	0.15154500
C	-4.28853800	2.56724500	-1.68715500
C	-5.63975100	2.23500700	-1.67254100
H	-7.10755800	0.81446200	-0.97157200
H	-3.95629400	3.46233400	-2.20358900
H	-6.36000000	2.86881500	-2.18047400
C	-1.91729500	2.27414000	-1.11665400
C	-1.41537000	3.13447600	-0.12759400
C	-1.11962500	1.98767100	-2.23364700
C	-0.13590700	3.68407600	-0.24923800
H	-2.03803900	3.38314300	0.72670000
C	0.15926000	2.53470200	-2.35093000
H	-1.50861600	1.33960900	-3.01291800
C	0.65506300	3.38337800	-1.35991400
H	0.23123800	4.35855700	0.51902200
H	0.76433200	2.30669000	-3.22312500
H	1.64907300	3.80922900	-1.45371100
C	2.91276400	-0.50606600	0.41968300
C	2.71738700	-1.27198500	-0.71588600
C	2.79539200	-2.72765200	-0.83055600
C	1.79040100	-3.41430100	-1.54315600
C	3.85430700	-3.45903600	-0.25923600
C	1.82146700	-4.79977000	-1.63747400
H	0.97932500	-2.85822200	-1.99844000
C	3.89567800	-4.84332700	-0.39125700
H	4.65630300	-2.94433300	0.25343900
C	2.87587700	-5.51773500	-1.06582000
H	1.03073000	-5.32057500	-2.16812400
H	4.72750000	-5.39582300	0.03334200
H	2.90718900	-6.59920700	-1.15461700
Cl	2.26308700	-0.44617300	-2.17776700
C	3.88590500	1.98623800	0.46222900
C	3.58866300	3.31954300	0.79029800
C	5.19461000	1.61970600	0.12231900
C	4.59769000	4.27608400	0.76406900
H	2.57517200	3.58802300	1.06941700
C	6.19951500	2.58365000	0.10045500
H	5.41684000	0.58628900	-0.12558500
C	5.90284600	3.91060600	0.41864200
H	4.37054400	5.30647200	1.01926300
H	7.21319900	2.29995500	-0.16392200
H	6.68805600	4.65995900	0.40244700
Cl	3.39190000	-1.27210700	1.93193400

Cartesian coordinates of the optimized geometry for **46c** at B3LYP-D3BJ/6-31G(d),def2-TZVP level of theory (number of imaginary frequencies = 1):

C	1.30835600	0.35603600	0.47107400
C	2.25352800	1.21261600	0.47401900
Au	-0.64164900	-0.21552700	0.36005200
P	-2.88014200	-0.91125600	0.28364400

C	-3.00521400	-2.40373300	-0.87683400
C	-3.42703300	-1.26133800	2.07408200
C	-2.37036300	-1.94894000	-2.20663200
H	-1.32302500	-1.65577700	-2.08165500
H	-2.41037800	-2.77879100	-2.92174800
H	-2.91604300	-1.10600700	-2.64229400
C	-4.43750400	-2.88109400	-1.16731500
H	-4.38248700	-3.75665300	-1.82493300
H	-4.97757000	-3.18260600	-0.26760000
H	-5.02040800	-2.11780000	-1.68775400
C	-2.17788600	-3.55860100	-0.28574100
H	-1.16054400	-3.24293000	-0.03030100
H	-2.64392500	-3.98258000	0.60801000
H	-2.10514800	-4.36054600	-1.02948100
C	-2.24489200	-1.91221500	2.82309200
H	-1.36882800	-1.25724400	2.84481100
H	-2.54995900	-2.10320000	3.85854200
H	-1.94310000	-2.86606400	2.38446300
C	-4.65530500	-2.17268600	2.22678000
H	-4.47168700	-3.17851700	1.84012900
H	-4.88132100	-2.27179300	3.29496700
H	-5.54991000	-1.77027500	1.74808300
C	-3.70905500	0.11443300	2.70915800
H	-2.85832200	0.79502000	2.58705800
H	-4.59427800	0.59221000	2.28260700
H	-3.87667800	-0.01975100	3.78392800
C	-4.02633100	0.35917400	-0.38957500
C	-5.41082900	0.10432000	-0.34332000
C	-3.58514300	1.59794000	-0.91097800
C	-6.34418900	1.03493400	-0.78733100
H	-5.77125200	-0.83669500	0.04794000
C	-4.54318600	2.52618600	-1.35136400
C	-5.90704800	2.25804500	-1.29323500
H	-7.40360800	0.80429600	-0.73506800
H	-4.19439600	3.47375100	-1.74982200
H	-6.62121400	2.99748400	-1.64220200
C	-2.16107700	2.03142200	-1.05292200
C	-1.59429600	2.90906300	-0.11576700
C	-1.42179700	1.69184700	-2.19613500
C	-0.31375800	3.42857400	-0.31491800
H	-2.16827700	3.18942700	0.76251200
C	-0.14348000	2.21469400	-2.39506000
H	-1.86083600	1.03125500	-2.93668600
C	0.41144300	3.08717500	-1.45773200
H	0.11384800	4.10664200	0.41775300
H	0.41233400	1.94963000	-3.28956400
H	1.40215400	3.50106100	-1.61641100
C	2.64042500	-0.43860400	0.55112200
C	3.25996300	-0.98779600	-0.56354100
C	4.42250000	-1.86156800	-0.59940600
C	4.50471200	-2.91225900	-1.53672300
C	5.50695800	-1.63139900	0.27357700
C	5.62373800	-3.73418500	-1.56916700
H	3.68046600	-3.09079400	-2.21779800
C	6.63500700	-2.44000100	0.21169300
H	5.47480400	-0.80260800	0.96962000
C	6.69205200	-3.49902100	-0.69852800
H	5.66798300	-4.55464900	-2.27795400
H	7.47237000	-2.24315500	0.87322700
H	7.57059200	-4.13571900	-0.73458600
Cl	2.55664700	-0.59480200	-2.10843000
C	3.12502800	2.33847800	0.59319300
C	2.99922200	3.17834900	1.71585400
C	4.08066500	2.62188300	-0.40087200
C	3.81144300	4.30336400	1.82675400
H	2.26609400	2.94015300	2.47905700

C	4.89212300	3.74271700	-0.27356900
H	4.17117800	1.96603200	-1.26057600
C	4.75634000	4.58416900	0.83666500
H	3.71327600	4.95676100	2.68769300
H	5.62909800	3.96606300	-1.03827200
H	5.39151600	5.45961800	0.92978900
Cl	3.04977700	-1.05411800	2.16039200

Cartesian coordinates of the optimized geometry for **18d** at B3LYP-D3BJ/6-31G(d),def2-TZVP level of theory (number of imaginary frequencies = 0):

C	-1.04077600	-1.49969600	-0.05760200
C	-1.55774300	-1.97679600	-1.21486700
C	-1.84918500	-1.50806200	1.18165400
Cl	-0.63137400	-1.97512900	-2.72689600
C	-2.57347700	-2.63695900	1.60704800
C	-1.88220300	-0.35605900	1.98981800
C	-2.64984100	-0.31862600	3.15296900
C	-3.37702500	-1.44166500	3.55476900
C	-3.32409700	-2.60569700	2.78165200
H	-3.86135700	-3.49465000	3.09939700
H	-2.52307900	-3.55295500	1.02741500
H	-1.30728700	0.51075500	1.68428700
H	-2.66469700	0.58463800	3.75668200
Au	0.89385400	-0.77160300	0.06452100
H	-3.95842300	-1.42123600	4.47166500
P	3.15326300	-0.12929100	0.23517700
C	4.10808300	-1.16304500	-1.03978400
C	3.69803600	-0.39974100	2.03935800
C	3.40516900	-0.89180200	-2.38640600
H	2.35625800	-1.20202400	-2.36855500
H	3.91544700	-1.45764200	-3.17483000
H	3.45081600	0.16918700	-2.65502900
C	5.60063700	-0.83391800	-1.20405200
H	6.03304200	-1.54709900	-1.91601500
H	6.16356800	-0.92234400	-0.27226600
H	5.75302100	0.16661500	-1.61452500
C	3.94896100	-2.64975000	-0.67310100
H	2.90122000	-2.91891300	-0.50334700
H	4.52574100	-2.91420700	0.21800300
H	4.32464500	-3.26197800	-1.50127300
C	3.02248000	-1.68241000	2.56709600
H	1.93206200	-1.61839900	2.50689900
H	3.29926800	-1.81650200	3.61961400
H	3.33811000	-2.57619800	2.02416300
C	5.21215600	-0.51517800	2.27022200
H	5.63870800	-1.38583500	1.76488100
H	5.39311600	-0.64196900	3.34438100
H	5.75817400	0.37704400	1.95749400
C	3.13801600	0.80296300	2.82495800
H	2.06380700	0.93170700	2.64772700
H	3.64384000	1.73581400	2.56299000
H	3.28178600	0.62527100	3.89711000
C	3.54414400	1.63178500	-0.14315900
C	4.88050600	2.06309900	-0.03370800
C	2.55557900	2.59083700	-0.46576700
C	5.24735300	3.38947700	-0.23734500
H	5.65344800	1.35307900	0.22152400
C	2.94365500	3.92710400	-0.66229700
C	4.27025600	4.33103000	-0.55458200
H	6.28870300	3.68182900	-0.14427100
H	2.17579300	4.65312800	-0.91091600
H	4.53689700	5.37103500	-0.71610300
C	1.09223600	2.32571100	-0.60095200
C	0.21719000	2.80011400	0.38701100

C	0.55536200	1.73161700	-1.75224400
C	-1.16358800	2.68060500	0.22856200
H	0.62804400	3.27047200	1.27553700
C	-0.82697300	1.61570300	-1.91094600
H	1.22250700	1.37392800	-2.52965800
C	-1.68835900	2.08986200	-0.92187300
H	-1.82900700	3.05703600	1.00075400
H	-1.22315600	1.14820100	-2.80694700
H	-2.76244800	2.00835800	-1.04848000
C	-3.75090500	-1.46966700	-1.27628200
C	-2.95315700	-2.47189600	-1.45590000
C	-4.48685000	-0.36898800	-0.95726300
C	-4.88477000	0.57320700	-1.96421300
C	-4.84312900	-0.12099100	0.40703400
C	-5.58425300	1.69705000	-1.61849000
H	-4.61400800	0.38172000	-2.99685600
C	-5.54519500	1.01142100	0.75080500
H	-4.55279700	-0.83838100	1.16431200
C	-5.92091800	1.93179600	-0.25659700
H	-5.89667900	2.42941400	-2.35427600
H	-5.80126100	1.18833200	1.78751500
C	-3.26884800	-3.87466600	-1.89301800
H	-2.75953700	-4.08271900	-2.83924700
H	-2.88898300	-4.58136800	-1.14764400
H	-4.34294400	-4.02408600	-2.02127800
O	-6.59615000	3.05106500	-0.03139300
C	-6.99905000	3.39471700	1.30461700
H	-7.52789000	4.34154100	1.20945500
H	-7.66872500	2.63108400	1.71139500
H	-6.12341100	3.51806500	1.94919500

Cartesian coordinates of the optimized geometry for **21d** at B3LYP-D3BJ/6-31G(d),def2-TZVP level of theory (number of imaginary frequencies = 0):

C	-0.54342200	2.61060800	-0.66778000
C	0.63009900	2.20807700	-0.58446900
Au	-0.76134700	0.36203900	-0.50795400
C	2.01629600	1.91954300	-0.74507400
C	2.73189600	1.33413800	0.25703900
C	4.10618200	0.84318500	0.18724600
C	4.57242200	0.17387200	-0.96562600
C	4.99195400	0.99060100	1.26787300
C	5.86758200	-0.30537600	-1.03756100
H	3.90042300	0.00329700	-1.79840900
C	6.30000800	0.52644700	1.20152400
H	4.65479900	1.48831800	2.17060900
C	6.75004700	-0.12664600	0.04277800
H	6.22615200	-0.83083500	-1.91602700
H	6.95792800	0.67297600	2.04901500
P	-1.16596300	-1.92876200	-0.67600100
C	-2.24953300	-2.19151600	-2.20939900
C	0.55158500	-2.75213700	-0.75335600
C	-1.71182400	3.44687700	-0.65264800
C	-1.73379800	4.58367400	0.17420800
C	-2.83323900	3.13256300	-1.43767100
C	-2.87222600	5.38294500	0.22110000
H	-0.86452400	4.81879300	0.77891100
C	-3.96615600	3.93934400	-1.38704900
H	-2.80649000	2.25712700	-2.07863200
C	-3.99041100	5.06075900	-0.55357100
H	-2.88829000	6.25863700	0.86245000
H	-4.83071900	3.69392100	-1.99576700
H	-4.87685100	5.68598300	-0.51147800
Cl	1.92977100	1.10002000	1.81114800
C	2.60038500	2.31747600	-2.08667100

H	2.32149700	1.60308700	-2.87107300
H	2.20707300	3.29489900	-2.38205800
H	3.68893500	2.37707600	-2.04332200
C	-1.47464400	-1.71856500	-3.45184700
H	-2.15804800	-1.69533000	-4.30826700
H	-1.06506700	-0.71101000	-3.32197300
H	-0.65534800	-2.39720300	-3.70527600
C	-3.48426800	-1.29174000	-1.99857400
H	-3.21050500	-0.23447100	-1.92136200
H	-4.15954200	-1.40685400	-2.85410500
H	-4.03655400	-1.57168000	-1.09578800
C	-2.73858000	-3.63358100	-2.42531500
H	-3.30807600	-3.66568500	-3.36142200
H	-1.92101500	-4.35191600	-2.51653800
H	-3.40707800	-3.95916500	-1.62563500
C	1.49312000	-1.88335800	-1.61106100
H	1.60056700	-0.87784400	-1.19698500
H	2.48543400	-2.34885600	-1.61621600
H	1.15910700	-1.79543900	-2.64737500
C	1.07725300	-2.75419000	0.69626600
H	2.11827400	-3.09699500	0.69172100
H	1.06151900	-1.75025900	1.13452100
H	0.50090200	-3.42255700	1.34056200
C	0.55710600	-4.18050900	-1.31966600
H	0.22933300	-4.21203500	-2.36222600
H	1.58525700	-4.55978100	-1.29022200
H	-0.05367700	-4.87052800	-0.73511400
C	-2.04612400	-2.69532700	0.73987500
C	-2.24481700	-4.09033800	0.72556300
C	-2.46353400	-1.96344800	1.87543500
C	-2.83406700	-4.75678300	1.79456900
H	-1.93281200	-4.67172300	-0.13000900
C	-3.05003600	-2.65643300	2.94735900
C	-3.23742100	-4.03445600	2.91660400
H	-2.97221700	-5.83245200	1.74951600
H	-3.36696800	-2.08547200	3.81430600
H	-3.69622400	-4.53905900	3.76123400
C	-2.33259100	-0.48768600	2.05332600
C	-3.25205100	0.39623600	1.46569400
C	-1.36291500	0.02136000	2.92745200
C	-3.20111900	1.76136000	1.75076300
H	-4.02286900	0.00521400	0.80884600
C	-1.30766500	1.38839700	3.20379700
H	-0.65575600	-0.66013500	3.39075800
C	-2.22856600	2.25844600	2.62033300
H	-3.91728700	2.43602800	1.29352000
H	-0.55088500	1.77081200	3.88198500
H	-2.19379400	3.31998000	2.84158300
O	7.99225300	-0.62310800	-0.12662100
C	8.93982100	-0.47238100	0.92800300
H	9.85813300	-0.93568800	0.56754400
H	9.12335900	0.58631700	1.14546700
H	8.60312700	-0.98327500	1.83777500

Cartesian coordinates of the optimized geometry for **22d** at B3LYP-D3BJ/6-31G(d),def2-TZVP level of theory (number of imaginary frequencies = 0):

C	-0.96888300	-1.22312800	-1.16490500
C	-1.75830400	-2.03838100	-0.42058400
Au	0.90661700	-0.53927200	-0.66840800
P	3.08999700	0.18001900	-0.17988900
C	3.84622100	-0.95803300	1.13537900
C	4.04866000	0.19564700	-1.82891900
C	2.86594200	-0.92716400	2.32478400
H	1.86264900	-1.25707000	2.04259600

H	3.23638100	-1.59985300	3.10770700
H	2.79794700	0.07700500	2.75495100
C	5.23261100	-0.53514800	1.64632800
H	5.56845500	-1.27117500	2.38671800
H	5.98591100	-0.49897600	0.85705800
H	5.19974500	0.43677500	2.14424600
C	3.91131400	-2.38664700	0.56749400
H	2.94731500	-2.70391500	0.15729300
H	4.66931300	-2.48355300	-0.21485200
H	4.18155300	-3.07904100	1.37369800
C	3.58108900	-1.01481500	-2.66540300
H	2.50836300	-0.97196600	-2.87435400
H	4.11510800	-1.00538600	-3.62297900
H	3.79233300	-1.96891800	-2.17654000
C	5.58075300	0.13815300	-1.72303400
H	5.92546400	-0.79496100	-1.26970700
H	5.99948900	0.18097300	-2.73558100
H	6.00640900	0.97558800	-1.16725000
C	3.61817000	1.48338900	-2.55845400
H	2.52660900	1.56291200	-2.61970300
H	4.00100600	2.38248200	-2.06950400
H	4.00867200	1.45808000	-3.58241800
C	3.20506100	1.88379000	0.51133600
C	4.47548900	2.47412100	0.65397700
C	2.06599700	2.64964300	0.85353900
C	4.63412700	3.78033700	1.10525000
H	5.36126900	1.90719200	0.40443400
C	2.24495600	3.96914600	1.30100400
C	3.50861100	4.53716700	1.42651200
H	5.63008800	4.20170400	1.20033000
H	1.36326100	4.54737900	1.55951000
H	3.61280500	5.55981300	1.77604400
C	0.64996100	2.17435200	0.80015500
C	-0.19358600	2.58442400	-0.24283900
C	0.10869200	1.42584700	1.85503700
C	-1.54713600	2.24501600	-0.23383600
H	0.21808200	3.17015100	-1.05922200
C	-1.24433800	1.08916800	1.86473200
H	0.74955400	1.11748100	2.67358300
C	-2.07473400	1.50169500	0.82225500
H	-2.18734400	2.56206100	-1.05154300
H	-1.64344200	0.50294000	2.68616300
H	-3.13035400	1.25460000	0.83847700
C	-3.96775700	-1.57310800	-0.69521400
C	-3.11880000	-2.52563100	-0.87493000
Cl	-1.59213900	-0.67112900	-2.74238800
C	-1.31659100	-2.55021500	0.90493100
C	-2.09616200	-2.34926600	2.05228300
C	-0.10452200	-3.24754000	1.02548800
C	-1.65834500	-2.79713300	3.29753900
H	-3.04090600	-1.82041000	1.96848400
C	0.33319000	-3.69599200	2.27135900
H	0.48508000	-3.43946200	0.13560200
C	-0.43830300	-3.46720600	3.41176900
H	-2.26842600	-2.62362700	4.17925900
H	1.27505300	-4.23120700	2.34772000
H	-0.09834600	-3.81887000	4.38105700
C	-4.73526800	-0.45359800	-0.53632900
C	-5.46128400	-0.23006900	0.67357300
C	-4.78453400	0.54216600	-1.56670200
C	-6.18572800	0.92402500	0.85967100
H	-5.42918400	-0.98753400	1.44958200
C	-5.50116900	1.69494900	-1.37882600
H	-4.22356500	0.37170600	-2.47766100
C	-6.20815300	1.90286500	-0.16662000
H	-6.72933000	1.07986800	1.78269200

H	-5.54893600	2.46948000	-2.13605800
C	-3.30823800	-3.92248200	-1.39112200
H	-4.35608200	-4.14449500	-1.60360200
H	-2.71703700	-4.04127400	-2.30552100
H	-2.92620100	-4.63146200	-0.64917900
O	-6.86614200	3.05165900	-0.08465700
C	-7.61893400	3.37296600	1.09674400
H	-8.05043100	4.35348000	0.90285200
H	-8.41516400	2.63951800	1.25581800
H	-6.96082900	3.42022900	1.96970900

Cartesian coordinates of the optimized geometry for **23d** at B3LYP-D3BJ/6-31G(d),def2-TZVP level of theory (number of imaginary frequencies = 0):

C	1.11572700	0.54293700	-0.43676900
C	1.74402700	1.59021200	0.14825400
Au	-0.83623000	0.01260200	-0.62798000
P	-3.16285800	-0.25910400	-0.71466700
C	-3.91343100	1.44315200	-0.33997500
C	-3.63593700	-0.97052000	-2.41450600
C	-3.27770900	1.89287600	0.99203600
H	-2.19054400	1.98568500	0.92229700
H	-3.67948000	2.87622300	1.26214100
H	-3.51918600	1.19833400	1.80300300
C	-5.44103300	1.45096700	-0.17319300
H	-5.76250200	2.48241300	0.01376600
H	-5.96907900	1.10055200	-1.06244400
H	-5.75746200	0.84986400	0.68228700
C	-3.50358400	2.42082500	-1.45523600
H	-2.42225900	2.41520400	-1.62358400
H	-4.00750900	2.20068600	-2.40062800
H	-3.78990100	3.43640300	-1.15806600
C	-2.68853300	-0.35763800	-3.46753800
H	-1.64194500	-0.59689300	-3.25677400
H	-2.94049400	-0.77271300	-4.45040800
H	-2.77991700	0.72920800	-3.53172500
C	-5.08403700	-0.72126400	-2.86588600
H	-5.29890800	0.34340600	-2.98926800
H	-5.22896200	-1.19735000	-3.84283300
H	-5.82455600	-1.15174100	-2.18919800
C	-3.36204300	-2.48504700	-2.33355000
H	-2.34263600	-2.69062700	-1.98623400
H	-4.06021600	-2.99512500	-1.66546500
H	-3.46702400	-2.91889700	-3.33460400
C	-3.84848000	-1.39241200	0.56221900
C	-5.22200300	-1.70050900	0.51992100
C	-3.05809000	-1.97635500	1.57929000
C	-5.81189200	-2.55668500	1.44384700
H	-5.84693600	-1.26414700	-0.24667300
C	-3.67181300	-2.83556800	2.50555700
C	-5.03057200	-3.12866800	2.44648400
H	-6.87370300	-2.77265800	1.37864700
H	-3.05595400	-3.27757100	3.28256700
H	-5.47402200	-3.79847500	3.17676600
C	-1.58874900	-1.77223800	1.76832300
C	-0.67752000	-2.69483800	1.23182800
C	-1.10760300	-0.74818200	2.59591300
C	0.68646900	-2.58674000	1.50758700
H	-1.04731100	-3.50370500	0.60859900
C	0.25657100	-0.64171600	2.87010700
H	-1.80628300	-0.04049800	3.03022300
C	1.15678400	-1.55900700	2.32735600
H	1.37748600	-3.31409100	1.09104600
H	0.61146100	0.15152600	3.52064300
H	2.21719000	-1.47785700	2.54911700

C	3.19114100	1.53812600	0.33164000
C	3.80325200	0.42907600	-0.14074400
C	5.16623100	-0.04750000	-0.26940900
C	5.53278200	-1.33989300	0.13882200
C	6.15956900	0.79834000	-0.80776100
C	6.84541000	-1.78401000	0.02970600
H	4.78224000	-2.00422400	0.55826900
C	7.47137200	0.37120900	-0.90471700
H	5.88730300	1.78859500	-1.15775000
C	7.82841600	-0.92460400	-0.48904500
H	7.09725000	-2.78552100	0.35522700
H	8.24364400	1.01258100	-1.31498000
Cl	2.50154300	-0.64226500	-0.86866000
C	0.88613400	2.71698000	0.60650500
C	0.70604100	2.97048600	1.97362400
C	0.20230600	3.50208300	-0.33221200
C	-0.14170200	3.99436600	2.39359900
H	1.21523200	2.35086600	2.70515000
C	-0.64522600	4.52641500	0.09012100
H	0.34151700	3.30355600	-1.39020300
C	-0.81848100	4.77427200	1.45262200
H	-0.27754300	4.18071300	3.45463000
H	-1.16795500	5.13031500	-0.64537500
H	-1.47714100	5.57224200	1.78171500
C	3.94795600	2.64624700	1.01088400
H	3.91902400	3.55518800	0.39895000
H	3.48828500	2.89417300	1.97186800
H	4.99050200	2.37191100	1.17969500
O	9.12874500	-1.24669100	-0.63350600
C	9.56286500	-2.54691800	-0.24102200
H	10.63214000	-2.57552100	-0.44952900
H	9.39289700	-2.71356200	0.82919700
H	9.05438100	-3.32502800	-0.82242300

Cartesian coordinates of the optimized geometry for **24d** at B3LYP-D3BJ/6-31G(d),def2-TZVP level of theory (number of imaginary frequencies = 1):

C	-0.60365200	1.32795200	0.17698900
C	-1.84429000	1.24472900	-0.06515400
Au	1.27910600	0.59917500	0.34954700
C	-3.21833800	0.91565400	-0.24655900
C	-3.73914500	-0.13627300	0.44832200
C	-5.13148100	-0.58376800	0.43716100
C	-6.18459300	0.35323300	0.50916600
C	-5.46176400	-1.94748100	0.38133400
C	-7.50531800	-0.05735000	0.50087600
H	-5.95792800	1.40923000	0.60836900
C	-6.78530000	-2.37157100	0.36125900
H	-4.67013300	-2.68831100	0.34428700
C	-7.82075800	-1.42514700	0.41881600
H	-8.31809100	0.65710200	0.57277100
H	-7.00200200	-3.43108600	0.30550800
P	3.42312100	-0.30347600	0.61327100
C	4.70250500	1.02249200	0.17165800
C	3.54763000	-0.95289100	2.39927900
C	-1.15653600	2.81897700	-0.22580900
C	-1.55436500	3.72607400	0.77804700
C	-0.89254400	3.28154400	-1.53290600
C	-1.68300400	5.07630800	0.47526300
H	-1.75947400	3.35091600	1.77529800
C	-1.00734400	4.63461200	-1.82186100
H	-0.58885900	2.56368000	-2.28748100
C	-1.40569200	5.52888500	-0.82002300
H	-1.99325100	5.77866200	1.24219800
H	-0.79624700	4.99850900	-2.82233900

H	-1.50280300	6.58529900	-1.05138500
Cl	-2.65032800	-1.09513000	1.45230000
C	-3.98608100	1.72098000	-1.27102800
H	-4.23990500	2.71743500	-0.89006800
H	-3.37310100	1.86338700	-2.16651200
H	-4.90819000	1.21496400	-1.55718000
C	4.56995700	2.18672600	1.16894300
H	5.18161200	3.02626700	0.81883500
H	3.53531100	2.53631500	1.25305600
H	4.92683300	1.91537200	2.16642900
C	4.31874300	1.51182800	-1.23964300
H	3.31204900	1.94138300	-1.26326200
H	5.02843000	2.28620000	-1.55317100
H	4.36539500	0.69912600	-1.97153800
C	6.15868800	0.53155600	0.13096400
H	6.80410500	1.38529800	-0.10700600
H	6.49701600	0.12275500	1.08513600
H	6.31076700	-0.22021200	-0.64683100
C	2.80793500	0.03588200	3.32523700
H	1.75206700	0.13043400	3.05495100
H	2.86197800	-0.34085000	4.35328600
H	3.25141300	1.03418200	3.31319500
C	2.79129800	-2.29587300	2.41611800
H	2.69785400	-2.63642100	3.45369100
H	1.77949800	-2.19192900	2.00712700
H	3.31291100	-3.07016700	1.84838300
C	4.97329600	-1.14480100	2.94069300
H	5.52321100	-0.20157500	2.99476200
H	4.90510700	-1.53979100	3.96113500
H	5.55954200	-1.86032100	2.36127500
C	3.76269800	-1.72709300	-0.50046000
C	4.96265200	-2.44325700	-0.32320200
C	2.86367700	-2.15579000	-1.50455800
C	5.27892300	-3.55243400	-1.10018400
H	5.66643300	-2.13196900	0.43578300
C	3.20065500	-3.27892000	-2.27884000
C	4.38928400	-3.97543300	-2.08628300
H	6.21267700	-4.08022800	-0.93331700
H	2.50428000	-3.59983800	-3.04719300
H	4.61817600	-4.83975100	-2.70204500
C	1.55172800	-1.52258300	-1.84647400
C	1.48282900	-0.49703800	-2.80096800
C	0.35646900	-2.06536600	-1.34862500
C	0.24639300	-0.02643000	-3.24732700
H	2.40108300	-0.08818900	-3.21035700
C	-0.87811500	-1.59107200	-1.79220800
H	0.40210700	-2.86956800	-0.62039100
C	-0.93584000	-0.57455700	-2.74662600
H	0.21108700	0.75058100	-4.00628500
H	-1.79250600	-2.01781400	-1.39332900
H	-1.89791900	-0.21995200	-3.10489200
O	-9.13646500	-1.72407300	0.41279100
C	-9.52640000	-3.09331400	0.35231100
H	-9.17008200	-3.56581400	-0.57082900
H	-9.15182400	-3.64966400	1.21969900
H	-10.61632100	-3.09050100	0.36336500

Cartesian coordinates of the optimized geometry for **32d** at B3LYP-D3BJ/6-31G(d),def2-TZVP level of theory (number of imaginary frequencies = 1):

C	-1.17239900	-1.46718200	0.01220500
C	-1.53618500	-1.67182800	-1.18854000
C	-1.71955100	-1.58614500	1.36109700
Cl	-1.25968600	-1.65666200	-2.82678800
C	-2.46128200	-2.71743100	1.73753500

C	-1.52214000	-0.55322200	2.29307100
C	-2.09699900	-0.63102900	3.55908900
C	-2.85209800	-1.74910400	3.92087000
C	-3.02302000	-2.79448400	3.00997800
H	-3.59631000	-3.67290600	3.29003600
H	-2.59511300	-3.52823900	1.03338700
H	-0.94394000	0.31578800	2.00319000
H	-1.95013800	0.17973100	4.26641500
Au	0.85336000	-0.76061500	-0.12181700
H	-3.29517900	-1.81171700	4.90997400
P	3.09931500	-0.17774700	0.09008300
C	4.05060000	-0.98372300	-1.33709500
C	3.63526100	-0.74628100	1.82679400
C	3.33543100	-0.52443600	-2.62375000
H	2.29091600	-0.85111100	-2.64894800
H	3.84950200	-0.95716000	-3.48965800
H	3.36132200	0.56486000	-2.72983800
C	5.53098800	-0.57998200	-1.43961500
H	5.98175300	-1.13704200	-2.26924100
H	6.10140900	-0.81769200	-0.53934300
H	5.64640200	0.48356400	-1.65976100
C	3.93498200	-2.51232800	-1.20504000
H	2.89498100	-2.83509100	-1.08706700
H	4.51603000	-2.89646800	-0.36203500
H	4.33028100	-2.97741000	-2.11533600
C	2.95432400	-2.09525500	2.13944200
H	1.86355000	-2.01011500	2.13033800
H	3.25896700	-2.41459200	3.14279600
H	3.23930400	-2.88353200	1.43921400
C	5.15119200	-0.90992000	2.02040500
H	5.56606300	-1.69060200	1.37739100
H	5.33686800	-1.21093600	3.05802100
H	5.70470100	0.01589800	1.85336900
C	3.08974600	0.31301900	2.80476800
H	2.01438300	0.47262200	2.66397100
H	3.59730100	1.27451500	2.69660500
H	3.24250700	-0.04183000	3.83034900
C	3.43454400	1.62770300	0.00303700
C	4.75064700	2.07175300	0.23827400
C	2.42430400	2.59384900	-0.21535300
C	5.07463200	3.42414400	0.26543600
H	5.53941700	1.35277400	0.40806300
C	2.77046300	3.95453400	-0.17699300
C	4.07554500	4.37421000	0.05986400
H	6.09979600	3.72974500	0.44948700
H	1.98810700	4.68758600	-0.34673100
H	4.30998900	5.43400400	0.08053900
C	0.98713700	2.29855800	-0.49304200
C	0.02472800	2.49434500	0.50882600
C	0.56107200	1.94956500	-1.78340800
C	-1.33243100	2.32026000	0.23096500
H	0.34906100	2.78654900	1.50380400
C	-0.79712200	1.78580500	-2.05982700
H	1.29686100	1.82513500	-2.57138100
C	-1.74710200	1.96335600	-1.05259800
H	-2.07127200	2.46453400	1.01331700
H	-1.11327600	1.52676300	-3.06551600
H	-2.80118800	1.83825300	-1.26750600
C	-4.13085200	-1.68808300	-1.05083900
C	-3.68586900	-2.79443300	-1.32408500
C	-4.59559400	-0.38468300	-0.74751100
C	-5.04031900	0.47018600	-1.78192600
C	-4.61178700	0.08833000	0.57715100
C	-5.47359300	1.75203100	-1.49769000
H	-5.03912500	0.11015700	-2.80551400
C	-5.03691200	1.37999700	0.86732300

H	-4.28586000	-0.56194300	1.37980300
C	-5.47062100	2.22215100	-0.17018300
H	-5.82171300	2.41818400	-2.27955400
H	-5.03898300	1.71752500	1.89642000
C	-3.40951000	-4.18398400	-1.68301300
H	-2.83913100	-4.24252100	-2.61681300
H	-2.82993700	-4.69310800	-0.90493800
H	-4.34632000	-4.73498600	-1.82461700
O	-5.90250700	3.48898100	0.00040700
C	-5.93941700	4.02768400	1.31837300
H	-6.32048500	5.04326100	1.21203200
H	-6.61040900	3.44919800	1.96411500
H	-4.93629900	4.05686100	1.76161900

Cartesian coordinates of the optimized geometry for **33d** at B3LYP-D3BJ/6-31G(d),def2-TZVP level of theory (number of imaginary frequencies = 1):

C	-0.97527600	-1.43786600	-1.33927100
C	-1.47186800	-1.75417600	-0.20478300
Au	0.92158500	-0.69152300	-0.75512200
P	2.99099400	-0.01994700	0.10083500
C	3.09900200	-0.80008200	1.83286800
C	4.36429400	-0.55364800	-1.09808900
C	1.83184100	-0.33179100	2.57720000
H	0.91811900	-0.68541300	2.09410100
H	1.84943300	-0.73493300	3.59641500
H	1.78548500	0.75978400	2.64734100
C	4.32434600	-0.38554200	2.66396200
H	4.29583000	-0.93429300	3.61275300
H	5.27233300	-0.62372000	2.17813100
H	4.30953000	0.67972500	2.90337800
C	3.06634000	-2.33123900	1.68503700
H	2.22332700	-2.66305900	1.06977400
H	3.99052100	-2.72247000	1.25024000
H	2.95389200	-2.78055900	2.67859000
C	3.96369700	-1.90868700	-1.71735600
H	3.01725800	-1.83915300	-2.26246700
H	4.74114200	-2.21476900	-2.42689000
H	3.86630300	-2.69877300	-0.96893000
C	5.76240500	-0.69585000	-0.47559100
H	5.79699600	-1.48433100	0.28063500
H	6.46739500	-0.97388700	-1.26772000
H	6.12958100	0.23230500	-0.03282300
C	4.39188800	0.50750800	-2.21502900
H	3.40515800	0.63040000	-2.67521400
H	4.72588600	1.48210500	-1.85004200
H	5.08465300	0.17977300	-2.99842800
C	3.15301900	1.78985900	0.38347100
C	4.36947700	2.28159000	0.89448600
C	2.08541600	2.69861800	0.18791700
C	4.53308000	3.61992600	1.23772500
H	5.20444100	1.60968900	1.03751400
C	2.26158100	4.03996500	0.56491900
C	3.46494200	4.50299800	1.08798500
H	5.48647500	3.96527300	1.62506300
H	1.43602100	4.72830000	0.41313200
H	3.57185400	5.54826000	1.36111300
C	0.78282700	2.36808300	-0.46087100
C	0.72278400	2.20785400	-1.85511200
C	-0.40786600	2.33357600	0.27801400
C	-0.50087600	2.00651800	-2.49401300
H	1.63895900	2.26561400	-2.43461700
C	-1.63218900	2.12679100	-0.36288400
H	-0.36757100	2.46879700	1.35512200
C	-1.67963900	1.96459900	-1.74763900

H	-0.53278300	1.88737500	-3.57249600
H	-2.55453000	2.10680800	0.21097600
H	-2.63248800	1.81130800	-2.23966500
C	-4.08269000	-1.89500600	-0.51975500
C	-3.57181700	-2.99138900	-0.68825800
Cl	-1.54018100	-1.51147900	-2.94745100
C	-1.48344700	-1.87276700	1.21234600
C	-1.91211100	-0.78725000	2.00497700
C	-1.02456900	-3.05477100	1.83139200
C	-1.88523400	-0.89164900	3.39118200
H	-2.23831800	0.12115900	1.51596900
C	-0.99857100	-3.14518500	3.21771500
H	-0.67731700	-3.87582700	1.21322600
C	-1.43190000	-2.06718100	3.99786700
H	-2.20950700	-0.05482700	4.00192800
H	-0.63919700	-4.05167700	3.69422000
H	-1.40930800	-2.14214400	5.08076700
C	-4.62050200	-0.60145600	-0.30768400
C	-4.98759900	-0.17770300	0.98268000
C	-4.78475800	0.29515300	-1.38747400
C	-5.48291900	1.10424500	1.20341400
H	-4.88136300	-0.86487000	1.81516200
C	-5.28451400	1.56640200	-1.17668900
H	-4.51312300	-0.02769300	-2.38685000
C	-5.63448100	1.98636600	0.12148100
H	-5.76296600	1.40114600	2.20661700
H	-5.42357200	2.26305500	-1.99649400
C	-3.13792000	-4.36470100	-0.92054000
H	-3.99819600	-5.00821400	-1.13877200
H	-2.44734100	-4.41965300	-1.76891600
H	-2.63207300	-4.77026600	-0.03676800
O	-6.10874400	3.24628000	0.21816900
C	-6.51754300	3.72758300	1.49516900
H	-6.87054500	4.74515500	1.32827000
H	-7.33194900	3.11879200	1.90503700
H	-5.67745700	3.74192000	2.20040400

Cartesian coordinates of the optimized geometry for **44d** at B3LYP-D3BJ/6-31G(d),def2-TZVP level of theory (number of imaginary frequencies = 1):

C	0.71202500	1.06410700	0.78435100
C	1.86880400	1.65496800	0.74579400
Au	-0.99049800	0.12437100	0.63159600
P	-2.85807800	-1.32717900	0.46005200
C	-2.18863100	-2.98468300	-0.17498900
C	-3.71765600	-1.40912300	2.15261800
C	-1.39836200	-2.64591800	-1.45620600
H	-0.54137600	-1.99745100	-1.25012900
H	-1.01960300	-3.57662800	-1.89401600
H	-2.03132300	-2.15763100	-2.20409700
C	-3.26809800	-4.01944300	-0.53103600
H	-2.76755100	-4.94189900	-0.84768100
H	-3.91327300	-4.27148400	0.31303100
H	-3.89171100	-3.68405800	-1.36252200
C	-1.23111400	-3.56903900	0.87838400
H	-0.46763000	-2.84602200	1.18480100
H	-1.76188900	-3.91462500	1.77001800
H	-0.71681100	-4.43503100	0.44621800
C	-2.63330000	-1.37066200	3.24962400
H	-2.04211700	-0.45104800	3.20268000
H	-3.12423400	-1.40529000	4.22897100
H	-1.94674400	-2.21846000	3.19200000
C	-4.59826900	-2.64802600	2.38078900
H	-4.01731700	-3.57368100	2.36452400
H	-5.05666700	-2.56878300	3.37340000

H	-5.41305100	-2.73202800	1.65894700
C	-4.57004000	-0.12970200	2.25952700
H	-3.96838300	0.77007200	2.08559200
H	-5.40219600	-0.12798300	1.55125000
H	-4.98232400	-0.06199100	3.27264800
C	-4.10055500	-0.78656600	-0.77951200
C	-5.28183000	-1.53977800	-0.92088800
C	-3.89989800	0.32598500	-1.62943700
C	-6.23822500	-1.22597800	-1.88098900
H	-5.45809500	-2.39163100	-0.27875300
C	-4.86826600	0.61583700	-2.60419700
C	-6.02517500	-0.14589500	-2.73637800
H	-7.13908500	-1.82605900	-1.96163700
H	-4.70365100	1.46907500	-3.25464000
H	-6.75783900	0.10759200	-3.49625200
C	-2.74100500	1.26579200	-1.56170900
C	-2.74095100	2.31826800	-0.62940200
C	-1.70234000	1.19276600	-2.50048500
C	-1.72406000	3.27765900	-0.64383500
H	-3.55832700	2.39816600	0.08135100
C	-0.68325100	2.14816900	-2.50613600
H	-1.69922900	0.38713400	-3.22847700
C	-0.69586000	3.19642100	-1.58498800
H	-1.73934600	4.09154500	0.07383100
H	0.11682300	2.07453900	-3.23634400
H	0.09085300	3.94307600	-1.59354800
C	3.13396700	0.86340600	0.79652900
C	3.41911000	-0.14257600	-0.06408000
C	4.71978200	-0.81339000	-0.19971600
C	4.81355400	-2.20762100	-0.32123000
C	5.91081200	-0.06029900	-0.23835500
C	6.04547800	-2.84257100	-0.44027200
H	3.90923600	-2.80710100	-0.31205500
C	7.14186000	-0.67905100	-0.36680900
H	5.86209300	1.02261400	-0.19301400
C	7.22273100	-2.07896800	-0.46234100
H	6.08066100	-3.92213100	-0.51823300
H	8.06024100	-0.10372700	-0.40967500
Cl	2.19715200	-0.72716800	-1.20848600
C	1.88932400	3.14462700	0.65299700
C	1.05802000	3.93218900	1.45926000
C	2.71253300	3.74264100	-0.31107200
C	1.04223900	5.31676100	1.29063400
H	0.44385400	3.45972600	2.21896600
C	2.69762400	5.12650700	-0.46691900
H	3.34750100	3.12039400	-0.93455200
C	1.85687200	5.91407400	0.32716600
H	0.40245200	5.92892300	1.91886100
H	3.33925300	5.59213900	-1.20865600
H	1.84687800	6.99219000	0.20073300
C	4.06442300	1.30107800	1.90492800
H	4.50430700	2.28296500	1.69612400
H	3.50067600	1.39516300	2.84015400
H	4.86966900	0.58153000	2.05573300
O	8.46807200	-2.58814100	-0.58113300
C	8.61961100	-3.99926200	-0.69839500
H	8.23268300	-4.51459400	0.18901500
H	8.11360900	-4.37892000	-1.59420600
H	9.69200400	-4.17543100	-0.78323100

Cartesian coordinates of the optimized geometry for **45d** at B3LYP-D3BJ/6-31G(d),def2-TZVP level of theory (number of imaginary frequencies = 0):

C	1.04832700	1.51704800	0.70311400
C	2.18840200	2.10272000	0.57614000

Au	-0.62543700	0.43525800	0.65590000
P	-2.44760300	-1.03632000	0.51056600
C	-1.82218000	-2.57939700	-0.40149000
C	-3.09727300	-1.37122800	2.26713700
C	-1.23354800	-2.06402700	-1.73083900
H	-0.41311400	-1.35758100	-1.57190800
H	-0.84865700	-2.91733100	-2.30238200
H	-1.99484500	-1.57019600	-2.34248000
C	-2.90664900	-3.61857900	-0.72663200
H	-2.43367800	-4.46495600	-1.23884500
H	-3.40110100	-4.01064500	0.16399900
H	-3.66595700	-3.21270100	-1.39891800
C	-0.70344300	-3.22922900	0.43198800
H	0.07532600	-2.50879400	0.70432400
H	-1.08663200	-3.68859300	1.34756300
H	-0.23203000	-4.02221900	-0.16070300
C	-1.88766300	-1.39723500	3.22570700
H	-1.35210700	-0.44327700	3.22601900
H	-2.25065100	-1.58189500	4.24342800
H	-1.17344800	-2.18673800	2.97883800
C	-3.88504200	-2.67814100	2.45159800
H	-3.26530000	-3.55983700	2.26830600
H	-4.22677900	-2.73184300	3.49187800
H	-4.77391400	-2.73807300	1.82082300
C	-3.98298200	-0.16338400	2.63054100
H	-3.45266600	0.78408000	2.47912400
H	-4.90501300	-0.13659000	2.04498200
H	-4.25238900	-0.22770000	3.69093100
C	-3.85114900	-0.40856700	-0.49663700
C	-5.03788700	-1.16516900	-0.54077600
C	-3.78636400	0.79297400	-1.24093200
C	-6.13563400	-0.76333100	-1.29459000
H	-5.10908100	-2.08695100	0.01998900
C	-4.90170100	1.17729600	-2.00267400
C	-6.06595800	0.41593500	-2.03476700
H	-7.03598200	-1.36960800	-1.30322600
H	-4.84160500	2.09969400	-2.57174700
H	-6.91227500	0.74313300	-2.63078700
C	-2.61521500	1.72111300	-1.28721400
C	-2.49955400	2.76258500	-0.35345600
C	-1.68238400	1.63939800	-2.33063400
C	-1.46172600	3.69301600	-0.45549200
H	-3.23378100	2.84718800	0.44230900
C	-0.64499600	2.56777800	-2.42842300
H	-1.77654600	0.84791000	-3.06765600
C	-0.53094200	3.59590300	-1.49170700
H	-1.39210000	4.49871400	0.26977000
H	0.07045900	2.49242700	-3.24144900
H	0.27728600	4.31611200	-1.56925700
C	2.75611300	0.69600700	0.71964600
C	2.85469200	-0.14519900	-0.37438500
C	3.46456900	-1.46750500	-0.39175300
C	2.88721900	-2.51298400	-1.14003900
C	4.64999600	-1.74505500	0.32854600
C	3.43023900	-3.78943100	-1.14042300
H	1.98521100	-2.32570000	-1.71094500
C	5.21593300	-3.00459100	0.30941000
H	5.15486400	-0.95245900	0.86635300
C	4.60695800	-4.04640400	-0.41507700
H	2.94600600	-4.57261200	-1.71032800
H	6.13892600	-3.21439900	0.83843500
Cl	2.16846300	0.35732100	-1.90353700
C	2.89012700	3.36813600	0.46860300
C	2.19855500	4.57384700	0.67581400
C	4.25278000	3.39121000	0.14314700
C	2.86812300	5.78592600	0.54652700

H	1.14720600	4.54581600	0.94342300
C	4.91707400	4.60906200	0.01585400
H	4.78012100	2.45601900	-0.02032100
C	4.22711800	5.80632500	0.21593100
H	2.33389400	6.71703200	0.70893700
H	5.97183900	4.62423400	-0.24017500
H	4.74655600	6.75451800	0.11815300
C	3.25777000	0.31909200	2.09687300
H	4.28482000	0.66668700	2.25980400
H	2.63001100	0.80249400	2.84842100
H	3.23119100	-0.76143800	2.25995300
O	5.22705400	-5.23758100	-0.35864000
C	4.68562200	-6.33505700	-1.09310000
H	4.66533900	-6.11729800	-2.16697400
H	5.35382300	-7.17423700	-0.90190700
H	3.67612800	-6.58194400	-0.74430800

Cartesian coordinates of the optimized geometry for **46d** at B3LYP-D3BJ/6-31G(d),def2-TZVP level of theory (number of imaginary frequencies = 1):

C	0.92154100	0.68788000	0.63365400
C	1.69743400	1.69352000	0.59929400
Au	-0.92049100	-0.16300500	0.47272700
P	-3.02558200	-1.18807900	0.32169800
C	-2.84684600	-2.77314800	-0.70242700
C	-3.65545400	-1.47556200	2.09699400
C	-2.18039100	-2.33871700	-2.02356900
H	-1.19976800	-1.88157900	-1.85697300
H	-2.04367400	-3.22090000	-2.65988300
H	-2.80582100	-1.62622400	-2.57063700
C	-4.16884200	-3.48065700	-1.04116700
H	-3.93878500	-4.38897000	-1.61062600
H	-4.72901100	-3.78424000	-0.15454500
H	-4.81211800	-2.85701500	-1.66610200
C	-1.91185100	-3.74011000	0.04481200
H	-0.97271400	-3.25635800	0.33433900
H	-2.38033300	-4.15376200	0.94221100
H	-1.66833800	-4.58071200	-0.61533900
C	-2.45163000	-1.87114200	2.97793200
H	-1.68975800	-1.08621900	2.99407700
H	-2.80398500	-2.02378800	4.00480000
H	-1.97618300	-2.79776300	2.64796400
C	-4.74116300	-2.55208400	2.25514500
H	-4.38066900	-3.54475300	1.97311600
H	-5.02985300	-2.59954400	3.31182300
H	-5.64740500	-2.33367500	1.68738400
C	-4.18939200	-0.11460100	2.58538400
H	-3.44493600	0.67940300	2.45461800
H	-5.10158100	0.18211600	2.06230400
H	-4.41636000	-0.18634700	3.65534300
C	-4.29588500	-0.16962500	-0.53389400
C	-5.62743500	-0.62884300	-0.54784700
C	-4.00571800	1.07617000	-1.13770000
C	-6.65340900	0.10936200	-1.12802500
H	-5.87248200	-1.57980000	-0.09621300
C	-5.05612800	1.80888600	-1.71532700
C	-6.36615900	1.34099400	-1.71431300
H	-7.66817200	-0.27605400	-1.11848700
H	-4.82259500	2.76361500	-2.17604100
H	-7.15453000	1.93233100	-2.16978200
C	-2.65453700	1.70883800	-1.23524800
C	-2.29543700	2.74337300	-0.35781700
C	-1.79046500	1.38694800	-2.29278000
C	-1.09480700	3.43434000	-0.53069000
H	-2.96798000	3.00833900	0.45273800

C	-0.59293700	2.08126700	-2.46596300
H	-2.07022000	0.60262300	-2.98852200
C	-0.24436000	3.10854100	-1.58835800
H	-0.82706900	4.23228300	0.15567700
H	0.06256100	1.82496600	-3.29271300
H	0.68457300	3.65244300	-1.72633800
C	2.36452600	0.08900100	0.83240700
C	3.06487800	-0.38299200	-0.26485700
C	4.37294800	-1.00764000	-0.24569800
C	4.72268400	-2.03318900	-1.14685100
C	5.35787200	-0.56280900	0.67015500
C	5.97606900	-2.62529300	-1.11666400
H	3.99173400	-2.38284600	-1.86702000
C	6.61565600	-1.12962100	0.69241900
H	5.14012800	0.26621300	1.33289300
C	6.93733200	-2.17710200	-0.19278600
H	6.20250100	-3.42519700	-1.81038900
H	7.38088400	-0.77776000	1.37544100
Cl	2.31678700	-0.23898800	-1.84415400
C	2.40064800	2.93451500	0.64524700
C	2.07514800	3.87063700	1.64548400
C	3.39070200	3.23936800	-0.30886300
C	2.71999400	5.10420600	1.67315900
H	1.31553500	3.62146700	2.37902300
C	4.03669500	4.46903300	-0.26303000
H	3.63753900	2.51040100	-1.07367800
C	3.70028900	5.40267000	0.72369300
H	2.46416700	5.82959400	2.43895200
H	4.80037300	4.70541400	-0.99717800
H	4.20571400	6.36303300	0.75250700
C	2.74541700	-0.23262500	2.26210200
H	3.24407400	0.61131800	2.75267100
H	1.83041500	-0.44224800	2.82572100
H	3.40106100	-1.10357500	2.32567000
O	8.18243900	-2.66986900	-0.08500400
C	8.58822200	-3.72465300	-0.95802800
H	8.54608500	-3.40525500	-2.00545000
H	9.61845100	-3.95095300	-0.68480900
H	7.96489400	-4.61452500	-0.81467600

Cartesian coordinates of the optimized geometry for **18e** at B3LYP-D3BJ/6-31G(d),def2-TZVP level of theory (number of imaginary frequencies = 0):

C	-1.02342800	-1.36178100	0.37840400
C	-1.55859700	-2.06977900	-0.64282600
C	-1.81892400	-1.09700700	1.59912400
C	-2.54873800	-2.10195300	2.26022400
C	-1.83342500	0.20065100	2.14360800
C	-2.58971500	0.49287400	3.27771000
C	-3.32262400	-0.51123300	3.91499600
C	-3.28715700	-1.81370000	3.40730900
H	-3.82882900	-2.60898200	3.91126200
H	-2.51265700	-3.12059900	1.88791600
H	-1.25289500	0.97605100	1.65697500
H	-2.59084100	1.50404300	3.67534000
Au	0.91676400	-0.63828200	0.32536400
H	-3.89489300	-0.28992300	4.81092300
P	3.17917200	0.01233000	0.33437400
C	4.11708000	-1.24641900	-0.73354800
C	3.74807200	0.08491600	2.15070600
C	3.40859500	-1.22306400	-2.10373600
H	2.35683800	-1.51184600	-2.02442000
H	3.90765100	-1.93284600	-2.77415600
H	3.46554100	-0.23186700	-2.56647800
C	5.61059300	-0.96458900	-0.96307500

H	6.03331400	-1.79881300	-1.53568100
H	6.17942700	-0.88607400	-0.03417500
H	5.76662300	-0.05680900	-1.54992500
C	3.94944000	-2.63827300	-0.09741300
H	2.90034900	-2.86394200	0.12063600
H	4.52660200	-2.73649800	0.82666300
H	4.31921800	-3.39610600	-0.79797700
C	3.07105000	-1.06833100	2.92008100
H	1.98036200	-1.00340700	2.86716300
H	3.36631500	-1.00709200	3.97433900
H	3.36843700	-2.05145300	2.54811600
C	5.26422100	0.00004200	2.38228800
H	5.67660200	-0.95722100	2.05194500
H	5.45781400	0.08293400	3.45852900
H	5.81433900	0.80796500	1.89591800
C	3.20720000	1.41976500	2.70078700
H	2.13155400	1.52246600	2.51547000
H	3.71562000	2.28135400	2.26056800
H	3.36438800	1.44707900	3.78537400
C	3.56656500	1.66814900	-0.37692800
C	4.90364100	2.11091700	-0.35933200
C	2.57628200	2.54916300	-0.87180900
C	5.26980600	3.37352200	-0.81434300
H	5.67816500	1.46241100	0.02332400
C	2.96402600	3.82307900	-1.32135700
C	4.29114700	4.23859000	-1.29981400
H	6.31175300	3.67682600	-0.78442100
H	2.19514600	4.48909000	-1.70052300
H	4.55703900	5.22832900	-1.65830400
C	1.11216600	2.26610100	-0.94821400
C	0.23987300	2.94453300	-0.08441800
C	0.57111200	1.44162600	-1.94536500
C	-1.14150900	2.79951000	-0.21295900
H	0.65359000	3.59200700	0.68314300
C	-0.81186800	1.30024500	-2.07520000
H	1.23498400	0.92334800	-2.62932900
C	-1.67007200	1.97818200	-1.20995700
H	-1.80433500	3.33445300	0.46177400
H	-1.21002300	0.65041700	-2.84799000
H	-2.74461700	1.87517600	-1.31535800
C	-3.75230400	-1.57312000	-0.79033200
C	-2.95750700	-2.59350700	-0.76465000
C	-4.47824000	-0.42385000	-0.71121400
C	-4.87865800	0.28132900	-1.89561700
C	-4.81987400	0.11726600	0.56956600
C	-5.56796900	1.45894400	-1.79653800
H	-4.61790400	-0.13156600	-2.86404600
C	-5.51088100	1.30323600	0.66495200
H	-4.52606900	-0.42086800	1.46212500
C	-5.89082400	1.98589300	-0.51539600
H	-5.88237800	2.01707000	-2.67117800
H	-5.75550700	1.70339600	1.64049000
C	-3.28443400	-4.05488900	-0.89072200
H	-2.77514100	-4.46137100	-1.77005600
H	-2.90868400	-4.58855900	-0.01132700
H	-4.35951000	-4.22095000	-0.98528600
O	-6.55760800	3.13228300	-0.53310300
C	-6.94764300	3.76001000	0.69951800
H	-7.47306900	4.66687700	0.40505900
H	-7.61719000	3.10685100	1.26707800
H	-6.06605100	4.01485600	1.29554900
Br	-0.56717100	-2.42992800	-2.26533300

Cartesian coordinates of the optimized geometry for **21e** at B3LYP-D3BJ/6-31G(d),def2-TZVP level of theory (number of imaginary frequencies = 0):

C	-0.56226300	2.57037500	-0.82250200
C	0.60002600	2.13744100	-0.74640000
Au	-0.82955600	0.32531200	-0.57627500
C	1.97992400	1.81920600	-0.91178200
C	2.69697200	1.22813100	0.08422500
C	4.05995500	0.71181700	0.00560400
C	4.49492600	0.01275600	-1.14242900
C	4.96817000	0.86534000	1.06665800
C	5.78137600	-0.48736100	-1.22894300
H	3.80483600	-0.16200800	-1.95965200
C	6.26796500	0.38142500	0.98486800
H	4.65171800	1.38047900	1.96710600
C	6.68661700	-0.30067600	-0.16905500
H	6.11553800	-1.03510900	-2.10342500
H	6.94320800	0.53309300	1.81770700
P	-1.29614900	-1.95735600	-0.68868500
C	-2.37953300	-2.21945900	-2.22346300
C	0.39543200	-2.83647400	-0.74698400
C	-1.71820900	3.42255800	-0.81347400
C	-1.72969200	4.55812900	0.01521900
C	-2.83933000	3.12352000	-1.60520000
C	-2.85757100	5.37257500	0.05627300
H	-0.86097800	4.78016500	0.62556400
C	-3.96174200	3.94516100	-1.55965100
H	-2.82062900	2.24831600	-2.24679700
C	-3.97551400	5.06611400	-0.72513300
H	-2.86575900	6.24773800	0.69849300
H	-4.82626800	3.71186600	-2.17316000
H	-4.85376200	5.70305300	-0.68735400
C	2.55953700	2.19597200	-2.26222600
H	2.25324800	1.48381800	-3.03833100
H	2.18784100	3.18141800	-2.55914700
H	3.64962600	2.22710200	-2.23103200
C	-1.58442800	-1.79187900	-3.46968000
H	-2.26041700	-1.77206500	-4.33206500
H	-1.15345700	-0.79109900	-3.35801800
H	-0.77841800	-2.49357700	-3.70165700
C	-3.59067600	-1.28272400	-2.03983100
H	-3.28887200	-0.23213100	-1.97770300
H	-4.26098700	-1.39393700	-2.89976700
H	-4.15884000	-1.53297200	-1.13818600
C	-2.90833700	-3.65101100	-2.41537300
H	-3.47422700	-3.68396300	-3.35364200
H	-2.11176600	-4.39427500	-2.48926900
H	-3.58990900	-3.94229300	-1.61346000
C	1.37147900	-1.99880700	-1.59722100
H	1.51503300	-1.00045300	-1.17755500
H	2.34610600	-2.50029900	-1.60164100
H	1.04450100	-1.89317300	-2.63431300
C	0.90998200	-2.85532100	0.70624200
H	1.93819200	-3.23507700	0.70825500
H	0.92888100	-1.85109800	1.14282700
H	0.30662300	-3.50250800	1.34726600
C	0.36045500	-4.26482800	-1.31258800
H	0.04611100	-4.28735300	-2.35946200
H	1.37510400	-4.67757400	-1.26879700
H	-0.28028900	-4.93399700	-0.73593500
C	-2.20231800	-2.67310900	0.73778200
C	-2.43393100	-4.06314200	0.74511600
C	-2.59505500	-1.91562900	1.86578700
C	-3.02979300	-4.70138700	1.82746100
H	-2.14026400	-4.66360200	-0.10366200
C	-3.18657700	-2.58132600	2.95268600
C	-3.40548300	-3.95479400	2.94316500

H	-3.19347600	-5.77400200	1.79804200
H	-3.48525000	-1.99118500	3.81322900
H	-3.86838300	-4.43680800	3.79868100
C	-2.43719000	-0.44080400	2.02141800
C	-3.29370000	0.45560900	1.36153100
C	-1.51208900	0.06075800	2.94662700
C	-3.22329600	1.82440900	1.62416100
H	-4.03684300	0.07234800	0.66952200
C	-1.43557500	1.43116300	3.19985400
H	-0.85602400	-0.63017100	3.46774300
C	-2.29177900	2.31373700	2.54163700
H	-3.89394800	2.50721400	1.11341200
H	-0.71307600	1.80731400	3.91797100
H	-2.24088000	3.37844000	2.74411300
O	7.91791600	-0.81951500	-0.35115000
C	8.88664900	-0.66305800	0.68308300
H	9.79129000	-1.14708400	0.31531800
H	9.08986900	0.39669700	0.87674600
H	8.55910500	-1.15124200	1.60856500
Br	1.82592600	0.99372400	1.78611100

Cartesian coordinates of the optimized geometry for **22e** at B3LYP-D3BJ/6-31G(d),def2-TZVP level of theory (number of imaginary frequencies = 0):

C	-0.90113800	-1.24955900	-0.93117600
C	-1.71404200	-1.99663900	-0.14643800
Au	0.98794900	-0.56123000	-0.51243700
P	3.18212600	0.17813200	-0.12959500
C	3.98520800	-0.90094300	1.20658300
C	4.08089300	0.12702000	-1.81063600
C	3.04390700	-0.82079000	2.42491800
H	2.03365700	-1.16684300	2.19047300
H	3.44383400	-1.45547700	3.22491300
H	2.98316500	0.20146000	2.81147200
C	5.38647200	-0.45534900	1.65390400
H	5.75019900	-1.16214600	2.40942000
H	6.11244600	-0.44664600	0.83858800
H	5.36645300	0.53439500	2.11607200
C	4.03588500	-2.35228600	0.69791600
H	3.06045200	-2.68801400	0.33118700
H	4.76948400	-2.48127600	-0.10290600
H	4.33272000	-3.00971200	1.52376700
C	3.59545800	-1.12259300	-2.57644500
H	2.51526000	-1.09859000	-2.74628100
H	4.09303800	-1.14914900	-3.55312900
H	3.83529300	-2.05300200	-2.05613400
C	5.61599800	0.08744700	-1.75611000
H	5.98540200	-0.82046300	-1.27207300
H	5.99928000	0.08633400	-2.78348700
H	6.05158300	0.95387300	-1.25511600
C	3.61148400	1.37793200	-2.57935700
H	2.51759900	1.44340300	-2.60683500
H	4.00021400	2.30090400	-2.14226100
H	3.96781600	1.31254700	-3.61394500
C	3.31221800	1.91078200	0.48210100
C	4.58485400	2.50781400	0.56438200
C	2.18091800	2.69061300	0.81859900
C	4.75272400	3.83338600	0.95137500
H	5.46490100	1.93066800	0.31801700
C	2.36905400	4.02923900	1.20061200
C	3.63451600	4.60328000	1.26701400
H	5.75002700	4.25934400	1.00112800
H	1.49331100	4.61805800	1.45541600
H	3.74582900	5.64089600	1.56668400
C	0.76486900	2.21212900	0.82483700

C	-0.10652400	2.56887500	-0.21486300
C	0.25326500	1.51598100	1.92942900
C	-1.45856000	2.22877300	-0.15353100
H	0.28240900	3.11276500	-1.07040300
C	-1.09808200	1.17838500	1.99114100
H	0.91651300	1.24861900	2.74473800
C	-1.95628400	1.53783100	0.95140900
H	-2.11993900	2.50225100	-0.96997400
H	-1.47435200	0.63130600	2.84941900
H	-3.01028900	1.28870800	1.00687400
C	-3.93212200	-1.51822400	-0.42263300
C	-3.08499300	-2.47637700	-0.58021100
C	-1.29010900	-2.44075500	1.20980000
C	-2.09097700	-2.19539400	2.33383200
C	-0.07056000	-3.11359900	1.38450200
C	-1.66754500	-2.57482200	3.60650800
H	-3.04148200	-1.68539700	2.21033400
C	0.35270700	-3.49330000	2.65770700
H	0.53600300	-3.34198300	0.51497500
C	-0.44029500	-3.21993500	3.77338200
H	-2.29495400	-2.36735200	4.46852400
H	1.29992700	-4.01140900	2.77501800
H	-0.11154700	-3.51852000	4.76409100
C	-4.69465800	-0.39157500	-0.30119800
C	-5.42749000	-0.12620300	0.89661500
C	-4.73136000	0.57158200	-1.36321200
C	-6.14764500	1.03618300	1.04028300
H	-5.40480000	-0.85879600	1.69647600
C	-5.44346200	1.73289800	-1.21707000
H	-4.15801200	0.36949900	-2.26011600
C	-6.15872900	1.98177100	-0.01746200
H	-6.69702800	1.22416400	1.95384500
H	-5.48128600	2.48383400	-1.99828400
C	-3.28385000	-3.88018400	-1.07469700
H	-4.33398200	-4.10160000	-1.27611100
H	-2.70063400	-4.00973600	-1.99276000
H	-2.89741900	-4.58038600	-0.32684900
O	-6.81261900	3.13480600	0.02323000
C	-7.57355400	3.49651400	1.18780100
H	-8.00001100	4.47166300	0.95890300
H	-8.37353100	2.77112700	1.36397400
H	-6.92207300	3.56975400	2.06389800
Br	-1.55128800	-0.73605000	-2.69567500

Cartesian coordinates of the optimized geometry for **23e** at B3LYP-D3BJ/6-31G(d),def2-TZVP level of theory (number of imaginary frequencies = 0):

C	0.99322600	0.48451500	-0.36649100
C	1.65779500	1.51755000	0.20360800
Au	-0.97523500	0.02678100	-0.57000300
P	-3.30877400	-0.16408900	-0.69594300
C	-4.00574300	1.55493400	-0.29630000
C	-3.77732800	-0.82245300	-2.41827900
C	-3.37394100	1.95458400	1.05333000
H	-2.28346500	2.01181200	0.99985100
H	-3.74590300	2.94521100	1.33908100
H	-3.64985200	1.25198300	1.84628000
C	-5.53441500	1.61494300	-0.15070800
H	-5.82103300	2.65366200	0.05206600
H	-6.06207300	1.30137600	-1.05394700
H	-5.88485500	1.00963400	0.68837800
C	-3.54513600	2.54010900	-1.38489600
H	-2.46252600	2.49848800	-1.53898300
H	-4.04404800	2.35855800	-2.34112000
H	-3.79792800	3.55908300	-1.06951100

C	-2.79540900	-0.21784900	-3.44400200
H	-1.75993200	-0.49239500	-3.22170400
H	-3.04387200	-0.60577400	-4.43880200
H	-2.85327800	0.87227500	-3.48767400
C	-5.21002900	-0.51785300	-2.88415500
H	-5.39010000	0.55565800	-2.98474400
H	-5.35534500	-0.96605900	-3.87416400
H	-5.97288000	-0.94113700	-2.22805700
C	-3.55251300	-2.34632300	-2.36644300
H	-2.54536300	-2.59146400	-2.00914800
H	-4.27629200	-2.84796300	-1.71951900
H	-3.65632300	-2.75533500	-3.37804000
C	-4.05562300	-1.30176500	0.54203900
C	-5.43952800	-1.55447100	0.47657300
C	-3.30003100	-1.94631400	1.54874300
C	-6.07235900	-2.41398400	1.36849300
H	-6.03846900	-1.07174900	-0.28294200
C	-3.95658700	-2.80851800	2.44224800
C	-5.32500000	-3.04608700	2.36090000
H	-7.14099100	-2.58613200	1.28637100
H	-3.36696700	-3.29722600	3.21160700
H	-5.80229100	-3.71954300	3.06607000
C	-1.82625900	-1.80435700	1.75772500
C	-0.94660700	-2.75360600	1.21503400
C	-1.31403100	-0.81426100	2.60765500
C	0.41730700	-2.70480000	1.50673500
H	-1.34168200	-3.53643000	0.57429500
C	0.05031400	-0.76714800	2.89790900
H	-1.98893500	-0.08644900	3.04628100
C	0.91902400	-1.71040400	2.34877800
H	1.08392200	-3.45181500	1.08540600
H	0.42968200	0.00023700	3.56553500
H	1.97930300	-1.67539800	2.58214200
C	3.10854700	1.48621500	0.37634200
C	3.77629800	0.40300800	-0.08072800
C	5.16605400	0.00575000	-0.19017300
C	5.61096400	-1.24455800	0.26838200
C	6.10756200	0.88745900	-0.76390200
C	6.94890500	-1.61047200	0.17985900
H	4.89965000	-1.93725800	0.70979300
C	7.44357000	0.53842200	-0.84337300
H	5.77587000	1.84464800	-1.15267500
C	7.87877700	-0.71405900	-0.37346400
H	7.26082900	-2.58078000	0.54547700
H	8.17591400	1.20887700	-1.27959200
C	0.81149900	2.65473500	0.66971900
C	0.58849600	2.86552600	2.03772900
C	0.18706600	3.49263200	-0.26425200
C	-0.24064100	3.90214400	2.46378300
H	1.05265300	2.20459400	2.76315800
C	-0.64210700	4.52967100	0.16404900
H	0.35603400	3.32410000	-1.32323000
C	-0.85662300	4.73638600	1.52758000
H	-0.40960000	4.05618700	3.52526200
H	-1.11904800	5.17493200	-0.56744700
H	-1.50126400	5.54390500	1.86116000
C	3.82806900	2.62387500	1.05362800
H	3.75432800	3.53298800	0.44573700
H	3.36605500	2.84811600	2.01957000
H	4.88243900	2.39325400	1.21222300
O	9.19708800	-0.96125000	-0.50392300
C	9.70935800	-2.21389600	-0.05527100
H	10.77920000	-2.18503800	-0.26095400
H	9.54572700	-2.34481000	1.02089800
H	9.25250700	-3.04641400	-0.60326400
Br	2.43488900	-0.83629700	-0.86459400

Cartesian coordinates of the optimized geometry for **24e** at B3LYP-D3BJ/6-31G(d),def2-TZVP level of theory (number of imaginary frequencies = 1):

C	0.46507900	1.45790300	-0.15358300
C	1.70636100	1.37608500	0.08655000
Au	-1.39021200	0.66027200	-0.31614300
C	3.07821500	1.04561700	0.27641900
C	3.58244300	-0.06710400	-0.32878900
C	4.97030300	-0.52376500	-0.29587000
C	6.02555900	0.39590100	-0.48117000
C	5.29855700	-1.87530200	-0.10148500
C	7.34528300	-0.01682700	-0.44736300
H	5.79982500	1.43674400	-0.68664600
C	6.62105800	-2.30002500	-0.05326000
H	4.50453200	-2.60448100	0.01931000
C	7.65829900	-1.36930600	-0.22444000
H	8.15874800	0.68290800	-0.60514200
H	6.83557500	-3.34875600	0.11048300
P	-3.47962900	-0.35723300	-0.59267000
C	-4.83504600	0.93647900	-0.31350300
C	-3.48977600	-1.14827900	-2.32489200
C	1.01499100	2.96256000	0.16364600
C	1.41671900	3.81070800	-0.88944000
C	0.76238600	3.49774700	1.44536300
C	1.55970500	5.17398000	-0.66063100
H	1.61319300	3.38015300	-1.86584600
C	0.89152900	4.86315200	1.66013600
H	0.45625800	2.82452300	2.23893700
C	1.29327200	5.69824100	0.60961700
H	1.87294600	5.83092200	-1.46564300
H	0.68922800	5.28262400	2.64047300
H	1.40175800	6.76455800	0.78333400
C	3.86937200	1.92780700	1.21784200
H	4.12294400	2.88600400	0.74888000
H	3.27216200	2.15056200	2.10758000
H	4.79281500	1.43997700	1.52978600
C	-4.70836600	2.02297900	-1.39556400
H	-5.36988000	2.85867900	-1.13961300
H	-3.68687100	2.41187100	-1.46621400
H	-5.00950400	1.65696000	-2.38111400
C	-4.53633400	1.55248300	1.06839600
H	-3.55193800	2.03060000	1.09783400
H	-5.29322500	2.31370800	1.29032100
H	-4.57811700	0.79793300	1.86045000
C	-6.26884500	0.38238600	-0.29458800
H	-6.96118200	1.22130000	-0.15685300
H	-6.54408200	-0.11847500	-1.22505000
H	-6.42447000	-0.30969000	0.53610800
C	-2.75758400	-0.19815400	-3.29618300
H	-1.72150500	-0.02657000	-2.98966300
H	-2.74489300	-0.65783300	-4.29132600
H	-3.24979000	0.77304900	-3.38496400
C	-2.66815200	-2.44676800	-2.20183400
H	-2.50979900	-2.86162300	-3.20389400
H	-1.68311300	-2.25768200	-1.75929000
H	-3.17686900	-3.20103900	-1.59669300
C	-4.87804700	-1.45711700	-2.90754000
H	-5.47076200	-0.55132200	-3.06001400
H	-4.74447500	-1.92674500	-3.88918800
H	-5.45362200	-2.15496800	-2.29675200
C	-3.80637700	-1.70731900	0.61259200
C	-4.96735700	-2.48735300	0.44515800
C	-2.93020500	-2.02236900	1.67717100
C	-5.26678600	-3.55121100	1.28931900

H	-5.65359800	-2.26307000	-0.35919300
C	-3.24943000	-3.10155300	2.51857200
C	-4.39916200	-3.86268300	2.33477700
H	-6.17043000	-4.13067500	1.12818700
H	-2.57086600	-3.33498400	3.33303300
H	-4.61533000	-4.69024400	3.00337500
C	-1.66166500	-1.30693100	2.02068500
C	-1.67759800	-0.20726100	2.89160200
C	-0.42437700	-1.83319700	1.61757700
C	-0.48272500	0.35089900	3.34986300
H	-2.62922200	0.19195300	3.22784400
C	0.76856400	-1.27229800	2.07330500
H	-0.40347600	-2.69100300	0.95218300
C	0.74214200	-0.18310200	2.94506500
H	-0.51348600	1.18650400	4.04396100
H	1.71598900	-1.68724000	1.74601000
H	1.67203500	0.24006600	3.31415900
O	8.97321500	-1.67133300	-0.20437600
C	9.36032500	-3.02775100	-0.00263800
H	9.01484700	-3.39746200	0.97018900
H	8.97313200	-3.67229700	-0.80067300
H	10.45001900	-3.03047600	-0.02771300
Br	2.37388700	-1.19465100	-1.31759800

Cartesian coordinates of the optimized geometry for **32e** at B3LYP-D3BJ/6-31G(d),def2-TZVP level of theory (number of imaginary frequencies = 1):

C	-1.13407700	-1.32872000	0.42583700
C	-1.50309300	-1.73440200	-0.72298300
C	-1.67570700	-1.20978400	1.77602300
C	-2.44197700	-2.24484900	2.33646700
C	-1.44776500	-0.04255300	2.52451500
C	-2.01729200	0.10462200	3.78672500
C	-2.79671100	-0.91929700	4.33003800
C	-2.99737400	-2.09677000	3.60529600
H	-3.58893900	-2.90285000	4.02843200
H	-2.59945700	-3.15759800	1.77689800
H	-0.84881000	0.75092400	2.09437000
H	-1.84640700	1.01712600	4.35007200
Au	0.89343700	-0.68136300	0.13762200
H	-3.23517800	-0.80660000	5.31677200
P	3.15236800	-0.11405600	0.17932600
C	4.03673100	-1.15866300	-1.13169700
C	3.74490300	-0.40664700	1.96502600
C	3.28219600	-0.89581800	-2.45065100
H	2.23327700	-1.20250400	-2.38903100
H	3.75938400	-1.47039000	-3.25289000
H	3.31902000	0.16206500	-2.73005700
C	5.51891500	-0.80998100	-1.34777400
H	5.92937200	-1.50252000	-2.09191000
H	6.11798500	-0.91358700	-0.44074300
H	5.64418600	0.20183400	-1.73953800
C	3.90042200	-2.64349700	-0.75222600
H	2.85979000	-2.92007500	-0.55158000
H	4.50352100	-2.90014300	0.12308700
H	4.25643900	-3.25698500	-1.58776300
C	3.05499300	-1.67256000	2.51529300
H	1.96594900	-1.56769500	2.52684900
H	3.38954600	-1.83056800	3.54710500
H	3.30347300	-2.57009600	1.94456200
C	5.26421600	-0.56935800	2.13177200
H	5.64314300	-1.45254100	1.61074700
H	5.48278600	-0.70252800	3.19772200
H	5.82585600	0.30521500	1.79834400
C	3.25297000	0.80800400	2.77686800

H	2.17602300	0.96700600	2.64819800
H	3.77211100	1.72808500	2.49800600
H	3.43728000	0.61937300	3.84059700
C	3.51279900	1.64567500	-0.21108700
C	4.84347200	2.09361200	-0.09591200
C	2.51069000	2.58477800	-0.55044800
C	5.18966600	3.42499200	-0.30127100
H	5.62638600	1.39480100	0.16224600
C	2.87954200	3.92573700	-0.74718300
C	4.19887700	4.35014500	-0.62613300
H	6.22557900	3.73433700	-0.20416600
H	2.10316100	4.63803200	-1.00819900
H	4.45064900	5.39382400	-0.78712700
C	1.05971300	2.27961000	-0.72795000
C	0.13841100	2.65391700	0.26162100
C	0.58062500	1.73946900	-1.93086200
C	-1.23078900	2.46876800	0.06057000
H	0.50403600	3.09330700	1.18560200
C	-0.78926900	1.56589300	-2.13230000
H	1.28484600	1.47359900	-2.71265200
C	-1.69816700	1.92203600	-1.13491700
H	-1.93766800	2.75258300	0.83438000
H	-1.14686000	1.15550300	-3.07123200
H	-2.76095300	1.78782400	-1.29264600
C	-4.08962800	-1.69347100	-0.58793300
C	-3.64133400	-2.82931100	-0.67402200
C	-4.54658300	-0.35636700	-0.50758700
C	-4.97944100	0.31769500	-1.67273100
C	-4.56812800	0.32948100	0.72089800
C	-5.41026400	1.62958900	-1.60694600
H	-4.97108100	-0.20636900	-2.62275200
C	-4.99056600	1.65204900	0.79073500
H	-4.24799100	-0.18007800	1.62166900
C	-5.41589500	2.31182000	-0.37468800
H	-5.74979100	2.15896200	-2.49052600
H	-4.99692200	2.15562700	1.74951600
C	-3.38350500	-4.26310100	-0.79356100
H	-2.79266300	-4.47911000	-1.69066900
H	-2.82968900	-4.64517200	0.07119700
H	-4.32689200	-4.81596700	-0.86641500
O	-5.84590800	3.58954800	-0.41835700
C	-5.88938100	4.33929600	0.79215700
H	-6.26729300	5.32370200	0.51648800
H	-6.56576300	3.87662700	1.52031400
H	-4.88884100	4.43988200	1.23055100
Br	-1.17844400	-2.01305600	-2.49122400

Cartesian coordinates of the optimized geometry for **33e** at B3LYP-D3BJ/6-31G(d),def2-TZVP level of theory (number of imaginary frequencies = 1):

C	-0.96547600	-1.52171100	-0.89850900
C	-1.45336900	-1.63933700	0.27712700
Au	0.96126000	-0.73721900	-0.52630600
P	3.07124800	0.00898000	0.14506300
C	3.21597300	-0.47380300	1.97944400
C	4.39256300	-0.75622600	-0.98465900
C	1.98085200	0.14214600	2.66841200
H	1.04600600	-0.26437300	2.27553200
H	2.02134100	-0.08601600	3.73983300
H	1.95808500	1.23100600	2.55665200
C	4.47316900	0.04288200	2.69834500
H	4.46365500	-0.34325600	3.72448800
H	5.40193700	-0.29221000	2.23271000
H	4.48445800	1.13297100	2.76068900
C	3.14971600	-2.00709900	2.08989200

H	2.28557900	-2.41689800	1.55636500
H	4.05523500	-2.48606400	1.70649000
H	3.05354000	-2.28238200	3.14660700
C	3.94609800	-2.18751500	-1.34819300
H	2.98030800	-2.19029300	-1.86293700
H	4.69026000	-2.62746800	-2.02216100
H	3.86513100	-2.83624300	-0.47279000
C	5.80859100	-0.82153900	-0.39084200
H	5.85461000	-1.46497400	0.49158300
H	6.48020200	-1.25210100	-1.14275400
H	6.20817600	0.16093600	-0.13109300
C	4.39973200	0.09588600	-2.26845800
H	3.40040500	0.15713500	-2.71345700
H	4.75985300	1.11186700	-2.08734500
H	5.06182900	-0.37573000	-3.00350200
C	3.28463300	1.83501400	0.11579100
C	4.52586900	2.37154900	0.50826500
C	2.23371900	2.72711500	-0.20571700
C	4.73048600	3.74352500	0.61585000
H	5.34759800	1.70980200	0.74462100
C	2.45189800	4.10738200	-0.06601600
C	3.67993900	4.61818000	0.34284700
H	5.70194800	4.12255100	0.91730300
H	1.63887700	4.78303400	-0.31279000
H	3.81909000	5.69115700	0.43217900
C	0.90585000	2.32802000	-0.75776300
C	0.80141700	1.94512200	-2.10527000
C	-0.26302300	2.44421200	0.00706000
C	-0.44421700	1.67441100	-2.67135400
H	1.70122900	1.88382700	-2.70945000
C	-1.50966200	2.16746600	-0.56025300
H	-0.18842700	2.75093500	1.04647100
C	-1.60118600	1.78451200	-1.89842500
H	-0.51026000	1.38118200	-3.71434100
H	-2.41489500	2.26431900	0.03268700
H	-2.57077200	1.57702500	-2.33462400
C	-4.05129300	-1.77706200	0.07142200
C	-3.55480700	-2.89333600	0.07996100
C	-1.42234500	-1.50393700	1.69378200
C	-1.80520800	-0.28441400	2.29027500
C	-0.96293700	-2.56520900	2.50149700
C	-1.73183800	-0.13795600	3.67112000
H	-2.13274200	0.52854000	1.65574400
C	-0.89059300	-2.40533600	3.88000200
H	-0.65058800	-3.49187400	2.03185700
C	-1.27803500	-1.19504300	4.46596500
H	-2.02033300	0.80254900	4.13011800
H	-0.53027900	-3.21943700	4.50080000
H	-1.21914300	-1.07443100	5.54336100
C	-4.56205300	-0.45649200	0.06312300
C	-4.91544100	0.18784600	1.26293500
C	-4.70901400	0.24672500	-1.15393200
C	-5.38464700	1.49801200	1.26176600
H	-4.81858200	-0.34930900	2.20030000
C	-5.18298300	1.54473700	-1.16269300
H	-4.43998200	-0.24612600	-2.08192000
C	-5.52239300	2.18564400	0.04504200
H	-5.65468000	1.96748100	2.19963900
H	-5.30898100	2.09535500	-2.08881200
C	-3.14728000	-4.29379100	0.06267400
H	-4.02238300	-4.94791600	-0.02686100
H	-2.48288000	-4.49368100	-0.78495500
H	-2.62130800	-4.56135400	0.98619400
O	-5.97208400	3.45226400	-0.07432900
C	-6.36860000	4.15194000	1.10182900
H	-6.70284100	5.13261100	0.76353200

H	-7.19322700	3.63731600	1.60873100
H	-5.52656400	4.27029300	1.79474300
Br	-1.62933400	-1.88963900	-2.59961900

Cartesian coordinates of the optimized geometry for **44e** at B3LYP-D3BJ/6-31G(d),def2-TZVP level of theory (number of imaginary frequencies = 1):

C	-0.60580800	1.09550300	-0.83419900
C	-1.75933700	1.69433000	-0.81452000
Au	1.10275800	0.16084100	-0.69140300
P	3.02884700	-1.21385900	-0.55649600
C	2.41400300	-2.96286500	-0.15552900
C	3.99017800	-1.06027500	-2.18896100
C	1.52725400	-2.81101300	1.09796500
H	0.64530800	-2.19281400	0.90477600
H	1.18117000	-3.80382600	1.40785300
H	2.08174700	-2.37392800	1.93468600
C	3.52481500	-3.97822400	0.15715300
H	3.05745700	-4.95410000	0.33318600
H	4.23535900	-4.09989600	-0.66292400
H	4.07461300	-3.71102500	1.06217600
C	1.55828000	-3.46905800	-1.33001100
H	0.77836200	-2.75217900	-1.60837900
H	2.16262800	-3.68756700	-2.21497500
H	1.06449900	-4.40085600	-1.03146400
C	2.97220100	-0.95138800	-3.34334400
H	2.32740700	-0.07414200	-3.23325800
H	3.52148200	-0.84914200	-4.28631300
H	2.33314500	-1.83358400	-3.42508700
C	4.95176400	-2.21965800	-2.49462100
H	4.42462700	-3.16907700	-2.61869600
H	5.46293100	-2.00527000	-3.44037500
H	5.72570700	-2.34410200	-1.73481700
C	4.77317800	0.26402700	-2.09803000
H	4.11258700	1.10675900	-1.86274900
H	5.56203000	0.22578200	-1.34282000
H	5.23823400	0.46816100	-3.06913900
C	4.16781900	-0.75855000	0.81087000
C	5.37773500	-1.46737300	0.94077600
C	3.86620800	0.24672900	1.75903900
C	6.26653800	-1.21265900	1.97998300
H	5.63082400	-2.23767300	0.22557900
C	4.76843500	0.47783000	2.81000400
C	5.95548400	-0.23849900	2.92751500
H	7.19166900	-1.77614600	2.04911800
H	4.52623400	1.24855200	3.53492400
H	6.63488200	-0.03211100	3.74867000
C	2.66059600	1.12807400	1.72975100
C	2.65380600	2.28831800	0.93632700
C	1.57381700	0.88215800	2.58035200
C	1.57969200	3.18092100	0.99600600
H	3.50580600	2.49897000	0.29646000
C	0.49915200	1.77242700	2.63292400
H	1.57681900	-0.00759200	3.20285700
C	0.50222100	2.92510200	1.84684100
H	1.58605000	4.07684200	0.38366800
H	-0.33979400	1.56323500	3.28917800
H	-0.32988200	3.61954000	1.88933100
C	-3.02346000	0.90121400	-0.89028600
C	-3.33573200	-0.10751600	-0.04439100
C	-4.64126000	-0.77035500	0.06200000
C	-4.75423300	-2.16558700	0.15644700
C	-5.82487200	-0.00403900	0.09393300
C	-5.99531100	-2.78769500	0.24039700
H	-3.85613700	-2.77426700	0.15624000

C	-7.06509600	-0.61018300	0.18900900
H	-5.76251200	1.07889000	0.06797100
C	-7.16401000	-2.01066000	0.25621700
H	-6.04423500	-3.86796900	0.29845600
H	-7.97730300	-0.02472000	0.22634000
C	-1.78739200	3.18248800	-0.71726900
C	-0.91045700	3.97560800	-1.46823100
C	-2.66818500	3.77562700	0.19791800
C	-0.90826800	5.35949200	-1.29333500
H	-0.25187800	3.50858800	-2.19351400
C	-2.66618800	5.15893500	0.35970100
H	-3.33774600	3.14961300	0.78020200
C	-1.78158400	5.95150700	-0.37933700
H	-0.23314900	5.97534900	-1.87955300
H	-3.35278900	5.61999000	1.06304200
H	-1.78242900	7.02918500	-0.24906600
C	-3.92937300	1.34413100	-2.01851500
H	-4.36745100	2.32840300	-1.81812800
H	-3.34507100	1.43517500	-2.94131800
H	-4.73531100	0.62898900	-2.18537600
O	-8.41687300	-2.50698400	0.34387600
C	-8.58708700	-3.91828800	0.43100800
H	-8.19035700	-4.42102700	-0.45925700
H	-8.10176300	-4.32108100	1.32812600
H	-9.66282600	-4.08297600	0.49339100
Br	-2.03541900	-0.76602400	1.23054000

Cartesian coordinates of the optimized geometry for **45e** at B3LYP-D3BJ/6-31G(d),def2-TZVP level of theory (number of imaginary frequencies = 0):

C	-0.90382000	-1.49242600	0.83734900
C	-2.03925100	-2.09173000	0.74207600
Au	0.75077300	-0.38638500	0.72419500
P	2.54327300	1.11049700	0.50104100
C	1.89384900	2.59127500	-0.49341400
C	3.18001000	1.54941100	2.23932200
C	1.32425800	1.99645800	-1.79766000
H	0.51170500	1.28830900	-1.60975700
H	0.93220800	2.81241400	-2.41686800
H	2.09840300	1.48295700	-2.37614600
C	2.96049600	3.63320600	-0.86576900
H	2.47534000	4.44023900	-1.42767200
H	3.43907100	4.08390700	0.00560000
H	3.73378800	3.20689800	-1.50887000
C	0.75627700	3.26138300	0.29743500
H	-0.01199700	2.54124900	0.59891400
H	1.12166300	3.77381800	1.19187300
H	0.27677300	4.01496200	-0.33870200
C	1.96686000	1.60112300	3.19240600
H	1.45177200	0.63732200	3.24334600
H	2.32241800	1.84849800	4.19938600
H	1.23680000	2.36046000	2.90137900
C	3.94021000	2.88007000	2.35777800
H	3.30275400	3.73815300	2.12896400
H	4.27817300	2.99381100	3.39446600
H	4.82923200	2.92669200	1.72615400
C	4.08842300	0.38062000	2.66827800
H	3.57735300	-0.58383500	2.56616300
H	5.01284100	0.34120400	2.08722900
H	4.35281800	0.50639400	3.72442300
C	3.96155600	0.45672100	-0.46832600
C	5.13626700	1.22925200	-0.54503700
C	3.91868200	-0.77971700	-1.15479700
C	6.24313300	0.80960000	-1.27548000
H	5.19058900	2.17787000	-0.02889700

C	5.04284600	-1.18201800	-1.89393400
C	6.19501800	-0.40455100	-1.95875300
H	7.13371700	1.42920600	-1.31038700
H	4.99948400	-2.13127200	-2.41864000
H	7.04864300	-0.74615400	-2.53597100
C	2.76221600	-1.72719600	-1.16159900
C	2.65523200	-2.72233100	-0.17749700
C	1.83644100	-1.71265800	-2.21420500
C	1.63180600	-3.67208100	-0.23926500
H	3.38493300	-2.75623300	0.62613600
C	0.81401400	-2.66059900	-2.27221000
H	1.92440400	-0.95797500	-2.98955500
C	0.70746400	-3.64135200	-1.28534000
H	1.56866600	-4.44106900	0.52537500
H	0.10406600	-2.63791700	-3.09310800
H	-0.08944800	-4.37661900	-1.33231300
C	-2.62327900	-0.69040200	0.88260000
C	-2.77563200	0.13840300	-0.21221700
C	-3.43503300	1.43581300	-0.22348200
C	-2.91147300	2.50835200	-0.97295200
C	-4.62272100	1.66138900	0.51213500
C	-3.50611800	3.76150200	-0.95902100
H	-2.01242400	2.35866100	-1.55952000
C	-5.24088800	2.89633700	0.50643300
H	-5.08662500	0.84572700	1.05278500
C	-4.68374300	3.96622400	-0.21868300
H	-3.06157600	4.56661700	-1.53069400
H	-6.16537600	3.06495700	1.04749800
C	-2.73314400	-3.36311900	0.65526600
C	-2.03535800	-4.56126900	0.88360500
C	-4.09462600	-3.39883200	0.32603400
C	-2.69769400	-5.77908700	0.77116300
H	-0.98537900	-4.52288700	1.15510800
C	-4.75143900	-4.62230200	0.21531900
H	-4.62623000	-2.46924300	0.14555900
C	-4.05535200	-5.81236900	0.43631700
H	-2.15886300	-6.70444600	0.95034600
H	-5.80519200	-4.64752000	-0.04407100
H	-4.56911400	-6.76491600	0.35173900
C	-3.09482700	-0.31204100	2.27110800
H	-4.09744000	-0.70676000	2.47419100
H	-2.41913700	-0.75072600	3.00846800
H	-3.11677400	0.77114300	2.41437600
O	-5.35181800	5.13065000	-0.14872600
C	-4.86285000	6.25356400	-0.88134000
H	-4.84556000	6.04373200	-1.95686300
H	-5.56199400	7.06405600	-0.67739800
H	-3.86026300	6.53848800	-0.54174800
Br	-2.06190600	-0.40417500	-1.90003800

Cartesian coordinates of the optimized geometry for **46e** at B3LYP-D3BJ/6-31G(d),def2-TZVP level of theory (number of imaginary frequencies = 1):

C	0.80591200	0.77786300	0.79721700
C	1.60368800	1.76342800	0.72395800
Au	-1.01612300	-0.10554800	0.60116300
P	-3.07374100	-1.20161700	0.35684600
C	-2.79009900	-2.74535500	-0.70652100
C	-3.75127300	-1.56831500	2.09893600
C	-2.09808400	-2.24230900	-1.98990300
H	-1.14093500	-1.75597600	-1.77659100
H	-1.90828700	-3.09740000	-2.64927700
H	-2.73201300	-1.53531100	-2.53426500
C	-4.06769400	-3.49741000	-1.11279800
H	-3.77916000	-4.37605400	-1.70185600

H	-4.64370600	-3.85366500	-0.25635100
H	-4.71649600	-2.88186800	-1.74008400
C	-1.83955900	-3.69422000	0.04421000
H	-0.93317400	-3.17943000	0.38077700
H	-2.31946200	-4.15566100	0.91179800
H	-1.53692200	-4.50245100	-0.63176400
C	-2.56206400	-1.94069700	3.00939500
H	-1.83450000	-1.12626200	3.07402600
H	-2.94178300	-2.13796700	4.01874600
H	-2.03773200	-2.83704100	2.66990400
C	-4.79542700	-2.69287700	2.18738300
H	-4.38415500	-3.66045700	1.88814200
H	-5.11666100	-2.78531900	3.23161300
H	-5.69079600	-2.49432500	1.59556300
C	-4.35704100	-0.24516400	2.60675900
H	-3.64115900	0.58107500	2.52516500
H	-5.26198900	0.03073500	2.06021300
H	-4.61745800	-0.35774200	3.66550300
C	-4.35399400	-0.21355800	-0.51849900
C	-5.66420300	-0.72529900	-0.59194300
C	-4.09131800	1.05300100	-1.09046600
C	-6.69580200	-0.01923100	-1.20136100
H	-5.88729500	-1.69312700	-0.16545600
C	-5.14666300	1.75171000	-1.70029000
C	-6.43559400	1.23168600	-1.75875600
H	-7.69380500	-0.44473900	-1.23754700
H	-4.93438800	2.72282500	-2.13638500
H	-7.22868200	1.79816200	-2.23706400
C	-2.76536200	1.74391600	-1.11994400
C	-2.47169400	2.74774800	-0.18427900
C	-1.86399900	1.51130100	-2.16943700
C	-1.29858900	3.49649800	-0.29360500
H	-3.17396600	2.94431000	0.62053000
C	-0.69458700	2.26382600	-2.27907400
H	-2.09338500	0.75085000	-2.90892000
C	-0.41099200	3.26032400	-1.34458900
H	-1.08285300	4.27124500	0.43630600
H	-0.00930400	2.07703900	-3.10015500
H	0.49537400	3.85014200	-1.43458800
C	2.25350800	0.17095600	0.98622200
C	2.92270200	-0.36089600	-0.10217800
C	4.21781600	-1.00801600	-0.08212100
C	4.53354900	-2.08888700	-0.93008600
C	5.22903200	-0.53083600	0.78907100
C	5.77585000	-2.70251400	-0.88779500
H	3.78344700	-2.46050000	-1.61873600
C	6.47704300	-1.11822800	0.82032200
H	5.03777600	0.33751500	1.40832100
C	6.76281800	-2.22064600	-0.00873000
H	5.97494200	-3.54401500	-1.53941700
H	7.26127000	-0.74237900	1.46812400
C	2.32863600	2.99351600	0.70548000
C	2.00657900	3.99559100	1.64023200
C	3.33514400	3.22058000	-0.25260100
C	2.67173000	5.21794800	1.59814000
H	1.23437800	3.80551200	2.37826500
C	4.00090800	4.44033200	-0.27631900
H	3.57562400	2.44113900	-0.96803800
C	3.66846000	5.44000200	0.64480800
H	2.41901500	5.99432000	2.31331500
H	4.77704500	4.61731900	-1.01414100
H	4.18968300	6.39197700	0.61967800
C	2.65835100	-0.10740300	2.41995200
H	3.18451000	0.74315900	2.86832300
H	1.75141200	-0.27682500	3.00935600
H	3.29769600	-0.98890500	2.50149900

O	8.00072600	-2.72964500	0.10396200
C	8.37055700	-3.84066300	-0.71365100
H	8.31416000	-3.58133100	-1.77685700
H	9.40155400	-4.07051300	-0.44640100
H	7.73403900	-4.70860700	-0.50763500
Br	2.07064800	-0.25760000	-1.81379400

Cartesian coordinates of the optimized geometry for **18f** at B3LYP-D3BJ/6-31G(d),def2-TZVP level of theory (number of imaginary frequencies = 0):

C	-0.22554500	0.33346500	-0.52335200
C	-0.81111900	1.55325600	-0.29201900
C	-0.96982300	-0.73094400	-1.21350300
Cl	0.06444800	2.87103600	0.46729700
C	-1.76748800	-0.48182500	-2.34978400
C	-0.87252900	-2.05655200	-0.74239200
C	-1.58316600	-3.08692300	-1.35463300
C	-2.37411700	-2.82301100	-2.47519200
C	-2.45197600	-1.51894600	-2.97746900
H	-3.04213400	-1.31237600	-3.86512700
H	-1.81831900	0.52069600	-2.76094100
H	-0.24271000	-2.26321900	0.11757400
H	-1.50457000	-4.09862500	-0.96826100
Au	1.75096400	-0.02365900	-0.00934000
H	-2.90977000	-3.62854100	-2.96801400
P	4.00404900	-0.40634100	0.49582000
C	-2.93534400	1.13815300	0.30538100
C	-2.23244100	1.86713200	-0.50022900
C	-3.57074200	0.20431000	1.07975800
C	-3.91141400	0.49128000	2.43810800
C	-3.87503500	-1.07570000	0.51668900
C	-4.52486900	-0.48035700	3.20292500
H	-3.67541700	1.46852300	2.84484500
C	-4.49834300	-2.02836300	1.30300800
H	-3.62756700	-1.27001700	-0.51915500
C	-4.81732400	-1.73403700	2.63610500
H	-4.78696800	-0.27899500	4.23598900
H	-4.73935300	-3.00101500	0.88791900
H	-5.30627500	-2.48878500	3.24501900
Cl	-2.83063900	3.07653300	-1.59752000
C	4.85333900	-1.54435000	-0.66786400
H	5.90472600	-1.67003100	-0.38919200
H	4.35809900	-2.51936500	-0.65604300
H	4.79559300	-1.14094000	-1.68274300
C	4.29986000	-1.13592100	2.15497300
H	5.37155800	-1.27631800	2.33000100
H	3.89328800	-0.47390400	2.92470400
H	3.79411300	-2.10288000	2.22762400
C	5.02027300	1.12220900	0.47616500
H	4.62817500	1.83261700	1.20938300
H	6.06493800	0.89655100	0.71407600
H	4.96776500	1.58492000	-0.51337800

Cartesian coordinates of the optimized geometry for **21f** at B3LYP-D3BJ/6-31G(d),def2-TZVP level of theory (number of imaginary frequencies = 0):

C	-0.93689000	-1.65620000	0.13671100
C	0.22387700	-1.24036200	0.26028200
Au	-1.07185700	0.65028700	0.13662900
C	1.60239500	-1.02057000	0.51623700
C	2.49668400	-0.63520100	-0.43390900
C	3.94571800	-0.47367200	-0.29319100
C	4.59747800	0.62758200	-0.87705900
C	4.70360500	-1.43222800	0.40322900

C	5.97211400	0.78278100	-0.73600500
H	4.02250400	1.36149300	-1.43149800
C	6.08114200	-1.27849000	0.52561400
H	4.21805000	-2.30538900	0.82228700
C	6.71716800	-0.16899000	-0.03502700
H	6.46441600	1.64271200	-1.17896100
H	6.65913600	-2.03063800	1.05296400
H	7.79164600	-0.05088600	0.06525800
P	-1.80367000	2.83473000	0.06969400
C	-0.47550000	3.99969800	-0.41472500
H	-0.86362400	5.02300400	-0.44822400
H	0.34284300	3.94776300	0.30867500
H	-0.08815500	3.72730400	-1.40030800
C	-3.15807200	3.09935000	-1.13473200
H	-3.46128400	4.15148000	-1.13705700
H	-2.82075900	2.81852900	-2.13623100
H	-4.01673100	2.47693100	-0.86846900
C	-2.43862800	3.44460600	1.67540600
H	-1.65357000	3.37394100	2.43328000
H	-2.76060100	4.48715300	1.58408800
H	-3.28635800	2.83164100	1.99365500
C	-2.17953000	-2.33054600	-0.07466800
C	-3.07538400	-2.51619100	0.99620500
C	-2.49787100	-2.81408200	-1.35882900
C	-4.27203000	-3.19072900	0.77993500
H	-2.81741300	-2.14244500	1.98180200
C	-3.69955600	-3.48479500	-1.55946600
H	-1.79810900	-2.66724300	-2.17484900
C	-4.58521400	-3.67347200	-0.49431500
H	-4.96039900	-3.34375900	1.60480500
H	-3.94492900	-3.86495800	-2.54585800
H	-5.52038200	-4.20007900	-0.65699100
Cl	1.83515800	-0.28191900	-2.02415200
Cl	2.05286900	-1.20990900	2.20076100

Cartesian coordinates of the optimized geometry for **22f** at B3LYP-D3BJ/6-31G(d),def2-TZVP level of theory (number of imaginary frequencies = 0):

C	0.27266800	-0.58937800	0.68963200
C	1.08128800	0.52222500	0.71956200
Au	-1.70410400	-0.57420700	0.11085100
C	2.40121200	0.49312600	1.41647200
C	3.29968600	0.04681100	0.61130300
C	4.14897200	-0.48375400	-0.32784200
C	4.87058200	0.37457500	-1.21195100
C	4.31264300	-1.90036100	-0.41349700
C	5.72679200	-0.17655000	-2.14644700
H	4.73240900	1.44735600	-1.13276300
C	5.17374300	-2.42886700	-1.35758900
H	3.75247300	-2.53268300	0.26583600
C	5.87737800	-1.57124700	-2.21756200
H	6.28287200	0.46361600	-2.82285500
H	5.30868100	-3.50251400	-1.43295900
H	6.55469400	-1.99497700	-2.95307600
P	-3.95850300	-0.49580200	-0.48931800
C	-5.03887700	0.18850500	0.82721500
H	-6.08115700	0.22172900	0.49340800
H	-4.96644900	-0.43530300	1.72248600
H	-4.70912500	1.19928500	1.08331700
C	-4.28037900	0.56529600	-1.95255200
H	-5.35127100	0.60413100	-2.17704300
H	-3.91445300	1.57712500	-1.75616300
H	-3.74564300	0.16653800	-2.81931900
C	-4.70341100	-2.11899900	-0.91051700
H	-4.62421500	-2.79174500	-0.05195800

H	-5.75778100	-2.00212800	-1.18153100
H	-4.16274400	-2.56571000	-1.74963900
C	0.68647000	1.79060300	0.06766400
C	0.20060000	1.78747700	-1.25245300
C	0.80028400	3.01437800	0.74840300
C	-0.18954400	2.97637300	-1.86417700
H	0.15220000	0.85085000	-1.79873800
C	0.40855300	4.20144800	0.13258000
H	1.17359300	3.03417000	1.76672600
C	-0.09027900	4.18610300	-1.17169300
H	-0.55734200	2.96129000	-2.88588400
H	0.48952400	5.13904200	0.67378900
H	-0.39206200	5.11317600	-1.64947800
Cl	0.90994900	-2.10347200	1.31497900
Cl	2.62341800	0.96109000	3.07732200

Cartesian coordinates of the optimized geometry for **23f** at B3LYP-D3BJ/6-31G(d),def2-TZVP level of theory (number of imaginary frequencies = 0):

C	0.21605900	-0.14977800	-0.00195300
C	0.91723400	1.00744700	-0.04188300
Au	-1.74321600	-0.70809400	0.03806900
C	2.37264000	0.91433800	-0.04639900
C	2.92839300	-0.32194300	-0.01834400
C	4.27021500	-0.87734800	-0.00142800
C	4.59801800	-1.99595400	-0.78905900
C	5.25368500	-0.28628800	0.81281800
C	5.89077800	-2.50790900	-0.76712200
H	3.84704900	-2.44895100	-1.42979400
C	6.54660200	-0.80083600	0.81983500
H	4.99947500	0.56214400	1.43738300
C	6.86737200	-1.90988600	0.03381200
H	6.13904700	-3.36813600	-1.38027900
H	7.30192800	-0.34123500	1.44908800
H	7.87630000	-2.30990400	0.04692200
P	-4.02693900	-1.17194200	0.10684300
C	-4.74363600	-1.07779200	1.79255600
H	-5.81923300	-1.28027100	1.76395300
H	-4.25599600	-1.81014100	2.44195300
H	-4.57525600	-0.08029800	2.20802900
C	-5.01573500	0.00432200	-0.89494100
H	-6.08259700	-0.23011300	-0.82103700
H	-4.84452600	1.02387900	-0.53818800
H	-4.70605800	-0.05322900	-1.94220900
C	-4.48665600	-2.83491600	-0.51553100
H	-3.99475000	-3.60347700	0.08716100
H	-5.57111200	-2.97616400	-0.46329000
H	-4.15895100	-2.94435500	-1.55308000
C	0.16066700	2.28259300	-0.09449100
C	-0.73980600	2.51692200	-1.14425700
C	0.31354700	3.24147700	0.91710800
C	-1.48039400	3.69822600	-1.17903700
H	-0.83497600	1.78377400	-1.93904100
C	-0.42780100	4.41997500	0.87622700
H	1.00321800	3.05939700	1.73516200
C	-1.32572400	4.64955700	-0.16953900
H	-2.16654600	3.88012800	-2.00056300
H	-0.30603700	5.15902100	1.66191000
H	-1.89804000	5.57164300	-0.19963300
Cl	1.55665100	-1.49943500	0.02598100
Cl	3.35349200	2.32835100	-0.18508600

Cartesian coordinates of the optimized geometry for **24f** at B3LYP-D3BJ/6-31G(d),def2-TZVP level of theory (number of imaginary frequencies = 1):

C	0.30365400	0.44095300	0.12149600
C	-0.95456500	0.49681100	0.25984600
Au	2.21408800	-0.24749300	0.09264800
C	-2.34458100	0.24335900	0.43834000
C	-3.00685800	-0.76148200	-0.19554800
C	-4.43246500	-1.09130400	-0.09496900
C	-4.83963700	-2.42795400	0.06165000
C	-5.40446200	-0.07960200	-0.18657900
C	-6.19170500	-2.73962100	0.15721600
H	-4.09481200	-3.21425600	0.12013300
C	-6.75589400	-0.40190400	-0.10936800
H	-5.10183000	0.94923800	-0.34177200
C	-7.15225400	-1.72880600	0.07091600
H	-6.49674100	-3.77224800	0.29339400
H	-7.50021900	0.38337700	-0.19508100
H	-8.20735900	-1.97579000	0.13669800
P	4.37637100	-1.11284000	0.07028000
C	4.93722500	-1.71805700	1.70814300
H	5.95268400	-2.12164000	1.63954100
H	4.92398900	-0.89638400	2.42962200
H	4.26132000	-2.50124800	2.06255400
C	4.57326400	-2.54424700	-1.05920500
H	5.60199700	-2.91764500	-1.02760300
H	3.89019100	-3.34475800	-0.76181000
H	4.33028200	-2.24335500	-2.08208100
C	5.65632400	0.09049000	-0.45756500
H	5.65657000	0.94962600	0.21899500
H	6.64673200	-0.37616200	-0.44762500
H	5.43530000	0.44433700	-1.46840300
C	-0.26947700	1.97438700	-0.16301000
C	-0.14324900	2.93097000	0.86806800
C	-0.47353600	2.38864700	-1.49942000
C	-0.20468600	4.28368000	0.56045300
H	-0.00153300	2.59130900	1.88844100
C	-0.54248500	3.74184500	-1.79367500
H	-0.57766300	1.63642700	-2.27452600
C	-0.40513500	4.68530300	-0.76545900
H	-0.10626700	5.02609700	1.34545700
H	-0.70141900	4.07054500	-2.81544400
H	-0.45926800	5.74383600	-1.00123600
Cl	-2.05718700	-1.80662400	-1.23622300
Cl	-3.12560000	1.28382500	1.61147900

Cartesian coordinates of the optimized geometry for **32f** at B3LYP-D3BJ/6-31G(d),def2-TZVP level of theory (number of imaginary frequencies = 1):

C	-0.32488900	0.26011400	-0.39633800
C	-0.69441700	1.39889100	0.04148500
C	-0.92763900	-0.85281200	-1.13162700
Cl	-0.41666400	2.81496400	0.85824600
C	-0.84985700	-2.15568700	-0.61403200
C	-1.59842000	-0.62448800	-2.34315200
C	-2.20741800	-1.68608000	-3.00960200
C	-2.15127100	-2.97737200	-2.47826100
C	-1.46960000	-3.20929600	-1.28101000
H	-1.41958600	-4.21149600	-0.86684400
H	-0.33060600	-2.32833100	0.32358300
H	-1.63820600	0.37721000	-2.75549400
H	-2.72564700	-1.50391000	-3.94596700
Au	1.76286400	0.06572800	0.04689600
C	-2.84322500	1.96494100	-0.77013300
C	-3.29137600	1.15149800	0.02942000
C	-3.69607200	0.14805900	0.94173400

C	-3.84564500	0.44928400	2.31099200
C	-3.94789500	-1.15882600	0.47477300
C	-4.23665200	-0.54767300	3.19710400
H	-3.65589200	1.45936000	2.65886900
C	-4.33969300	-2.14485100	1.37189700
H	-3.82864800	-1.38114500	-0.57923600
C	-4.48312500	-1.84267300	2.72973000
H	-4.35476100	-0.31759900	4.25112500
H	-4.53441100	-3.15071600	1.01374400
Cl	-2.69816400	3.14385200	-1.91719600
H	-4.79180300	-2.61692000	3.42555000
H	-2.62864800	-3.80048700	-3.00078600
P	4.01695400	-0.33857700	0.39117800
C	5.10676400	0.96925300	-0.28862600
H	6.15786900	0.71856700	-0.11141500
H	4.93587800	1.06867600	-1.36409000
H	4.87859500	1.92574000	0.18963400
C	4.48124500	-0.48903500	2.15810000
H	5.55352500	-0.68915000	2.25402100
H	4.23927100	0.43885700	2.68358400
H	3.91927300	-1.30617400	2.61875100
C	4.58227100	-1.89585000	-0.39289900
H	4.40108800	-1.85296000	-1.47042500
H	5.65157000	-2.04683600	-0.21205600
H	4.02411800	-2.74076800	0.01979000

Cartesian coordinates of the optimized geometry for **33f** at B3LYP-D3BJ/6-31G(d),def2-TZVP level of theory (number of imaginary frequencies = 1):

C	0.09439800	-1.05091500	0.40177900
C	0.89811900	-0.08581200	0.70782400
Au	-1.82247600	-0.32889800	-0.06196700
C	2.72400300	-1.04369700	1.74592500
C	3.27955900	-0.93408300	0.65824100
C	3.79988500	-0.68047300	-0.63118200
C	4.53479400	0.50121000	-0.86578600
C	3.55595000	-1.58154300	-1.68878500
C	5.01879100	0.76998700	-2.14170700
H	4.71547000	1.18632900	-0.04485100
C	4.04164800	-1.29733900	-2.95903700
H	2.98618100	-2.48430700	-1.49679200
C	4.77246300	-0.12523600	-3.18631700
H	5.59040400	1.67468500	-2.32270700
H	3.85639700	-1.98878500	-3.77478300
H	5.15358300	0.08757800	-4.18040400
P	-3.98562600	0.33939400	-0.58573500
C	-4.92877200	0.97858100	0.85161300
H	-5.94143500	1.26442500	0.54871000
H	-4.98795300	0.20729700	1.62456100
H	-4.41777100	1.85024200	1.26980600
C	-4.07136900	1.67140000	-1.84398300
H	-5.11355700	1.93386200	-2.05344500
H	-3.54296100	2.55679300	-1.47970700
H	-3.59181200	1.33593000	-2.76773700
C	-5.01028300	-1.02282600	-1.26145600
H	-5.07143600	-1.83456800	-0.53126300
H	-6.01983300	-0.66555900	-1.48948700
H	-4.55060800	-1.41212900	-2.17412000
C	1.23320100	1.29460700	0.73713600
C	1.60434900	1.94212800	-0.46284600
C	1.23099700	2.01306900	1.95298600
C	1.95108900	3.28869200	-0.44144400
H	1.60776100	1.37764500	-1.38840300
C	1.58303300	3.35557800	1.96094300
H	0.95373200	1.50377500	2.86978100

C	1.94351400	3.99251900	0.76628000
H	2.23058300	3.78951100	-1.36279300
H	1.57875000	3.91147900	2.89289900
H	2.21809000	5.04291900	0.77927400
Cl	0.36922800	-2.75113800	0.38525000
Cl	2.37043200	-1.38769600	3.31838400

Cartesian coordinates of the optimized geometry for **44f** at B3LYP-D3BJ/6-31G(d),def2-TZVP level of theory (number of imaginary frequencies = 1):

C	-0.25178900	0.68331100	0.16219400
C	0.93770100	1.20454300	0.15778400
Au	-2.04003200	-0.12733600	0.10494200
P	-4.17870200	-1.10915600	0.01505200
C	2.08264900	0.29020200	0.41833900
C	2.40059200	-0.82751300	-0.27935200
C	3.62198900	-1.63489400	-0.13166100
C	3.53958800	-3.03658800	-0.07618200
C	4.88210400	-1.01542200	-0.07997700
C	4.69438300	-3.79973600	0.06420400
H	2.57069200	-3.52146800	-0.13086900
C	6.03445300	-1.78584600	0.04214100
H	4.95748600	0.06326400	-0.15619800
C	5.94338700	-3.17709800	0.12240500
H	4.62048300	-4.88104800	0.12215600
H	7.00444900	-1.29965800	0.06885800
H	6.84393300	-3.77509100	0.22207000
Cl	1.30330400	-1.39470000	-1.53389300
C	1.11296800	2.66243300	-0.08790800
C	2.08687800	3.08043600	-1.00527600
C	0.29512700	3.59655800	0.56336300
C	2.24777400	4.43945700	-1.25640000
H	2.71015100	2.34582600	-1.50558800
C	0.45912800	4.95523800	0.29618400
H	-0.42977300	3.25894500	1.29710800
C	1.43235500	5.37596700	-0.61090300
H	3.01050000	4.77077600	-1.95397100
H	-0.16348100	5.68347000	0.80608700
H	1.56403200	6.43462200	-0.81137800
C	-4.87473900	-1.50399500	1.66361600
H	-4.95715300	-0.58987900	2.25814200
H	-5.86593200	-1.95679900	1.55854800
H	-4.21442800	-2.20099000	2.18716100
C	-4.20446900	-2.68395800	-0.92240200
H	-3.53135600	-3.40630000	-0.45227300
H	-5.21829100	-3.09664000	-0.94207600
H	-3.86641500	-2.50860600	-1.94749400
C	-5.41882600	-0.03224600	-0.79918000
H	-6.39289400	-0.53138700	-0.82422000
H	-5.51091900	0.90867600	-0.24968600
H	-5.10159300	0.19013600	-1.82175900
Cl	3.03116900	0.81022700	1.78761500

Cartesian coordinates of the optimized geometry for **45f** at B3LYP-D3BJ/6-31G(d),def2-TZVP level of theory (number of imaginary frequencies = 0):

C	0.23710000	-0.86491000	-0.10864300
C	1.44076900	-1.32481400	-0.13592500
Au	-1.73542700	-0.45931700	-0.01127500
C	1.53458800	0.14322500	-0.49359500
C	1.84842000	1.13144300	0.45175400
C	2.06505000	2.54489900	0.21873400
C	2.97625700	3.27314600	1.01711500
C	1.32587200	3.22543100	-0.77637200

C	3.16942300	4.62970600	0.79785900
H	3.54752400	2.76290400	1.78368000
C	1.49870200	4.59005400	-0.95975300
H	0.59965300	2.68966400	-1.37344100
C	2.42898100	5.29209900	-0.18627800
H	3.89168100	5.17411900	1.39696200
H	0.91101700	5.10924100	-1.70964200
H	2.57356900	6.35585400	-0.34765200
P	-4.03329500	-0.07600700	0.10113900
C	-4.54072200	1.58293600	-0.49624700
H	-5.62711400	1.69882100	-0.42509400
H	-4.05665800	2.35633100	0.10664900
H	-4.23333600	1.70947500	-1.53808900
C	-5.01364000	-1.26096200	-0.89782000
H	-6.08280900	-1.04152400	-0.81180900
H	-4.71471500	-1.19392200	-1.94759800
H	-4.82613700	-2.28030100	-0.54896000
C	-4.71929300	-0.20061800	1.79751900
H	-4.23673800	0.53612600	2.44577200
H	-5.79927000	-0.02098000	1.78810700
H	-4.52373700	-1.19791300	2.20133200
C	2.43599900	-2.36850000	-0.08613200
C	3.79587000	-2.05777500	-0.23028200
C	2.03017100	-3.69907900	0.11546200
C	4.74585800	-3.07293700	-0.16886800
H	4.09817100	-1.02834200	-0.39579500
C	2.98731300	-4.70553200	0.17935500
H	0.97454300	-3.92906500	0.22122500
C	4.34349700	-4.39482200	0.03691800
H	5.79853300	-2.83494700	-0.28289300
H	2.67811600	-5.73389200	0.33734000
H	5.08633700	-5.18500300	0.08393300
Cl	1.95812100	0.61137700	2.09826400
Cl	1.80647900	0.48871600	-2.21939100

Cartesian coordinates of the optimized geometry for **46f** at B3LYP-D3BJ/6-31G(d),def2-TZVP level of theory (number of imaginary frequencies = 1):

C	0.05163300	0.62152900	-0.14680400
C	-0.95085500	1.40881400	-0.23667200
Au	2.02075500	0.10284400	-0.02269200
C	-1.22506700	-0.21484600	-0.47627600
C	-2.02510900	-0.84120200	0.48024400
C	-3.00343100	-1.90112800	0.28842500
C	-4.22598200	-1.86490600	0.99508300
C	-2.73384500	-2.99697900	-0.55977900
C	-5.16497500	-2.87266500	0.82306900
H	-4.44256700	-1.03116200	1.65299700
C	-3.66577500	-4.01837600	-0.69613600
H	-1.78472600	-3.06496300	-1.07440900
C	-4.88636900	-3.95350300	-0.01897400
H	-6.11189300	-2.82084900	1.35029400
H	-3.43916400	-4.86789600	-1.33203800
H	-5.61691900	-4.74704400	-0.14171700
P	4.28398700	-0.43251000	0.12197500
C	4.63058100	-2.23156000	0.02812600
H	5.70719200	-2.41804400	0.09875800
H	4.12126000	-2.74835400	0.84628800
H	4.25788300	-2.62951100	-0.91982900
C	5.29518300	0.31651400	-1.21233600
H	6.34786000	0.03820900	-1.09746300
H	4.93495500	-0.02855300	-2.18549200
H	5.20425100	1.40566900	-1.17594100
C	5.07095400	0.12206300	1.68316900
H	4.57042400	-0.34630000	2.53521000

H	6.13179400	-0.14859500	1.69436000
H	4.97522300	1.20723900	1.77849600
C	-1.88898100	2.48969000	-0.20774500
C	-2.02140200	3.24795600	0.97097000
C	-2.67357800	2.78612700	-1.33821500
C	-2.92330700	4.30725000	1.00569100
H	-1.41466800	3.00297100	1.83586900
C	-3.57757400	3.84031300	-1.28459500
H	-2.56430600	2.19211200	-2.23930100
C	-3.70163400	4.60087300	-0.11675400
H	-3.02274700	4.90066600	1.90889200
H	-4.18376100	4.07497200	-2.15357000
H	-4.40709200	5.42533300	-0.08311000
Cl	-1.87610600	-0.25427700	2.10660000
Cl	-1.38538000	-0.69329400	-2.17825900

Cartesian coordinates of the optimized geometry for **18g** at B3LYP-D3BJ/6-31G(d),def2-TZVP level of theory (number of imaginary frequencies = 0):

C	-0.29667800	0.61249200	0.54537100
C	0.10411800	1.87781700	0.30529500
C	0.55798900	-0.30846500	1.33295500
Cl	-0.88175400	3.02063200	-0.61962100
C	1.02284700	0.02576500	2.61709200
C	0.88990400	-1.57235500	0.81347000
C	1.68295700	-2.45964200	1.54013400
C	2.14352800	-2.11310300	2.81302900
C	1.80330300	-0.86837100	3.35068200
H	2.13675100	-0.59666400	4.34812700
H	0.74634300	0.98224800	3.05005800
H	0.51863800	-1.85105800	-0.16849800
H	1.92761400	-3.43018100	1.11778900
Au	-2.14633300	-0.10442900	-0.06122900
H	2.74220600	-2.81203300	3.38939900
P	-4.24426900	-0.91233400	-0.69705000
C	2.40956800	1.87447300	0.16144800
C	1.41119900	2.50081500	0.69764300
C	3.39692500	1.09558700	-0.34898500
C	3.97584200	1.37056900	-1.63625200
C	3.87039900	-0.03239700	0.40097600
C	4.96187300	0.56548200	-2.13255700
H	3.61285800	2.22154800	-2.20218200
C	4.86680800	-0.83492600	-0.09866700
H	3.43041200	-0.23830600	1.36916000
C	5.42204100	-0.54538800	-1.37033900
H	5.41839600	0.74290600	-3.09970300
H	5.21272300	-1.68372100	0.47708000
C	1.48230700	3.72753400	1.57101000
H	0.93689400	4.54676000	1.09314100
H	0.99656200	3.51323900	2.52889800
H	2.51440300	4.03654300	1.74861900
O	6.38162100	-1.25541000	-1.94172400
C	6.93867500	-2.39956700	-1.27018100
H	7.69756200	-2.78623700	-1.94803600
H	7.39826800	-2.10017200	-0.32378100
H	6.16668900	-3.15584300	-1.10052400
C	-4.67209600	-2.56254000	-0.01278800
H	-5.66977700	-2.87367500	-0.33969900
H	-3.93700200	-3.30010300	-0.34713300
H	-4.64668100	-2.52291300	1.07986300
C	-4.46838000	-1.09919300	-2.51083900
H	-3.72985900	-1.80501500	-2.90141500
H	-5.47425400	-1.46404200	-2.74357100
H	-4.31308500	-0.13309600	-2.99937400
C	-5.63474900	0.17010100	-0.17861600

H	-5.50497100	1.16515400	-0.61348200
H	-6.59430000	-0.24482200	-0.50445700
H	-5.63550500	0.26875900	0.91058400

Cartesian coordinates of the optimized geometry for **21g** at B3LYP-D3BJ/6-31G(d),def2-TZVP level of theory (number of imaginary frequencies = 0):

C	1.55859800	-1.67297000	-0.26631100
C	0.35654400	-1.38937400	-0.38495500
Au	1.42545700	0.62774400	-0.17746600
C	-1.04529200	-1.34903900	-0.64985700
C	-1.91743400	-0.84611800	0.26853100
C	-3.35456400	-0.65009500	0.09751000
C	-3.86677400	-0.13045800	-1.11119900
C	-4.26264900	-0.95036900	1.12670000
C	-5.22550500	0.05703300	-1.28696500
H	-3.18544500	0.15570200	-1.90507100
C	-5.63091500	-0.77840500	0.95674400
H	-3.89312000	-1.33821900	2.06992500
C	-6.12476400	-0.27356100	-0.25722200
H	-5.62353800	0.46831700	-2.20818600
H	-6.30206900	-1.03565100	1.76664400
P	1.86701300	2.87858500	0.02782200
C	2.87284100	-2.20173200	-0.05663600
C	3.36060600	-2.36971300	1.25418300
C	3.67293400	-2.55929200	-1.15808700
C	4.63164500	-2.89809300	1.45292600
H	2.73320100	-2.09563300	2.09616000
C	4.94092200	-3.09044700	-0.94435100
H	3.28947400	-2.42503600	-2.16424900
C	5.42147500	-3.25890300	0.35722400
H	5.00588800	-3.03462400	2.46252300
H	5.55539300	-3.37410300	-1.79279800
H	6.41169600	-3.67358700	0.51814500
Cl	-1.27954700	-0.36448300	1.84818900
C	-1.46101200	-1.94072600	-1.98272200
H	-1.25801200	-1.25190700	-2.81140100
H	-0.89235200	-2.85673100	-2.17097200
H	-2.52458900	-2.18290900	-1.98843800
O	-7.42819900	-0.06076300	-0.52725400
C	-8.39547200	-0.37267600	0.47294200
H	-9.36243400	-0.12679800	0.03435500
H	-8.36859800	-1.43820600	0.72935600
H	-8.23786400	0.22885500	1.37588800
C	0.39181100	3.81387800	0.58153800
H	0.03466900	3.40644000	1.53132800
H	0.63798000	4.87312800	0.70903900
H	-0.40670700	3.71504000	-0.15892400
C	2.41626500	3.68863100	-1.52043500
H	2.59298500	4.75558800	-1.34932700
H	3.34012800	3.22152900	-1.87256300
H	1.64998400	3.56874700	-2.29120400
C	3.17053600	3.24215200	1.26303600
H	4.10706600	2.76345500	0.96409500
H	3.32624100	4.32301900	1.34322900
H	2.87281200	2.84698000	2.23817600

Cartesian coordinates of the optimized geometry for **22g** at B3LYP-D3BJ/6-31G(d),def2-TZVP level of theory (number of imaginary frequencies = 0):

C	0.15231300	-0.06088800	1.05427900
C	-0.41096100	1.15926000	0.88746600
Au	1.93470500	-0.67480600	0.21808200
P	3.94410600	-1.32064000	-0.77027500

C	-2.73738900	1.02289900	1.01279900
C	-1.70416800	1.59099300	1.53855900
Cl	-0.67742500	-1.24540300	2.09759700
C	0.23949700	2.21656000	0.06485600
C	-0.45399100	2.83384500	-0.98559300
C	1.55479900	2.62505400	0.34075400
C	0.16227500	3.80941200	-1.76749200
H	-1.47582600	2.53301200	-1.19973200
C	2.17101400	3.60010900	-0.44353700
H	2.08058900	2.18530500	1.18187100
C	1.47870600	4.19198800	-1.50155400
H	-0.38366800	4.27025100	-2.58538100
H	3.18702600	3.90933900	-0.21583000
H	1.95685900	4.95561600	-2.10743300
C	-3.76467200	0.31359000	0.46976700
C	-4.49925700	0.82492700	-0.64805700
C	-4.14016200	-0.95901500	1.02186200
C	-5.54727800	0.11901300	-1.18529600
H	-4.21536200	1.78656100	-1.06237100
C	-5.18579100	-1.66113700	0.48730300
H	-3.56962800	-1.34805100	1.85676900
C	-5.90273300	-1.13416200	-0.61975800
H	-6.09501700	0.51805900	-2.02938300
H	-5.49188200	-2.62383300	0.88089900
C	-1.68150600	2.62100000	2.63813300
H	-2.68578900	2.90169600	2.96200300
H	-1.12552000	2.21164800	3.48840300
H	-1.14983500	3.50915100	2.28202800
O	-6.89517000	-1.89117900	-1.06039400
C	-7.69791900	-1.46635200	-2.17626000
H	-8.42676700	-2.26168000	-2.32175000
H	-8.20947100	-0.52772600	-1.94380000
H	-7.07969200	-1.35645500	-3.07208900
C	3.77339400	-1.97033400	-2.47975500
H	4.75152500	-2.23300800	-2.89607000
H	3.13459600	-2.85796800	-2.47195200
H	3.30320700	-1.21269600	-3.11303800
C	5.13905000	0.06567100	-0.92907000
H	6.05722800	-0.26261500	-1.42712500
H	4.68215500	0.87490200	-1.50594700
H	5.38639100	0.44930800	0.06490800
C	4.86969700	-2.62829100	0.12676200
H	4.25569600	-3.53091300	0.19248300
H	5.80696500	-2.86511000	-0.38747200
H	5.09104300	-2.28907300	1.14262500

Cartesian coordinates of the optimized geometry for **23g** at B3LYP-D3BJ/6-31G(d),def2-TZVP level of theory (number of imaginary frequencies = 0):

C	-0.17518000	0.02993000	-0.00808400
C	0.33003900	1.28572000	0.03489500
Au	-2.04276600	-0.78953500	-0.01097500
P	-4.24041800	-1.56530200	-0.00428200
C	1.78060500	1.46250700	0.06830700
C	2.51662800	0.33158200	0.02326800
C	3.91953000	-0.01605000	-0.05665200
C	4.50251500	-0.93289200	0.83331600
C	4.73269800	0.58298900	-1.04336500
C	5.85514000	-1.24242400	0.76029600
H	3.89231500	-1.40045000	1.60082800
C	6.08261000	0.29296200	-1.11272100
H	4.29033100	1.27121000	-1.75618800
C	6.65835100	-0.62376200	-0.21318100
H	6.27745500	-1.95041500	1.46220500
H	6.71916300	0.74856600	-1.86319800

Cl	1.32593200	-1.08214300	-0.05595800
C	-0.63180300	2.42010500	0.05689600
C	-0.75332900	3.23233300	1.19346700
C	-1.44683900	2.66669100	-1.05718700
C	-1.67207300	4.28021700	1.21062800
H	-0.14349000	3.02895400	2.06893200
C	-2.36662500	3.71566100	-1.03573900
H	-1.34452600	2.04199700	-1.93922900
C	-2.47896700	4.52410100	0.09645900
H	-1.76214500	4.90294800	2.09538900
H	-2.98830200	3.90523200	-1.90559900
H	-3.19163000	5.34305600	0.11154000
C	2.41476700	2.82432800	0.14852200
H	2.17777400	3.40413100	-0.75064100
H	2.02161300	3.38283800	1.00276000
H	3.49909500	2.75237800	0.24493700
O	7.97718000	-0.84320900	-0.36897100
C	8.63144600	-1.76217000	0.50451600
H	9.67507400	-1.77282900	0.19117700
H	8.56399500	-1.43070700	1.54721000
H	8.20792800	-2.76852900	0.40662000
C	-5.38246200	-0.46046700	-0.92200100
H	-6.40753900	-0.84255500	-0.87836600
H	-5.35137400	0.54201800	-0.48594300
H	-5.06723900	-0.39283200	-1.96701600
C	-4.47171400	-3.22025000	-0.76200100
H	-3.88012700	-3.96102500	-0.21680800
H	-5.52684600	-3.51129400	-0.73468000
H	-4.13020700	-3.20056900	-1.80070600
C	-4.97210200	-1.70721000	1.67232400
H	-6.00936700	-2.05253300	1.61199100
H	-4.39050200	-2.41528800	2.26920200
H	-4.94439200	-0.73280100	2.16807100

Cartesian coordinates of the optimized geometry for **24g** at B3LYP-D3BJ/6-31G(d),def2-TZVP level of theory (number of imaginary frequencies = 1):

C	0.79493900	0.64585000	-0.14670800
C	-0.44594100	0.84833400	-0.31300100
Au	2.56290400	-0.35134700	-0.05992800
C	-1.86542800	0.82755800	-0.43859200
C	-2.54331900	-0.28101100	-0.01841500
C	-3.99402700	-0.44997900	0.02003700
C	-4.82394200	0.59539100	0.48069600
C	-4.60128100	-1.65542500	-0.36961700
C	-6.19767600	0.44615300	0.52645900
H	-4.37876700	1.51701200	0.83930000
C	-5.98156600	-1.81216100	-0.34012300
H	-3.98400400	-2.47836500	-0.71339700
C	-6.79323100	-0.75746700	0.10809300
H	-6.83897900	1.23902500	0.89554600
H	-6.41555900	-2.75072000	-0.66151600
P	4.54044700	-1.57265900	0.04581100
C	0.49123700	2.25779100	-0.01598100
C	0.29454800	2.82952500	1.25959900
C	0.78771200	3.08224700	-1.12215100
C	0.39894400	4.20461500	1.42162100
H	0.05744900	2.18060100	2.09635800
C	0.90436000	4.45545400	-0.94789100
H	0.92718900	2.62591200	-2.09673900
C	0.70726200	5.01399100	0.32061100
H	0.24513400	4.65101100	2.39869700
H	1.13949300	5.09392600	-1.79321400
H	0.79074900	6.08853400	0.45215000
Cl	-1.60965000	-1.67568400	0.51996700

C	-2.51760200	2.02903500	-1.08640600
H	-2.55592100	2.88424500	-0.40085000
H	-1.94112500	2.33957500	-1.96316300
H	-3.53341400	1.79665400	-1.40618100
O	-8.13847600	-0.80269000	0.18489000
C	-8.80758600	-1.99857700	-0.20821700
H	-8.61948200	-2.22782400	-1.26374300
H	-8.49924300	-2.84647600	0.41463000
H	-9.86977800	-1.80402500	-0.06055300
C	6.04415000	-0.54769000	0.27973700
H	5.96117100	0.02078100	1.21023700
H	6.93659800	-1.18058400	0.32226300
H	6.14228800	0.15768600	-0.55012900
C	4.57597100	-2.78487700	1.42219500
H	3.74649800	-3.48915400	1.31340900
H	5.52116700	-3.33750600	1.42246500
H	4.46323600	-2.26234700	2.37619200
C	4.87229600	-2.56206600	-1.46285600
H	5.80586100	-3.12401500	-1.35578300
H	4.04835600	-3.26071100	-1.63283900
H	4.94790400	-1.89893500	-2.32911500

Cartesian coordinates of the optimized geometry for **32g** at B3LYP-D3BJ/6-31G(d),def2-TZVP level of theory (number of imaginary frequencies = 1):

C	-0.01366800	0.87697500	0.37256100
C	0.01570100	1.90225200	-0.37557700
C	0.83786600	0.20558300	1.35179500
Cl	-0.59762800	2.94398900	-1.50393900
C	1.38596500	0.93110400	2.42020800
C	1.12548200	-1.16117600	1.21371200
C	1.97939900	-1.78585000	2.11803000
C	2.53135600	-1.06134600	3.17665800
C	2.22955300	0.29450800	3.32731300
H	2.65269100	0.85830100	4.15308700
H	1.15454200	1.98488400	2.52558100
H	0.71109300	-1.71379300	0.37652400
H	2.21408400	-2.83862600	1.99549600
Au	-1.93028400	-0.02538100	0.01647900
H	3.19067900	-1.55193200	3.88611600
P	-3.93733600	-1.15965000	-0.18426000
C	2.58907200	2.28427700	-0.38515500
C	2.10186100	3.33026400	0.01830800
C	3.14473200	1.02851800	-0.74460900
C	2.74776100	0.36486600	-1.92488800
C	4.05956400	0.39569800	0.11364300
C	3.23231400	-0.89629300	-2.21702500
H	2.04738400	0.84743100	-2.59896700
C	4.56240700	-0.86575400	-0.18131700
H	4.35725100	0.89233600	1.03012900
C	4.14291700	-1.52482400	-1.34648400
H	2.93416700	-1.42229500	-3.11754000
H	5.26270400	-1.33031000	0.50129400
C	1.69287800	4.65989900	0.45925400
H	0.93356900	5.08431900	-0.20674200
H	1.27971500	4.63723600	1.47437900
H	2.55233400	5.34052200	0.45959300
O	4.54848700	-2.75912300	-1.71848700
C	5.49642100	-3.43897700	-0.90145900
H	5.68549400	-4.39142300	-1.39676200
H	6.43208700	-2.87274300	-0.82528500
H	5.09526000	-3.62110700	0.10329800
C	-3.98739900	-2.68655900	0.82840400
H	-4.95351200	-3.18864500	0.71241700
H	-3.18907800	-3.36443000	0.51389300

H	-3.83529400	-2.43554400	1.88176700
C	-5.39033200	-0.17756100	0.34887300
H	-5.47245000	0.72347400	-0.26516500
H	-6.30669200	-0.76839800	0.24756800
H	-5.26833200	0.12317300	1.39309100
C	-4.31548100	-1.69977800	-1.89465900
H	-5.26745200	-2.24021900	-1.92135300
H	-4.37739900	-0.82804400	-2.55186300
H	-3.51875700	-2.35407900	-2.25913800

Cartesian coordinates of the optimized geometry for **33g** at B3LYP-D3BJ/6-31G(d),def2-TZVP level of theory (number of imaginary frequencies = 1):

C	-0.42946900	1.12639300	-0.87505700
C	0.37507500	1.20000600	0.11982300
Au	-2.09498000	-0.02481100	-0.28845900
P	-3.98949800	-1.27175500	0.20999500
C	2.64409400	1.96994600	-0.77711700
C	2.09619700	3.05506300	-0.66694500
Cl	-0.36011400	1.75072800	-2.47258600
C	0.88061700	0.94245900	1.41103400
C	1.62478000	-0.23675500	1.64989900
C	0.66742600	1.86707700	2.46048900
C	2.13451800	-0.48149300	2.91981100
H	1.78494300	-0.93639200	0.83817600
C	1.18560400	1.61073000	3.72057100
H	0.09695900	2.76860200	2.26312100
C	1.91969400	0.43834500	3.94967600
H	2.70472800	-1.38574500	3.10589100
H	1.02277900	2.31625200	4.52882000
H	2.32350400	0.24352200	4.93862000
C	3.33238800	0.72981200	-0.84533200
C	4.35325900	0.43498900	0.07488000
C	2.96117300	-0.25557300	-1.78657100
C	4.99521100	-0.80010300	0.06440700
H	4.63921200	1.18428300	0.80539200
C	3.58550600	-1.48899300	-1.79380200
H	2.17473700	-0.03816300	-2.50162000
C	4.61172900	-1.77276700	-0.87082900
H	5.78471200	-0.99477100	0.77967700
H	3.31252300	-2.25606500	-2.51049300
C	1.47422700	4.36802200	-0.57036300
H	2.18606700	5.15229600	-0.85444900
H	0.60760500	4.44029900	-1.23609500
H	1.14233700	4.57564100	0.45409700
O	5.15723700	-3.00428400	-0.96703900
C	6.23410800	-3.34364100	-0.09811500
H	6.52452000	-4.35856300	-0.36941900
H	7.08500800	-2.66727600	-0.24059700
H	5.91848800	-3.32117300	0.95224200
C	-5.10634500	-1.47225600	-1.23008400
H	-4.57409700	-1.97553800	-2.04201500
H	-5.98504600	-2.06375800	-0.95291600
H	-5.43019200	-0.48988600	-1.58488900
C	-3.62856100	-2.97814200	0.77744400
H	-4.55881600	-3.51749900	0.98403700
H	-3.06680200	-3.51105700	0.00527900
H	-3.02226400	-2.94344200	1.68684100
C	-5.03364900	-0.53924400	1.52788600
H	-5.91553300	-1.16267900	1.70836300
H	-4.45564400	-0.45574200	2.45240600
H	-5.35532400	0.46193000	1.22790300

Cartesian coordinates of the optimized geometry for **44g** at B3LYP-D3BJ/6-31G(d),def2-TZVP level of theory (number of imaginary frequencies = 1):

C	-0.81296900	0.79351300	0.21271200
C	0.20231900	1.60150300	0.22619900
Au	-2.34091900	-0.44298500	0.12341700
P	-4.17040200	-1.91276800	-0.02206600
C	1.55114300	1.02150100	0.52093300
C	2.08796600	-0.02761100	-0.15249300
C	3.48372700	-0.47047800	-0.07128300
C	3.81976500	-1.83362500	-0.03994300
C	4.53288200	0.47350400	-0.05323700
C	5.14287500	-2.25119200	0.04501300
H	3.03276800	-2.57957000	-0.06799900
C	5.85333800	0.07043900	0.01868000
H	4.30443600	1.53114500	-0.12850800
C	6.17330800	-1.29809100	0.07622100
H	5.36338700	-3.31069100	0.08296000
H	6.66369600	0.79115300	0.01734100
Cl	1.11138200	-0.96437100	-1.29336300
C	0.01919800	3.06682000	0.02107000
C	-1.02646200	3.75775000	0.64804400
C	0.90177400	3.73973100	-0.83480500
C	-1.18517500	5.12385200	0.41717400
H	-1.68460400	3.23233100	1.33301900
C	0.73921300	5.10513700	-1.05330800
H	1.70274900	3.19138000	-1.32155800
C	-0.30471900	5.79709100	-0.43092800
H	-1.98904700	5.66308200	0.90842400
H	1.42646200	5.63062900	-1.70900700
H	-0.42602600	6.86190000	-0.60358700
C	2.24930500	1.71679100	1.66824600
H	2.59319900	2.71813800	1.38713700
H	1.54761800	1.84235800	2.50078600
H	3.10315100	1.13621800	2.01944800
O	7.48768000	-1.58942600	0.14918700
C	7.88514600	-2.95762500	0.19098900
H	7.47976500	-3.45994400	1.07731500
H	7.56617800	-3.48924500	-0.71326500
H	8.97372700	-2.94686300	0.24357600
C	-4.72105300	-2.57298000	1.59694200
H	-5.01562900	-1.74733900	2.25070300
H	-5.57186100	-3.24885300	1.46320400
H	-3.90055500	-3.11721300	2.07288700
C	-3.82841300	-3.38466700	-1.06009400
H	-4.70939000	-4.03317500	-1.10330500
H	-3.56366200	-3.06735900	-2.07256600
H	-2.98851700	-3.94584200	-0.64109000
C	-5.65492600	-1.13019300	-0.76068300
H	-6.47536200	-1.85290600	-0.81682000
H	-5.96679800	-0.27823900	-0.15022000
H	-5.42146800	-0.77109900	-1.76682500

Cartesian coordinates of the optimized geometry for **45g** at B3LYP-D3BJ/6-31G(d),def2-TZVP level of theory (number of imaginary frequencies = 0):

C	0.68064100	0.95554600	-0.19929600
C	-0.13065000	1.94369000	-0.17797600
Au	2.24531400	-0.31780700	-0.12469400
P	4.09994600	-1.72457200	-0.01491600
C	-0.93766200	0.67944800	-0.61477100
C	-1.71525100	0.02112200	0.35127900
C	-2.95344900	-0.68657000	0.12653800
C	-3.33557400	-1.78194500	0.93558300
C	-3.85633800	-0.27124200	-0.88859200
C	-4.52394900	-2.45888700	0.72663400

H	-2.67313700	-2.11940800	1.72363900
C	-5.05672900	-0.91798400	-1.08275700
H	-3.63199200	0.59947100	-1.48995800
C	-5.40262900	-2.03006900	-0.28717000
H	-4.76871300	-3.30867100	1.35120700
H	-5.76074700	-0.58472800	-1.83701000
Cl	-1.14854300	0.02868700	1.99694500
C	-0.55368700	3.31552600	-0.03952400
C	0.41275800	4.32620200	0.10765800
C	-1.91702100	3.64513200	-0.04292200
C	0.00922400	5.64906600	0.25434700
H	1.46612200	4.06411900	0.10566300
C	-2.31157400	4.97221700	0.10286700
H	-2.65837400	2.85895200	-0.15052300
C	-1.35065000	5.97481500	0.25086500
H	0.75514300	6.42924900	0.36987600
H	-3.36719400	5.22502500	0.10210700
H	-1.65972700	7.00933900	0.36325600
C	-1.20085100	0.53236300	-2.10404800
H	-1.82206900	1.34575300	-2.49634500
H	-0.24388100	0.58029100	-2.62944700
H	-1.68193600	-0.41875400	-2.34587000
O	-6.58119900	-2.59889900	-0.56493300
C	-7.01847400	-3.72042400	0.20889800
H	-7.12432600	-3.44775900	1.26438400
H	-7.99009300	-3.99473700	-0.20000000
H	-6.32295000	-4.56031400	0.10544800
C	3.80108000	-3.41833100	-0.65565500
H	3.00088200	-3.89353600	-0.08138000
H	3.49242100	-3.36634500	-1.70348800
H	4.70954400	-4.02446400	-0.57738400
C	5.54207200	-1.11037300	-0.96840800
H	5.83961400	-0.12744600	-0.59255100
H	6.38608300	-1.80184000	-0.87739200
H	5.27054200	-1.00936700	-2.02291300
C	4.73553300	-1.97265500	1.68839300
H	5.61023700	-2.63115300	1.68075200
H	5.01478200	-1.00760200	2.12039600
H	3.95468900	-2.41709500	2.31183300

Cartesian coordinates of the optimized geometry for **46g** at B3LYP-D3BJ/6-31G(d),def2-TZVP level of theory (number of imaginary frequencies = 1):

C	0.59898800	0.75958500	-0.16026400
C	-0.17647600	1.75604300	0.00303100
Au	2.39797200	-0.19979400	-0.11400600
P	4.47010400	-1.26183400	-0.02207000
C	-0.84262400	0.28061000	-0.58510400
C	-1.65011100	-0.37744400	0.33641400
C	-2.97682500	-0.89777400	0.09164400
C	-3.46113400	-2.04867900	0.74882800
C	-3.85191700	-0.22083700	-0.79578200
C	-4.73533000	-2.53577000	0.50750900
H	-2.81720900	-2.57864300	1.44123200
C	-5.13079600	-0.68310900	-1.02229800
H	-3.53369000	0.70209000	-1.26503800
C	-5.58567500	-1.85466600	-0.38370900
H	-5.06526400	-3.43471100	1.01275600
H	-5.81326600	-0.15583000	-1.67955200
Cl	-1.02953300	-0.61193300	1.95477300
C	-0.85180900	3.01070800	0.12069200
C	-0.45441000	4.08562100	-0.69778800
C	-1.91206500	3.17400400	1.03325700
C	-1.10085000	5.31324400	-0.58605700
H	0.35787300	3.94625400	-1.40348500

C	-2.55530100	4.40188000	1.12903600
H	-2.21552700	2.33915000	1.65640900
C	-2.15007500	5.47128200	0.32260500
H	-0.79083200	6.14467800	-1.21098200
H	-3.37104900	4.53049500	1.83326000
H	-2.65550800	6.42875800	0.40250600
C	-1.11122900	0.26282100	-2.07670400
H	-1.54895900	1.20516700	-2.42570800
H	-0.15783600	0.13532300	-2.59997700
H	-1.77692900	-0.55345600	-2.36461300
O	-6.83864000	-2.22937700	-0.67996400
C	-7.38113500	-3.39841200	-0.06156600
H	-7.41565100	-3.28448000	1.02753200
H	-8.39326200	-3.49290400	-0.45342800
H	-6.79815800	-4.28748600	-0.32634700
C	5.66653800	-0.66614100	-1.27937600
H	6.62113100	-1.19361400	-1.18254200
H	5.26202400	-0.83391700	-2.28144800
H	5.83295200	0.40679500	-1.14885800
C	5.33116100	-1.04457400	1.58361900
H	4.71427500	-1.45104700	2.38994400
H	6.29804800	-1.55827600	1.57454200
H	5.49066400	0.02040500	1.77410900
C	4.39429800	-3.07863900	-0.27092700
H	5.39577900	-3.51719300	-0.21107900
H	3.75774700	-3.52849500	0.49613000
H	3.96311600	-3.29843100	-1.25159400

Cartesian coordinates of the optimized geometry for **54a** at B3LYP-D3BJ/6-311++G(d,p), aug-cc-pVTZ-PP level of theory (number of imaginary frequencies = 0):

C	1.56817900	0.52956100	0.00061500
C	2.81136700	0.87408900	0.00043700
Au	-0.35564600	0.15055200	0.00145600
P	-2.65406000	-0.32603000	0.00059600
C	4.07268200	0.13796400	0.00279800
C	4.17597600	-1.19774900	-0.00259700
C	-3.25521600	-1.03432600	1.57433500
H	-4.32817800	-1.22897500	1.51382400
H	-2.73144600	-1.96834000	1.78214000
H	-3.06065100	-0.33666100	2.38993900
C	-3.14310800	-1.52034100	-1.29371900
H	-2.61683500	-2.46385900	-1.14313200
H	-4.21984500	-1.69850500	-1.25240900
H	-2.87990000	-1.12648800	-2.27622500
C	-3.67909400	1.15974400	-0.29130500
H	-4.73739900	0.88986500	-0.28604200
H	-3.49234000	1.89684900	0.49070500
H	-3.42464800	1.59994300	-1.25627000
C	2.84455000	2.42262900	-0.00297500
H	1.85510500	2.88131200	-0.00434800
H	3.37591000	2.74024900	0.89583200
H	3.37674600	2.73634300	-0.90264800
C	5.46622600	-1.94222500	-0.00020600
H	5.52732600	-2.59817500	0.87462100
H	5.53511800	-2.59045700	-0.88018100
H	6.32603000	-1.27077700	0.00660700
H	4.96346100	0.75782800	0.00905300
H	3.26912500	-1.79937500	-0.00969500

Cartesian coordinates of the optimized geometry for **55a** at B3LYP-D3BJ/6-311++G(d,p), aug-cc-pVTZ-PP level of theory (number of imaginary frequencies = 0):

C	-1.69519700	-0.76075700	-0.01836900

C	-2.94405500	-1.05160000	0.02499000
Au	0.22505500	-0.26262600	-0.00397300
P	2.50661500	0.23302500	0.00738200
C	-3.35162500	0.34204500	0.45783600
C	-3.29149000	1.41093900	-0.36077000
C	2.88792900	1.95026600	-0.49299300
H	2.51501400	2.13097400	-1.50195800
H	2.40367400	2.64955200	0.18989500
H	3.96709100	2.11717300	-0.47249900
C	3.29088800	0.02214000	1.64510000
H	3.16872100	-1.00852800	1.98069300
H	4.35548500	0.25900100	1.58727300
H	2.81361500	0.68293400	2.36995400
C	3.46885100	-0.82642300	-1.13036600
H	4.52723000	-0.56029400	-1.08748400
H	3.34917100	-1.87315900	-0.84791600
H	3.10522600	-0.69670900	-2.15049000
C	-3.85668600	-2.22117000	-0.10865700
H	-3.29753300	-3.12972500	-0.32751800
H	-4.41798100	-2.35817200	0.81917900
H	-4.57656200	-2.03388600	-0.90909300
H	-3.66995800	0.43636400	1.49287200
H	-2.92570400	1.25855300	-1.37241800
C	-3.71515800	2.78674500	0.01183800
H	-2.87586900	3.48186100	-0.10157000
H	-4.49485300	3.13665600	-0.67275200
H	-4.08608900	2.84637000	1.03528100

Cartesian coordinates of the optimized geometry for **54b** at B3LYP-D3BJ/6-311++G(d,p), aug-cc-pVTZ-PP level of theory (number of imaginary frequencies = 0):

C	0.17541300	1.09442000	-0.00038400
C	1.29123300	1.74775100	-0.00081700
Au	-1.59069800	0.24286500	0.00119500
P	-3.69347500	-0.79602300	0.00128700
C	2.69481500	1.38597400	-0.00012300
C	3.14207700	0.11246400	-0.00078600
C	-4.11300300	-1.60596400	1.58517600
H	-5.10181400	-2.06558100	1.52335600
H	-3.37283400	-2.37401000	1.81355300
H	-4.10780600	-0.86779700	2.38822900
C	-3.85724100	-2.09813200	-1.27135100
H	-3.11223400	-2.87638900	-1.10060700
H	-4.85510000	-2.54024000	-1.23061800
H	-3.69272500	-1.66848000	-2.26030700
C	-5.05815300	0.37721800	-0.32295200
H	-6.01426900	-0.15066200	-0.31651500
H	-5.06984300	1.15134100	0.44538800
H	-4.91456500	0.85042300	-1.29512900
H	3.38107200	2.22395800	0.00082400
H	2.40569100	-0.68797300	-0.00213400
C	4.52529100	-0.33298700	-0.00017800
C	5.61877600	0.55256600	0.00221700
C	4.78064200	-1.71500600	-0.00211800
C	6.91689700	0.06693800	0.00258100
H	5.45640800	1.62330300	0.00387300
C	6.08294700	-2.20024600	-0.00176900
H	3.94705900	-2.40889000	-0.00394300
C	7.15406700	-1.31032100	0.00057400
H	7.75042800	0.75868500	0.00445700
H	6.26243100	-3.26833400	-0.00330500
H	8.17082300	-1.68401100	0.00087300
C	0.89391500	3.24701700	-0.00234800
H	-0.18393400	3.41526900	-0.00257500
H	1.31611800	3.69747400	0.89740100

H	1.31628900	3.69574900	-0.90287200

Cartesian coordinates of the optimized geometry for **55b** at B3LYP-D3BJ/6-311++G(d,p), aug-cc-pVTZ-PP level of theory (number of imaginary frequencies = 0):

C	-0.14703700	1.84347700	0.03855700
C	-1.03930900	2.76388200	0.02030300
Au	1.24414300	0.41680600	0.00498000
P	2.92120200	-1.19609800	-0.03359900
C	-2.03972900	1.71047400	-0.40033500
C	-2.54088600	0.79039600	0.47149300
C	2.29852500	-2.89578500	-0.29911000
H	1.60517500	-3.16220400	0.49966800
H	1.76990600	-2.94864700	-1.25178400
H	3.12865600	-3.60553600	-0.30776600
C	4.15762700	-0.92958900	-1.35430900
H	4.63327300	0.04286500	-1.22030400
H	4.91980100	-1.71129200	-1.32394000
H	3.66466200	-0.94370600	-2.32724800
C	3.88859500	-1.27824400	1.51623600
H	4.66213600	-2.04550600	1.44000200
H	4.35736800	-0.31205300	1.70749700
H	3.22692100	-1.51451800	2.35058200
C	-1.25026400	4.23011800	0.13265300
H	-0.31095800	4.74013700	0.34380500
H	-1.66829000	4.61864500	-0.79983700
H	-1.96699200	4.44160000	0.93014600
H	-2.33122500	1.72617700	-1.44534400
H	-2.17558200	0.83702500	1.49329300
C	-3.51779900	-0.23740500	0.19583600
C	-3.98882400	-1.01224300	1.27316400
C	-4.02689400	-0.48723900	-1.09446900
C	-4.94698700	-1.99636500	1.07272500
H	-3.60213100	-0.82703600	2.26909600
C	-4.98177500	-1.47097600	-1.29072600
H	-3.67024900	0.08353400	-1.94264800
C	-5.44513600	-2.22596700	-0.20879800
H	-5.30671900	-2.58196500	1.90952000
H	-5.36927100	-1.65711100	-2.28472900
H	-6.19242200	-2.99385600	-0.36875700

Cartesian coordinates of the optimized geometry for **54c** at B3LYP-D3BJ/6-311++G(d,p), aug-cc-pVTZ-PP level of theory (number of imaginary frequencies = 0):

C	1.09968200	-0.57846200	-0.00056000
C	2.37924100	-0.65914200	-0.00005800
Au	-0.86526500	-0.29837800	-0.00016200
P	-3.17802800	-0.01608500	0.00062200
C	3.53256100	-1.53228500	0.00048600
C	3.44428200	-2.87037100	0.00015100
C	-3.98349700	-0.56846600	1.54660100
H	-5.06298500	-0.41316600	1.48790700
H	-3.78028900	-1.62794200	1.70805700
H	-3.58320700	-0.00535100	2.39067600
C	-4.02487400	-0.93434500	-1.33494000
H	-3.82204600	-2.00122500	-1.23262300
H	-5.10285900	-0.76595500	-1.28552800
H	-3.65137000	-0.60084700	-2.30397100
C	-3.69486300	1.72566100	-0.21265200
H	-4.78455700	1.79941500	-0.20607300
H	-3.28817700	2.33159700	0.59803700
H	-3.31362800	2.10877400	-1.16007200
C	4.61420700	-3.79338300	0.00071900
H	4.58404600	-4.44877400	0.87765300

H	4.58522600	-4.44835700	-0.87656700
H	5.56188700	-3.25289800	0.00148500
H	4.49722500	-1.03721900	0.00118200
H	2.45843600	-3.33034600	-0.00060400
C	2.44646500	0.88631700	-0.00002500
C	2.54977700	1.57260900	-1.22007400
C	2.54890000	1.57250400	1.22016300
C	2.81325400	2.93556000	-1.21431700
H	2.43500000	1.02861200	-2.14914300
C	2.81239100	2.93544600	1.21472600
H	2.43344000	1.02842000	2.14909900
C	2.94539800	3.61259300	0.00028000
H	2.90931500	3.47286800	-2.14952500
H	2.90780600	3.47265900	2.15005600
H	3.14555400	4.67748300	0.00039900

Cartesian coordinates of the optimized geometry for **55c** at B3LYP-D3BJ/6-311++G(d,p), aug-cc-pVTZ-PP level of theory (number of imaginary frequencies = 0):

C	-0.88972200	0.17918100	0.02858400
C	-2.16434100	0.36394000	0.09808000
Au	1.07619100	-0.07750100	0.01267200
P	3.37453400	-0.47311100	-0.00611700
C	-1.97511700	1.82020200	0.47137100
C	-1.55248200	2.74684700	-0.41307500
C	4.36286000	0.95348100	-0.58390700
H	4.06520500	1.21991900	-1.59888300
H	4.18905600	1.81046800	0.06819600
H	5.42618500	0.70419100	-0.57390000
C	4.05763700	-0.89647500	1.63614200
H	3.56548700	-1.79171200	2.01848400
H	5.13215000	-1.07822400	1.56383200
H	3.87682100	-0.07653800	2.33248200
C	3.85086700	-1.86057300	-1.09784700
H	4.93276500	-2.00758700	-1.07020600
H	3.35491500	-2.77449200	-0.76834700
H	3.54177700	-1.64599500	-2.12163500
H	-2.17011100	2.06959500	1.51042100
H	-1.33938600	2.42090000	-1.42722000
C	-1.37733300	4.19073800	-0.10739000
H	-0.34085400	4.49120800	-0.29725700
H	-1.99572300	4.79319700	-0.78114800
H	-1.63142300	4.43328600	0.92468100
C	-3.42398200	-0.35889600	0.04139500
C	-3.42825100	-1.75765100	-0.07541000
C	-4.63464500	0.34059800	0.09829100
C	-4.63376500	-2.44063900	-0.13733600
H	-2.48979100	-2.29819400	-0.11296900
C	-5.83962400	-0.35182800	0.03907100
H	-4.63005400	1.42081400	0.18143000
C	-5.84062300	-1.73974400	-0.07893100
H	-4.63769400	-3.51987400	-0.22848300
H	-6.77571300	0.19089300	0.08287600
H	-6.77962400	-2.27766800	-0.12589300

Cartesian coordinates of the optimized geometry for **56a** at B3LYP-D3BJ/6-31G(d),def2-TZVP level of theory (number of imaginary frequencies = 0):

C	2.90643800	-0.08833500	-0.01787600
C	3.83534600	-1.22880100	-0.01424900
C	3.74785200	1.21937600	-0.03249200
C	5.10306100	-0.78582200	0.01662500
C	5.21596100	0.70740100	0.04542800
H	3.55132600	1.76825800	-0.95592600

H	3.47774900	1.85837300	0.81014700
H	5.81427500	1.08505800	-0.79215200
H	5.70919600	1.04871200	0.96385200
H	5.96822600	-1.44107600	0.02945100
H	3.50053300	-2.25739900	-0.02952000
Au	-0.35409200	-0.02760600	-0.00320400
P	-2.71142000	0.02385000	0.00665400
C	-3.46101000	-0.87181500	-1.40567800
H	-4.55295800	-0.81015400	-1.35610900
H	-3.11728700	-0.43219100	-2.34612300
H	-3.15925500	-1.92266100	-1.38117100
C	-3.37839600	1.72932600	-0.08206300
H	-4.47293500	1.70509300	-0.07595400
H	-3.02714900	2.31083000	0.77482400
H	-3.03477900	2.21499600	-0.99958200
C	-3.45020900	-0.71673100	1.51129600
H	-3.10081100	-0.17942500	2.39734000
H	-4.54255100	-0.66163600	1.46300800
H	-3.14685200	-1.76394100	1.59662700
C	1.61060400	-0.08121900	-0.01189400

Cartesian coordinates of the optimized geometry for **57a** at B3LYP-D3BJ/6-31G(d),def2-TZVP level of theory (number of imaginary frequencies = 0):

C	2.92990800	0.74279900	-0.40255000
C	2.86376100	-0.98504200	-0.54397300
C	4.29381600	1.10044900	0.04119300
C	3.98997500	-1.30468200	0.11699000
C	4.71727400	-0.17978100	0.81908200
H	4.95414700	1.27525500	-0.81395700
H	4.26574600	2.00450500	0.65599200
H	5.80124600	-0.31471500	0.78316400
H	4.42811700	-0.12824500	1.87792200
H	4.42808700	-2.29306400	0.01249300
H	2.39244800	-1.59953500	-1.29931000
Au	-0.26075300	0.09535600	-0.08819400
P	-2.57599300	-0.07731200	0.12248600
C	-3.22580400	0.75708800	1.61966900
H	-4.31444700	0.65236400	1.67009800
H	-2.77949000	0.31299800	2.51375600
H	-2.96745300	1.81931200	1.59254300
C	-3.17775900	-1.80566900	0.23152800
H	-4.26788900	-1.82157000	0.33177300
H	-2.89192300	-2.35516300	-0.66966800
H	-2.72969000	-2.29958600	1.09818000
C	-3.48812100	0.66708100	-1.28243900
H	-3.20629200	0.16718500	-2.21327900
H	-4.56759100	0.56490800	-1.13088400
H	-3.23530800	1.72762300	-1.36701000
C	1.75189600	0.32679900	-0.25146300

Cartesian coordinates of the optimized geometry for **58a** at B3LYP-D3BJ/6-31G(d),def2-TZVP level of theory (number of imaginary frequencies = 0):

C	4.73895000	0.14975600	-0.08534900
C	3.84860300	1.49422100	0.17775100
C	2.59852000	0.90468300	-0.17540000
C	1.86661600	-0.12695500	-0.07851200
C	2.72767700	-1.36166700	0.03080500
C	4.05650000	-1.18110500	0.10767800
H	3.89620400	1.80089000	1.22881200
H	4.23244400	2.28319400	-0.47094700
H	5.07644300	0.21276100	-1.12737500
H	5.61930600	0.25044800	0.55658500

H	4.70900900	-2.03908400	0.24884700
H	2.25009300	-2.33191200	0.08633400
Au	-0.21525500	-0.01688700	-0.02002200
P	-2.54568400	0.03539400	0.02107800
C	-3.26711400	1.20384700	-1.19318400
H	-4.36035700	1.18307200	-1.13590800
H	-2.91516900	2.21800300	-0.98496100
H	-2.95421300	0.92712300	-2.20376300
C	-3.30862800	-1.58830400	-0.35522400
H	-2.98286400	-2.32958300	0.37977200
H	-4.40035600	-1.50978700	-0.32750200
H	-2.99731500	-1.92226700	-1.34887900
C	-3.23774100	0.53211100	1.64419700
H	-2.88424100	1.53316900	1.90617500
H	-4.33198300	0.53494200	1.60494600
H	-2.90686100	-0.16732900	2.41687000

Cartesian coordinates of the optimized geometry for **56b** at B3LYP-D3BJ/6-31G(d),def2-TZVP level of theory (number of imaginary frequencies = 0):

Au	0.59165400	0.09805800	0.02688800
P	2.93804800	-0.09480100	-0.04643000
C	3.71837800	1.15877900	-1.13262700
H	4.80526600	1.02762600	-1.13898100
H	3.33677500	1.05412200	-2.15203500
H	3.48005900	2.16321800	-0.77189400
C	3.50361200	-1.71744800	-0.68489400
H	4.59759400	-1.75351000	-0.70634700
H	3.13089700	-2.52032200	-0.04277300
H	3.11833600	-1.87154500	-1.69662100
C	3.74268500	0.09940300	1.58839200
H	3.37382100	-0.66686300	2.27590600
H	4.82879300	0.00295600	1.49049900
H	3.50446200	1.08277600	2.00329100
C	-1.36988500	0.28007300	0.08386000
C	-2.65977600	0.29893100	0.14699000
C	-3.19211600	-1.13435100	0.47105400
C	-3.64512100	1.36830600	0.00048800
C	-4.44199200	-1.37232000	-0.38378000
H	-3.42949100	-1.13304600	1.54075300
H	-2.43474500	-1.90239300	0.29446300
C	-4.95361500	1.08183900	-0.09287600
C	-5.51473600	-0.31141700	-0.11419800
H	-4.82651700	-2.37265600	-0.15650700
H	-4.16362100	-1.36411900	-1.44352400
H	-6.30387300	-0.37196000	-0.87285200
H	-6.01546000	-0.50438000	0.84675500
H	-3.27987800	2.38833500	-0.02454800
H	-5.65840400	1.90641300	-0.16776500

Cartesian coordinates of the optimized geometry for **57b** at B3LYP-D3BJ/6-31G(d),def2-TZVP level of theory (number of imaginary frequencies = 0):

Au	0.46805500	0.10075600	-0.11128400
P	2.77531000	-0.07966800	0.16823000
C	3.62482500	1.54434900	0.21270700
H	4.70277300	1.40682400	0.34597000
H	3.44326300	2.08209000	-0.72199000
H	3.23347000	2.14337500	1.03965700
C	3.60747100	-1.02203300	-1.16686100
H	4.68594600	-1.06892900	-0.98402700
H	3.20501300	-2.03795800	-1.20851800
H	3.42676500	-0.53631900	-2.12978500
C	3.26207100	-0.91943400	1.72403400

H	2.85328300	-1.93356000	1.74019600
H	4.35280200	-0.96930600	1.80463000
H	2.86268600	-0.36972300	2.58083900
C	-1.54484500	0.31735100	-0.32563600
C	-2.71066200	0.79973100	-0.41252500
C	-3.99260800	1.40548500	0.00164700
C	-2.60743400	-0.76585700	-0.99857000
C	-4.90774700	0.32748500	0.65756100
H	-4.48987100	1.88215700	-0.84805700
H	-3.73887000	2.19274900	0.72095400
C	-3.54360800	-1.47434800	-0.31475700
C	-4.11688800	-0.97867800	0.97383800
H	-5.73048800	0.09171200	-0.02411600
H	-5.34966300	0.71596700	1.57929400
H	-3.31574100	-0.74601700	1.68706900
H	-4.78021300	-1.71861700	1.42593800
H	-2.36384400	-0.99279700	-2.03108600
H	-4.02672000	-2.31065200	-0.81762500

Cartesian coordinates of the optimized geometry for **58b** at B3LYP-D3BJ/6-31G(d),def2-TZVP level of theory (number of imaginary frequencies = 0):

C	1.78917800	0.62295000	0.06161800
C	1.78583700	-0.61199000	0.23211400
C	2.64029700	-1.76325500	0.23694900
C	2.69154000	1.79095500	0.01094200
C	3.91353900	-1.47384300	-0.10876700
C	4.09246700	1.15869500	0.26043700
C	4.42763400	-0.11929200	-0.57221800
H	2.45918100	2.53546500	0.77893100
H	4.19044900	0.93594100	1.32732500
H	2.63180900	2.28822000	-0.96271400
H	4.84044800	1.92079000	0.01823800
H	5.51683800	-0.18619400	-0.63823300
H	4.07680700	0.03572100	-1.60481500
H	4.64000800	-2.28343900	-0.07578000
H	2.30383900	-2.74601700	0.54010700
Au	-0.32708900	-0.00063100	0.04477600
P	-2.67302600	-0.00686800	-0.05995300
C	-3.35245800	1.62932700	-0.53036400
H	-2.96751100	1.92297400	-1.51078800
H	-4.44558900	1.58228700	-0.57199500
H	-3.05350100	2.38230200	0.20401600
C	-3.33413400	-1.18801000	-1.29475200
H	-3.02240400	-2.20508900	-1.04150900
H	-4.42789400	-1.14129800	-1.31274400
H	-2.94585700	-0.94047800	-2.28656900
C	-3.47828700	-0.43960700	1.52756500
H	-4.56743900	-0.41699700	1.41784400
H	-3.16892600	-1.44098400	1.83957200
H	-3.17851500	0.27407600	2.29997500

Cartesian coordinates of the optimized geometry for **56c** at B3LYP-D3BJ/6-31G(d),def2-TZVP level of theory (number of imaginary frequencies = 0):

Au	0.79309100	-0.13865700	-0.04206900
P	3.12712900	0.13270300	0.10195600
C	4.04102400	-1.43967700	-0.12683200
H	5.11923700	-1.26295200	-0.05760100
H	3.74458200	-2.15712200	0.64340000
H	3.80746900	-1.86383800	-1.10729600
C	3.69170700	0.80101100	1.71216200
H	4.78175600	0.90224400	1.71996300
H	3.23784100	1.78068600	1.88571800

H	3.38694600	0.12860300	2.51895600
C	3.79359400	1.27588400	-1.16680200
H	3.34244100	2.26515200	-1.05004800
H	4.88001100	1.36216100	-1.06272200
H	3.55653300	0.89907900	-2.16560300
C	-1.16029000	-0.39479800	-0.15171600
C	-2.44469700	-0.46765600	-0.26780500
C	-3.39465800	-1.54680300	0.00324200
C	-2.89764500	0.85403100	-0.96681000
C	-4.72399100	-1.38561100	0.11938900
C	-3.72100700	1.77131200	-0.05386500
H	-3.46963400	0.49651700	-1.83044600
H	-2.04331400	1.40750300	-1.37221200
C	-5.50263200	-0.09671100	0.17091700
C	-4.74647200	1.06517700	0.84139700
H	-4.22922400	2.47802900	-0.72367100
H	-3.04375000	2.36410000	0.57057400
H	-5.83341400	0.18712300	-0.84018600
H	-6.42511000	-0.29281400	0.72765600
H	-5.46824700	1.81153100	1.18926100
H	-4.24788300	0.67729900	1.73749000
H	-5.30360500	-2.29769200	0.24798600
H	-2.96334400	-2.53922700	0.08323700

Cartesian coordinates of the optimized geometry for **57c** at B3LYP-D3BJ/6-31G(d),def2-TZVP level of theory (number of imaginary frequencies = 0):

Au	-0.65326900	-0.23406300	-0.08048400
P	-2.94171400	0.21711400	0.16044900
C	-4.00197000	-0.77996200	-0.95377300
H	-5.05689900	-0.53288200	-0.79680100
H	-3.73614100	-0.57876900	-1.99527600
H	-3.84816900	-1.84434000	-0.75539500
C	-3.37896100	1.96376600	-0.18587000
H	-4.45520600	2.11750900	-0.05689800
H	-2.83697900	2.62339100	0.49747500
H	-3.10025100	2.21918200	-1.21199800
C	-3.56633400	-0.11393100	1.85201400
H	-3.02816300	0.50742800	2.57318200
H	-4.63654200	0.10897100	1.91155300
H	-3.40332900	-1.16460900	2.10767800
C	1.28317300	-0.65803900	-0.26488900
C	2.52753200	-0.86839300	-0.48893900
C	2.64298600	0.34201300	-1.42663400
C	3.70063400	-1.61754000	0.05821900
C	3.22950800	1.47884700	-0.99417200
C	4.72416700	-0.61114400	0.64855100
H	4.16398900	-2.21860400	-0.73138700
H	3.33579400	-2.30195500	0.82964700
C	3.79642200	1.78157700	0.36234600
C	4.06205400	0.60406600	1.32696700
H	5.39648500	-0.26799100	-0.14577400
H	5.34594800	-1.15623100	1.36530100
H	4.74552900	2.30447000	0.17785600
H	3.15653200	2.53693800	0.84379800
H	4.71937000	0.98290300	2.11583700
H	3.13525600	0.29788100	1.82439800
H	3.38745800	2.25282700	-1.74612000
H	2.33397600	0.22061600	-2.45846300

Cartesian coordinates of the optimized geometry for **58c** at B3LYP-D3BJ/6-31G(d),def2-TZVP level of theory (number of imaginary frequencies = 0):

C	1.62925600	-0.61596800	-0.16169900

C	1.63765300	0.62087000	-0.29536100
C	4.47336500	-0.48503000	-0.22281700
C	2.24622700	1.90997300	-0.32646700
C	4.29979700	0.79727900	0.66353800
C	3.53285500	1.96099300	0.08177500
H	4.18300800	-0.27618700	-1.25920600
H	5.28866300	1.15895600	0.95564300
H	5.54191700	-0.71614400	-0.25579400
H	3.79907800	0.50601600	1.59921500
C	2.28461900	-1.93170600	-0.21591300
H	2.28608300	-2.27651400	-1.25785500
H	1.76139800	-2.68748500	0.37675900
C	3.74405500	-1.74822400	0.29287400
H	3.73641000	-1.74101200	1.38857400
H	4.29385600	-2.64532300	-0.01107300
H	4.04475800	2.91809200	0.00663500
H	1.71623300	2.77026900	-0.71652900
Au	-0.50705000	-0.00756200	-0.06964800
P	-2.83552600	0.00318500	0.10249900
C	-3.53372800	1.69398100	-0.01076100
H	-4.62451000	1.65532700	0.07647000
H	-3.13093100	2.31721600	0.79235200
H	-3.26556800	2.14273100	-0.97115500
C	-3.66576400	-0.96235200	-1.21447200
H	-3.34689600	-2.00725800	-1.16692700
H	-4.75252700	-0.91202500	-1.09109400
H	-3.39475500	-0.55896800	-2.19404900
C	-3.45234900	-0.67986900	1.68643600
H	-3.04800000	-0.09970000	2.52050700
H	-4.54612700	-0.63946700	1.71495300
H	-3.12814100	-1.71861000	1.79447800

Cartesian coordinates of the optimized geometry for **56d** at B3LYP-D3BJ/6-31G(d),def2-TZVP level of theory (number of imaginary frequencies = 0):

C	-3.02958000	-1.61726200	-0.12174900
C	-4.36847500	-1.75273800	-0.21002600
C	-3.60823200	1.49402500	-0.74709700
C	-5.56763700	-0.85406200	-0.08141500
C	-4.99180500	1.67500600	-0.10909900
C	-5.48919900	0.46855500	0.69516200
H	-5.94470400	-0.66535600	-1.10030100
H	-3.65808100	0.76896100	-1.56553800
H	-5.71188000	1.90165500	-0.90537000
H	-3.27875100	2.43839900	-1.19077300
H	-6.34430100	-1.47676000	0.38050100
H	-4.97133800	2.55257600	0.54917000
H	-6.49790100	0.70308000	1.05206200
H	-4.88781000	0.32087400	1.60044000
C	-2.57477000	1.03245100	0.30454600
H	-2.94883200	1.10018800	1.33023000
H	-1.68431600	1.67490200	0.27406500
H	-2.45250000	-2.52412200	-0.27824700
H	-4.66424100	-2.77564400	-0.44309900
Au	1.07691800	-0.11883900	0.02646000
P	3.41511900	0.13341600	-0.01168500
C	4.26521600	-1.28940700	-0.79496200
H	5.34815500	-1.12870200	-0.79366200
H	4.03752700	-2.20663600	-0.24482100
H	3.91955300	-1.40550200	-1.82588200
C	3.96924700	1.60942200	-0.94694200
H	3.55520700	2.51414600	-0.49315600
H	5.06220300	1.67246100	-0.94072000
H	3.61797700	1.54512400	-1.98052100
C	4.16346100	0.29637600	1.65329500

H	3.93218700	-0.59018100	2.25031500
H	5.24998800	0.40351200	1.57254600
H	3.75219400	1.17384200	2.16006900
C	-2.17473400	-0.45479000	0.08394100
C	-0.88243200	-0.37587700	0.05394400

Cartesian coordinates of the optimized geometry for **57d** at B3LYP-D3BJ/6-31G(d),def2-TZVP level of theory (number of imaginary frequencies = 0):

Au	0.88455600	0.24806900	-0.07039700
P	3.15777400	-0.24568600	0.17630100
C	3.56820000	-2.00133900	-0.15881200
H	4.64129400	-2.17279600	-0.02564100
H	3.01295900	-2.64813100	0.52618200
H	3.28822300	-2.25756000	-1.18443000
C	3.78761000	0.08345500	1.86645400
H	4.85375600	-0.15762400	1.92836900
H	3.64245800	1.13823400	2.11598700
H	3.23816200	-0.52412500	2.59086100
C	4.24046300	0.72493800	-0.94028100
H	4.10596500	1.79306500	-0.74811500
H	5.29017600	0.45895500	-0.77899900
H	3.97381100	0.52320700	-1.98147500
C	-1.05178500	0.72091100	-0.25157600
C	-2.28477400	1.00146900	-0.43111900
C	-3.44872300	1.82589600	0.00973100
C	-2.31831200	-0.16925500	-1.44075700
C	-4.42795600	1.06454100	0.93679600
H	-3.98602300	2.17065000	-0.88132400
H	-3.06144500	2.71012200	0.52575300
C	-2.74734800	-1.39167800	-1.04585500
C	-5.16233100	-0.13112900	0.29053800
H	-3.89750900	0.74870800	1.84340500
H	-5.16427000	1.80784800	1.26088100
C	-3.09678000	-1.75547500	0.35423600
C	-4.61432000	-1.52963100	0.62188100
H	-6.20641200	-0.12158900	0.62217300
H	-5.20020200	0.00787800	-0.79717200
H	-2.51246400	-1.15639600	1.05758900
H	-2.86776300	-2.81057900	0.53699400
H	-4.77517800	-1.74539600	1.68482600
H	-5.19386800	-2.27312200	0.06234500
H	-2.13493800	0.06564100	-2.48404300
H	-2.95692000	-2.12540500	-1.82472000

Cartesian coordinates of the optimized geometry for **58d** at B3LYP-D3BJ/6-31G(d),def2-TZVP level of theory (number of imaginary frequencies = 0):

Au	0.69684400	0.10998300	-0.14146500
P	2.98807600	-0.11209900	0.20479200
C	3.41916500	-1.45158900	1.37855700
H	4.50450300	-1.49973200	1.51499300
H	2.94220700	-1.26294400	2.34428700
H	3.06193400	-2.41101100	0.99442800
C	3.73764800	1.40829500	0.90140900
H	4.81259300	1.26353100	1.05086400
H	3.58007600	2.24736700	0.21812400
H	3.27007000	1.64581800	1.86092900
C	3.93275400	-0.46889000	-1.32403300
H	3.77559100	0.33312800	-2.05055200
H	5.00138700	-0.54797200	-1.09896100
H	3.58660800	-1.40889000	-1.76256100
C	-1.52656100	-0.48836600	-0.66465700
C	-1.38649100	0.71234700	-0.36441800

C	-2.02646700	2.04129300	-0.19599500
C	-2.02182600	-1.80582000	-0.82964000
C	-3.42325900	1.83974700	0.44460200
H	-2.11749300	2.50673800	-1.18554600
H	-1.41493900	2.70820700	0.41787200
C	-3.08571700	-2.17295400	-0.07626700
C	-4.34361600	0.84387000	-0.30816700
H	-3.27964500	1.51523700	1.48181000
H	-3.89295000	2.82756800	0.49779700
C	-3.70766400	-1.34358500	1.00829300
C	-4.81250900	-0.36393000	0.52348200
H	-5.24171600	1.37398800	-0.64268200
H	-3.85851900	0.49237400	-1.22682400
H	-2.92760000	-0.77353900	1.52759600
H	-4.15714200	-2.01942600	1.74335600
H	-5.32898500	0.00229000	1.41926100
H	-5.55791200	-0.92376100	-0.05330500
H	-1.61911100	-2.44258900	-1.61013500
H	-3.54352400	-3.13600800	-0.29591300

5. ^{1}H and ^{13}C NMR Spectra of the ^{13}C-Labeled Products

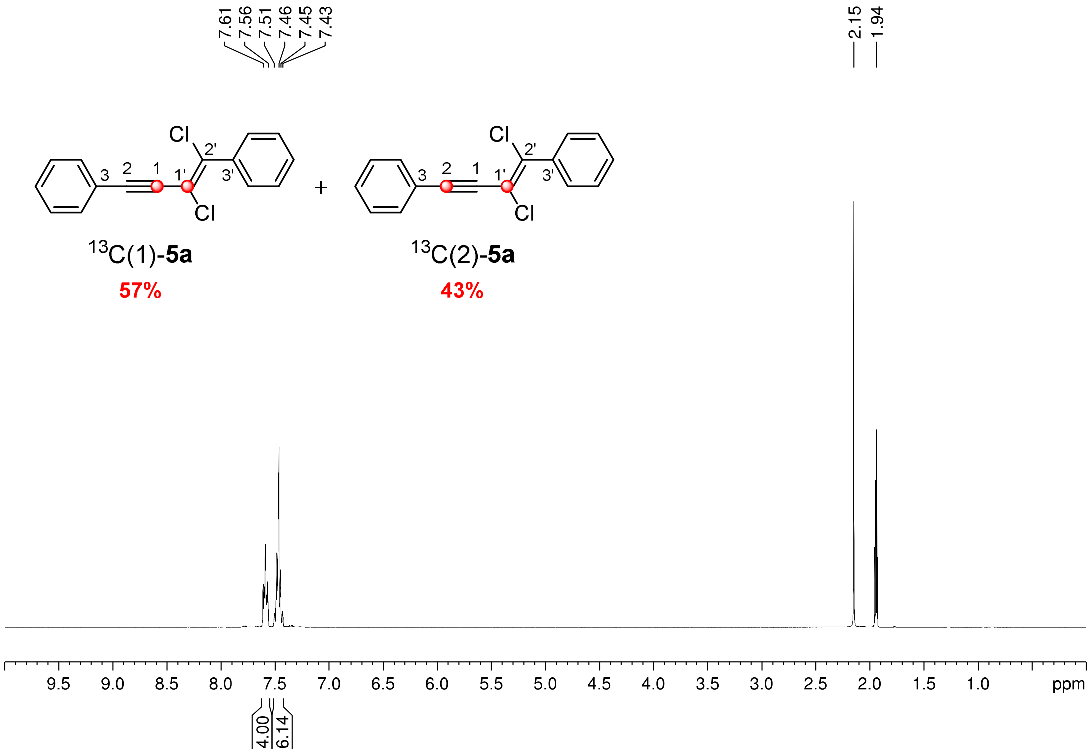

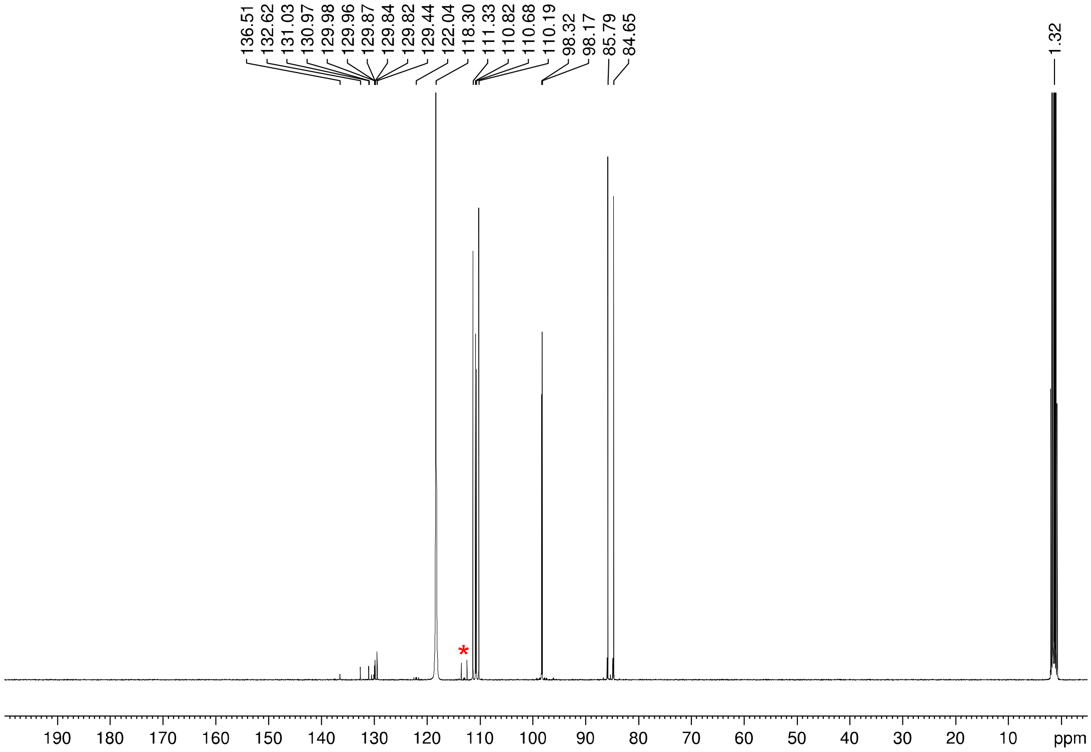

* = ^{13}C-labeled impurity

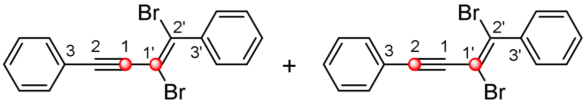

^{13}C(1)-**6a** **40%**

^{13}C(2)-**6a** **60%**

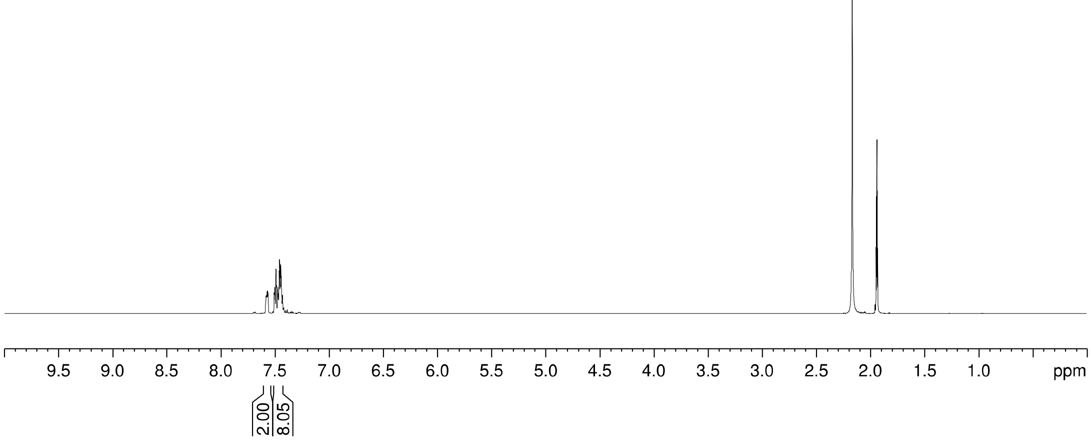

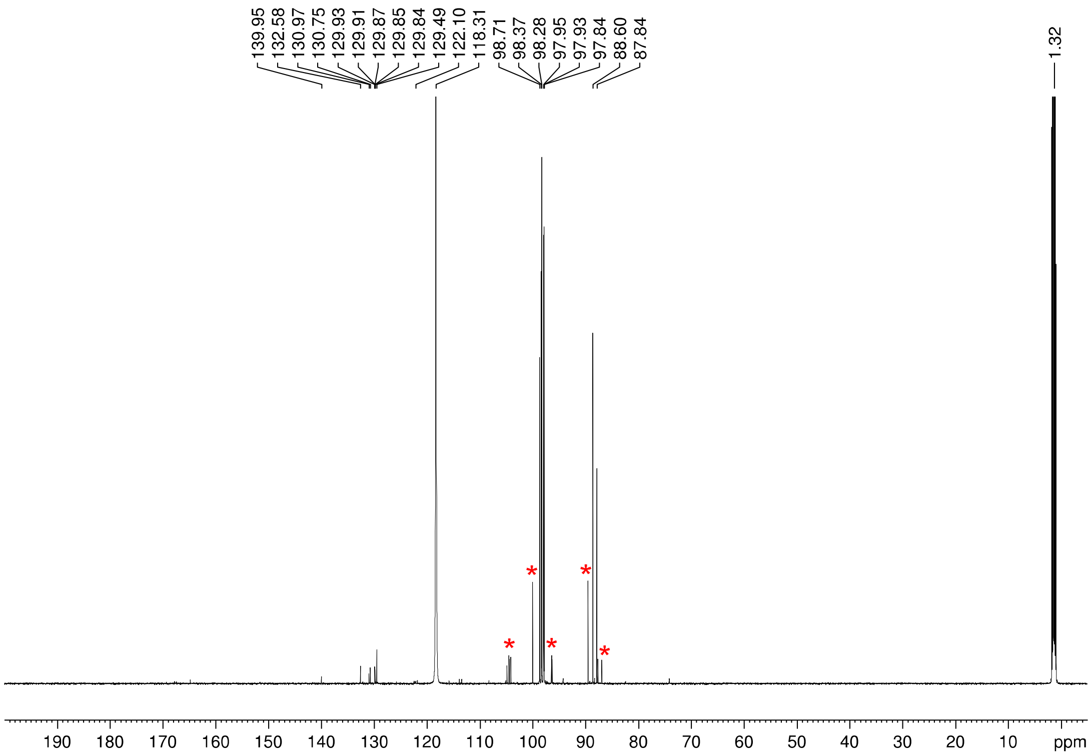

* = ^{13}C-labeled impurity

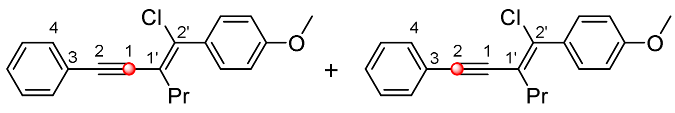

^{13}C(1)-**10a**

2%

^{13}C(2)-**10a**

98%

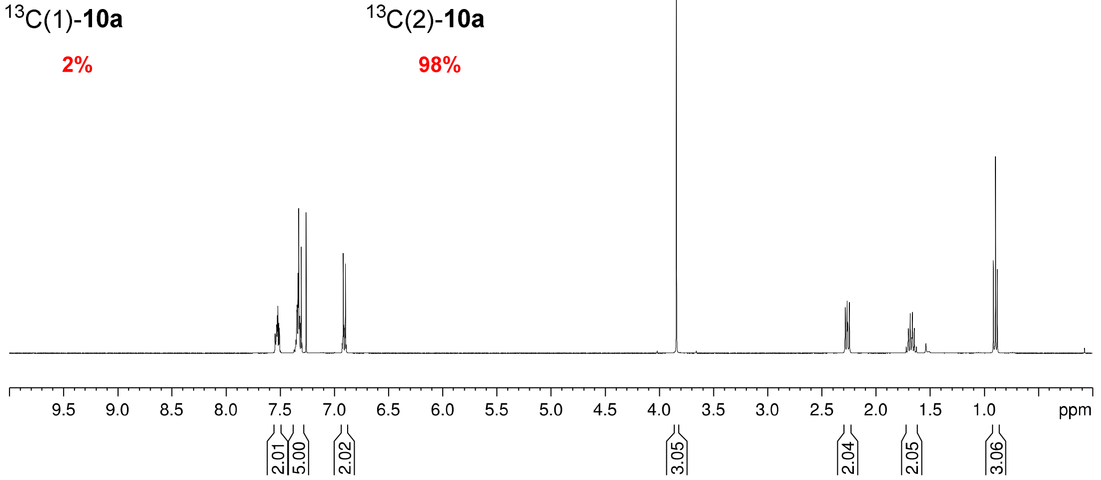

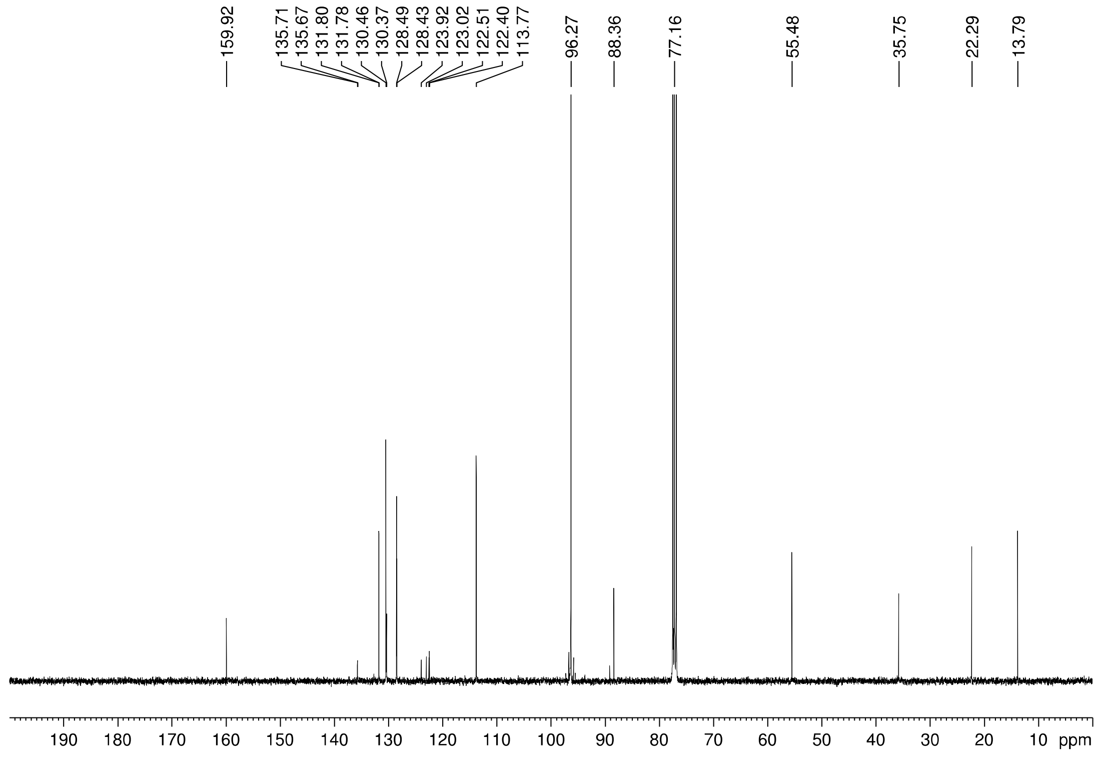

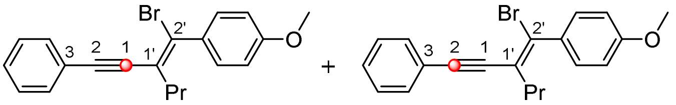

^{13}C(1)-**11a** **13%**

^{13}C(2)-**11a** **87%**

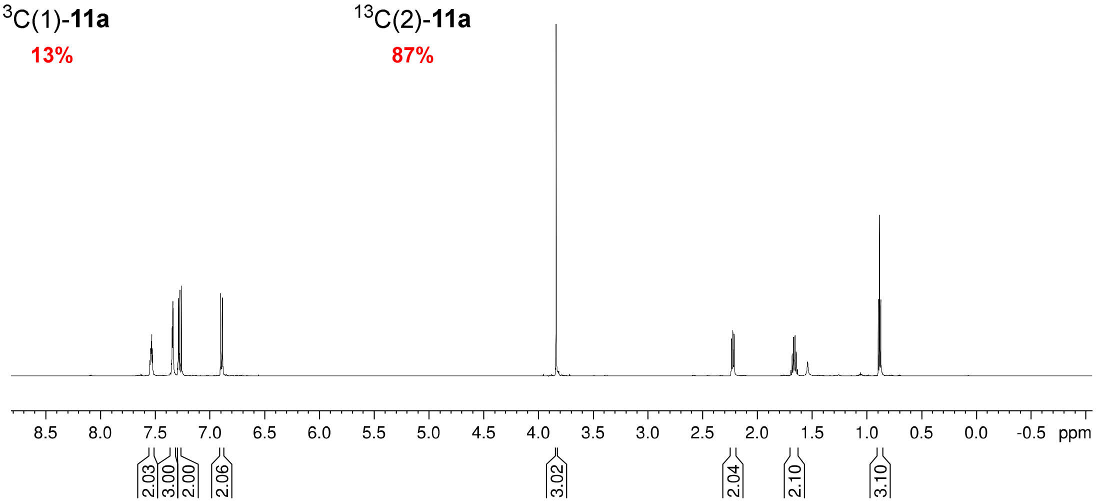

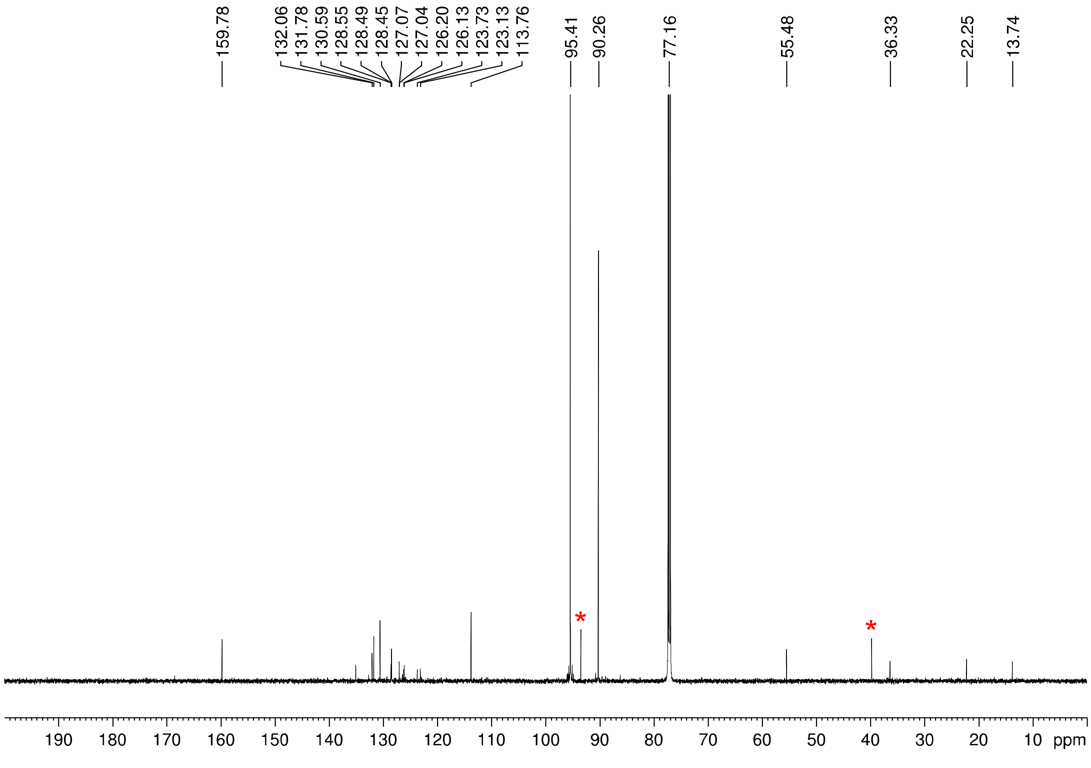

* = ^{13}C-labeled impurity

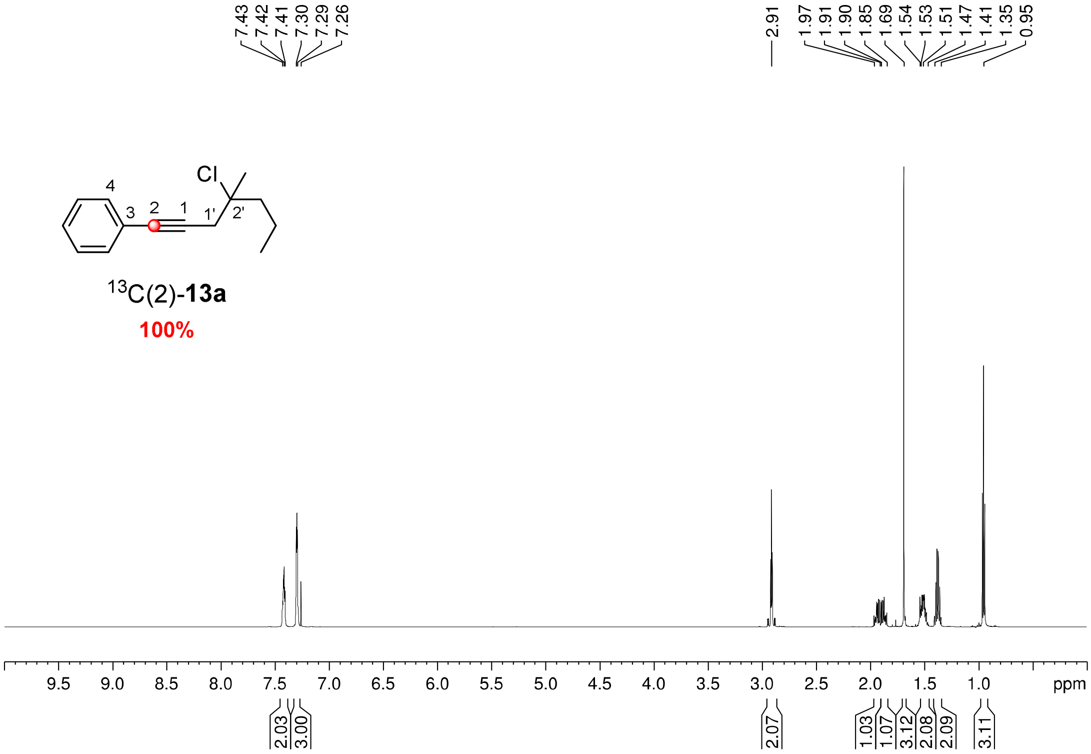

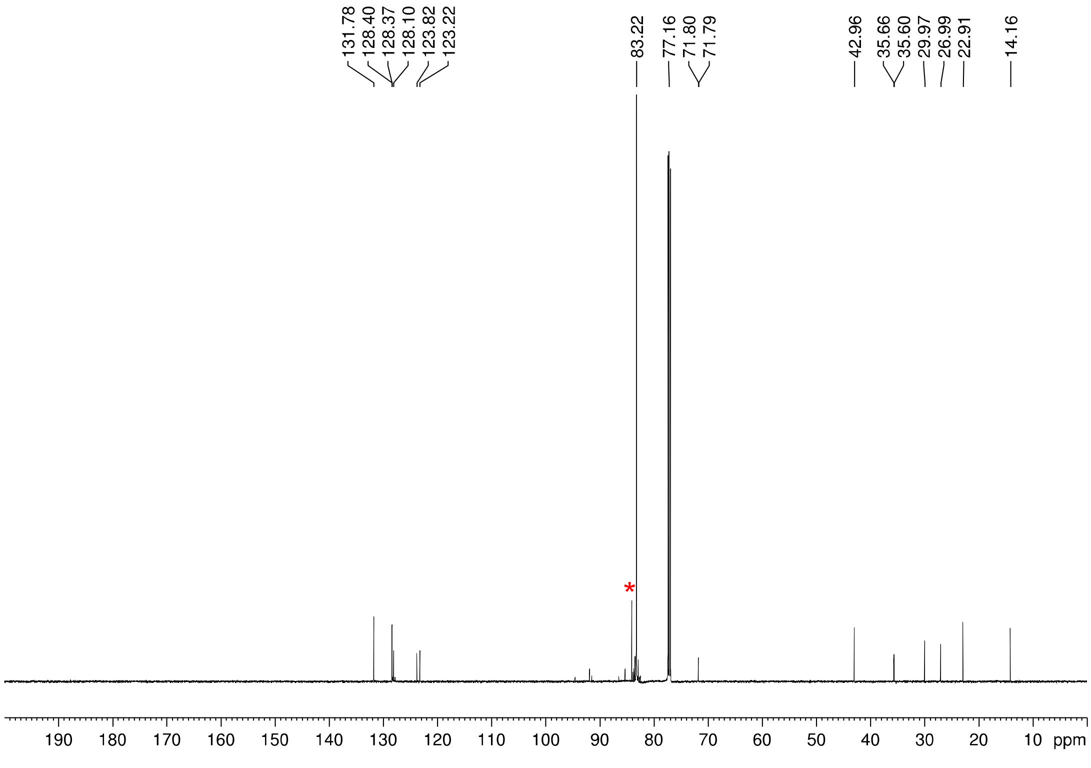

* = ^{13}C-labeled impurity

6. Supporting Information References

[1] M. Kreuzahler, G. Haberhauer, *Angew. Chem.* **2020**, *132*, 9519-9524; *Angew. Chem., Int. Ed.* **2020**, *59*, 9433-9437.

[2] M. E. de Orbe, M. Zanini, O. Quinonero, A. M. Echavarren, *ACS Catal.* **2019**, *9*, 7817-7822.

[3] Z. Zhang, Y. Luo, H. Du, J. Xu, P. Li, *Chem. Sci.* **2019**, *10*, 5156-5161.

[4] M. J. Frisch, G. W. Trucks, H. B. Schlegel, G. E. Scuseria, M. A. Robb, J. R. Cheeseman, G. Scalmani, V. Barone, G. A. Petersson, H. Nakatsuji, X. Li, M. Caricato, A. V. Marenich, J. Bloino, B. G. Janesko, R. Gomperts, B. Mennucci, H. P. Hratchian, J. V. Ortiz, A. F. Izmaylov, J. L. Sonnenberg, D. Williams-Young, F. Ding, F. Lipparini, F. Egidi, J. Goings, B. Peng, A. Petrone, T. Henderson, D. Ranasinghe, V. G. Zakrzewski, J. Gao, N. Rega, G. Zheng, W. Liang, M. Hada, M. Ehara, K. Toyota, R. Fukuda, J. Hasegawa, M. Ishida, T. Nakajima, Y. Honda, O. Kitao, H. Nakai, T. Vreven, K. Throssell, J. A. Montgomery, Jr.; , J. E. Peralta, F. Ogliaro, M. J. Bearpark, J. J. Heyd, E. N. Brothers, K. N. Kudin, V. N. Staroverov, T. A. Keith, R. Kobayashi, J. Normand, K. Raghavachari, A. P. Rendell, J. C. Burant, S. S. Iyengar, J. Tomasi, M. Cossi, J. M. Millam, M. Klene, C. Adamo, R. Cammi, J. W. Ochterski, R. L. Martin, K. Morokuma, O. Farkas, J. B. Foresman, D. J. Fox, *Gaussian 16, Revision A.03*, Gaussian, Inc., Wallingford CT, **2016**.

[5] B. Miehlich, A. Savin, H. Stoll, H. Preuss, *Chem. Phys. Lett.* **1989**, *157*, 200-206.

[6] A. D. Becke, *Phys. Rev. A* **1988**, *38*, 3098-3100.

[7] C. Lee, W. Yang, R. G. Parr, *Phys. Rev. B* **1988**, *37*, 785-789.

[8] S. Grimme, S. Ehrlich, L. Goerigk, *J. Comp. Chem.* **2011**, *32*, 1456-1465.

[9] S. Grimme, *J. Chem. Phys.* **2006**, *124*, 034108.

[10] G. D. Purvis III, R. J. Bartlett, *J. Chem. Phys.* **1982**, *76*, 1910-1918.
